# The Novel Action of miR-193b-3p/CDK1 Signaling in HCC Proliferation and Migration: A Study Based on Bioinformatic Analysis and Experimental Investigation

**DOI:** 10.1155/2022/8755263

**Published:** 2022-12-13

**Authors:** Xue Pang, Wei Wan, Xingxing Wu, Yu Shen

**Affiliations:** ^1^Department of Anesthesiology, The Second People's Hospital of Lianyungang, Lianyungang, China; ^2^Department of Hepatobiliary Surgery, The Second People's Hospital of Lianyungang, Lianyungang, China; ^3^Department of Pediatric Surgery, The Second People's Hospital of Lianyungang, Lianyungang, China

## Abstract

Hepatocellular carcinoma (HCC) is a common human malignancy with high mortality and dismal prognosis. A growing number of novel targets underlying HCC pathophysiology have been detected using microarray high throughput screening platforms. This study carried out bioinformatics analysis to explore underlying biomarkers in HCC and assessed the potential action of the miR-193b-3p/CDK1 signaling pathway in HCC progression. A total of 241 common differentially expressed genes (DEGs) were screened from GSE33294, GSE104310, and GSE144269. Functional analysis results implicated that DEGs are significantly associated with “cell cycle,” “cell division,” and “proliferation.” The protein–protein interaction network analysis extracted ten hub genes from common DEGs. Ten hub genes were significantly overexpression in HCC tissues. Kaplan–Meier survival analysis revealed that 10 hub genes were linked with a poorer prognosis in HCC patients. Functional assays showed that CDK1 knockdown repressed HCC cell proliferation and migration. Luciferase reporter assay showed that miR-193b-3p could target CDK1 3′ untranslated region, and miR-193b-3p negatively modulated CDK1. Enforced CDK1 expression attenuated miR-193b-3p-modulated suppressive actions on HCC cell proliferation and migration. To summarize, we performed a comprehensive bioinformatics analysis and identified 10 hub genes linked to the prognosis in HCC patients. Functional analysis revealed that CDK1, negatively regulated by miR-193b-3p, may act as an oncogene to promote HCC cell proliferation and migration and may predict poor prognosis of HCC patients. However, the role of CDK1/miR-193b-3p may still require further investigation.

## 1. Introduction

Hepatocellular carcinoma (HCC) is a common human malignancy with high mortality and dismal prognosis [1, 2]. Studies have found that viral hepatitis infection is a risk factor contributing to HCC development [3–6]. Early-stage HCC patients often have a 5-year overall survival (OS) rate of about 30%, whereas for the patients with distant metastases, the rate drops to around 5% [7, 8]. For the treatment of HCC, surgical resection is the primary therapeutic option for HCC at its early stages, whereas patients with advanced-stage HCC often were unsuitable for surgical resection. So far, though significant improvements have been made in targeted therapy in the clinical aspects, the OS of patients with HCC remains poor due to the insufficient understanding of molecular mechanisms underlying HCC pathophysiology [7, 8]. Therefore, it is necessary to understand its underlying mechanisms to design valuable and effective strategies.

Recently, genome-wide studies using microarray high throughput technology have significantly advanced our understating of the genetic landscape and driver pathways contributing to the HCC development [9–14]. Chen et al. conducted a tissue microarray analysis and identified a correlation between ALKBH5 and HCC progression [15]. Hamdane et al. proposed that the increased risk of HCC is affected by epigenetic alterations [16]. Lu et al. performed the long noncoding RNA (lncRNA) and mRNA profiling using RNA sequencing and found that lncRNA TSPAN12 acted as a potential microvascular invasion-related biomarker in HCC [17]. Huang and co-workers performed the integrated analysis of RAN-Seq data of HCC. They identified the intrinsic gene expression pattern of HCC, which provides a novel perspective to understanding the heterogeneity of pathogenesis in the HCC tumorigenesis [18]. Thus, more studies may be performed on microarray datasets to reveal novel potential targets related to HCC pathophysiology.

MicroRNA (miRNA) belongs to a class of small non-coding RNA with 21–30 nucleotides in length [19, 20]. miRNA can negatively regulate target genes by binding to the 3′ untranslated region (UTR) of the mRNAs [19]. Due to this mechanism, miRNAs have been found to regulate the HCC pathophysiology via distinct mechanisms [21]. Komoll et al. showed that miR-342-3p acted as a potent tumor suppressor in HCC [22]. Zhou et al. demonstrated that the miR-28-5p-interleukin-34-macrophage feedback loop could regulate the HCC metastasis [23]. Bhattacharya et al. found that serum miR-30e and miR-223 could act as novel noninvasive biomarkers for HCC [24]. Recently, studies also demonstrated that upregulation of miR-520c-3p via hepatitis B virus enhanced HCC migration and invasion by the PTEN/AKT/NF-*κ*B axis [25]. Therefore, further understanding the involvement of miRNAs in HCC progression is of great importance for developing novel therapies.

This study utilized the Gene Expression Omnibus (GEO) datasets (GSE33294, GSE104310, and GSE144269) to extract differentially expressed genes (DEGs) in the liver tissues between HCC and non-tumor groups. Overlapping DEGs among GSE33294, GSE104310, and GSE144269 were utilized for functional analysis and protein–protein interaction (PPI) network establishment. Identified hub genes were further subjected to survival analysis of patients with HCC. Besides, the biological functions of CDK1 (one hub gene) were examined by experimental assays, and its regulatory miRNA was also determined. The present study may advance our understanding of HCC progression and prognosis.

## 2. Materials and Methods

### 2.1. Microarray Datasets Extraction

RNA-sequencing datasets (GSE33294, GSE104310, and GSE144269) were extracted from GEO. For GSE33294, HCC tissues (*n* = 3) and non-tumor tissues (*n* = 3) were utilized in this dataset; for GSE104310, HCC tissues (*n* = 12) and control tissues (*n* = 8) were used, and GPL16791 Illumina HiSeq 2500 (*Homo sapiens*) platform was used; for GSE144269, HCC tissues (*n* = 70) and non-tumor tissues (*n* = 70) were utilized, and GPL24676 Illumina NovaSeq 6000 (*Homo sapiens*) was used.

### 2.2. DEGs Extractions

Gene expression sets in GSE33294, GSE104310, and GSE144269 were extracted using Geo RNA-seq experiments Interactive Navigator (GREIN), an online pipeline for analyzing the GEO datasets uniformly. The DEGs were selected as false discovery rate <0.05, −2 > log (fold change) > 2. Common DEGs among GSE33294, GSE104310, and GSE144269 were collected for subsequent evaluation.

### 2.3. Functional Analysis of DEGs

The g:Profiler tool [26] was utilized to carry out Gene Ontology (GO) enrichment, Kyoto Encyclopedia of Genes and Genomes (KEGG) pathway, and miRNA pathway enrichment analysis. GO enrichment analysis field included biological processes, cellular components, and molecular function [26]. The KEGG was utilized to understand relevant pathways [26].

### 2.4. PPI Network Establishment

Interactive details of DEGs were evaluated by the STRING tool. Subsequently, Cytoscape (version 3.6.1; https://cytoscape.org/) was utilized to establish the PPI network of DEGs. The submodules from the PPI network were extracted using CytoHubba and Molecular Complex Detection (MCODE) applications.

### 2.5. Hub Gene Expression Analysis

The Gene Expression Profiling Interactive Analysis (GEPIA) database (a newly developed interactive web server for analyzing the RNA sequencing expression data of 9,736 tumors and 8,587 normal samples from the TCGA and the GTEx projects, using a standard processing pipeline) [27] was utilized to explore 10 hub genes expression, and *P* < 0.05 was statistically significant. Protein levels of relevant hub genes were evaluated using Human Protein Atlas database.

### 2.6. Survival Analysis

OS and disease-free survival (DFS) of HCC patients were assessed by GEPIA [27]. Kaplan–Meier curves were plotted using the data from 369 HCC patients. *P* < 0.05 was statistically significant.

### 2.7. Cell Lines and Cell Culture

LO2, HCC-LM3, SMMC7221, and HEK293T cells were purchased from the Chinese Academy of Sciences (Shanghai, China). Cells were cultured in the Dulbecco's modified eagle medium (Sigma, St. Louis, USA) supplemented with 10% fetal bovine serum (Sigma) and were maintained in a humidified atmosphere with 5% CO_2_ at 37°C.

### 2.8. Transfection with Small Interfering RNAs, miRNAs, and Plasmids

CDK1 siRNA (si-CDK1) and scrambled control siRNA (si-NC) were from RiboBio (Guangzhou, China). The miR-193b-3p mimics and its respective negative controls (mimics NC) were also from RiboBio. CDK1-overexpressing vector (pcDNA3.1-CDK1) and the control vector were from GenePharma (Shanghai, China). SMMC7721 and HCC-LM3 cells were transfected/co-transfected with siRNAs, miRNAs, and plasmids using Lipofectamine 2000 (Invitrogen, Waltham, USA) according to the manufacturer's protocol. At 24-hour post-treatment, cells were utilized for subsequent experimental assays.

### 2.9. RNA Isolation and RT-qPCR

RNA extraction from respective cells was carried out TRIzol (TaKaRa, Dalian China). The purity and concentration of the extracted RNA were determined using the spectrophotometer (Sigma). One microgram RNA was transcribed into cDNA using PrimeScript 1st strand cDNA Synthesis Kit (TaKaRa). Real-time PCR was carried out utilizing One Step PrimeScript™ RT-PCR Kit (TaKaRa) with ABI7900 (Applied Biosystems, Foster City, USA). Glyceraldehyde-3-phosphate dehydrogenase was selected for reference control of CDK1, and U6 was chosen for reference control of miR-193b-3p. mRNA and miRNA expression levels were displayed using the 2^−*ΔΔ*Ct^ method. The primer sequences for real-time PCR are presented in Table S1.

### 2.10. Cell Counting Kit-8 (CCK-8) Assay

CCK-8 kit (Beyotime, Beijing, China) was utilized for the determination of cell viability. Briefly, respective cells with corresponding interventions were seeded and cultured for indicated periods. Subsequently, cells were treated with 10 *μ*l CCK-8 reagent for 4 hours. Absorbance value at 450 nm was utilized to evaluate cell viability.

### 2.11. Bromodeoxyuridine (BrdU) Assay

BrdU staining assay was utilized to measure the proliferative potential of respective cells with corresponding interventions. Briefly, cells after different interventions were subjected to BrdU (10 *μ*g/ml; Sigma-Aldrich) and BrdU incubation for 30 minutes. After rinsing with phosphate-buffered saline, cells were fixed in 4% paraformaldehyde for 20 minutes. Subsequently, cells were probed with the primary antibody against BrdU (1 : 200; Abcam, Cambridge, MA, USA) for 1 hour, followed by probing with Alexa FluorR^®^ 594-conjugated secondary antibody. The 4′,6-diamidino-2-phenylindole (DAPI) (300 nM) was used for nuclear staining. The number of BrdU^+^ cells was determined using a fluorescent microscope.

### 2.12. TdT-UTP Nick End Labeling Assay

TdT-UTP nick-end labeling (TUNEL) assay was carried out with the one-step TUNEL kit (Elabscience, Wuhan, China) according to the manufacturer's instructions. Briefly, corresponding cells with different interventions were fixed onto poly-(L-lysine)-coated slides with 4% paraformaldehyde. Cells were permeabilized with 0.1% Triton X-100 followed by incubation in 50 ml TUNEL reaction mixture for 1 hour at 37°C in the dark. Next, DAPI was added and incubated for 2 minutes at room temperature for the staining of the nucleus. Cells exhibiting green fluorescence were defined as TUNEL-positive, apoptotic cells.

### 2.13. Caspase-3/-7 Activity Assay

The caspase-Glo assay kit (Promega, Madison, USA) was utilized to determine caspase-3/-7 activity. Briefly, corresponding cells with respective interventions were incubated with caspase-Glo for 2 hours according to the manufacturer's protocol. HCC cell caspase-3/-7 activity was determined by measuring the luminescence.

### 2.14. Wound Healing Assay

A wound healing assay was utilized to measure HCC cell migratory potential. Briefly, HCC cells after different treatments were seeded onto six-well plates. After cells grew to about 80% confluence, a wound was generated utilizing a sterile pipette tip to scratch the cellular monolayer. Wound healing was evaluated for 24 hours. Wound width was measured at 0 and 24 hours, respectively. The wound closure was calculated as follows: (width of the wound at 0 hour − width of the wound at 24 hours)/width of the wound at 0 hour.

### 2.15. Luciferase Reporter Assay

The binding sites between miR-193b-3p and CKD1 3′UTR were predicted using the TargetScan tool (https://www.targetscan.org/vert_80/). The CKD1 3′UTR fragment was amplified from the genomic DNA, and mutant CDK1 3′UTR was generated by a site-directed mutagenesis kit (Thermo Fisher Scientific, Waltham, USA). CDK1 3′UTR containing wild-type (WT) and mutant (MUT) of miR-193b-3p binding sites was subcloned into the pmirGLO vector (Promega). SMMC7721 cells were co-transfected with WT or MUT vector and mimics NC or miR-193b-3p mimics. Cells were harvested 48 hours after co-transfection. A Dual-Glo luciferase assay kit (Promega) was utilized to evaluate luciferase activity. The firefly luciferase activity was normalized to Renilla luciferase activity.

### 2.16. Statistical Analysis

Statistical analysis was performed using GraphPad Prism software V7.0 (GraphPad Software, La Jolla, CA, USA). Unpaired Student's *t*-test or one-way ANOVA was utilized for evaluating the significance of differences between/among different groups. *P* < 0.05 was statistically significant.

## 3. Results

### 3.1. DEGs Extraction from GSE33294, GSE104310, and GSE144269 Datasets

**Figure 1:**
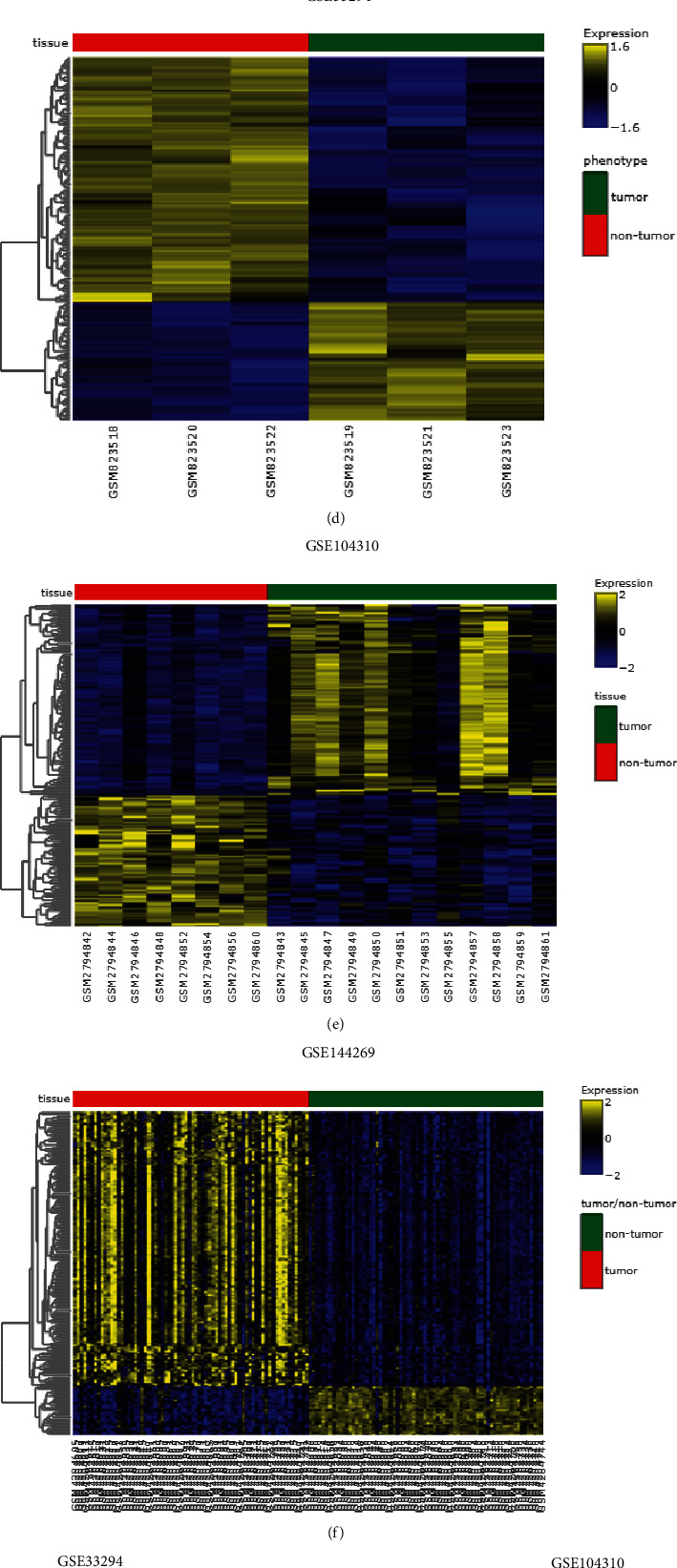
Analysis of DEGs from GSE33294, GSE104310, and GSE144269. PCA analysis of GSE33294(a), GSE104310(b), and GSE144269(c). Heatmap shows the DEGs ranked by adjusted *P* values in GSE33294(d), GSE104310(e), and GSE144269(f). Volcano plots of DEGs in GSE33294(g), GSE104310(h), and GSE144269(i). UP = significantly up-regulated genes; DOWN = significantly down-regulated genes; NOT = not significantly expressed genes.

**Figure 2:**
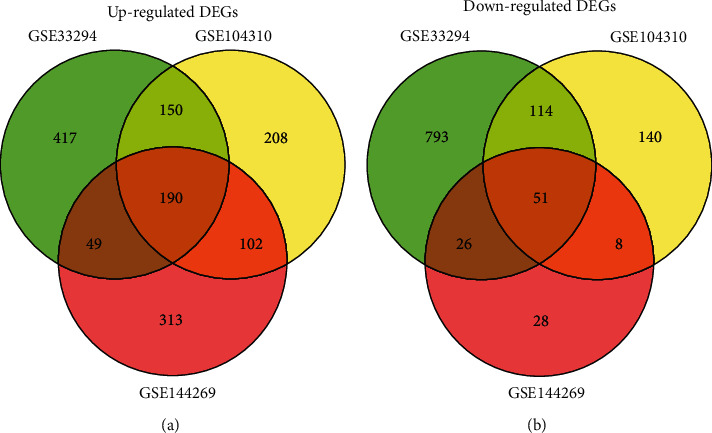
Venn diagram of common DEGs among GSE33294, GSE104310, and GSE144269. (a) Venn diagram shows the common up-regulated DEGs among GSE33294, GSE104310, and GSE144269. (b) Venn diagram shows the common down-regulated DEGs among GSE33294, GSE104310, and GSE144269.

The PCA analysis of GSE33294, GSE104310, and GSE144269 was shown in Figures 1(a), 1(b), and 1(c). In GSE33294, DEGs between 3 normal tissues and 3 HCC tissues were analyzed; in GSE104310, DEGs between 8 normal tissues and 12 HCC tissues were analyzed; and in GSE144269, DEGs between 140 normal tissues and 140 HCC tissues were analyzed. The top 200 DEGs of GSE33294, GSE104310, and GSE144269 ranked by adjusted *P*-value were shown as heatmaps (Figures 1(d), 1(e), and 1(f)). The DEGs in the GSE33294, GSE104310, and GSE144269 were presented as volcano plots (Figures 1(g), 1(h), and 1(i)). In GSE33294, 1790 DEGs (806 up-regulated; 984 down-regulated) were detected; in GSE104310, 863 DEGs (550 up-regulated; 313 down-regulated) were detected; and in GSE144269, 767 DEGs (654 up-regulated; 113 down-regulated) were detected. Common DEGs among collected datasets were displayed in Venn diagrams. One hundred ninety up-regulated and 51 down-regulated DEGs among the datasets were shown (Figures 2(a) and 2(b)).

### 3.2. GO and KEGG Analysis of DEGs among GSE33294, GSE104310, and GSE144269

**Figure 3:**
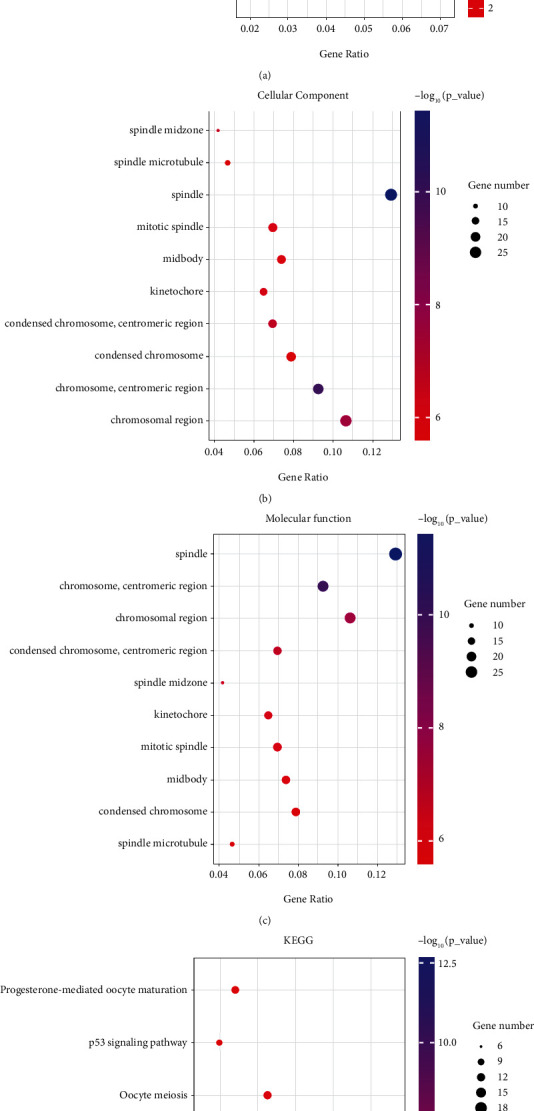
GO and KEGG enrichment analyses of DEGs among GSE33294, GSE104310, and GSE144269. (a) GO: biological process enrichment analysis of DEGs among GSE33294, GSE104310, and GSE144269. (b) GO: cellular component enrichment analysis of DEGs among GSE33294, GSE104310, and GSE144269. (c) GO: molecular function enrichment analysis of DEGs among GSE33294, GSE104310, and GSE144269. (d) KEGG pathway enrichment analysis of DEGs among GSE33294, GSE104310, and GSE144269.

In GO enrichment analysis under biological process, the DEGs were mainly classified into “tubulin binding,” “motor activity,” “microtubule motor activity,” “microtubule binding,” and so on (Figure 3(a)); in cellular component, DEGs were mainly classified into “spindle midzone,” “spindle microtubule,” “spindle,” and “mitotic spindle” (Figure 3(b)); in molecular function, DEGs were mainly classified into “spindle,” “chromosome, centromeric region,” and “chromosomal region” (Figure 3(c)). In KEGG enrichment analysis, DEGs were mainly classified into “progesterone-mediated oocyte maturation,” “p53 signaling pathway,” “oocyte meiosis,” “cellular senescence,” “cell cycle,” and “bladder cancer” (Figure 3(d)).

### 3.3. PPI Network

**Figure 4:**
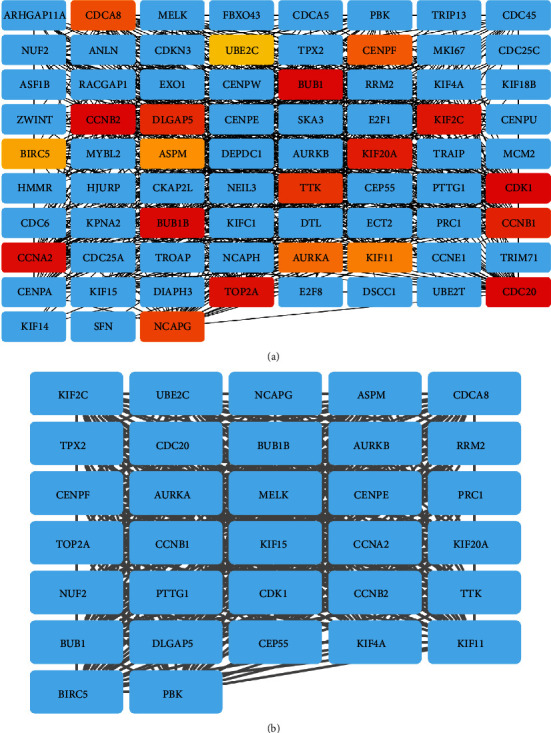
PPI network analysis of DEGs. (a) PPI network constructed by using CytoHubba. (b) PPI network established by MCODE.

Common DEGs were further processed by using PPI network analysis. The network was first established with the STRING database and then visualized by the Cytoscape software. Furthermore, sub-modules of the network were derived using CytoHubba and MCODE applications. As shown in Figure 4(a), a total of 72 DEGs was included in the submodule as analyzed by CytoHubba; based on the MCODE, a total of 7 submodules was detected (Figure 4(b) and Figure S1), and the submodule with the highest score had 32 DEGs (Figure 4(b)). The top 10 overlapped DEGs (CDK1, CDC20, CCNB2, BUB1, BUB1B, CCNA2, TOP2A, KIF2C, KIF20A, and CCNB1) between CytoHubba and MCODE analysis were chosen as hub genes.

### 3.4. The Expression and Survival Analysis of Hub Genes in HCC

**Figure 5:**
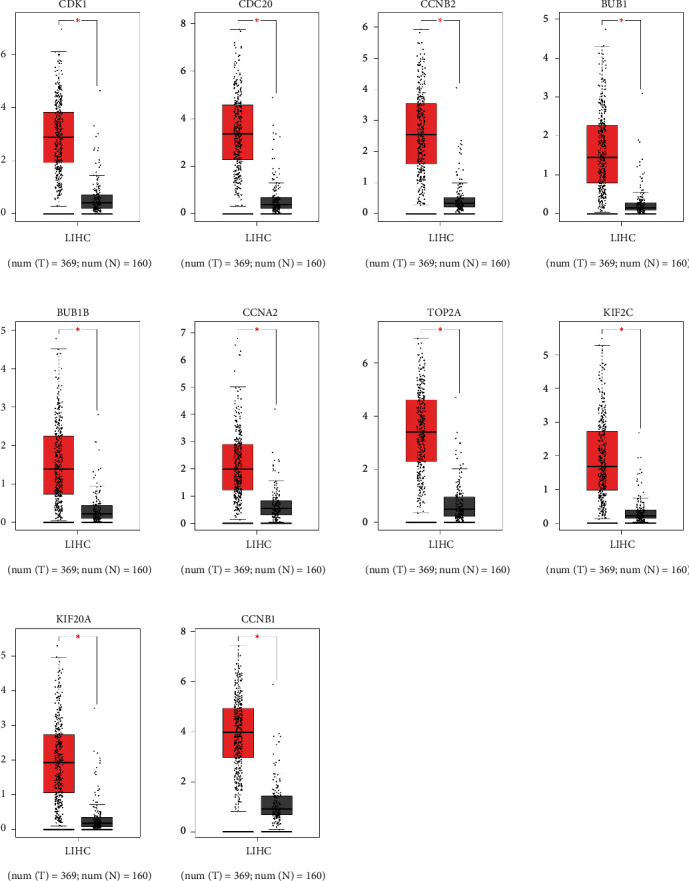
Expression of DEGs in HCC and control tissues. Expression of CDK1, CDC20, CCNB2, BUB1, BUB1B, CCNA2, TOP2A, KIF2C, KIF20A, and CCNB1 in HCC and control tissues were analyzed by GEPIA. LIHC = liver hepatocellular carcinoma.

**Figure 6:**
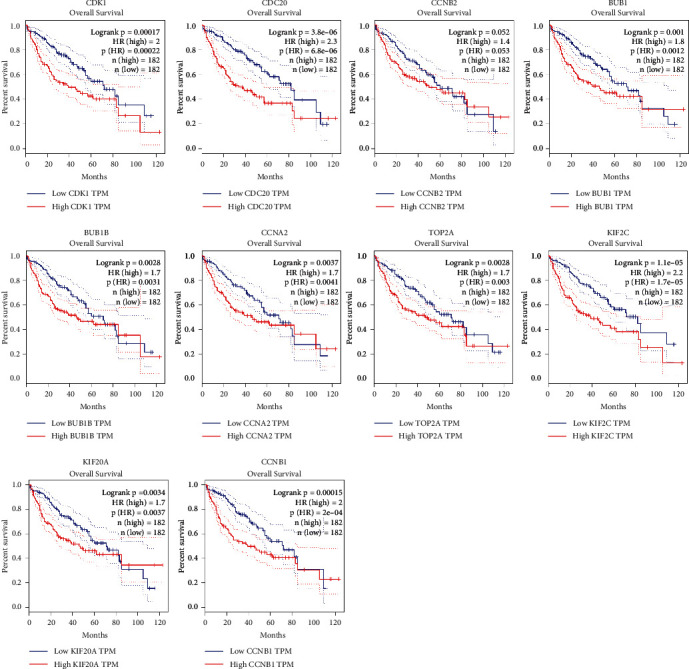
The link between CDK1, CDC20, CCNB2, BUB1, BUB1B, CCNA2, TOP2A, KIF2C, KIF20A, and CCNB1 expression and OS of HCC patients was determined by GEPIA.

**Figure 7:**
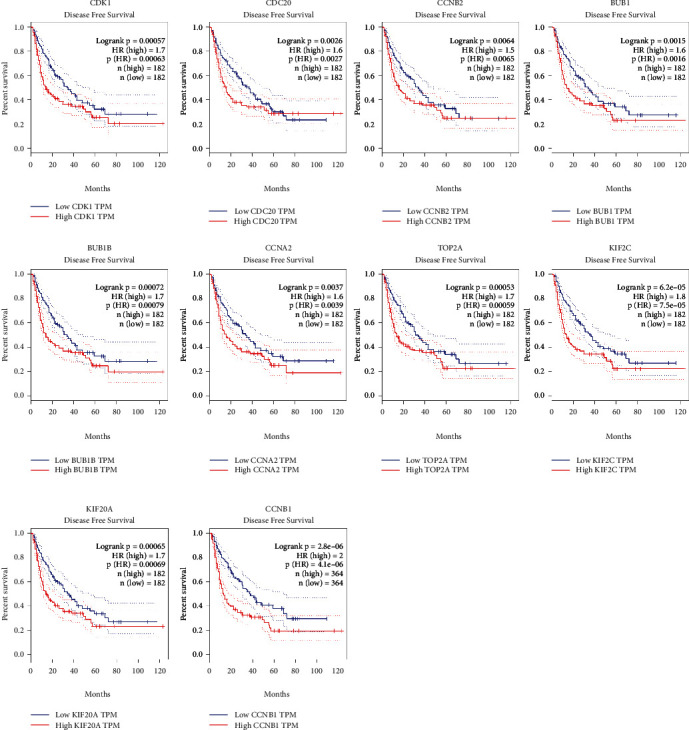
The link between CDK1, CDC20, CCNB2, BUB1, BUB1B, CCNA2, TOP2A, KIF2C, KIF20A, and CCNB1 expression and DFS of HCC patients was determined by GEPIA.

**Figure 8:**
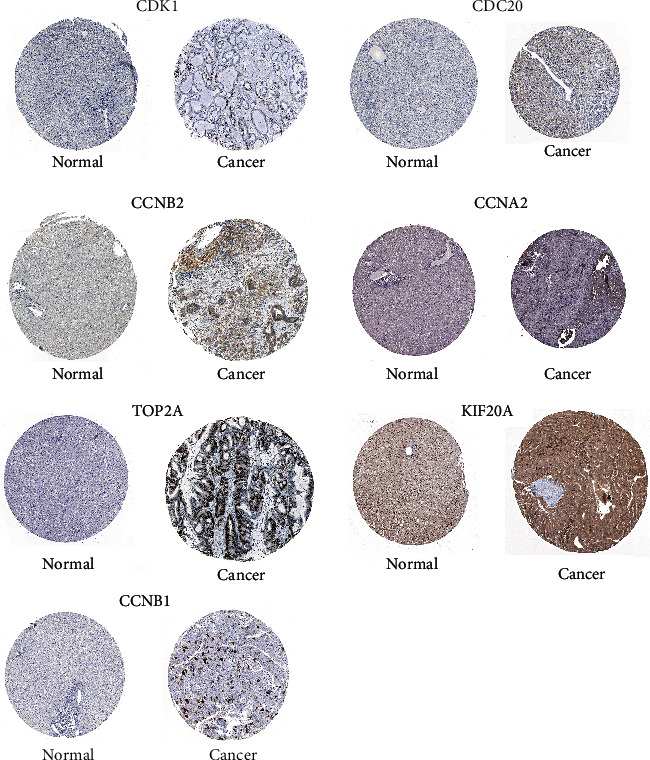
Protein expression of CDK1, CDC20, CCNB2, CCNA2, TOP2A, KIF20A, and CCNB2 in HCC and control tissues was evaluated using Human Protein Atlas.

Ten hub genes in control and HCC tissues were analyzed using the GEPIA tool.  Figure 5 showed that CDK1, BUB1, CDC20, CCNB2, BUB1B, CCNA2, TOP2A, KIF2C, KIF20A, and CCNB1 were all overexpressed in HCC tissues compared to controls (Figure 5). The link between hub genes and OS of HCC patients was analyzed by the GEPIA tool. High expression of CDK1, CDC20, BUB1, BUB1B, CCNA2, TOP2A, KIF2C, KIF20A, and CCNB1, but not CCNB2 was significantly associated with shorter OS of HCC patients (Figure 6). Furthermore, the link between hub genes and DFS of HCC patients was determined by the GEPIA tool. High expression of CDK1, CDC20, BUB1, BUB1B, CCNA2, TOP2A, KIF2C, KIF20A, CCNB1, and CCNB2 was significantly associated with shorter DFS in HCC patients (Figure 7). Protein expression of CDK1, CDC20, CCNB2, CCNA2, TOP2A, KIF20A, and CCNB1 in HCC and control tissues was evaluated by Human Protein Atlas, and results showed the up-regulation of CDK1, CDC20, CCNB2, CCNA2, TOP2A, KIF20A, and CCNB1 protein in HCC tissues compared with controls (Figure 8).

### 3.5. CDK1 Silence Attenuates HCC Cell Proliferation and Migration

**Figure 9:**
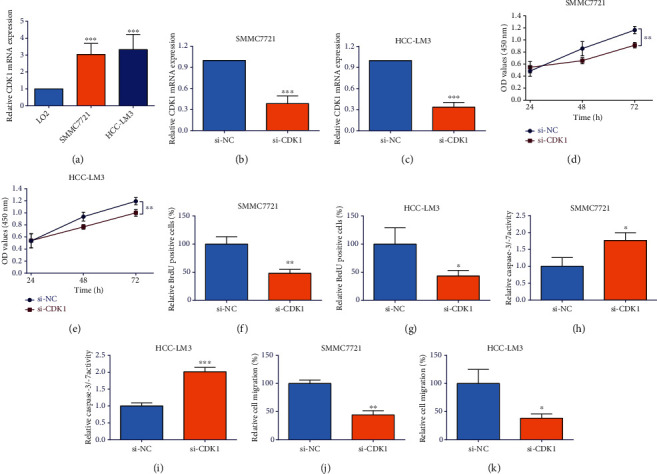
CDK1 silence attenuated HCC cell proliferation and migration. (a) CDK1 expression in the LO2, SMMC7721, and HCC-LM3 cells. (b)–(k) SMMC7721 and HCC-LM3 cells were transfected with si-CDK1 or si-NC, (b) and (c) the CDK1 mRNA expression in SMMC7721 and HCC-LM3 cells; (d) and (e) cell viability of SMMC7721 and HCC-LM3 cells; (f) and (g) cell proliferation of SMMC7721 and HCC-LM3 cells; (h) and (i) caspase-3/-7 activities of SMMC7721 and HCC-LM3 cells; (j) and (k) the cell migration of SMMC7721 and HCC-LM3 cells. *N* = 3; ∗*P* < 0.05, ∗∗*P* < 0.01, and ∗∗∗*P* < 0.001.

CDK1 has the highest score as a hub gene based on the CytoHubba analysis, so CDK1 was chosen for further in vitro functional studies. As shown in Figure 9(a), CDK1 was up-regulated in the SMMC7721 and HCC-LM3 cells compared with that in LO2. Furthermore, the down-regulation of CDK1 was achieved by treating HCC cells with CDK1 siRNA. CDK1 siRNA transfection repressed CDK1 expression (Figures 9(b) and 9(c)). The CCK-8 and BrdU assays showed that CDK1 knockdown reduced the HCC cell viability and proliferation (Figures 9(d), 9(e), 9(f), and 9(g)). For the caspase-3/-7 activity, CDK1 knockdown significantly elevated HCC cell caspase-3/-7 activity (Figures 9(h) and 9(i)). The wound healing assay showed that CDK1 silence significantly attenuated the HCC cell migratory potentials (Figures 9(j) and 9(k)).

### 3.6. miR-193b-3p Negatively Regulated CDK1

**Figure 10:**
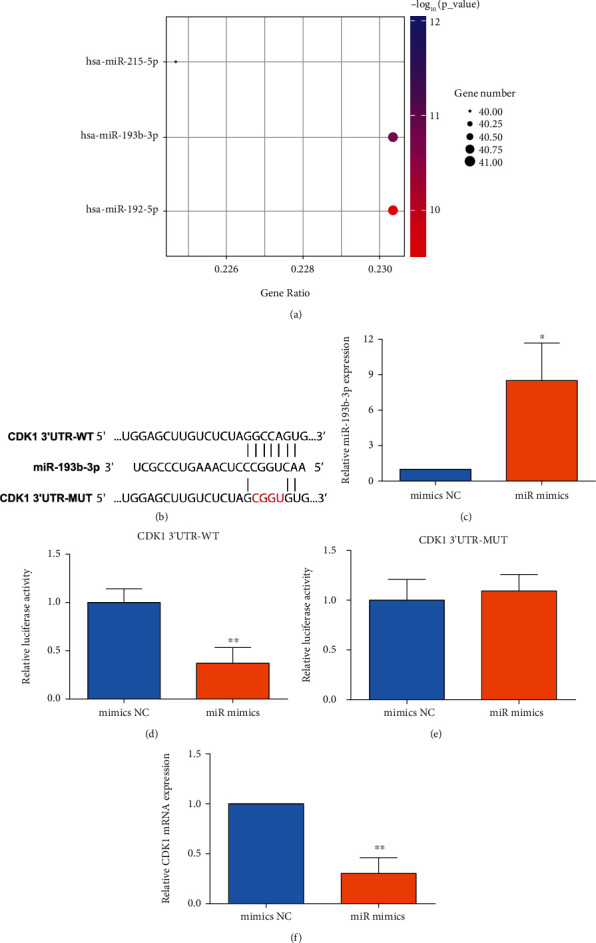
miR-193b-3p negatively modulated CDK1. (a) miRNA enrichment analysis of DEGs. (b) The predicted binding location between miR-193b-3p and CDK1 3′UTR. (c)–(e) The HEK293T cells were transfected with mimics NC or miR-193b-3p mimics, and relative expression of miR-193b-3p in HEK293T cells was measured by qRT-PCR; (d) relative luciferase activity of wild-type (WT) CDK1 3′UTR reporter vector in HEK293T cells was evaluated by Dual-Luciferase reporter assay kit; (e) the relative luciferase activity of mutant (MUT) CDK1 3′UTR reporter vector in HEK293T cells was determined by the Dual-Luciferase reporter assay kit; and (f) mRNA expression level of CDK1 in the SMMC7721 cells. *N* = 3; ∗∗*P* < 0.01 and ∗∗∗*P* < 0.001.

Based on the miRNA pathway enrichment analysis, miR-215-5p, miR-193b-3p, and miR-192-5p could target most overlapping DEGs, and CDK1 showed the potential interaction with miR-193b-3p (Figure 10(a)). The online bioinformatics analysis using the TargetScan tool was used to predict the binding sites between miR-193b-3p and CDK1, and CDK1 3′UTR harbored the binding sites for miR-193b-3p (Figure 10(b)). The luciferase reporter assay showed that miR-193b-3p overexpression significantly repressed the luciferase activity of WT CDK1 3′UTR reporter vector, but not the mutant CDK1 3′UTR in HEK293T cells (Figures 10(c), 10(d), and 10(e)). In addition, miR-193b-3p overexpression down-regulated the mRNA expression of CDK1 in SMMC7721 cells (Figure 10(f)). For the upstream targets of miR-193b-3p, we further predicted potential circular RNAs and long non-coding RNAs that may interact with miR-193b-3p by using the starBASE v2.0 tool [28]. The predicted circular RNAs and long coding RNAs were summarized in Tables S2 and S3, respectively.

### 3.7. CDK1 Overexpression Attenuated miR-193b-3p-Mediated Effects on the HCC Cell Proliferation and Migration

**Figure 11:**
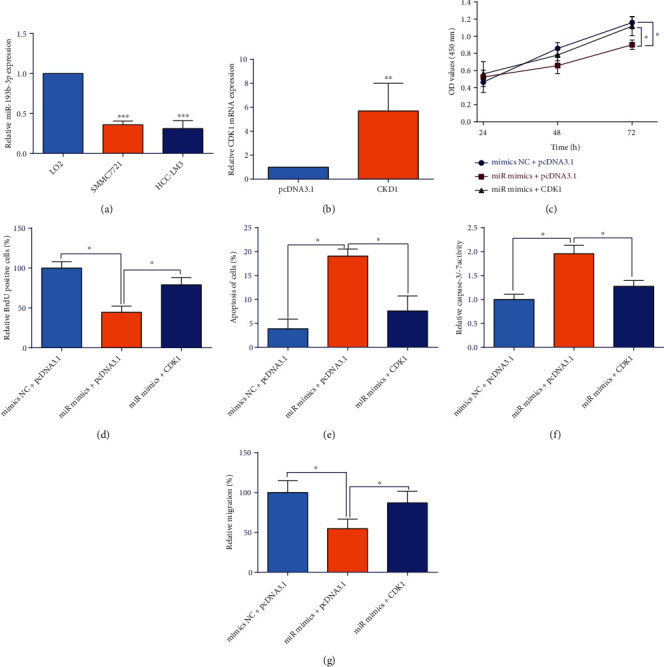
CDK1 overexpression attenuated miR-193b-3p-modulated actions on the HCC cell proliferation and migration. (a) Relative expression of miR-193b-3p in LO2, SMMC7721, and HCC-LM3. (b) SMMC7721 cells were transfected with pcDNA3.1 or pcDNA3.1-CDK1. (c)–(f) SMMC7721 cells were co-transfected with miRNAs and plasmids, and at 24 hours after co-transfection, (c) cell viability of SMMC7721 cells; (d) cell proliferation of SMMC7721 cells; (e) cell apoptosis of SMMC7721 cells as determined by TUNEL assay; (f) the caspase-3/-7 activities of SMMC7721 cells; and (g) cell migration of SMMC7721 cells. *N* = 3; ∗*P* < 0.05, ∗∗*P* < 0.01, and ∗∗∗*P* < 0.001.

The expression of miR-193b-3p in the LO2, SMMC7721, and HCC-LM3 cells was evaluated by qRT-PCR, and the expression level of miR-193b-3p was significantly down-regulated in the SMMC7721 and HCC-LM3 cells when compared with LO2 cells (Figure 11(a)). The functional experiments showed that miR-193b-3p overexpression significantly repressed cell viability, cell proliferation, and migration but increased the number of apoptotic cells and enhanced the caspase-3/-7 activity of SMMC7721 cells, which was significantly reversed by the enforced expression of CDK1 (Figures 11(b), 11(c), 11(d), 11(e), 11(f), and 11(g)).

## 4. Discussion

HCC patient survival is poor owing to insufficient diagnostic and effective strategy HCC [1]. Using microarray high throughput screening platforms, a growing number of novel targets underlying HCC pathophysiology have been identified [12]. This analyzed GSE33294, GSE104310, and GSE144269 datasets and found 241 common DEGs. Functional analysis revealed essential pathways of DEGs. Ten hub genes from DEGs were detected from the PPI network. TGCA database analysis showed that the high expression of ten hub genes was closely linked to a worse prognosis for HCC patients. Functional assays indicated that CDK1 knockdown repressed the HCC cell proliferation and migration; CDK1 was negatively modulated by miR-193b-3p and enforced CKD1 expression attenuated the miR-193b-3p-mediated suppressive actions on the HCC cell proliferation and migration. The present study highlighted the underlying action of miR-193b-3p/CDK1 signaling in HCC pathophysiology.

Recently, many GEO microarray datasets have been explored to retrieve novel targets associated with the HCC progression. In the GSE33294, researchers utilized large-scale transcriptomic analysis and revealed the differentially expressed ADARs in HCC [29]. In GSE144296, Candia et al. described molecular features of Mongolian HCC patients using whole-exome and transcriptomic analysis and proposed novel mechanisms of hepatocarcinogenesis in the Mongolians [30]. In the present study, we extracted common DEGs from the above datasets and detected 241 common DEGs, and functional analysis revealed that these common DEGs were mainly classified into pathways associated with cell proliferation and division.

We identified 10 highly connective hub genes based on the PPI network results. Based on the GEPIA tool analysis, ten hub genes were overexpressed in HCC tissues. High expression of these hub genes was linked with poor prognosis of HCC patients. Previous studies have demonstrated that the role of CDK1, CDC20, BUB1, BUB1B, CCNA2, TOP2A, KIF2C, KIF20A, CCNB1, and CCNB2 has been explored in HCC to some extent [31–34]. Among the ten hub genes, we selected CDK1 for further investigation, due to the highest score in the PPI network based on the CytoHubba. Our study found that CDK1 silence impaired HCC cell proliferation and migration, which was consistent with previous studies [31, 32, 34]. Studies also showed that the block of CKD1 could attenuate *in vivo* tumor growth of HCC cells [35]. Furthermore, to further decipher the regulatory action of CDK1 in HCC cell progression, we found that miR-193b-3p targeted most of the common DEGs including CDK1. Thus, we further confirmed the link between miR-193b-3p and CDK1 3′UTR by bioinformatic prediction and luciferase reporter assay. CDK1 was negatively modulated by miR-193b-3p, and enforced CKD1 expression attenuated the miR-193b-3p-mediated suppressive actions on HCC cell proliferation and migration. Tumor-suppressive action of miR-193b-3p has been examined in various types of cancers [36–40]. miR-193b-3p could target various downstream targets, such as MORC4 [40], CCND1 [41], ETS1 [42], MYB [43], and so on. In the *in vivo* studies, miR-193b-3p exerted suppressive effects on the tumor growth of HCC cells in the nude mice [44]. In the clinical aspect, Wang et al. demonstrated that miR-193b-3p was down-regulated in the HCC tissues by analyzing the Cancer Genome Atlas database [45]; Xu et al. demonstrated the down-regulation of miR-139b-3p in most of the HCC tissues compared to the matching non-tumoural liver tissues [44]. However, in our study, we demonstrated that miR-193b-3p targeted CDK1 3′UTR and subsequently repressed the expression of CDK1 in HCC cells. The above evidence suggested the novel miR-193b-3p/CDK1 signaling pathway in modulating the HCC progression.

There are several limitations existing in our work. First, the correlation between miR-193b-3p and CDK1 was only examined in the *in vitro* studies, and further studies should confirm the *in vitro* findings using the clinical samples from HCC patients. Second, the predicted upstream circular RNAs and long non-coding RNAs that may affect miR-193b-3p expression should be confirmed by mechanistic studies.

To summarize, we performed a comprehensive bioinformatics analysis and identified 10 hub genes linked to the prognosis of HCC patients. Functional analysis revealed the novel functional action of miR-193b-3p/CDK1 signaling in HCC pathophysiology.

## Data Availability

Data supporting this research article are available from the corresponding author or first author on reasonable request.

## Supplementary Materials

Supplementary Materials Supplemental Figure S1. Submodule PPI network as determined by MCODE.

Supplementary Materials Supplemental Table S1. Primers sequences for qRT-PCR Supplemental Table S2. Predicted circular RNAs that target miR-193b-3p based on the starBase tool (https://starbase.sysu.edu.cn/). Supplemental Table S3. Predicted long non-coding RNAs that target miR-193b-3p based on the starBase tool (https://starbase.sysu.edu.cn/).
