# Supplementary figures and images for: The Novel Action of miR-193b-3p/CDK1 Signaling in HCC Proliferation and Migration: A Study Based on Bioinformatic Analysis and Experimental Investigation
Source: Int J Genomics. 2022 Dec 13;2022:8755263. doi: 10.1155/2022/8755263 (PMC9806689; doi:10.1155/2022/8755263)

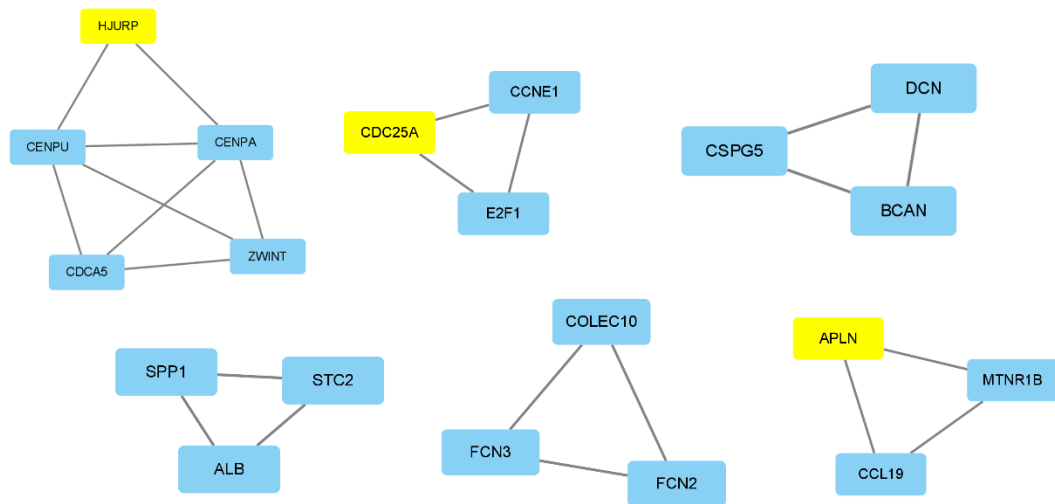

Supplemental Figure S1. Submodule PPI network as determined by MCODE.

Supplement: Supplementary Materials — Supplemental Figure S1. Submodule PPI network as determined by MCODE. [file 8755263.f1.pdf]
