# Supplementary material for: The Novel Action of miR-193b-3p/CDK1 Signaling in HCC Proliferation and Migration: A Study Based on Bioinformatic Analysis and Experimental Investigation
Source: Int J Genomics. 2022 Dec 13;2022:8755263. doi: 10.1155/2022/8755263 (PMC9806689; doi:10.1155/2022/8755263)
Supplement: Supplementary Materials — Supplemental Table S1. Primers sequences for qRT-PCR Supplemental Table S2. Predicted circular RNAs that target miR-193b-3p based on the starBase tool (https://starbase.sysu.edu.cn/). Supplemental Table S3. Predicted long non-coding RNAs that target miR-193b-3p based on the starBase tool (https://starbase.sysu.edu.cn/). [file 8755263.f2.docx]

Supplemental Table S1. Primers sequences for qRT-PCR

| Gene name | Primers sequences |
| --- | --- |
| CDK1 | F: 5’ CAGACTAGAAAGTGAAGAGGAAGG 3’ |
|  | R: 5’ ACTGACCAGGAGGGATAGAATC 3’ |
| GAPDH | F: 5’ CGGAGTCAACGGATTTGGTCGTAT 3’ |
|  | R: 5’ AGCCTTCTCCATGGTGGTGAAGAC 3’ |
| miR-193b-3p | RT: 5' GTCGTATCCAGTGCAGGGTCCGAGGTATTCGCACTGGATACGACAGCGGG 3' |
|  | F: 5' GCGCAACTGGCCCTCAAAGT 3' |
| U6 | RT: 5'GTCGTATCCAGTGCAGGGTCCGAGGTATTCGCA CTGGATACGACAAAATATG 3' |
|  | F: 5' CTCGCTTCGGCAGCACA 3' |
|  | R: 5' AACGCTTCACGAATTTGCGT 3' |

Supplemental Table S2. Predicted circular RNAs that target miR-193b-3p based on the starBase tool (<https://starbase.sysu.edu.cn/>).

| geneID | geneName | geneType | chromosome | start | end | strand | clipExpNum | degraExpNum | RBP | merClass | miRseq | targetSeq |
| --- | --- | --- | --- | --- | --- | --- | --- | --- | --- | --- | --- | --- |
| NM_198576 | AGRN | circRNA | chr1 | 980781 | 980802 | + | 1 | 0 | AGO2 | 7mer-m8 | ucgcccugaaacucCCGGUCAa | gugacccagccacaGGCCAGUg |
| NM_198576 | AGRN | circRNA | chr1 | 982080 | 982100 | + | 1 | 0 | AGO1-4 | 7mer-m8 | ucgCCCUGAAACUCCCGGUCAa | gcgGGGACCAGGA-GGCCAGUg |
| hsa_circ_0000006 | hsa_circ_001621 | circRNA | chr1 | 1643282 | 1643303 | - | 1 | 0 | AGO1-4 | 8mer | ucgcccugaaacucCCGGUCAa | gagccaccgcggccGGCCAGUa |
| hsa_circ_0000006 | hsa_circ_001621 | circRNA | chr1 | 1652977 | 1652998 | - | 1 | 0 | AGO1-4 | 7mer-m8 | ucgcccugaaacucCCGGUCAa | cacagcccuggccuGGCCAGUc |
| NM_002074 | GNB1 | circRNA | chr1 | 1717072 | 1717093 | - | 16 | 2 | AGO1-4,AGO2 | 7mer-m8 | ucgcccugAAACUCCCGGUCAa | gguguucaUUUCAAGGCCAGUg |
| NM_015215 | CAMTA1 | circRNA | chr1 | 7805018 | 7805039 | + | 1 | 0 | AGO2 | 7mer-m8 | ucgccCUGAAACUCCCGGUCAa | acaauGAGCUGGCUGGCCAGUu |
| NM_004285 | H6PD | circRNA | chr1 | 9322289 | 9322310 | + | 1 | 0 | AGO1-4 | 8mer | ucgCCCUGAAACUCCCGGUCAa | gcaGUGCCGUCGUGGGCCAGUa |
| NM_001009566 | CLSTN1 | circRNA | chr1 | 9790464 | 9790485 | - | 7 | 0 | AGO1-4,AGO2 | 8mer | ucgcccugaaacucCCGGUCAa | uacaaagucauuucGGCCAGUa |
| NM_015074 | KIF1B | circRNA | chr1 | 10397530 | 10397551 | + | 2 | 0 | AGO2 | 7mer-m8 | ucgcccugaAACUCCCGGUCAa | guaacagugUUGCAGGCCAGUg |
| NM_015074 | KIF1B | circRNA | chr1 | 10440849 | 10440870 | + | 12 | 0 | AGO1,AGO1-4,AGO2,AGO3 | 7mer-m8 | ucgcccugaaacUCCCGGUCAa | caguuguucaccAGGGCCAGUg |
| NM_138346 | KIAA2013 | circRNA | chr1 | 11980169 | 11980190 | - | 4 | 0 | AGO1-4,AGO2 | 7mer-m8 | ucgCCCUGAAACUCCCGGUCAa | gugGGGAGGGAAGGGGCCAGUg |
| NM_001066 | TNFRSF1B | circRNA | chr1 | 12262111 | 12262132 | + | 1 | 0 | AGO2 | 7mer-m8 | ucgcccugaaacucCCGGUCAa | ucccuggagagcucGGCCAGUg |
| NM_001066 | TNFRSF1B | circRNA | chr1 | 12268429 | 12268454 | + | 15 | 0 | AGO1-4,AGO2 | 7mer-m8 | ucgcccUGAAAC----UCCCGGUCAa | ccugccACUUUGGUACAUGGCCAGUg |
| NM_015378 | VPS13D | circRNA | chr1 | 12443097 | 12443118 | + | 1 | 0 | AGO2 | 7mer-m8 | ucGCCCUGAAACUCCCGGUCAa | caUGGAGCUUUUGGGGCCAGUu |
| NM_004753 | DHRS3 | circRNA | chr1 | 12628217 | 12628238 | - | 5 | 0 | AGO1-4,AGO2 | 7mer-m8 | ucgcccugaaacuCCCGGUCAa | uccggcugccccuGGGCCAGUc |
| NM_004753 | DHRS3 | circRNA | chr1 | 12639325 | 12639346 | - | 1 | 0 | AGO2 | 7mer-m8 | ucgcccugaaacuCCCGGUCAa | acaucaacacccuGGGCCAGUu |
| NM_012231 | PRDM2 | circRNA | chr1 | 14104966 | 14104987 | + | 2 | 0 | AGO1-4,AGO2 | 7mer-m8 | ucgcccugaaaCUCCCGGUCAa | acccuccaggaGGUGGCCAGUc |
| NM_012231 | PRDM2 | circRNA | chr1 | 14109217 | 14109237 | + | 2 | 0 | AGO1-4,AGO2 | 7mer-m8 | ucgcCCUGAAACUCCCGGUCAa | gagcGGA-GUGGGGGGCCAGUc |
| NM_015001 | SPEN | circRNA | chr1 | 16260826 | 16260847 | + | 2 | 0 | AGO1-4,AGO2 | 7mer-m8 | ucgcccuGAAACUCCCGGUCAa | gaauguuCUUACGGGGCCAGUg |
| NM_004431 | EPHA2 | circRNA | chr1 | 16464513 | 16464534 | - | 4 | 0 | AGO1-4,AGO2 | 7mer-m8 | ucGCCCUG-AAACUCCCGGUCAa | ugCGGGCCGUGUGA-GGCCAGUg |
| NM_001135248 | MFAP2 | circRNA | chr1 | 17303669 | 17303690 | - | 2 | 0 | AGO1-4,AGO2 | 8mer | ucgcccugaaACUCCCGGUCAa | gcuugcuggcUCAGGGCCAGUa |
| NM_002403 | MFAP2 | circRNA | chr1 | 17303669 | 17303690 | - | 2 | 0 | AGO1-4,AGO2 | 8mer | ucgcccugaaACUCCCGGUCAa | gcuugcuggcUCAGGGCCAGUa |
| NM_020765 | UBR4 | circRNA | chr1 | 19490808 | 19490829 | - | 2 | 0 | AGO1-4 | 7mer-m8 | ucgcccugaaacucCCGGUCAa | gugauggccacucuGGCCAGUg |
| NR_038125 | CAPZB | circRNA | chr1 | 19775785 | 19775806 | - | 1 | 0 | AGO2 | 7mer-m8 | ucgcccugaaacucCCGGUCAa | cucgcaucucgccaGGCCAGUg |
| NM_016287 | HP1BP3 | circRNA | chr1 | 21083699 | 21083720 | - | 4 | 0 | AGO1,AGO1-4 | 7mer-m8 | ucgcccugaaacUCCCGGUCAa | gagcaguagagaGGGGCCAGUu |
| hsa_circ_0000023 | hsa_circ_000809 | circRNA | chr1 | 21083699 | 21083720 | - | 4 | 0 | AGO1,AGO1-4 | 7mer-m8 | ucgcccugaaacUCCCGGUCAa | gagcaguagagaGGGGCCAGUu |
| hsa_circ_0000024 | hsa_circ_001228 | circRNA | chr1 | 21083699 | 21083720 | - | 4 | 0 | AGO1,AGO1-4 | 7mer-m8 | ucgcccugaaacUCCCGGUCAa | gagcaguagagaGGGGCCAGUu |
| NM_005529 | HSPG2 | circRNA | chr1 | 22162051 | 22162072 | - | 2 | 0 | AGO1-4,AGO2 | 7mer-m8 | ucgcccugaaacucCCGGUCAa | ugggggaaggcccaGGCCAGUg |
| NM_005529 | HSPG2 | circRNA | chr1 | 22165926 | 22165944 | - | 4 | 0 | AGO1-4,AGO2 | 8mer | ucGCCCUGAAACUCCCGGUCAa | agCAGGACU---CGGGCCAGUa |
| NM_005529 | HSPG2 | circRNA | chr1 | 22186681 | 22186702 | - | 1 | 0 | AGO1-4 | 7mer-m8 | ucgcccugaaaCUCCCGGUCAa | ccagugugcaaGGGGGCCAGUg |
| NM_004442 | EPHB2 | circRNA | chr1 | 23233323 | 23233344 | + | 1 | 0 | AGO1 | 7mer-m8 | ucgcccugaaacuCCCGGUCAa | aagccuccaucauGGGCCAGUu |
| NM_033631 | LUZP1 | circRNA | chr1 | 23415303 | 23415322 | - | 4 | 0 | AGO1-4,AGO2 | 7mer-m8 | ucgcCCUGAAACUCCCGGUCAa | cgaaGGA--UCCAAGGCCAGUu |
| NM_017707 | ASAP3 | circRNA | chr1 | 23759723 | 23759744 | - | 1 | 0 | AGO1-4 | 7mer-m8 | ucgccCUGAAACUCCCGGUCAa | gcccaGGCUCACUGGGCCAGUg |
| NM_020451 | SEPN1 | circRNA | chr1 | 26143172 | 26143193 | + | 5 | 0 | AGO1,AGO1-4,AGO2 | 7mer-m8 | ucgcccugaaacUCCCGGUCAa | gagccacugaccACGGCCAGUc |
| NM_020451 | SEPN1 | circRNA | chr1 | 26144494 | 26144515 | + | 10 | 0 | AGO1-4,AGO2 | 7mer-m8 | ucGCCCUGAAACUCCCGGUCAa | ugCUGCUCUGGGAGGGCCAGUu |
| NM_022778 | CEP85 | circRNA | chr1 | 26603138 | 26603161 | + | 2 | 0 | AGO1-4,AGO2 | 7mer-m8 | ucGCCCUGAAACUC--CCGGUCAa | agCUGCACCAGGAGUUGGCCAGUu |
| NM_022778 | CEP85 | circRNA | chr1 | 26604921 | 26604943 | + | 2 | 0 | AGO2 | 7mer-m8 | ucgCCCUG-AAACUCCCGGUCAa | cagGGGCCACGUGUGGGCCAGUc |
| NM_002953 | RPS6KA1 | circRNA | chr1 | 26901397 | 26901418 | + | 1 | 0 | AGO2 | 7mer-m8 | ucgcccugaaaCUCCCGGUCAa | cagaguucacaGGAGGCCAGUg |
| NM_006015 | ARID1A | circRNA | chr1 | 27087889 | 27087911 | + | 2 | 0 | AGO1-4 | 7mer-m8 | ucgcccugaaacUC-CCGGUCAa | ccucggccacccAGUGGCCAGUc |
| NM_001990 | EYA3 | circRNA | chr1 | 28299821 | 28299842 | - | 1 | 0 | AGO2 | 7mer-m8 | ucgcccugaaacucCCGGUCAa | cuccaggcagcucuGGCCAGUc |
| NM_031459 | SESN2 | circRNA | chr1 | 28608725 | 28608746 | + | 4 | 1 | AGO1-4,AGO2 | 7mer-m8 | ucgcccugaaacucCCGGUCAa | caggagaggagccuGGCCAGUg |
| NM_001166006 | EPB41 | circRNA | chr1 | 29415162 | 29415183 | + | 2 | 0 | AGO1-4,AGO2 | 7mer-m8 | ucgcccugaaacucCCGGUCAa | uuauauuuucuucuGGCCAGUc |
| NM_001166006 | EPB41 | circRNA | chr1 | 29422925 | 29422946 | + | 1 | 0 | AGO1-4 | 7mer-m8 | ucgcccugaaacucCCGGUCAa | ggaguggaggaacaGGCCAGUg |
| NM_001703 | BAI2 | circRNA | chr1 | 32203046 | 32203067 | - | 1 | 0 | AGO2 | 7mer-m8 | ucGCCCUGAAACUCCCGGUCAa | ucCUGAUCCUCGUGGGCCAGUc |
| NM_006559 | KHDRBS1 | circRNA | chr1 | 32508165 | 32508186 | + | 11 | 0 | AGO1,AGO1-4,AGO2 | 7mer-m8 | ucgcccugaaacucCCGGUCAa | ggccccuccugcuaGGCCAGUg |
| NM_175852 | TXLNA | circRNA | chr1 | 32663043 | 32663063 | + | 4 | 0 | AGO1-4,AGO2 | 7mer-m8 | ucgccCUGAAACUCCCGGUCAa | gucuaGACCUUCA-GGCCAGUc |
| NM_175852 | TXLNA | circRNA | chr1 | 32663185 | 32663206 | + | 7 | 0 | AGO1-4,AGO2 | 7mer-m8 | ucgcccugaaACUCCCGGUCAa | uauccaugagUGAAGGCCAGUg |
| NM_175852 | TXLNA | circRNA | chr1 | 32663551 | 32663571 | + | 5 | 0 | AGO1-4,AGO2 | 7mer-m8 | ucgccCUGAAACUCCCGGUCAa | gggcuGACCCGGA-GGCCAGUg |
| NM_023009 | MARCKSL1 | circRNA | chr1 | 32799996 | 32800017 | - | 20 | 0 | AGO1,AGO1-4,AGO2 | 7mer-m8 | ucgcccuGAAACUCCCGGUCAa | gguucucCUCUUAAGGCCAGUu |
| hsa_circ_0011527 | hsa_circ_0011527 | circRNA | chr1 | 35645452 | 35645473 | - | 1 | 0 | AGO1-4 | 7mer-m8 | ucgcccugaAACUCCCGGUCAa | uccauuaaaUCGGUGGCCAGUg |
| NM_024874 | KIAA0319L | circRNA | chr1 | 35900444 | 35900466 | - | 2 | 0 | AGO1-4,AGO2 | 7mer-m8 | ucGCCCUGAAAC-UCCCGGUCAa | ccCAGCACACUGCCCGGCCAGUc |
| NM_022111 | CLSPN | circRNA | chr1 | 36212526 | 36212547 | - | 4 | 0 | AGO1-4,AGO2 | 8mer | ucgcccugaaacucCCGGUCAa | ccucgauugccauuGGCCAGUa |
| NM_018067 | MAP7D1 | circRNA | chr1 | 36643676 | 36643697 | + | 1 | 0 | AGO1 | 8mer | ucgcccugaaacUCCCGGUCAa | cagagcaagcgcAGGGCCAGUa |
| NM_032017 | STK40 | circRNA | chr1 | 36809790 | 36809810 | - | 3 | 0 | AGO1,AGO1-4 | 7mer-m8 | ucgcccUGAAACUCCCGGUCAa | caccauGCUGU-AUGGCCAGUu |
| NM_018101 | CDCA8 | circRNA | chr1 | 38172652 | 38172671 | + | 1 | 0 | AGO1-4 | 7mer-m8 | ucGCCCUGAAACUCCCGGUCAa | ccUGCGAUUAU--UGGCCAGUg |
| NM_198446 | C1orf122 | circRNA | chr1 | 38274813 | 38274834 | + | 2 | 0 | AGO1-4 | 7mer-m8 | ucgcccugaaacUCCCGGUCAa | cccuucaguaaaGGGGCCAGUc |
| NM_012090 | MACF1 | circRNA | chr1 | 39824392 | 39824412 | + | 3 | 0 | AGO1-4,AGO2 | 8mer | ucgcccUGAAACUCCCGGUCAa | gaauauGCUGUUA-GGCCAGUa |
| NM_012090 | MACF1 | circRNA | chr1 | 39853868 | 39853885 | + | 2 | 0 | AGO1-4,AGO2 | 7mer-m8 | ucgCCCUGAAACUCCCGGUCAa | ggaGGGGAUU----GGCCAGUu |
| NM_012421 | RLF | circRNA | chr1 | 40706008 | 40706029 | + | 5 | 0 | AGO1,AGO1-4,AGO2 | 7mer-m8 | ucgcccugaaacucCCGGUCAa | gcuucauuaucuucGGCCAGUg |
| NM_001255 | CDC20 | circRNA | chr1 | 43826515 | 43826534 | + | 7 | 0 | AGO1,AGO1-4,AGO2,AGO3 | 7mer-m8 | ucgccCUGAAACUCCCGGUCAa | uggacGACAUU--UGGCCAGUg |
| NM_001255 | CDC20 | circRNA | chr1 | 43828739 | 43828762 | + | 2 | 1 | AGO1-4 | 7mer-m8 | ucGCCCUGAAACUC--CCGGUCAa | ggCGGGAGCGGGAGAAGGCCAGUg |
| NM_002840 | PTPRF | circRNA | chr1 | 44088814 | 44088840 | + | 6 | 0 | AGO1-4,AGO2 | 7mer-m8 | ucGCCCUGAAACUC-----CCGGUCAa | ggCUGGCCUUUCAGGUCCAGGCCAGUg |
| NM_014652 | IPO13 | circRNA | chr1 | 44422523 | 44422544 | + | 1 | 0 | AGO2 | 7mer-m8 | ucgcccugaaacucCCGGUCAa | ccagcagguguaccGGCCAGUc |
| NM_201649 | SLC6A9 | circRNA | chr1 | 44475687 | 44475708 | - | 1 | 0 | AGO2 | 7mer-m8 | ucgcccugaaacucCCGGUCAa | uggagcucuccuucGGCCAGUu |
| NM_024602 | HECTD3 | circRNA | chr1 | 45468401 | 45468422 | - | 1 | 0 | AGO2 | 7mer-m8 | ucgcccugaaACUCCCGGUCAa | uuugcacaggUUAAGGCCAGUu |
| NM_004799 | ZFYVE9 | circRNA | chr1 | 52703237 | 52703255 | + | 1 | 0 | AGO2 | 7mer-m8 | ucgcccUGAAACUCCCGGUCAa | aacccuACUUU---GGCCAGUg |
| NM_024646 | ZYG11B | circRNA | chr1 | 53288618 | 53288639 | + | 1 | 0 | AGO1-4 | 7mer-m8 | ucgcccuGAAACUCCCGGUCAa | uuuuuuuCAUAGAUGGCCAGUg |
| NM_002979 | SCP2 | circRNA | chr1 | 53443910 | 53443929 | + | 5 | 0 | AGO1-4,AGO2 | 7mer-m8 | ucGCCCUGAAACUCCCGGUCAa | agCAGCAAUUU--UGGCCAGUg |
| NM_004631 | LRP8 | circRNA | chr1 | 53730046 | 53730068 | - | 1 | 0 | AGO2 | 7mer-m8 | ucgcccUGAAAC--UCCCGGUCAa | #NAME? |
| NM_018087 | TMEM48 | circRNA | chr1 | 54293708 | 54293729 | - | 4 | 0 | AGO1,AGO1-4,AGO2 | 8mer | ucgcccugaaacUCCCGGUCAa | cagugauaacccAGGGCCAGUa |
| NM_015306 | USP24 | circRNA | chr1 | 55548959 | 55548980 | - | 2 | 0 | AGO1,AGO2 | 7mer-m8 | ucgcccugaaACUCCCGGUCAa | agcuuccuccUAGGGGCCAGUc |
| NM_002227 | JAK1 | circRNA | chr1 | 65307249 | 65307270 | - | 3 | 0 | AGO1-4 | 7mer-m8 | ucgccCUGAAACUCCCGGUCAa | ugaaaGCCGGUGCAGGCCAGUg |
| NM_002227 | JAK1 | circRNA | chr1 | 65311210 | 65311231 | - | 3 | 0 | AGO1-4 | 7mer-m8 | ucgcccugaaacucCCGGUCAa | guugccaaacagcuGGCCAGUg |
| NM_015139 | SLC35D1 | circRNA | chr1 | 67468533 | 67468556 | - | 2 | 0 | AGO2 | 7mer-m8 | ucgCCCUGAAAC--UCCCGGUCAa | uuaGUGUGUUUGCUCAGGCCAGUu |
| NM_012302 | LPHN2 | circRNA | chr1 | 82417685 | 82417706 | + | 2 | 0 | AGO1-4,AGO2 | 7mer-m8 | ucgcccugaaacUCCCGGUCAa | uaaacauaccaaAGGGCCAGUg |
| NM_001162536 | RBMXL1 | circRNA | chr1 | 89445254 | 89445274 | - | 4 | 0 | AGO1,AGO2 | 7mer-m8 | ucgCCCUGAAACUCCCGGUCAa | uaaGGUACUUU-UUGGCCAGUu |
| NR_036634 | TGFBR3 | circRNA | chr1 | 92146036 | 92146057 | - | 19 | 0 | AGO1,AGO1-4,AGO2 | 7mer-m8 | ucgcccugaaacucCCGGUCAa | cuuaaaaucccuguGGCCAGUu |
| NM_005665 | EVI5 | circRNA | chr1 | 93202094 | 93202115 | - | 1 | 0 | AGO1-4 | 7mer-m8 | ucgcccUGAAACUCCCGGUCAa | acagucAGUCAGAUGGCCAGUc |
| NM_001938 | DR1 | circRNA | chr1 | 93811801 | 93811824 | + | 3 | 0 | AGO1-4 | 7mer-m8 | ucGCCCUGAAACU-C-CCGGUCAa | ccCGGUACCGCGGCGAGGCCAGUg |
| NM_001938 | DR1 | circRNA | chr1 | 93819539 | 93819560 | + | 17 | 1 | AGO1-4,AGO2 | 7mer-m8 | ucgcccugaaacUCCCGGUCAa | uuaaaaagaagaAAGGCCAGUu |
| NM_001408 | CELSR2 | circRNA | chr1 | 109804466 | 109804487 | + | 1 | 0 | AGO1-4 | 7mer-m8 | ucgCCCUGAAACUCCCGGUCAa | gagGAGUCAGUGAUGGCCAGUg |
| NM_001408 | CELSR2 | circRNA | chr1 | 109808433 | 109808457 | + | 1 | 0 | AGO1-4 | 7mer-m8 | ucGCCCUGAAACUC---CCGGUCAa | ucUGUGACCCUGAGGAUGGCCAGUg |
| NM_001408 | CELSR2 | circRNA | chr1 | 109817488 | 109817509 | + | 8 | 0 | AGO1,AGO1-4,AGO2 | 7mer-m8 | ucgcccugaaacucCCGGUCAa | ccgcgccccgggcaGGCCAGUc |
| NM_020963 | MOV10 | circRNA | chr1 | 113217563 | 113217581 | + | 2 | 0 | AGO1-4 | 7mer-m8 | ucGCCCUGAAACUCCCGGUCAa | ucCGGGA---GGCGGGCCAGUg |
| NM_020190 | OLFML3 | circRNA | chr1 | 114524468 | 114524489 | + | 3 | 0 | AGO2 | 7mer-m8 | ucgcccugaaACUCCCGGUCAa | auucuucaaaUGUGGGCCAGUu |
| NM_001242891 | CSDE1 | circRNA | chr1 | 115259746 | 115259767 | - | 13 | 0 | AGO1,AGO1-4,AGO2 | 7mer-m8 | ucgcccugaaacucCCGGUCAa | uauacagggucccuGGCCAGUg |
| NM_138959 | VANGL1 | circRNA | chr1 | 116226571 | 116226592 | + | 2 | 0 | AGO1-4 | 7mer-m8 | ucgcccugaaacucCCGGUCAa | guaacaaugccacuGGCCAGUc |
| NM_001542 | IGSF3 | circRNA | chr1 | 117146344 | 117146365 | - | 1 | 0 | AGO1 | 8mer | ucgcCCUGAAACUCCCGGUCAa | ggaaGGAGGACGAGGGCCAGUa |
| NM_006699 | MAN1A2 | circRNA | chr1 | 118067368 | 118067387 | + | 1 | 0 | AGO2 | 7mer-m8 | ucgcCCUGAAACUCCCGGUCAa | auaaGGA--UGCAGGGCCAGUu |
| NM_006623 | PHGDH | circRNA | chr1 | 120278011 | 120278035 | + | 5 | 0 | AGO1-4,AGO2 | 7mer-m8 | ucGCCCUGAAACUC---CCGGUCAa | ucCGGGCCCUGCAGUCUGGCCAGUg |
| NM_024408 | NOTCH2 | circRNA | chr1 | 120469157 | 120469181 | - | 4 | 0 | AGO1-4,AGO2 | 8mer | ucgCCCUGAA-AC--UCCCGGUCAa | ggaGGGACUUGUGCUGUGGCCAGUa |
| NM_024408 | NOTCH2 | circRNA | chr1 | 120539721 | 120539742 | - | 4 | 1 | AGO1-4 | 8mer | ucGCCCUGAAACUCCCGGUCAa | cuCAGGGCUUCACAGGCCAGUa |
| TCONS_l2_00000554 | TCONS_l2_00000554 | circRNA | chr1 | 142684217 | 142684238 | + | 1 | 0 | AGO1-4 | 7mer-m8 | ucgcccugaaacucCCGGUCAa | uccaaaacccagcaGGCCAGUc |
| TCONS_00001137 | TCONS_00001137 | circRNA | chr1 | 143180685 | 143180706 | + | 1 | 0 | AGO1-4 | 7mer-m8 | ucgcccugaaacucCCGGUCAa | uccaaaacccagcaGGCCAGUc |
| TCONS_l2_00000554 | TCONS_l2_00000554 | circRNA | chr1 | 143180685 | 143180706 | + | 1 | 0 | AGO1-4 | 7mer-m8 | ucgcccugaaacucCCGGUCAa | uccaaaacccagcaGGCCAGUc |
| NM_001037675 | NBPF9 | circRNA | chr1 | 144604216 | 144604236 | + | 1 | 0 | AGO1-4 | 7mer-m8 | ucgCCCUGAAACUCCCGGUCAa | aagGGAACUCAGA-GGCCAGUg |
| NM_001039703 | NBPF10 | circRNA | chr1 | 144604216 | 144604236 | + | 1 | 0 | AGO1-4 | 7mer-m8 | ucgCCCUGAAACUCCCGGUCAa | aagGGAACUCAGA-GGCCAGUg |
| hsa_circ_0003960 | hsa_circ_0003960 | circRNA | chr1 | 144604216 | 144604236 | + | 1 | 0 | AGO1-4 | 7mer-m8 | ucgCCCUGAAACUCCCGGUCAa | aagGGAACUCAGA-GGCCAGUg |
| hsa_circ_0009085 | hsa_circ_0009085 | circRNA | chr1 | 144604216 | 144604236 | + | 1 | 0 | AGO1-4 | 7mer-m8 | ucgCCCUGAAACUCCCGGUCAa | aagGGAACUCAGA-GGCCAGUg |
| hsa_circ_0013827 | hsa_circ_0013827 | circRNA | chr1 | 144604216 | 144604236 | + | 1 | 0 | AGO1-4 | 7mer-m8 | ucgCCCUGAAACUCCCGGUCAa | aagGGAACUCAGA-GGCCAGUg |
| NR_037182 | LOC653513 | circRNA | chr1 | 144906318 | 144906339 | - | 1 | 0 | AGO1-4 | 7mer-m8 | ucgcccugaaacucCCGGUCAa | acaucgaaucccuuGGCCAGUg |
| NM_001037675 | NBPF9 | circRNA | chr1 | 145034193 | 145034215 | + | 1 | 0 | AGO1-4 | 7mer-m8 | ucgcccugaaacUC-CCGGUCAa | gaugauagaagcAGAGGCCAGUg |
| NM_001039703 | NBPF10 | circRNA | chr1 | 145034193 | 145034215 | + | 1 | 0 | AGO1-4 | 7mer-m8 | ucgcccugaaacUC-CCGGUCAa | gaugauagaagcAGAGGCCAGUg |
| NM_001037675 | NBPF9 | circRNA | chr1 | 145055145 | 145055166 | + | 1 | 0 | AGO2 | 7mer-m8 | ucgcccugaaacucCCGGUCAa | gcucccuagggcuaGGCCAGUg |
| NM_001039703 | NBPF10 | circRNA | chr1 | 145055145 | 145055166 | + | 1 | 0 | AGO2 | 7mer-m8 | ucgcccugaaacucCCGGUCAa | gcucccuagggcuaGGCCAGUg |
| NM_001037675 | NBPF9 | circRNA | chr1 | 145059525 | 145059546 | + | 3 | 0 | AGO1-4 | 7mer-m8 | ucgcccugaaacUCCCGGUCAa | acugaaguccccAGGGCCAGUc |
| NM_001039703 | NBPF10 | circRNA | chr1 | 145059525 | 145059546 | + | 3 | 0 | AGO1-4 | 7mer-m8 | ucgcccugaaacUCCCGGUCAa | acugaaguccccAGGGCCAGUc |
| NM_001037675 | NBPF9 | circRNA | chr1 | 145096502 | 145096523 | + | 12 | 0 | AGO1-4,AGO2 | 7mer-m8 | ucgcCCUGAAACUCCCGGUCAa | ccaaGGGCCUUCCGGGCCAGUg |
| NM_001039703 | NBPF10 | circRNA | chr1 | 145096502 | 145096523 | + | 12 | 0 | AGO1-4,AGO2 | 7mer-m8 | ucgcCCUGAAACUCCCGGUCAa | ccaaGGGCCUUCCGGGCCAGUg |
| NM_001037675 | NBPF9 | circRNA | chr1 | 145101527 | 145101546 | + | 1 | 0 | AGO1-4 | 7mer-m8 | ucgcccUGAAACUCCCGGUCAa | cagaauGCUUU--UGGCCAGUu |
| NM_001039703 | NBPF10 | circRNA | chr1 | 145101527 | 145101546 | + | 1 | 0 | AGO1-4 | 7mer-m8 | ucgcccUGAAACUCCCGGUCAa | cagaauGCUUU--UGGCCAGUu |
| NM_001037675 | NBPF9 | circRNA | chr1 | 145254672 | 145254693 | + | 1 | 0 | AGO1-4 | 7mer-m8 | ucgcccugaaacucCCGGUCAa | gauagcagaagcuuGGCCAGUu |
| NM_001039703 | NBPF10 | circRNA | chr1 | 145254672 | 145254693 | + | 1 | 0 | AGO1-4 | 7mer-m8 | ucgcccugaaacucCCGGUCAa | gauagcagaagcuuGGCCAGUu |
| NM_001037675 | NBPF9 | circRNA | chr1 | 145259994 | 145260016 | + | 1 | 0 | AGO1-4 | 7mer-m8 | ucgCCCUGA-AACUCCCGGUCAa | acaGGCUCUCAUGUUGGCCAGUu |
| NM_001039703 | NBPF10 | circRNA | chr1 | 145259994 | 145260016 | + | 1 | 0 | AGO1-4 | 7mer-m8 | ucgCCCUGA-AACUCCCGGUCAa | acaGGCUCUCAUGUUGGCCAGUu |
| NM_001037675 | NBPF9 | circRNA | chr1 | 145281582 | 145281603 | + | 5 | 1 | AGO1-4 | 8mer | ucGCCCUGAAACUCCCGGUCAa | uuCAGGGCUUCACAGGCCAGUa |
| NM_001039703 | NBPF10 | circRNA | chr1 | 145281582 | 145281603 | + | 5 | 1 | AGO1-4 | 8mer | ucGCCCUGAAACUCCCGGUCAa | uuCAGGGCUUCACAGGCCAGUa |
| NM_203458 | NOTCH2NL | circRNA | chr1 | 145281582 | 145281603 | + | 5 | 1 | AGO1-4 | 8mer | ucGCCCUGAAACUCCCGGUCAa | uuCAGGGCUUCACAGGCCAGUa |
| NM_001039703 | NBPF10 | circRNA | chr1 | 145414780 | 145414801 | + | 1 | 0 | AGO1-4 | 7mer-m8 | ucgcccugaaacucCCGGUCAa | guaugggggagccaGGCCAGUc |
| NM_001039703 | NBPF10 | circRNA | chr1 | 145701567 | 145701586 | + | 1 | 0 | AGO1-4 | 7mer-m8 | ucgccCUGAAACUCCCGGUCAa | ucaaaGGCUCU--GGGCCAGUu |
| NM_001097616 | GPR89C | circRNA | chr1 | 147096349 | 147096370 | + | 1 | 0 | AGO2 | 7mer-m8 | ucGCCCUGAAACUCCCGGUCAa | caUGGGAGGUCCAGGGCCAGUg |
| NM_004326 | BCL9 | circRNA | chr1 | 147096349 | 147096370 | + | 1 | 0 | AGO2 | 7mer-m8 | ucGCCCUGAAACUCCCGGUCAa | caUGGGAGGUCCAGGGCCAGUg |
| NM_020205 | OTUD7B | circRNA | chr1 | 149982597 | 149982619 | - | 1 | 0 | AGO1-4 | 7mer-m8 | ucgcccUGAAACUC-CCGGUCAa | aaagccACUGGGGGAGGCCAGUc |
| NM_019032 | ADAMTSL4 | circRNA | chr1 | 150526320 | 150526341 | + | 1 | 0 | AGO1-4 | 7mer-m8 | ucgcccugaaacuCCCGGUCAa | aguucccaggguuGGGCCAGUc |
| NM_021960 | MCL1 | circRNA | chr1 | 150549517 | 150549539 | - | 35 | 0 | AGO1,AGO1-4,AGO2 | 8mer | ucgcccugaaacUC-CCGGUCAa | ugagaacaggaaAGUGGCCAGUa |
| NM_005997 | VPS72 | circRNA | chr1 | 151156891 | 151156912 | - | 2 | 0 | AGO1-4 | 7mer-m8 | ucGCCCUGAAACUCCCGGUCAa | uaCAGGAGAGGCAGGGCCAGUc |
| NM_001194937 | POGZ | circRNA | chr1 | 151400366 | 151400387 | - | 2 | 0 | AGO1-4 | 7mer-m8 | ucgcccugaaacuCCCGGUCAa | gggccagaguccuGGGCCAGUg |
| NM_015100 | POGZ | circRNA | chr1 | 151400366 | 151400387 | - | 2 | 0 | AGO1-4 | 7mer-m8 | ucgcccugaaacuCCCGGUCAa | gggccagaguccuGGGCCAGUg |
| NM_015100 | POGZ | circRNA | chr1 | 151400625 | 151400646 | - | 9 | 0 | AGO1-4,AGO2 | 7mer-m8 | ucgcccugaaacucCCGGUCAa | cuguucagucuccaGGCCAGUc |
| NM_014624 | S100A6 | circRNA | chr1 | 153508485 | 153508505 | - | 3 | 0 | AGO1-4,AGO2 | 7mer-m8 | ucgcccUGAAACUCCCGGUCAa | ccgaccGCUAU-AAGGCCAGUc |
| NM_001193495 | ADAR | circRNA | chr1 | 154562713 | 154562734 | - | 2 | 0 | AGO1-4 | 7mer-m8 | ucgcccugaaacUCCCGGUCAa | accccagugacaGGGGCCAGUc |
| NM_001193495 | ADAR | circRNA | chr1 | 154573757 | 154573781 | - | 13 | 0 | AGO1-4,AGO2 | 7mer-m8 | ucgccCUGAAAC---UCCCGGUCAa | auguuGACUUUGAAAAUGGCCAGUg |
| NM_001130040 | SHC1 | circRNA | chr1 | 154938083 | 154938104 | - | 8 | 0 | AGO1-4 | 8mer | ucgcccugaaacucCCGGUCAa | gcacgaccacaccuGGCCAGUa |
| NM_183001 | SHC1 | circRNA | chr1 | 154938083 | 154938104 | - | 8 | 0 | AGO1-4 | 8mer | ucgcccugaaacucCCGGUCAa | gcacgaccacaccuGGCCAGUa |
| NM_001105203 | RUSC1 | circRNA | chr1 | 155300403 | 155300424 | + | 8 | 0 | AGO1-4,AGO2 | 7mer-m8 | ucGCCCUGAAACUCCCGGUCAa | ccUGGGAAUGGAAUGGCCAGUg |
| NM_001198903 | YY1AP1 | circRNA | chr1 | 155644818 | 155644839 | - | 1 | 0 | AGO2 | 8mer | ucgcccUGAAACUCCCGGUCAa | aaucucAAUCCGGAGGCCAGUa |
| NM_139121 | YY1AP1 | circRNA | chr1 | 155644818 | 155644839 | - | 1 | 0 | AGO2 | 8mer | ucgcccUGAAACUCCCGGUCAa | aaucucAAUCCGGAGGCCAGUa |
| NM_001037533 | GON4L | circRNA | chr1 | 155736494 | 155736515 | - | 1 | 0 | AGO1-4 | 7mer-m8 | ucgcccugaaacucCCGGUCAa | ucuuguuuccaauaGGCCAGUc |
| NM_001198903 | YY1AP1 | circRNA | chr1 | 155736494 | 155736515 | - | 1 | 0 | AGO1-4 | 7mer-m8 | ucgcccugaaacucCCGGUCAa | ucuuguuuccaauaGGCCAGUc |
| hsa_circ_0000139 | hsa_circ_000022 | circRNA | chr1 | 155823149 | 155823170 | - | 1 | 0 | AGO2 | 7mer-m8 | ucgcccugaaacuCCCGGUCAa | gacacucaggccuGGGCCAGUu |
| NM_014655 | SLC25A44 | circRNA | chr1 | 156169883 | 156169904 | + | 6 | 0 | AGO1-4,AGO2 | 7mer-m8 | ucgcccugaaacucCCGGUCAa | ucacccucaucucuGGCCAGUg |
| NR_026678 | TMEM79 | circRNA | chr1 | 156261951 | 156261974 | + | 1 | 0 | AGO2 | 7mer-m8 | ucgcccUGAAACUC--CCGGUCAa | uuuacuGUUUACAGCUGGCCAGUg |
| NM_006617 | NES | circRNA | chr1 | 156642604 | 156642625 | - | 1 | 0 | AGO1-4 | 7mer-m8 | ucgcCCUGAAACUCCCGGUCAa | aagaGGCCAGUACAGGCCAGUc |
| NM_198236 | ARHGEF11 | circRNA | chr1 | 156908254 | 156908276 | - | 1 | 0 | AGO1-4 | 7mer-m8 | ucgcccugaaaCUC-CCGGUCAa | gcacuaccagaGAGUGGCCAGUc |
| NR_028103 | DCAF8 | circRNA | chr1 | 160194325 | 160194339 | - | 3 | 0 | AGO1-4 | 7mer-m8 | ucgcccugaaacucCCGGUCAa | #NAME? |
| NR_028103 | DCAF8 | circRNA | chr1 | 160209579 | 160209600 | - | 5 | 0 | AGO1-4,AGO2 | 7mer-m8 | ucGCCCUGAAACUCCCGGUCAa | cgCGGCACCUGGCUGGCCAGUg |
| NM_001098398 | COPA | circRNA | chr1 | 160268741 | 160268761 | - | 8 | 0 | AGO1-4,AGO2 | 7mer-m8 | ucgcccUGAAACUCCCGGUCAa | ugccaaAC-UAGUUGGCCAGUc |
| NM_001643 | APOA2 | circRNA | chr1 | 161192136 | 161192157 | - | 1 | 0 | AGO1-4 | 7mer-m8 | ucgcccugaaacucCCGGUCAa | cuagaacacccacuGGCCAGUc |
| NM_007348 | ATF6 | circRNA | chr1 | 161933453 | 161933474 | + | 2 | 0 | AGO1-4,AGO2 | 7mer-m8 | ucgcccUGAAACUCCCGGUCAa | ugccauAAUUUCCAGGCCAGUc |
| NM_001014796 | DDR2 | circRNA | chr1 | 162731157 | 162731178 | + | 1 | 0 | AGO1-4 | 7mer-m8 | ucgcccugaaacUCCCGGUCAa | cuccaccaccgaAUGGCCAGUg |
| NM_000696 | ALDH9A1 | circRNA | chr1 | 165649788 | 165649810 | - | 4 | 0 | AGO1-4 | 7mer-m8 | ucgCCCUGAAACU-CCCGGUCAa | ggaGGGGCUGCCACAGGCCAGUu |
| NM_012474 | UCK2 | circRNA | chr1 | 165859562 | 165859583 | + | 6 | 1 | AGO1-4,AGO2 | 7mer-m8 | ucgccCUGAAACUCCCGGUCAa | ccaaaGCCCUGAAGGGCCAGUu |
| hsa_circ_0000147 | hsa_circ_001357 | circRNA | chr1 | 165859562 | 165859583 | + | 6 | 1 | AGO1-4,AGO2 | 7mer-m8 | ucgccCUGAAACUCCCGGUCAa | ccaaaGCCCUGAAGGGCCAGUu |
| NM_199344 | SFT2D2 | circRNA | chr1 | 168211770 | 168211791 | + | 32 | 1 | AGO1,AGO1-4,AGO2,AGO3 | 7mer-m8 | ucgcccugaAACUCCCGGUCAa | cuugcauaaUUCAUGGCCAGUu |
| NM_015172 | PRRC2C | circRNA | chr1 | 171483739 | 171483760 | + | 14 | 1 | AGO1,AGO1-4,AGO2 | 8mer | ucgcccugaaacuCCCGGUCAa | gcucccaaaucauGGGCCAGUa |
| NM_001007239 | METTL13 | circRNA | chr1 | 171755066 | 171755087 | + | 2 | 0 | AGO1,AGO1-4 | 7mer-m8 | ucgcccugaaACUCCCGGUCAa | cggaaacagcUGGCGGCCAGUg |
| NM_015935 | METTL13 | circRNA | chr1 | 171755066 | 171755087 | + | 2 | 0 | AGO1,AGO1-4 | 7mer-m8 | ucgcccugaaACUCCCGGUCAa | cggaaacagcUGGCGGCCAGUg |
| NM_002826 | QSOX1 | circRNA | chr1 | 180166000 | 180166018 | + | 4 | 0 | AGO1-4 | 7mer-m8 | ucGCCCUGAAACUCCCGGUCAa | gaCGGGGC--CGA-GGCCAGUg |
| NM_032360 | ACBD6 | circRNA | chr1 | 180399347 | 180399368 | - | 1 | 3 | AGO1 | 7mer-m8 | ucGCCCUGAAACUCCCGGUCAa | uaCAGGUUUUGGUGGGCCAGUu |
| hsa_circ_0000164 | hsa_circ_000547 | circRNA | chr1 | 180399347 | 180399368 | - | 1 | 3 | AGO1 | 7mer-m8 | ucGCCCUGAAACUCCCGGUCAa | uaCAGGUUUUGGUGGGCCAGUu |
| NM_005819 | STX6 | circRNA | chr1 | 180945522 | 180945543 | - | 4 | 0 | AGO1-4 | 7mer-m8 | ucGCCCUGAAACUCCCGGUCAa | ccCAGCGCUGGAAGGGCCAGUg |
| NM_005819 | STX6 | circRNA | chr1 | 180991994 | 180992011 | - | 1 | 0 | AGO1-4 | 7mer-m8 | ucGCCCUGAAACUCCCGGUCAa | ucCGGGAUU----CGGCCAGUg |
| NM_002293 | LAMC1 | circRNA | chr1 | 183095293 | 183095314 | + | 9 | 0 | AGO1-4 | 7mer-m8 | ucgcccugaaacucCCGGUCAa | gugacauccgcaccGGCCAGUg |
| NM_002293 | LAMC1 | circRNA | chr1 | 183112350 | 183112371 | + | 19 | 0 | AGO1,AGO1-4,AGO2,AGO4 | 8mer | ucgcCCUGAAACUCCCGGUCAa | aguaGCAUUGUGAUGGCCAGUa |
| NM_005717 | ARPC5 | circRNA | chr1 | 183596281 | 183596301 | - | 6 | 0 | AGO1-4,AGO2 | 7mer-m8 | ucgccCUGAAACUCCCGGUCAa | ggucaGAAUUUGA-GGCCAGUu |
| NM_052966 | FAM129A | circRNA | chr1 | 184764266 | 184764287 | - | 1 | 0 | AGO1-4 | 7mer-m8 | ucgcccugaaacUCCCGGUCAa | gcggcccaggccACGGCCAGUg |
| NM_007212 | RNF2 | circRNA | chr1 | 185069068 | 185069090 | + | 1 | 0 | AGO1-4 | 7mer-m8 | ucgcccugaaacUC-CCGGUCAa | auagcaacagccAGUGGCCAGUu |
| NM_006469 | IVNS1ABP | circRNA | chr1 | 185278534 | 185278556 | - | 11 | 0 | AGO1-4,AGO2 | 7mer-m8 | ucgcccugaaacUC-CCGGUCAa | gcccugaggaaaAGUGGCCAGUu |
| NM_031935 | HMCN1 | circRNA | chr1 | 185953379 | 185953400 | + | 3 | 0 | AGO1-4 | 8mer | ucgcccUGAAACUCCCGGUCAa | gaauauACUUGUGUGGCCAGUa |
| NM_031935 | HMCN1 | circRNA | chr1 | 186113676 | 186113694 | + | 2 | 0 | AGO2 | 7mer-m8 | ucgCCCUGAAACUCCCGGUCAa | uggGCGACUU---GGGCCAGUu |
| NM_003292 | TPR | circRNA | chr1 | 186330980 | 186331001 | - | 2 | 0 | AGO1-4 | 8mer | ucgcccugaaacucCCGGUCAa | gccaaggaacaacaGGCCAGUa |
| NM_014875 | KIF14 | circRNA | chr1 | 200522833 | 200522850 | - | 1 | 0 | AGO1-4 | 7mer-m8 | ucGCCCUGAAACUCCCGGUCAa | uuCGCAACUU----GGCCAGUg |
| NM_001031725 | DDX59 | circRNA | chr1 | 200635663 | 200635684 | - | 1 | 0 | AGO2 | 7mer-m8 | ucgcccugaaaCUCCCGGUCAa | uccccagcccaGGUGGCCAGUu |
| NM_001142569 | C1orf106 | circRNA | chr1 | 200878435 | 200878456 | + | 1 | 0 | AGO1-4 | 7mer-m8 | ucGCCCUGAAACUCCCGGUCAa | ucCGUGGUGUUCCUGGCCAGUg |
| NM_020443 | NAV1 | circRNA | chr1 | 201687601 | 201687619 | + | 1 | 0 | AGO1-4 | 7mer-m8 | ucGCCCUGAAACUCCCGGUCAa | caUGGCGCU---AUGGCCAGUc |
| NM_020443 | NAV1 | circRNA | chr1 | 201766506 | 201766527 | + | 1 | 0 | AGO2 | 8mer | ucgcccugaaacucCCGGUCAa | auaugucuauccuuGGCCAGUa |
| NM_020443 | NAV1 | circRNA | chr1 | 201790313 | 201790333 | + | 1 | 0 | AGO2 | 7mer-m8 | ucgcccugaAACUCCCGGUCAa | uccucugucUUG-UGGCCAGUu |
| NM_018085 | IPO9 | circRNA | chr1 | 201847926 | 201847949 | + | 2 | 0 | AGO1-4,AGO2 | 8mer | ucgccCU-GA-AACUCCCGGUCAa | aaaaaGAUCUGUUGGAGGCCAGUa |
| NM_014176 | UBE2T | circRNA | chr1 | 202301004 | 202301025 | - | 5 | 0 | AGO1-4,AGO2 | 7mer-m8 | ucgcccugaaaCUCCCGGUCAa | acacagaaaagGAAGGCCAGUc |
| NM_021633 | KLHL12 | circRNA | chr1 | 202863737 | 202863759 | - | 1 | 0 | AGO1-4 | 7mer-m8 | ucGCCCUGAAACUC-CCGGUCAa | ugCCGGACUCGUAGUGGCCAGUg |
| NM_001001396 | ATP2B4 | circRNA | chr1 | 203710588 | 203710614 | + | 6 | 0 | AGO1-4,AGO2 | 7mer-m8 | ucgccCUGAA---AC--UCCCGGUCAa | guguuGACUUCUGUGUAGGGGCCAGUu |
| NM_014827 | ZC3H11A | circRNA | chr1 | 203822723 | 203822745 | + | 4 | 0 | AGO1-4 | 7mer-m8 | ucgcccUGA-AACUCCCGGUCAa | uauuuuGCUGUAGGUGGCCAGUu |
| NM_006338 | LRRN2 | circRNA | chr1 | 204587759 | 204587781 | + | 1 | 0 | AGO2 | 7mer-m8 | ucGCCCUGAAACUC---CCGGUCAa | cuCGGG--UUCGGGUUCGGCCAGUg |
| NM_004759 | MAPKAPK2 | circRNA | chr1 | 206904545 | 206904566 | + | 4 | 0 | AGO1-4,AGO2 | 8mer | ucgcccugaaacuCCCGGUCAa | cucgcauccgaauGGGCCAGUa |
| NM_000228 | LAMB3 | circRNA | chr1 | 209800790 | 209800813 | - | 1 | 0 | AGO1-4 | 7mer-m8 | ucgcccUGAAACUC--CCGGUCAa | ccuaccACUGGAAGCUGGCCAGUg |
| NM_001017402 | LAMB3 | circRNA | chr1 | 209800790 | 209800813 | - | 1 | 0 | AGO1-4 | 7mer-m8 | ucgcccUGAAACUC--CCGGUCAa | ccuaccACUGGAAGCUGGCCAGUg |
| NM_018194 | HHAT | circRNA | chr1 | 210848489 | 210848510 | + | 1 | 0 | AGO1-4 | 7mer-m8 | ucgCCCUGAAACUCCCGGUCAa | augGGUGUUAUCAAGGCCAGUc |
| NM_002763 | PROX1 | circRNA | chr1 | 214171148 | 214171172 | + | 1 | 0 | AGO1-4 | 7mer-m8 | ucGCCCUGAAAC-UC--CCGGUCAa | uuUGGCAAUGUGCAGAUGGCCAGUu |
| NM_004446 | EPRS | circRNA | chr1 | 220203729 | 220203751 | - | 3 | 0 | AGO1-4,AGO2 | 7mer-m8 | ucgcccuGAAACUC-CCGGUCAa | cagauuuCCUCCAGAGGCCAGUg |
| TCONS_l2_00001808 | TCONS_l2_00001808 | circRNA | chr1 | 223805786 | 223805803 | - | 1 | 0 | AGO1-4 | 7mer-m8 | ucGCCCUGAAACUCCCGGUCAa | caUGGGGC----AGGGCCAGUc |
| NM_014698 | TMEM63A | circRNA | chr1 | 226033383 | 226033404 | - | 1 | 0 | AGO1-4 | 7mer-m8 | ucgcccugaaacucCCGGUCAa | ugcagcauagcccuGGCCAGUg |
| NM_014698 | TMEM63A | circRNA | chr1 | 226033465 | 226033486 | - | 2 | 0 | AGO1-4 | 7mer-m8 | ucgcccugaaACUCCCGGUCAa | gcccccgcagUGGUGGCCAGUg |
| NM_014698 | TMEM63A | circRNA | chr1 | 226034145 | 226034168 | - | 1 | 0 | AGO2 | 7mer-m8 | ucgccCUGAAACUC--CCGGUCAa | gaacaGGUUGGGAGGUGGCCAGUg |
| NM_014698 | TMEM63A | circRNA | chr1 | 226034220 | 226034241 | - | 3 | 0 | AGO2 | 7mer-m8 | ucgcccugaaacUCCCGGUCAa | gcucccugcuccAUGGCCAGUc |
| NM_014698 | TMEM63A | circRNA | chr1 | 226050186 | 226050207 | - | 1 | 0 | AGO1-4 | 7mer-m8 | ucgcccugaaacucCCGGUCAa | accccaagcccuguGGCCAGUu |
| NM_002221 | ITPKB | circRNA | chr1 | 226821988 | 226822009 | - | 1 | 0 | AGO1-4 | 7mer-m8 | ucgcccugaaACUCCCGGUCAa | ugcugcccccUGGCGGCCAGUg |
| NM_020247 | ADCK3 | circRNA | chr1 | 227107666 | 227107687 | + | 1 | 0 | AGO2 | 8mer | ucgcccugaaacUCCCGGUCAa | uacauaauaaacACGGCCAGUa |
| NM_001024227 | ARF1 | circRNA | chr1 | 228284779 | 228284794 | + | 6 | 0 | AGO1,AGO1-4 | 7mer-m8 | ucgcccugaaacucCCGGUCAa | #NAME? |
| NM_001098623 | OBSCN | circRNA | chr1 | 228430931 | 228430952 | + | 1 | 0 | AGO1-4 | 7mer-m8 | ucgcccugaaacUCCCGGUCAa | caggcugaggcaGGGGCCAGUg |
| NM_001098623 | OBSCN | circRNA | chr1 | 228432044 | 228432065 | + | 1 | 0 | AGO1-4 | 7mer-m8 | ucgcccugaaaCUCCCGGUCAa | caggcugaggcGGGGGCCAGUg |
| NM_018230 | NUP133 | circRNA | chr1 | 229599408 | 229599423 | - | 4 | 0 | AGO1-4,AGO2 | 8mer | ucgcccugAAACU--CCCGGUCAa | #NAME? |
| NM_012089 | ABCB10 | circRNA | chr1 | 229653542 | 229653564 | - | 3 | 0 | AGO2 | 7mer-m8 | ucgccCUGA-AACUCCCGGUCAa | agccuGAUUAUUUUAGGCCAGUu |
| NM_014777 | URB2 | circRNA | chr1 | 229772634 | 229772653 | + | 2 | 0 | AGO2 | 7mer-m8 | ucGCCCUGAAACUCCCGGUCAa | ugUGGGA--UACCUGGCCAGUg |
| NM_022051 | EGLN1 | circRNA | chr1 | 231521527 | 231521548 | - | 2 | 0 | AGO1-4,AGO2 | 7mer-m8 | ucgcccugAAACUCCCGGUCAa | uuuacaugUUUUCUGGCCAGUc |
| NM_020808 | SIPA1L2 | circRNA | chr1 | 232600806 | 232600824 | - | 1 | 0 | AGO2 | 7mer-m8 | ucGCCCUGAAACUCCCGGUCAa | ccCGGGACUU---CGGCCAGUc |
| NM_014801 | PCNXL2 | circRNA | chr1 | 233225830 | 233225851 | - | 1 | 0 | AGO1-4 | 7mer-m8 | ucgcccugaaacucCCGGUCAa | cacaucauaugucaGGCCAGUg |
| NM_001079515 | TBCE | circRNA | chr1 | 235596397 | 235596420 | + | 1 | 0 | AGO1-4 | 7mer-m8 | ucgcCCUGAAAC--UCCCGGUCAa | ggauGAACGUGGCCAAGGCCAGUg |
| NM_080738 | EDARADD | circRNA | chr1 | 236647374 | 236647395 | + | 5 | 0 | AGO1,AGO1-4 | 7mer-m8 | ucgccCUGAAACUCCCGGUCAa | uggcaGAAGUUCACGGCCAGUg |
| NM_016002 | SCCPDH | circRNA | chr1 | 246887768 | 246887789 | + | 3 | 0 | AGO1-4,AGO2 | 7mer-m8 | ucgccCUGAAACUCCCGGUCAa | cgucuGGCUUCACCGGCCAGUu |
| NM_024836 | ZNF672 | circRNA | chr1 | 249143417 | 249143438 | + | 1 | 0 | AGO1-4 | 7mer-m8 | ucgccCUGAAACUCCCGGUCAa | acccuGGCAGAGGUGGCCAGUc |
| NM_012293 | PXDN | circRNA | chr2 | 1635991 | 1636012 | - | 9 | 0 | AGO1-4,AGO2 | 7mer-m8 | ucgcCCUGAAACUCCCGGUCAa | uaauGUGCGGUCUGGGCCAGUc |
| NM_012293 | PXDN | circRNA | chr2 | 1653081 | 1653102 | - | 1 | 0 | AGO1-4 | 7mer-m8 | ucgcccugaaacUCCCGGUCAa | ugcugaugcaguGGGGCCAGUu |
| NM_012293 | PXDN | circRNA | chr2 | 1667445 | 1667466 | - | 2 | 0 | AGO1-4 | 8mer | ucgcccugaaacUCCCGGUCAa | cccuccacgaccAGGGCCAGUa |
| NM_198182 | GRHL1 | circRNA | chr2 | 10104083 | 10104104 | + | 1 | 0 | AGO1-4 | 7mer-m8 | ucgcccugaaacUCCCGGUCAa | cguaccugaacaAAGGCCAGUu |
| NM_134421 | HPCAL1 | circRNA | chr2 | 10512959 | 10512980 | + | 1 | 0 | AGO1-4 | 7mer-m8 | ucgcccugaaacucCCGGUCAa | gaccccaaaacuguGGCCAGUu |
| NM_152391 | PQLC3 | circRNA | chr2 | 11312060 | 11312081 | + | 2 | 0 | AGO1-4,AGO2 | 8mer | ucgcccugaaacucCCGGUCAa | acuuucaucagcgcGGCCAGUa |
| NM_021643 | TRIB2 | circRNA | chr2 | 12880679 | 12880700 | + | 2 | 0 | AGO1-4 | 7mer-m8 | ucgcccugaaacucCCGGUCAa | gcaagauccggcguGGCCAGUu |
| NM_001006946 | SDC1 | circRNA | chr2 | 20400771 | 20400792 | - | 1 | 0 | AGO1-4 | 7mer-m8 | ucgcccugaaaCUCCCGGUCAa | cacuccgagagGUGGGCCAGUc |
| NM_002997 | SDC1 | circRNA | chr2 | 20400771 | 20400792 | - | 1 | 0 | AGO1-4 | 7mer-m8 | ucgcccugaaaCUCCCGGUCAa | cacuccgagagGUGGGCCAGUc |
| NM_002997 | SDC1 | circRNA | chr2 | 20424888 | 20424909 | - | 1 | 0 | AGO1-4 | 7mer-m8 | ucGCCCUGAAACUCCCGGUCAa | agCUGGGGGUGGGGGGCCAGUu |
| NM_147223 | NCOA1 | circRNA | chr2 | 24929702 | 24929723 | + | 2 | 0 | AGO2 | 7mer-m8 | ucgcccugaaacucCCGGUCAa | uuaaaccaaggacaGGCCAGUu |
| NM_001013663 | PTRHD1 | circRNA | chr2 | 25013308 | 25013329 | - | 6 | 0 | AGO1-4,AGO2 | 8mer | ucgcCCUGAAACUCCCGGUCAa | ccaaGGAAGAAGUGGGCCAGUa |
| NM_004036 | ADCY3 | circRNA | chr2 | 25141873 | 25141896 | - | 3 | 0 | AGO1-4,AGO2 | 8mer | ucgcccUGAA-A-CUCCCGGUCAa | caguccACUUCUCUAGGGCCAGUa |
| NM_000183 | HADHB | circRNA | chr2 | 26501488 | 26501509 | + | 5 | 0 | AGO1-4 | 7mer-m8 | ucgcccugaaacucCCGGUCAa | gcuugauugcuucuGGCCAGUg |
| NM_000183 | HADHB | circRNA | chr2 | 26508384 | 26508405 | + | 2 | 0 | AGO1-4 | 8mer | ucGCCCUGAAACUCCCGGUCAa | uaCGGAAAGAAGGAGGCCAGUa |
| NM_001253723 | DPYSL5 | circRNA | chr2 | 27170222 | 27170244 | + | 2 | 0 | AGO2 | 7mer-m8 | ucgcccugaaACU-CCCGGUCAa | gcccccuggcUGACUGGCCAGUu |
| NM_020134 | DPYSL5 | circRNA | chr2 | 27170222 | 27170244 | + | 2 | 0 | AGO2 | 7mer-m8 | ucgcccugaaACU-CCCGGUCAa | gcccccuggcUGACUGGCCAGUu |
| NM_017727 | TMEM214 | circRNA | chr2 | 27263814 | 27263836 | + | 2 | 0 | AGO1-4,AGO2 | 7mer-m8 | ucgcccugaaacUC-CCGGUCAa | cucuccuaggcaAGUGGCCAGUu |
| NM_001035507 | AGBL5 | circRNA | chr2 | 27279582 | 27279606 | + | 2 | 2 | AGO2 | 7mer-m8 | ucGCCCU-GAAACUC--CCGGUCAa | ccCGAGACCGUAGAGAUGGCCAGUc |
| NM_021831 | AGBL5 | circRNA | chr2 | 27279582 | 27279606 | + | 2 | 2 | AGO2 | 7mer-m8 | ucGCCCU-GAAACUC--CCGGUCAa | ccCGAGACCGUAGAGAUGGCCAGUc |
| NM_015662 | IFT172 | circRNA | chr2 | 27680529 | 27680550 | - | 1 | 0 | AGO1-4 | 7mer-m8 | ucgcccugaaacuCCCGGUCAa | gugaacauguaccGGGCCAGUg |
| NM_182551 | LCLAT1 | circRNA | chr2 | 30806491 | 30806509 | + | 1 | 0 | AGO1-4 | 7mer-m8 | ucgccCUGAAACUCCCGGUCAa | uguccGAAUUU---GGCCAGUg |
| NM_182551 | LCLAT1 | circRNA | chr2 | 30817485 | 30817507 | + | 2 | 0 | AGO1-4,AGO2 | 7mer-m8 | ucgcccugaaacUC-CCGGUCAa | uguuuacaccaaAGUGGCCAGUu |
| NM_000379 | XDH | circRNA | chr2 | 31604526 | 31604547 | - | 1 | 0 | AGO1-4 | 7mer-m8 | ucgcccugaAACUCCCGGUCAa | aaccccgugUUCAUGGCCAGUg |
| NM_016252 | BIRC6 | circRNA | chr2 | 32661158 | 32661179 | + | 1 | 0 | AGO1-4 | 8mer | ucgcccuGAAACUCCCGGUCAa | agacauuCUAUGUGGGCCAGUa |
| NM_206943 | LTBP1 | circRNA | chr2 | 33411938 | 33411959 | + | 5 | 0 | AGO1-4 | 7mer-m8 | ucgcccugaaACUCCCGGUCAa | cauguaugaaUGGUGGCCAGUg |
| NM_001166265 | LTBP1 | circRNA | chr2 | 33500117 | 33500136 | + | 6 | 0 | AGO1-4,AGO2 | 7mer-m8 | ucGCCCUGAAACUCCCGGUCAa | agCAGGAUUU--AUGGCCAGUg |
| NM_206943 | LTBP1 | circRNA | chr2 | 33500117 | 33500136 | + | 6 | 0 | AGO1-4,AGO2 | 7mer-m8 | ucGCCCUGAAACUCCCGGUCAa | agCAGGAUUU--AUGGCCAGUg |
| NM_206943 | LTBP1 | circRNA | chr2 | 33589307 | 33589328 | + | 5 | 0 | AGO1-4,AGO2 | 7mer-m8 | ucgcccugaAACUCCCGGUCAa | guaguuguaUUGAUGGCCAGUg |
| NM_016441 | CRIM1 | circRNA | chr2 | 36749414 | 36749439 | + | 11 | 0 | AGO1-4,AGO2 | 7mer-m8 | ucgcccugaAACUC----CCGGUCAa | agaccugucUUGAGAAAAGGCCAGUg |
| NM_005813 | PRKD3 | circRNA | chr2 | 37479382 | 37479404 | - | 2 | 0 | AGO1-4 | 7mer-m8 | ucgcccuGAAAC-UCCCGGUCAa | ucugcuuUUUAGCAGGGCCAGUg |
| NM_005813 | PRKD3 | circRNA | chr2 | 37496775 | 37496798 | - | 1 | 1 | AGO1-4 | 7mer-m8 | ucgCCCUGAA--ACUCCCGGUCAa | ugaGGUGCUUGGUUCAGGCCAGUu |
| NM_001083953 | THADA | circRNA | chr2 | 43823014 | 43823037 | - | 1 | 0 | AGO2 | 7mer-m8 | ucGCCCUGAAACU--CCCGGUCAa | cgCGGUAGCUCGGCUCGGCCAGUu |
| NM_005400 | PRKCE | circRNA | chr2 | 45990400 | 45990418 | + | 1 | 0 | AGO1-4 | 7mer-m8 | ucgcccuGAAACUCCCGGUCAa | guucuuuCUUU---GGCCAGUc |
| NM_005400 | PRKCE | circRNA | chr2 | 45998093 | 45998114 | + | 1 | 0 | AGO1-4 | 7mer-m8 | ucgccCUGAAACUCCCGGUCAa | ucauuGUUUAUGCUGGCCAGUu |
| NM_005400 | PRKCE | circRNA | chr2 | 46001916 | 46001939 | + | 1 | 0 | AGO1-4 | 7mer-m8 | ucGCCCUGAAA--CUCCCGGUCAa | agCUGCACAUUCCCAGGGCCAGUg |
| NM_001430 | EPAS1 | circRNA | chr2 | 46602840 | 46602862 | + | 3 | 0 | AGO1-4 | 8mer | ucgccCUGAAACUC-CCGGUCAa | ggucaGGUAGUAAGUGGCCAGUa |
| NM_001039348 | EFEMP1 | circRNA | chr2 | 56144953 | 56144973 | - | 7 | 0 | AGO1-4,AGO2 | 7mer-m8 | ucgCCCUGAAACUCCCGGUCAa | gggGUGGUUUUG-UGGCCAGUg |
| NM_001244710 | GFPT1 | circRNA | chr2 | 69565035 | 69565056 | - | 7 | 0 | AGO1-4,AGO2 | 8mer | ucGCCCUGAAACUC-CCGGUCAa | ccUGAGA-UUGGUGUGGCCAGUa |
| hsa_circ_0001025 | hsa_circ_000308 | circRNA | chr2 | 69565035 | 69565056 | - | 7 | 0 | AGO1-4,AGO2 | 8mer | ucGCCCUGAAACUC-CCGGUCAa | ccUGAGA-UUGGUGUGGCCAGUa |
| NM_017880 | C2orf42 | circRNA | chr2 | 70396731 | 70396751 | - | 2 | 0 | AGO1-4,AGO2 | 7mer-m8 | ucgccCUGAAACUCCCGGUCAa | uccaaGAC-UGGCUGGCCAGUg |
| NM_022173 | TIA1 | circRNA | chr2 | 70441550 | 70441571 | - | 9 | 0 | AGO1,AGO1-4,AGO2,AGO3 | 8mer | ucgcccugaaacucCCGGUCAa | augcacaacaaauuGGCCAGUa |
| NM_022173 | TIA1 | circRNA | chr2 | 70441580 | 70441600 | - | 2 | 0 | AGO1,AGO1-4 | 7mer-m8 | ucgcCCUGAAACUCCCGGUCAa | uuauGGCCAGUG-GGGCCAGUg |
| NM_022173 | TIA1 | circRNA | chr2 | 70444089 | 70444111 | - | 1 | 0 | AGO1-4 | 7mer-m8 | ucgcccugaaACUC-CCGGUCAa | auucaacagaUGGGUGGCCAGUg |
| NM_032822 | FAM136A | circRNA | chr2 | 70524163 | 70524185 | - | 4 | 0 | AGO1-4,AGO2 | 7mer-m8 | ucgcccUGAAACUC-CCGGUCAa | cuuauuAUUUCCUGUGGCCAGUg |
| NM_001130979 | DYSF | circRNA | chr2 | 71797010 | 71797033 | + | 1 | 0 | AGO1-4 | 7mer-m8 | ucgccCUGAA--ACUCCCGGUCAa | gacccGGCUUCCCGGAGGCCAGUg |
| NM_001130981 | DYSF | circRNA | chr2 | 71839840 | 71839862 | + | 1 | 0 | AGO1-4 | 7mer-m8 | ucGCCCUGAAACU-CCCGGUCAa | cgCCGGCCUGUGGUGGGCCAGUg |
| NM_015470 | RAB11FIP5 | circRNA | chr2 | 73302084 | 73302105 | - | 5 | 0 | AGO1-4,AGO2 | 7mer-m8 | ucgcccugaaacuCCCGGUCAa | gccuguguggucuGGGCCAGUc |
| NM_144993 | TET3 | circRNA | chr2 | 74273448 | 74273467 | + | 3 | 0 | AGO1-4,AGO2 | 7mer-m8 | ucgcCCUGAAACUCCCGGUCAa | agauGGAC--UCAGGGCCAGUg |
| NM_144993 | TET3 | circRNA | chr2 | 74273450 | 74273467 | + | 3 | 0 | AGO1-4,AGO2 | 7mer-m8 | ucgcCCUGAAACUCCCGGUCAa | #NAME? |
| NM_144993 | TET3 | circRNA | chr2 | 74328363 | 74328384 | + | 2 | 0 | AGO1-4 | 7mer-m8 | ucgcccugaaaCUCCCGGUCAa | cccacuucacaGAUGGCCAGUg |
| NM_144993 | TET3 | circRNA | chr2 | 74329756 | 74329777 | + | 6 | 0 | AGO1-4,AGO2 | 8mer | ucgcCCUGAAACUCCCGGUCAa | ggauGGGCAGGAAAGGCCAGUa |
| NM_133478 | SLC4A5 | circRNA | chr2 | 74587924 | 74587945 | - | 1 | 0 | AGO1-4 | 7mer-m8 | ucgcccugaaacucCCGGUCAa | uuuucugauguacuGGCCAGUc |
| NM_004082 | DCTN1 | circRNA | chr2 | 74598816 | 74598837 | - | 1 | 0 | AGO1-4 | 8mer | ucGCCCUGAAACUCCCGGUCAa | acUGGGGUGGCUGGGGCCAGUa |
| NR_033935 | DCTN1 | circRNA | chr2 | 74598816 | 74598837 | - | 1 | 0 | AGO1-4 | 8mer | ucGCCCUGAAACUCCCGGUCAa | acUGGGGUGGCUGGGGCCAGUa |
| NM_003761 | VAMP8 | circRNA | chr2 | 85809103 | 85809127 | + | 2 | 0 | AGO2 | 7mer-m8 | ucGCCC--UGA-AACUCCCGGUCAa | gcUGGGAAACUGUUGGUGGCCAGUg |
| NM_015425 | POLR1A | circRNA | chr2 | 86305349 | 86305369 | - | 2 | 0 | AGO1-4,AGO2 | 7mer-m8 | ucgcccUGAAACUCCCGGUCAa | gaguacACUUCCA-GGCCAGUc |
| NR_024204 | LINC00152 | circRNA | chr2 | 87431551 | 87431572 | + | 1 | 0 | AGO1-4 | 7mer-m8 | ucgcccugaaacUCCCGGUCAa | ccuuugaaacaaGGGGCCAGUc |
| NR_024204 | LINC00152 | circRNA | chr2 | 87431596 | 87431617 | + | 1 | 0 | AGO1-4 | 7mer-m8 | ucgcccugaaACUCCCGGUCAa | cuacuucuaaUGCUGGCCAGUc |
| NR_024204 | LINC00152 | circRNA | chr2 | 87820960 | 87820982 | + | 5 | 0 | AGO1-4,AGO2 | 7mer-m8 | ucgcccugAAAC-UCCCGGUCAa | guuucccaUUUGUCUGGCCAGUc |
| NM_020151 | STARD7 | circRNA | chr2 | 96851736 | 96851759 | - | 23 | 1 | AGO1,AGO1-4,AGO2 | 7mer-m8 | ucgcccuGAAACU--CCCGGUCAa | aaguuucCUGUGAAUCGGCCAGUu |
| NM_020151 | STARD7 | circRNA | chr2 | 96852051 | 96852074 | - | 19 | 0 | AGO1,AGO1-4,AGO2 | 7mer-m8 | ucGCCCUGAAACUC--CCGGUCAa | ccUGGAAAGUAUAGUUGGCCAGUu |
| NM_014014 | SNRNP200 | circRNA | chr2 | 96942711 | 96942723 | - | 2 | 0 | AGO1-4 | 7mer-m8 | ucgcccugaAACUCCCGGUCAa | #NAME? |
| NM_014014 | SNRNP200 | circRNA | chr2 | 96964132 | 96964153 | - | 16 | 0 | AGO1-4,AGO2 | 7mer-m8 | ucGCCCUGAAACUCCCGGUCAa | uaCUGUACCUUGCUGGCCAGUg |
| NM_020184 | CNNM4 | circRNA | chr2 | 97474405 | 97474430 | + | 1 | 0 | AGO2 | 8mer | ucGCCCU-GAAAC---UCCCGGUCAa | uuUGGCAGCUCUGUCCUGGGCCAGUa |
| NM_015904 | EIF5B | circRNA | chr2 | 99988155 | 99988175 | + | 4 | 2 | AGO1-4,AGO2 | 7mer-m8 | ucGCCCUGAAACUCCCGGUCAa | auUGGGAAGCU-AUGGCCAGUg |
| NM_017546 | C2orf29 | circRNA | chr2 | 101874355 | 101874382 | + | 9 | 0 | AGO1-4,AGO2 | 7mer-m8 | ucgcCCUGAAACU------CCCGGUCAa | ugauGGACGUUGGAAACAUGGGCCAGUc |
| hsa_circ_0001057 | hsa_circ_000665 | circRNA | chr2 | 101874355 | 101874382 | + | 9 | 0 | AGO1-4,AGO2 | 7mer-m8 | ucgcCCUGAAACU------CCCGGUCAa | ugauGGACGUUGGAAACAUGGGCCAGUc |
| hsa_circ_0001059 | hsa_circ_000312 | circRNA | chr2 | 105924591 | 105924613 | - | 1 | 0 | AGO2 | 7mer-m8 | ucgcccugaaaCUC-CCGGUCAa | uccuguuggagGAGAGGCCAGUg |
| NM_006267 | RANBP2 | circRNA | chr2 | 109080286 | 109080311 | + | 1 | 0 | AGO1-4 | 7mer-m8 | ucGCCCUGA--AACU--CCCGGUCAa | acUGUGAUUUGUUGAUUGGGCCAGUc |
| NM_182588 | RGPD4 | circRNA | chr2 | 109080286 | 109080311 | + | 1 | 0 | AGO1-4 | 7mer-m8 | ucGCCCUGA--AACU--CCCGGUCAa | acUGUGAUUUGUUGAUUGGGCCAGUc |
| NM_006267 | RANBP2 | circRNA | chr2 | 109153950 | 109153971 | + | 1 | 0 | AGO1-4 | 7mer-m8 | ucgcccugaaacUCCCGGUCAa | ccugauaagaaaAUGGCCAGUg |
| NM_182588 | RGPD4 | circRNA | chr2 | 109153950 | 109153971 | + | 1 | 0 | AGO1-4 | 7mer-m8 | ucgcccugaaacUCCCGGUCAa | ccugauaagaaaAUGGCCAGUg |
| NM_001193485 | LIMS1 | circRNA | chr2 | 109303315 | 109303333 | + | 2 | 0 | AGO1-4 | 8mer | ucgcccUGAAACUCCCGGUCAa | uggaaaACUU--A-GGCCAGUa |
| NM_004987 | LIMS1 | circRNA | chr2 | 109303315 | 109303333 | + | 2 | 0 | AGO1-4 | 8mer | ucgcccUGAAACUCCCGGUCAa | uggaaaACUU--A-GGCCAGUa |
| NM_006267 | RANBP2 | circRNA | chr2 | 109303315 | 109303333 | + | 2 | 0 | AGO1-4 | 8mer | ucgcccUGAAACUCCCGGUCAa | uggaaaACUU--A-GGCCAGUa |
| NM_182588 | RGPD4 | circRNA | chr2 | 109303315 | 109303333 | + | 2 | 0 | AGO1-4 | 8mer | ucgcccUGAAACUCCCGGUCAa | uggaaaACUU--A-GGCCAGUa |
| NM_001099289 | SH3RF3 | circRNA | chr2 | 109405229 | 109405248 | + | 1 | 0 | AGO1-4 | 7mer-m8 | ucgccCUGAAACUCCCGGUCAa | auuuaGACUU--AUGGCCAGUg |
| NM_001193485 | LIMS1 | circRNA | chr2 | 109405229 | 109405248 | + | 1 | 0 | AGO1-4 | 7mer-m8 | ucgccCUGAAACUCCCGGUCAa | auuuaGACUU--AUGGCCAGUg |
| NM_006267 | RANBP2 | circRNA | chr2 | 109405229 | 109405248 | + | 1 | 0 | AGO1-4 | 7mer-m8 | ucgccCUGAAACUCCCGGUCAa | auuuaGACUU--AUGGCCAGUg |
| NM_022336 | EDAR | circRNA | chr2 | 109472670 | 109472693 | - | 1 | 0 | AGO1-4 | 7mer-m8 | ucgccCUGAAA--CUCCCGGUCAa | agcaaGAUUUUACCCAGGCCAGUg |
| NM_001099289 | SH3RF3 | circRNA | chr2 | 109485056 | 109485076 | + | 1 | 0 | AGO1-4 | 8mer | ucgcccugaaACUCCCGGUCAa | uugacacacaUGA-GGCCAGUa |
| NM_001193485 | LIMS1 | circRNA | chr2 | 109485056 | 109485076 | + | 1 | 0 | AGO1-4 | 8mer | ucgcccugaaACUCCCGGUCAa | uugacacacaUGA-GGCCAGUa |
| NM_006267 | RANBP2 | circRNA | chr2 | 109485056 | 109485076 | + | 1 | 0 | AGO1-4 | 8mer | ucgcccugaaACUCCCGGUCAa | uugacacacaUGA-GGCCAGUa |
| NM_001193485 | LIMS1 | circRNA | chr2 | 110036196 | 110036216 | + | 1 | 0 | AGO2 | 8mer | ucgcccUGAAACUCCCGGUCAa | uguguuAC-UAGUUGGCCAGUa |
| NM_006267 | RANBP2 | circRNA | chr2 | 110036196 | 110036216 | + | 1 | 0 | AGO2 | 8mer | ucgcccUGAAACUCCCGGUCAa | uguguuAC-UAGUUGGCCAGUa |
| NM_022336 | EDAR | circRNA | chr2 | 110320422 | 110320443 | - | 1 | 0 | AGO2 | 7mer-m8 | ucgcccugaAACUCCCGGUCAa | ugcuucuggUUGUGGGCCAGUc |
| NM_001099289 | SH3RF3 | circRNA | chr2 | 110441114 | 110441135 | + | 1 | 0 | AGO1-4 | 7mer-m8 | ucgcccugaaacuCCCGGUCAa | acuuccaauacauGGGCCAGUc |
| NM_001193485 | LIMS1 | circRNA | chr2 | 110441114 | 110441135 | + | 1 | 0 | AGO1-4 | 7mer-m8 | ucgcccugaaacuCCCGGUCAa | acuuccaauacauGGGCCAGUc |
| NM_006267 | RANBP2 | circRNA | chr2 | 110441114 | 110441135 | + | 1 | 0 | AGO1-4 | 7mer-m8 | ucgcccugaaacuCCCGGUCAa | acuuccaauacauGGGCCAGUc |
| hsa_circ_0056016 | hsa_circ_0056016 | circRNA | chr2 | 110682815 | 110682833 | + | 1 | 0 | AGO1-4 | 8mer | ucgcccUGAAACUCCCGGUCAa | uggaaaACUU--A-GGCCAGUa |
| NM_001037866 | RGPD6 | circRNA | chr2 | 112036613 | 112036634 | - | 1 | 0 | AGO1-4 | 8mer | ucGCCCUGAAACUCCCGGUCAa | uuCGAGACCAGCCUGGCCAGUa |
| NM_001164463 | RGPD8 | circRNA | chr2 | 112036613 | 112036634 | - | 1 | 0 | AGO1-4 | 8mer | ucGCCCUGAAACUCCCGGUCAa | uuCGAGACCAGCCUGGCCAGUa |
| NM_022662 | ANAPC1 | circRNA | chr2 | 112036613 | 112036634 | - | 1 | 0 | AGO1-4 | 8mer | ucGCCCUGAAACUCCCGGUCAa | uuCGAGACCAGCCUGGCCAGUa |
| NM_001037866 | RGPD6 | circRNA | chr2 | 112186941 | 112186963 | - | 8 | 0 | AGO1-4,AGO2 | 7mer-m8 | ucgcccugAAAC-UCCCGGUCAa | guuucccaUUUGUCUGGCCAGUc |
| NM_001164463 | RGPD8 | circRNA | chr2 | 112186941 | 112186963 | - | 8 | 0 | AGO1-4,AGO2 | 7mer-m8 | ucgcccugAAAC-UCCCGGUCAa | guuucccaUUUGUCUGGCCAGUc |
| NM_022662 | ANAPC1 | circRNA | chr2 | 112186941 | 112186963 | - | 8 | 0 | AGO1-4,AGO2 | 7mer-m8 | ucgcccugAAAC-UCCCGGUCAa | guuucccaUUUGUCUGGCCAGUc |
| NM_001037866 | RGPD6 | circRNA | chr2 | 112229516 | 112229537 | - | 1 | 0 | AGO1-4 | 7mer-m8 | ucgcccuGAAACUCCCGGUCAa | ccauuucCUAGUAGGGCCAGUc |
| NM_001164463 | RGPD8 | circRNA | chr2 | 112229516 | 112229537 | - | 1 | 0 | AGO1-4 | 7mer-m8 | ucgcccuGAAACUCCCGGUCAa | ccauuucCUAGUAGGGCCAGUc |
| NM_022662 | ANAPC1 | circRNA | chr2 | 112229516 | 112229537 | - | 1 | 0 | AGO1-4 | 7mer-m8 | ucgcccuGAAACUCCCGGUCAa | ccauuucCUAGUAGGGCCAGUc |
| NM_001037866 | RGPD6 | circRNA | chr2 | 112518934 | 112518955 | - | 1 | 0 | AGO1-4 | 7mer-m8 | ucgcccugaaACUCCCGGUCAa | cuacuucuaaUGCUGGCCAGUc |
| NM_001164463 | RGPD8 | circRNA | chr2 | 112518934 | 112518955 | - | 1 | 0 | AGO1-4 | 7mer-m8 | ucgcccugaaACUCCCGGUCAa | cuacuucuaaUGCUGGCCAGUc |
| NM_022662 | ANAPC1 | circRNA | chr2 | 112518934 | 112518955 | - | 1 | 0 | AGO1-4 | 7mer-m8 | ucgcccugaaACUCCCGGUCAa | cuacuucuaaUGCUGGCCAGUc |
| NM_001037866 | RGPD6 | circRNA | chr2 | 112518979 | 112519000 | - | 1 | 0 | AGO1-4 | 7mer-m8 | ucgcccugaaacUCCCGGUCAa | ccuuugaaacaaGGGGCCAGUc |
| NM_001164463 | RGPD8 | circRNA | chr2 | 112518979 | 112519000 | - | 1 | 0 | AGO1-4 | 7mer-m8 | ucgcccugaaacUCCCGGUCAa | ccuuugaaacaaGGGGCCAGUc |
| NM_022662 | ANAPC1 | circRNA | chr2 | 112518979 | 112519000 | - | 1 | 0 | AGO1-4 | 7mer-m8 | ucgcccugaaacUCCCGGUCAa | ccuuugaaacaaGGGGCCAGUc |
| NM_198581 | ZC3H6 | circRNA | chr2 | 113095808 | 113095830 | + | 1 | 0 | AGO1-4 | 7mer-m8 | ucgcccUGAAAC-UCCCGGUCAa | ugagccACUGUGCCUGGCCAGUc |
| NM_032309 | CHCHD5 | circRNA | chr2 | 113343586 | 113343613 | + | 3 | 0 | AGO1-4,AGO2 | 7mer-m8 | ucGCCCUGAAACUC------CCGGUCAa | gcCGGGAGCUGGAGCAGUAUGGCCAGUg |
| NM_012455 | PSD4 | circRNA | chr2 | 113939937 | 113939959 | + | 1 | 0 | AGO1-4 | 7mer-m8 | ucgcccugaaacUC-CCGGUCAa | cccaguuucuccAGCGGCCAGUg |
| NM_015282 | CLASP1 | circRNA | chr2 | 122106252 | 122106273 | - | 1 | 0 | AGO2 | 7mer-m8 | ucgcccugaaacucCCGGUCAa | gcugcguccacacuGGCCAGUu |
| NM_017969 | IWS1 | circRNA | chr2 | 128262695 | 128262716 | - | 2 | 0 | AGO1-4 | 7mer-m8 | ucgcccugaaacucCCGGUCAa | ccuccgaggcaccaGGCCAGUg |
| NM_017969 | IWS1 | circRNA | chr2 | 128262773 | 128262794 | - | 5 | 1 | AGO1-4,AGO2 | 7mer-m8 | ucgcccugaaacucCCGGUCAa | cccccaaggcaccaGGCCAGUg |
| NM_001199140 | AMMECR1L | circRNA | chr2 | 128619941 | 128619962 | - | 2 | 0 | AGO1-4 | 8mer | ucgCCCUGAAACUCCCGGUCAa | gcaGUGAUGAGGGCGGCCAGUa |
| NM_001199140 | AMMECR1L | circRNA | chr2 | 128621278 | 128621300 | - | 13 | 0 | AGO1,AGO1-4,AGO2 | 7mer-m8 | ucgcccugaaaCUC-CCGGUCAa | auucacagggaGAGUGGCCAGUu |
| NM_001145928 | SAP130 | circRNA | chr2 | 128712564 | 128712586 | - | 1 | 0 | AGO1-4 | 7mer-m8 | ucgcCCUGAA--ACUCCCGGUCAa | caauGGAUAUCAUGA-GGCCAGUu |
| NR_027671 | UGGT1 | circRNA | chr2 | 128914826 | 128914848 | + | 3 | 0 | AGO1-4,AGO2 | 8mer | ucgcccuGAAACU--CCCGGUCAa | #NAME? |
| hsa_circ_0001067 | hsa_circ_000910 | circRNA | chr2 | 128914826 | 128914848 | + | 3 | 0 | AGO1-4,AGO2 | 8mer | ucgcccuGAAACU--CCCGGUCAa | #NAME? |
| NR_027671 | UGGT1 | circRNA | chr2 | 128939819 | 128939836 | + | 1 | 0 | AGO2 | 7mer-m8 | ucgCCCUGAAACUCCCGGUCAa | cagGGUACU----GGGCCAGUc |
| NM_005168 | RND3 | circRNA | chr2 | 151343256 | 151343277 | - | 5 | 0 | AGO1-4,AGO2 | 7mer-m8 | ucGCCCUGAAACUCCCGGUCAa | uuUGAGAAUUACACGGCCAGUu |
| NM_007366 | PLA2R1 | circRNA | chr2 | 160843675 | 160843696 | - | 1 | 0 | AGO2 | 7mer-m8 | ucgcccugaaacucCCGGUCAa | ucagagccuggucuGGCCAGUu |
| NM_016836 | RBMS1 | circRNA | chr2 | 161169690 | 161169711 | - | 2 | 0 | AGO1-4 | 7mer-m8 | ucgcccugaaACUCCCGGUCAa | gugucugcccUGAAGGCCAGUg |
| NM_172070 | UBR3 | circRNA | chr2 | 170930021 | 170930042 | + | 6 | 0 | AGO1-4,AGO2 | 7mer-m8 | ucgcccugaaacucCCGGUCAa | cuccugucuggauuGGCCAGUu |
| NM_025000 | DCAF17 | circRNA | chr2 | 172339021 | 172339043 | + | 2 | 0 | AGO1-4,AGO2 | 7mer-m8 | ucgcccugaaACUC-CCGGUCAa | acaagaccagUAAGAGGCCAGUg |
| NM_018981 | DNAJC10 | circRNA | chr2 | 183584843 | 183584864 | + | 6 | 0 | AGO1-4,AGO2 | 8mer | ucgcccugaaaCUCCCGGUCAa | aggauaaucaaGGUGGCCAGUa |
| NM_205842 | NCKAP1 | circRNA | chr2 | 183793544 | 183793565 | - | 13 | 0 | AGO1-4,AGO2 | 8mer | ucgcccugaaacucCCGGUCAa | ucuuugccaacacuGGCCAGUa |
| NM_002210 | ITGAV | circRNA | chr2 | 187532466 | 187532485 | + | 4 | 0 | AGO1-4,AGO2 | 7mer-m8 | ucgccCUGAAACUCCCGGUCAa | aagaaGAUGUU--GGGCCAGUu |
| NM_000090 | COL3A1 | circRNA | chr2 | 189875489 | 189875512 | + | 6 | 0 | AGO1-4,AGO2 | 7mer-m8 | ucgcccUGAAACU--CCCGGUCAa | uugcauACAUGGAUCAGGCCAGUg |
| NM_000393 | COL5A2 | circRNA | chr2 | 189931125 | 189931146 | - | 1 | 0 | AGO2 | 7mer-m8 | ucgcCCUGAAACUCCCGGUCAa | aguuGGUCCUCCAGGGCCAGUg |
| NM_001172509 | SATB2 | circRNA | chr2 | 200213468 | 200213489 | - | 2 | 0 | AGO1-4 | 7mer-m8 | ucgcccugaaaCUCCCGGUCAa | gaugagcugaaGAGGGCCAGUg |
| NM_015049 | TRAK2 | circRNA | chr2 | 202245522 | 202245543 | - | 3 | 0 | AGO1-4 | 7mer-m8 | ucgcccugaaACUCCCGGUCAa | acccuccugaUGUUGGCCAGUu |
| NM_020919 | ALS2 | circRNA | chr2 | 202626349 | 202626370 | - | 2 | 0 | AGO1,AGO1-4 | 7mer-m8 | ucgcCCUGAAACUCCCGGUCAa | gagaGAAUUCUGCUGGCCAGUg |
| hsa_circ_0001093 | hsa_circ_000321 | circRNA | chr2 | 202626349 | 202626370 | - | 2 | 0 | AGO1,AGO1-4 | 7mer-m8 | ucgcCCUGAAACUCCCGGUCAa | gagaGAAUUCUGCUGGCCAGUg |
| NM_017759 | INO80D | circRNA | chr2 | 206866084 | 206866109 | - | 9 | 0 | AGO1,AGO1-4,AGO2 | 7mer-m8 | ucGCCCUG---AA-ACUCCCGGUCAa | uuUGGGACUAUUUAUAAAGGCCAGUu |
| NM_212482 | FN1 | circRNA | chr2 | 216226735 | 216226756 | - | 14 | 0 | AGO1-4,AGO2 | 7mer-m8 | ucgccCUGAAACUCCCGGUCAa | ccgaaGGCACUACUGGCCAGUc |
| NM_018441 | PECR | circRNA | chr2 | 216930124 | 216930147 | - | 3 | 0 | AGO1-4,AGO2 | 7mer-m8 | ucgcccugaAACUC--CCGGUCAa | ggugaacaaUGGAGGAGGCCAGUu |
| NM_000599 | IGFBP5 | circRNA | chr2 | 217538982 | 217539003 | - | 14 | 0 | AGO1-4,AGO2 | 8mer | ucGCCCUG-AAACUCCCGGUCAa | uuCUGUAUGUCUUA-GGCCAGUa |
| NM_000599 | IGFBP5 | circRNA | chr2 | 217539524 | 217539545 | - | 2 | 0 | AGO1-4,AGO2 | 7mer-m8 | ucgcccugaaacucCCGGUCAa | ucuuccuccauucuGGCCAGUc |
| NM_022648 | TNS1 | circRNA | chr2 | 218665312 | 218665333 | - | 1 | 0 | AGO2 | 7mer-m8 | ucgcCCUGAAACUCCCGGUCAa | acucGCGUUUCCUUGGCCAGUg |
| NM_022648 | TNS1 | circRNA | chr2 | 218669156 | 218669180 | - | 1 | 0 | AGO1-4 | 7mer-m8 | ucGCCCUGAAAC---UCCCGGUCAa | ccCUGCCCCUUGCCCAGGGCCAGUg |
| NM_022648 | TNS1 | circRNA | chr2 | 218700869 | 218700890 | - | 2 | 0 | AGO1-4,AGO2 | 7mer-m8 | ucgcCCUGAAACUCCCGGUCAa | cugcGGAGGCGGGCGGCCAGUg |
| NM_022648 | TNS1 | circRNA | chr2 | 218827606 | 218827624 | - | 1 | 0 | AGO1-4 | 7mer-m8 | ucgccCUGAAACUCCCGGUCAa | uaguaGAAUUU---GGCCAGUg |
| NM_022648 | TNS1 | circRNA | chr2 | 218866562 | 218866584 | - | 1 | 0 | AGO2 | 7mer-m8 | ucgcccuGAA-ACUCCCGGUCAa | cccuuucCUUGUUCUGGCCAGUc |
| NM_000784 | CYP27A1 | circRNA | chr2 | 219674354 | 219674375 | + | 1 | 0 | AGO1-4 | 7mer-m8 | ucgcccugaaacucCCGGUCAa | augcacgugaaccuGGCCAGUg |
| NM_000784 | CYP27A1 | circRNA | chr2 | 219677731 | 219677754 | + | 1 | 0 | AGO1-4 | 7mer-m8 | ucGCCCUGAA-ACU-CCCGGUCAa | acCUGCACUUCUUACUGGCCAGUg |
| NM_015680 | CNPPD1 | circRNA | chr2 | 220039616 | 220039628 | - | 1 | 0 | AGO1-4 | 8mer | ucgcccugaaACUCCCGGUCAa | #NAME? |
| NM_012100 | DNPEP | circRNA | chr2 | 220251445 | 220251467 | - | 1 | 0 | AGO1-4 | 8mer | ucgcccUGA-AACUCCCGGUCAa | gcuuuuGCUGUAGGGGGCCAGUa |
| NM_005876 | SPEG | circRNA | chr2 | 220308483 | 220308505 | + | 1 | 0 | AGO2 | 7mer-m8 | ucgcccugAAACUC-CCGGUCAa | ugcaugugUGUGUGUGGCCAGUg |
| NM_001243252 | EFHD1 | circRNA | chr2 | 233546296 | 233546319 | + | 1 | 0 | AGO1-4 | 8mer | ucgccCUGAA-ACU-CCCGGUCAa | uccaaGCCUUGUCAUCGGCCAGUa |
| NM_001037131 | AGAP1 | circRNA | chr2 | 236957799 | 236957824 | + | 2 | 0 | AGO1-4 | 7mer-m8 | ucGCCCUGAA---ACU-CCCGGUCAa | cuCUGGACCUGGAUGACUGGCCAGUc |
| NM_004369 | COL6A3 | circRNA | chr2 | 238285930 | 238285951 | - | 2 | 0 | AGO1-4 | 7mer-m8 | ucgcccUGAAACUCCCGGUCAa | cagccaAUCUUGUGGGCCAGUu |
| NR_037904 | UBE2F-SCLY | circRNA | chr2 | 239005462 | 239005483 | + | 1 | 0 | AGO1-4 | 7mer-m8 | ucgcccugaaACUCCCGGUCAa | ugccgagugcUGAUGGCCAGUg |
| NM_000030 | AGXT | circRNA | chr2 | 241808220 | 241808244 | + | 1 | 0 | AGO1-4 | 7mer-m8 | ucgccCUGAAAC---UCCCGGUCAa | uuccaGGCUUUGGCCAAGGCCAGUg |
| NM_005336 | HDLBP | circRNA | chr2 | 242167241 | 242167262 | - | 1 | 0 | AGO1-4 | 7mer-m8 | ucgcccUGAAACUCCCGGUCAa | accuccACGGGGGAGGCCAGUg |
| NM_178326 | ATG4B | circRNA | chr2 | 242594704 | 242594725 | + | 1 | 0 | AGO2 | 7mer-m8 | ucgcccugaaacucCCGGUCAa | aaggcaaguccauaGGCCAGUg |
| NM_032329 | ING5 | circRNA | chr2 | 242664547 | 242664567 | + | 7 | 0 | AGO1,AGO1-4,AGO2 | 7mer-m8 | ucgcccUGAAACUCCCGGUCAa | uuaguaACUCCG-UGGCCAGUu |
| NM_152783 | D2HGDH | circRNA | chr2 | 242691089 | 242691111 | + | 1 | 0 | AGO1-4 | 7mer-m8 | ucgcccugaAACUC-CCGGUCAa | agccuguggUUAAGCGGCCAGUc |
| NM_182916 | TRNT1 | circRNA | chr3 | 3170739 | 3170760 | + | 3 | 0 | AGO1-4,AGO2 | 7mer-m8 | ucgcccugaaacucCCGGUCAa | guaucauuggcacaGGCCAGUg |
| NM_182760 | SUMF1 | circRNA | chr3 | 4247899 | 4247920 | - | 2 | 0 | AGO1-4 | 7mer-m8 | ucgcccugaaacUCCCGGUCAa | ucacccuuccccAAGGCCAGUc |
| hsa_circ_0064198 | hsa_circ_0064198 | circRNA | chr3 | 10035103 | 10035124 | + | 1 | 0 | AGO1-4 | 8mer | ucgcccUGAAACU-CCCGGUCAa | uagaaaAC-UGGAUGGGCCAGUa |
| NR_024272 | SEC13 | circRNA | chr3 | 10346820 | 10346840 | - | 3 | 1 | AGO1-4 | 7mer-m8 | ucgcCCUGAAACUCCCGGUCAa | #NAME? |
| NM_006395 | ATG7 | circRNA | chr3 | 11354843 | 11354864 | + | 1 | 0 | AGO1-4 | 7mer-m8 | ucgcccugAAACUCCCGGUCAa | accucucaUUCAGGGGCCAGUg |
| NM_006395 | ATG7 | circRNA | chr3 | 11398630 | 11398653 | + | 1 | 0 | AGO1-4 | 7mer-m8 | ucgcccUGAA--ACUCCCGGUCAa | ucaaacACUUCCCUAUGGCCAGUg |
| NM_024923 | NUP210 | circRNA | chr3 | 13413415 | 13413436 | - | 2 | 0 | AGO2 | 7mer-m8 | ucgcccugaaacUCCCGGUCAa | cucaugcccggcGGGGCCAGUg |
| NM_022340 | ZFYVE20 | circRNA | chr3 | 15112891 | 15112914 | - | 1 | 0 | AGO2 | 8mer | ucgCCCUGAAA--CUCCCGGUCAa | agaGGGUUUCUCAGAUGGCCAGUa |
| NM_033083 | EAF1 | circRNA | chr3 | 15481294 | 15481313 | + | 1 | 0 | AGO1-4 | 7mer-m8 | ucgccCUGAAACUCCCGGUCAa | caccuGGCUU--GUGGCCAGUc |
| hsa_circ_0001274 | hsa_circ_001828 | circRNA | chr3 | 17053351 | 17053372 | + | 5 | 0 | AGO1-4,AGO2 | 8mer | ucgccCUGAAACUCCCGGUCAa | gggauGAAUUCAUCGGCCAGUa |
| NM_001134381 | TBC1D5 | circRNA | chr3 | 17200328 | 17200349 | - | 1 | 0 | AGO1-4 | 7mer-m8 | ucgcccugaaacUCCCGGUCAa | ucccugggcagcAGGGCCAGUg |
| NM_014744 | TBC1D5 | circRNA | chr3 | 17200328 | 17200349 | - | 1 | 0 | AGO1-4 | 7mer-m8 | ucgcccugaaacUCCCGGUCAa | ucccugggcagcAGGGCCAGUg |
| NM_138410 | CMTM7 | circRNA | chr3 | 32496310 | 32496333 | + | 2 | 0 | AGO2 | 7mer-m8 | ucgcccUGAAACU--CCCGGUCAa | aauaaaAUAUUGACUCGGCCAGUu |
| NM_000404 | GLB1 | circRNA | chr3 | 33106967 | 33106988 | - | 2 | 0 | AGO2 | 7mer-m8 | ucgcccugaAACUCCCGGUCAa | cuaucagaaUGGAGGGCCAGUu |
| NM_014517 | UBP1 | circRNA | chr3 | 33431541 | 33431565 | - | 5 | 0 | AGO1-4,AGO2 | 8mer | ucgcccUGAAAC--UC-CCGGUCAa | ccugcuGCUCUGGCAGAGGCCAGUa |
| NM_001162429 | PDCD6IP | circRNA | chr3 | 33907875 | 33907896 | + | 3 | 0 | AGO1-4,AGO2 | 8mer | ucgcccugaaacUCCCGGUCAa | auccuuaugcguAUGGCCAGUa |
| NM_014831 | TRANK1 | circRNA | chr3 | 36874997 | 36875018 | - | 1 | 0 | AGO1-4 | 7mer-m8 | ucgcccugaaacucCCGGUCAa | ucugcuaucaaacuGGCCAGUu |
| NM_020839 | WDR48 | circRNA | chr3 | 39136495 | 39136516 | + | 3 | 0 | AGO1-4,AGO2 | 7mer-m8 | ucgcccugaaacucCCGGUCAa | gaagacugacaacaGGCCAGUg |
| NM_022842 | CDCP1 | circRNA | chr3 | 45134937 | 45134957 | - | 6 | 0 | AGO1-4,AGO2 | 7mer-m8 | ucgcccUGAAACUCCCGGUCAa | ucagcuACCUCG-UGGCCAGUg |
| NM_015175 | NBEAL2 | circRNA | chr3 | 47033346 | 47033370 | + | 2 | 0 | AGO1-4,AGO2 | 7mer-m8 | ucGCCCUGAAACUC---CCGGUCAa | gcUGGCAUGUGAAGACCGGCCAGUg |
| NM_002673 | PLXNB1 | circRNA | chr3 | 48453350 | 48453369 | - | 1 | 0 | AGO2 | 7mer-m8 | ucgcCCUGAAACUCCCGGUCAa | aaguGGA--UAAGGGGCCAGUg |
| NM_002673 | PLXNB1 | circRNA | chr3 | 48457835 | 48457856 | - | 1 | 0 | AGO2 | 7mer-m8 | ucgcccugaaacUCCCGGUCAa | gugugcaucaccGGGGCCAGUg |
| NM_006321 | ARIH2 | circRNA | chr3 | 48964169 | 48964195 | + | 3 | 0 | AGO1-4 | 7mer-m8 | ucGCCCUGAA-----ACUCCCGGUCAa | gaUGGGGUUUCGCCAUGUUGGCCAGUc |
| NM_006321 | ARIH2 | circRNA | chr3 | 48964265 | 48964286 | + | 3 | 0 | AGO1-4 | 8mer | ucgcccugaaacucCCGGUCAa | gagucaccacacccGGCCAGUa |
| NM_018031 | WDR6 | circRNA | chr3 | 49052335 | 49052356 | + | 1 | 0 | AGO2 | 7mer-m8 | ucgcccugAAACUCCCGGUCAa | ggccaccaUCUCGUGGCCAGUg |
| NM_018031 | WDR6 | circRNA | chr3 | 49053045 | 49053066 | + | 17 | 0 | AGO1,AGO1-4,AGO2 | 8mer | ucgcccugaaacucCCGGUCAa | acccagcauagccaGGCCAGUa |
| NM_017730 | QRICH1 | circRNA | chr3 | 49094912 | 49094935 | - | 3 | 0 | AGO1-4,AGO2 | 7mer-m8 | ucGCC-CUGAAAC-UCCCGGUCAa | agCGGCGGGUUGGCACGGCCAGUg |
| NM_017730 | QRICH1 | circRNA | chr3 | 49095022 | 49095043 | - | 4 | 0 | AGO1-4,AGO2 | 7mer-m8 | ucgcccugaAACUCCCGGUCAa | cucagcuggUGGCUGGCCAGUc |
| NM_002292 | LAMB2 | circRNA | chr3 | 49162800 | 49162821 | - | 1 | 0 | AGO1-4 | 7mer-m8 | ucgcccugaaacucCCGGUCAa | accgcugccagcguGGCCAGUg |
| NM_002292 | LAMB2 | circRNA | chr3 | 49167364 | 49167387 | - | 3 | 0 | AGO1-4 | 7mer-m8 | ucGCCCUGA--AACUCCCGGUCAa | acUGGGACUGGUCUCCGGCCAGUg |
| NM_006545 | NPRL2 | circRNA | chr3 | 50385655 | 50385672 | - | 1 | 0 | AGO1-4 | 7mer-m8 | ucgCCCUGAAACUCCCGGUCAa | #NAME? |
| NM_007024 | TMEM115 | circRNA | chr3 | 50392549 | 50392576 | - | 2 | 0 | AGO1,AGO3 | 7mer-m8 | ucGCC-CUGAAACUC-----CCGGUCAa | ccCGGAGCCUCUGAGGCAUCGGCCAGUc |
| NM_007024 | TMEM115 | circRNA | chr3 | 50396599 | 50396618 | - | 1 | 0 | AGO1-4 | 7mer-m8 | ucGCCCUGAAACUCCCGGUCAa | acCAGGGCCAU-A-GGCCAGUg |
| NM_015106 | RAD54L2 | circRNA | chr3 | 51690006 | 51690027 | + | 1 | 0 | AGO2 | 7mer-m8 | ucgcccugaaacucCCGGUCAa | gaugaaaagccuguGGCCAGUg |
| NM_001947 | DUSP7 | circRNA | chr3 | 52083069 | 52083090 | - | 10 | 0 | AGO1-4,AGO2 | 7mer-m8 | ucgcccugaaaCUCCCGGUCAa | guaugccagaaGGUGGCCAGUu |
| NM_001161581 | POC1A | circRNA | chr3 | 52183331 | 52183352 | - | 1 | 0 | AGO2 | 7mer-m8 | ucgcccugaaACUCCCGGUCAa | acuucugcagUGAUGGCCAGUc |
| NM_015426 | POC1A | circRNA | chr3 | 52183331 | 52183352 | - | 1 | 0 | AGO2 | 7mer-m8 | ucgcccugaaACUCCCGGUCAa | acuucugcagUGAUGGCCAGUc |
| NM_000688 | ALAS1 | circRNA | chr3 | 52233432 | 52233453 | + | 1 | 0 | AGO1-4 | 7mer-m8 | ucgcccugaaacucCCGGUCAa | aaagaaaccccuccGGCCAGUg |
| NM_025222 | WDR82 | circRNA | chr3 | 52290618 | 52290642 | - | 23 | 0 | AGO1,AGO1-4,AGO2 | 8mer | ucgcccUGAA---ACUCCCGGUCAa | caucacACUUACCUGUGGGCCAGUa |
| NM_006254 | PRKCD | circRNA | chr3 | 53223134 | 53223156 | + | 1 | 0 | AGO1-4 | 7mer-m8 | ucGCCCUGAAACU-CCCGGUCAa | uaCGAGAUGCUCAUUGGCCAGUc |
| NM_002841 | PTPRG | circRNA | chr3 | 61788185 | 61788206 | + | 1 | 0 | AGO1-4 | 8mer | ucgcccugaaacucCCGGUCAa | ucuuagguaaaguuGGCCAGUa |
| NM_002841 | PTPRG | circRNA | chr3 | 61788443 | 61788466 | + | 2 | 0 | AGO1-4 | 7mer-m8 | ucgcccUGAAACUC--CCGGUCAa | auggccAUUAUGAGAAGGCCAGUu |
| NM_002841 | PTPRG | circRNA | chr3 | 61800601 | 61800623 | + | 1 | 0 | AGO1-4 | 7mer-m8 | ucgcccUGAAACUC-CCGGUCAa | gcugaaGCUUAGAGUGGCCAGUg |
| NM_198859 | PRICKLE2 | circRNA | chr3 | 64082369 | 64082390 | - | 1 | 0 | AGO1-4 | 7mer-m8 | ucgcccugaaacUCCCGGUCAa | aaaaacugacacAUGGCCAGUg |
| NM_198859 | PRICKLE2 | circRNA | chr3 | 64085019 | 64085040 | - | 1 | 0 | AGO1-4 | 7mer-m8 | ucgCCCUGAAACUCCCGGUCAa | ggaGGGACCUGUACGGCCAGUg |
| NM_032505 | KBTBD8 | circRNA | chr3 | 67059673 | 67059693 | + | 4 | 0 | AGO1-4,AGO2 | 7mer-m8 | ucgcCCUGAAACUCCCGGUCAa | gauaGGAGGGU-AUGGCCAGUu |
| NM_173654 | C3orf64 | circRNA | chr3 | 69061869 | 69061890 | - | 1 | 0 | AGO1-4 | 8mer | ucgcccugaaacucCCGGUCAa | aggaaaaguagccuGGCCAGUa |
| hsa_circ_0006630 | hsa_circ_0006630 | circRNA | chr3 | 97618656 | 97618677 | + | 1 | 0 | AGO1-4 | 7mer-m8 | ucgcccugaaacucCCGGUCAa | uuguagaagaguguGGCCAGUu |
| NM_032359 | C3orf26 | circRNA | chr3 | 99561705 | 99561731 | + | 1 | 0 | AGO2 | 7mer-m8 | ucgcCCUGAAACUC-----CCGGUCAa | ugaaGUACUUAGAGGUGUUGGCCAGUu |
| NM_032359 | C3orf26 | circRNA | chr3 | 99673174 | 99673193 | + | 1 | 0 | AGO1-4 | 8mer | ucgcccUGAAACUCCCGGUCAa | agacacACUUU--GGGCCAGUa |
| NM_032359 | C3orf26 | circRNA | chr3 | 99732034 | 99732055 | + | 1 | 0 | AGO1-4 | 7mer-m8 | ucgcccugAAACUCCCGGUCAa | gcagcccaUUUCAAGGCCAGUg |
| NM_014820 | TOMM70A | circRNA | chr3 | 100119520 | 100119541 | - | 2 | 0 | AGO1-4,AGO2 | 7mer-m8 | ucgcccugaaacUCCCGGUCAa | accccggagggcAGGGCCAGUc |
| NM_015429 | ABI3BP | circRNA | chr3 | 100547295 | 100547314 | - | 2 | 0 | AGO1-4 | 7mer-m8 | ucgcccUGAAACUCCCGGUCAa | uuuauuAUUUU--UGGCCAGUu |
| NM_017699 | SIDT1 | circRNA | chr3 | 113321894 | 113321915 | + | 1 | 0 | AGO1-4 | 7mer-m8 | ucgcccugaaacuCCCGGUCAa | auggugggccaccGGGCCAGUc |
| hsa_circ_0001328 | hsa_circ_001134 | circRNA | chr3 | 113321894 | 113321915 | + | 1 | 0 | AGO1-4 | 7mer-m8 | ucgcccugaaacuCCCGGUCAa | auggugggccaccGGGCCAGUc |
| NM_005335 | HCLS1 | circRNA | chr3 | 121355309 | 121355330 | - | 2 | 0 | AGO1-4 | 8mer | ucgcCCUGAAACUCCCGGUCAa | ccaaGGGCUUUGGUGGCCAGUa |
| NM_198402 | PTPLB | circRNA | chr3 | 123213528 | 123213549 | - | 9 | 0 | AGO1,AGO1-4,AGO2 | 7mer-m8 | ucgcccugaaacucCCGGUCAa | acauaaaacacccuGGCCAGUu |
| NM_053025 | MYLK | circRNA | chr3 | 123426616 | 123426637 | - | 1 | 0 | AGO1-4 | 8mer | ucgcccugaaACUCCCGGUCAa | agcccuggcaUGCCGGCCAGUa |
| NM_002213 | ITGB5 | circRNA | chr3 | 124540203 | 124540224 | - | 3 | 0 | AGO1-4 | 7mer-m8 | ucgcccugaaaCUCCCGGUCAa | ugcagccacacGAUGGCCAGUg |
| NM_020733 | HEG1 | circRNA | chr3 | 124689264 | 124689283 | - | 16 | 0 | AGO1-4,AGO2 | 8mer | ucGCCCUGAAACUCCCGGUCAa | aaCGUGA--AUGUGGGCCAGUa |
| NM_024628 | SLC12A8 | circRNA | chr3 | 124953851 | 124953872 | - | 3 | 0 | AGO1-4 | 7mer-m8 | ucgcccUGAAACUCCCGGUCAa | gaaaucAUUGCGGGGGCCAGUg |
| NM_021964 | ZNF148 | circRNA | chr3 | 125032378 | 125032398 | - | 2 | 0 | AGO1-4 | 7mer-m8 | ucgcCCUGAAACUCCCGGUCAa | ggguGGAGUGU-CUGGCCAGUc |
| hsa_circ_0001333 | hsa_circ_001768 | circRNA | chr3 | 125032378 | 125032398 | - | 2 | 0 | AGO1-4 | 7mer-m8 | ucgcCCUGAAACUCCCGGUCAa | ggguGGAGUGU-CUGGCCAGUc |
| NM_001040653 | ZXDC | circRNA | chr3 | 126178196 | 126178217 | - | 5 | 0 | AGO1-4 | 7mer-m8 | ucgcccugaaacucCCGGUCAa | aaauaagcaaagcaGGCCAGUu |
| NM_013336 | SEC61A1 | circRNA | chr3 | 127778956 | 127778975 | + | 5 | 0 | AGO1,AGO1-4,AGO2 | 7mer-m8 | ucgcccUGAAACUCCCGGUCAa | aucauuACUAU--CGGCCAGUc |
| NM_013336 | SEC61A1 | circRNA | chr3 | 127785825 | 127785846 | + | 9 | 0 | AGO1-4,AGO2 | 8mer | ucgcccugaaacucCCGGUCAa | cggcccgcuaccguGGCCAGUa |
| NM_021937 | EEFSEC | circRNA | chr3 | 128127398 | 128127419 | + | 1 | 0 | AGO2 | 7mer-m8 | ucgcccugaaACUCCCGGUCAa | guuggccaccUGCAGGCCAGUc |
| NM_002950 | RPN1 | circRNA | chr3 | 128339159 | 128339180 | - | 2 | 0 | AGO1-4 | 7mer-m8 | ucgcccugaaACUCCCGGUCAa | agguugugcaUGGAGGCCAGUg |
| NM_004637 | RAB7A | circRNA | chr3 | 128525347 | 128525368 | + | 13 | 0 | AGO1-4,AGO2 | 7mer-m8 | ucgcccugaaacucCCGGUCAa | gaguuucucauccaGGCCAGUc |
| hsa_circ_0001337 | hsa_circ_001135 | circRNA | chr3 | 128525347 | 128525368 | + | 13 | 0 | AGO1-4,AGO2 | 7mer-m8 | ucgcccugaaacucCCGGUCAa | gaguuucucauccaGGCCAGUc |
| hsa_circ_0001339 | hsa_circ_001859 | circRNA | chr3 | 128889187 | 128889207 | - | 15 | 1 | AGO1-4,AGO2 | 7mer-m8 | ucgcccUGAAACUCCCGGUCAa | gaggcaACUCCCA-GGCCAGUg |
| NM_015268 | DNAJC13 | circRNA | chr3 | 132257142 | 132257164 | + | 5 | 0 | AGO1-4,AGO2 | 7mer-m8 | ucgcccUGAAAC-UCCCGGUCAa | caauaaACGCUGAAAGGCCAGUg |
| NM_001005861 | RYK | circRNA | chr3 | 133894510 | 133894535 | - | 2 | 1 | AGO1-4,AGO2 | 7mer-m8 | ucgCCCUGAAACU----CCCGGUCAa | uggGGGACAAUGAAAACAGGCCAGUu |
| NM_016201 | AMOTL2 | circRNA | chr3 | 134086489 | 134086512 | - | 2 | 0 | AGO1-4 | 7mer-m8 | ucgcccUGAAAC--UCCCGGUCAa | ccaccuGCUGUGGAGGGGCCAGUg |
| NM_020191 | MRPS22 | circRNA | chr3 | 139074562 | 139074583 | + | 1 | 2 | AGO1-4 | 7mer-m8 | ucgcccugaaaCUCCCGGUCAa | ugcuccauccaGAUGGCCAGUc |
| NR_023350 | COPB2 | circRNA | chr3 | 139092564 | 139092586 | - | 5 | 0 | AGO1-4,AGO2 | 7mer-m8 | ucgCCCUGAAACUC-CCGGUCAa | gagGGUAUGGUGCGUGGCCAGUc |
| NM_019001 | XRN1 | circRNA | chr3 | 142095394 | 142095416 | - | 1 | 0 | AGO2 | 7mer-m8 | ucGCCCUG-AAACUCCCGGUCAa | ccCAGGAUAUGUGUUGGCCAGUc |
| NM_014220 | TM4SF1 | circRNA | chr3 | 149093260 | 149093281 | - | 8 | 0 | AGO1-4,AGO2 | 7mer-m8 | ucgccCUGAAACUCCCGGUCAa | gucuuGAUUCCCUCGGCCAGUg |
| NM_007289 | MME | circRNA | chr3 | 154834498 | 154834520 | + | 8 | 0 | AGO1-4 | 8mer | ucgcccugAAACUC-CCGGUCAa | cagacauaUAUGGGUGGCCAGUa |
| NM_024996 | GFM1 | circRNA | chr3 | 158399807 | 158399828 | + | 3 | 0 | AGO1-4,AGO2 | 8mer | ucgccCUGAAACUCCCGGUCAa | aaucaGGUGGUGCAGGCCAGUa |
| NM_024665 | TBL1XR1 | circRNA | chr3 | 176743182 | 176743203 | - | 22 | 0 | AGO1-4,AGO2 | 7mer-m8 | ucgcccugaaacUCCCGGUCAa | cccacuugaaccAUGGCCAGUc |
| NM_024665 | TBL1XR1 | circRNA | chr3 | 176826663 | 176826682 | - | 2 | 0 | AGO1-4 | 7mer-m8 | ucgcccUGAAACUCCCGGUCAa | gccacaACUU--AUGGCCAGUg |
| NM_024665 | TBL1XR1 | circRNA | chr3 | 176868032 | 176868052 | - | 1 | 0 | AGO1-4 | 7mer-m8 | ucgcccuGAAACUCCCGGUCAa | uaugucuCUCU-AAGGCCAGUu |
| NM_024665 | TBL1XR1 | circRNA | chr3 | 176909187 | 176909207 | - | 1 | 0 | AGO2 | 7mer-m8 | ucGCCCUGAAACUCCCGGUCAa | ugUGUGAAUUCUA-GGCCAGUg |
| NM_022470 | ZMAT3 | circRNA | chr3 | 178740352 | 178740376 | - | 27 | 0 | AGO1,AGO1-4,AGO2,AGO3,AGO4 | 8mer | ucgCCCUGAA--ACU-CCCGGUCAa | uuaGGGCUUUUCUUAUUGGCCAGUa |
| NM_152240 | ZMAT3 | circRNA | chr3 | 178740352 | 178740376 | - | 27 | 0 | AGO1,AGO1-4,AGO2,AGO3,AGO4 | 8mer | ucgCCCUGAA--ACU-CCCGGUCAa | uuaGGGCUUUUCUUAUUGGCCAGUa |
| NM_022470 | ZMAT3 | circRNA | chr3 | 178742938 | 178742960 | - | 4 | 0 | AGO1-4,AGO2 | 7mer-m8 | ucgcccUGAAACUC-CCGGUCAa | uguguuACUCCAAGUGGCCAGUu |
| NM_152240 | ZMAT3 | circRNA | chr3 | 178742938 | 178742960 | - | 4 | 0 | AGO1-4,AGO2 | 7mer-m8 | ucgcccUGAAACUC-CCGGUCAa | uguguuACUCCAAGUGGCCAGUu |
| NM_006218 | PIK3CA | circRNA | chr3 | 178928040 | 178928066 | + | 4 | 0 | AGO1-4,AGO2 | 8mer | ucgccCUGAAACU-----CCCGGUCAa | aaaauGGCUUUGAAUCUUUGGCCAGUa |
| NM_003940 | USP13 | circRNA | chr3 | 179448419 | 179448439 | + | 1 | 0 | AGO1-4 | 8mer | ucgcccuGAAACUCCCGGUCAa | uggccuuCUCUCA-GGCCAGUa |
| NM_020166 | MCCC1 | circRNA | chr3 | 182733202 | 182733223 | - | 6 | 0 | AGO1-4,AGO2 | 7mer-m8 | ucgcccugaaacUCCCGGUCAa | uccagcaaggaaAUGGCCAGUu |
| NM_017644 | KLHL24 | circRNA | chr3 | 183396996 | 183397017 | + | 4 | 0 | AGO1-4 | 7mer-m8 | ucgcccugaaacucCCGGUCAa | agcugcaaugcccaGGCCAGUg |
| NM_005688 | ABCC5 | circRNA | chr3 | 183719228 | 183719253 | - | 1 | 0 | AGO1-4 | 7mer-m8 | ucgcccugAAACU---C-CCGGUCAa | auuaagcaUUUGACCUGUGGCCAGUc |
| NM_004068 | AP2M1 | circRNA | chr3 | 183901751 | 183901772 | + | 21 | 0 | AGO1,AGO1-4,AGO2 | 8mer | ucgcccugaaacUCCCGGUCAa | agagcucccccaAAGGCCAGUa |
| NM_033259 | CAMK2N2 | circRNA | chr3 | 183977255 | 183977276 | - | 2 | 0 | AGO1-4,AGO2 | 7mer-m8 | ucgcccugaaacUCCCGGUCAa | guuaccaaccacAUGGCCAGUc |
| NM_001009921 | VPS8 | circRNA | chr3 | 184567731 | 184567748 | + | 4 | 0 | AGO1-4,AGO2 | 7mer-m8 | ucGCCCUGAAACUCCCGGUCAa | ugUGGGAUUU----GGCCAGUg |
| NM_015303 | VPS8 | circRNA | chr3 | 184567731 | 184567748 | + | 4 | 0 | AGO1-4,AGO2 | 7mer-m8 | ucGCCCUGAAACUCCCGGUCAa | ugUGGGAUUU----GGCCAGUg |
| NM_001622 | AHSG | circRNA | chr3 | 186338654 | 186338677 | + | 2 | 0 | AGO1-4,AGO2 | 7mer-m8 | ucGCCCUGA-AAC-UCCCGGUCAa | guUGGUGCUGCUGCUGGGCCAGUg |
| NM_001167671 | LPP | circRNA | chr3 | 188599013 | 188599034 | + | 2 | 0 | AGO2 | 8mer | ucgcccugaaacucCCGGUCAa | aucuucuuaguccuGGCCAGUa |
| NM_001167671 | LPP | circRNA | chr3 | 188604147 | 188604168 | + | 3 | 0 | AGO1-4 | 7mer-m8 | ucgccCUGAAACUCCCGGUCAa | uugauGAAUAUUGUGGCCAGUg |
| hsa_circ_0068512 | hsa_circ_0068512 | circRNA | chr3 | 188805027 | 188805048 | + | 1 | 0 | AGO1-4 | 7mer-m8 | ucgcccuGAAACUCCCGGUCAa | ugcauuuCUCUGAUGGCCAGUg |
| NM_012287 | ACAP2 | circRNA | chr3 | 195131356 | 195131378 | - | 2 | 0 | AGO1-4 | 7mer-m8 | ucgcccugaaACU-CCCGGUCAa | uuucaugugcUGAUUGGCCAGUc |
| NM_152699 | SENP5 | circRNA | chr3 | 196612175 | 196612196 | + | 2 | 0 | AGO1-4,AGO2 | 7mer-m8 | ucgcccUGAAACUCCCGGUCAa | agcuaaGCUGGGAAGGCCAGUu |
| NM_152699 | SENP5 | circRNA | chr3 | 196658451 | 196658472 | + | 4 | 0 | AGO1-4,AGO2 | 8mer | ucgcccUGAAACUCCCGGUCAa | aaagcaACCUUGGAGGCCAGUa |
| NM_152699 | SENP5 | circRNA | chr3 | 196658899 | 196658921 | + | 5 | 0 | AGO1-4,AGO2 | 7mer-m8 | ucGCCCUGAAAC-UCCCGGUCAa | guCUGGCUUCUGCAUGGCCAGUg |
| NM_001204387 | DLG1 | circRNA | chr3 | 196792202 | 196792226 | - | 5 | 0 | AGO1,AGO1-4 | 8mer | ucgcccuGAAAC-UC--CCGGUCAa | auaaauuCAUUGAAGCUGGCCAGUa |
| NM_004087 | DLG1 | circRNA | chr3 | 196792202 | 196792226 | - | 5 | 0 | AGO1,AGO1-4 | 8mer | ucgcccuGAAAC-UC--CCGGUCAa | auaaauuCAUUGAAGCUGGCCAGUa |
| NM_003441 | ZNF141 | circRNA | chr4 | 366469 | 366490 | + | 1 | 0 | AGO2 | 7mer-m8 | ucgcccugaaacucCCGGUCAa | cacccaagaccauuGGCCAGUg |
| NM_001127178 | PIGG | circRNA | chr4 | 517584 | 517605 | + | 1 | 0 | AGO1-4 | 7mer-m8 | ucgcCCUGAAACUCCCGGUCAa | aaguGGAUGGUGCUGGCCAGUc |
| NM_005663 | WHSC2 | circRNA | chr4 | 1985089 | 1985113 | - | 1 | 0 | AGO1-4 | 7mer-m8 | ucgcCCUGAAAC--U-CCCGGUCAa | agauGAACUAUGCCACGGGCCAGUg |
| NM_176801 | ADD1 | circRNA | chr4 | 2900986 | 2901004 | + | 2 | 0 | AGO1-4 | 7mer-m8 | ucgcCCUGAAACUCCCGGUCAa | guucGAACUCU---GGCCAGUg |
| NM_003703 | NOP14 | circRNA | chr4 | 2958471 | 2958493 | - | 2 | 0 | AGO1-4 | 7mer-m8 | ucgccCUGA-AACUCCCGGUCAa | gaauuGACUCAUUAUGGCCAGUc |
| NM_002111 | HTT | circRNA | chr4 | 3180131 | 3180152 | + | 1 | 0 | AGO2 | 7mer-m8 | ucgcccugaaACUCCCGGUCAa | ugugauggcaUCAUGGCCAGUg |
| NM_002111 | HTT | circRNA | chr4 | 3242265 | 3242287 | + | 3 | 0 | AGO1,AGO1-4 | 7mer-m8 | ucgcCCUGAAAC-UCCCGGUCAa | ccauGGCCUGUGCUGGGCCAGUg |
| NM_001134647 | AFAP1 | circRNA | chr4 | 7765351 | 7765371 | - | 5 | 0 | AGO1-4 | 7mer-m8 | ucGCCCUGAAACUCCCGGUCAa | gaCUGAGCUCUGA-GGCCAGUg |
| NM_001134647 | AFAP1 | circRNA | chr4 | 7844906 | 7844927 | - | 4 | 0 | AGO1-4,AGO2 | 7mer-m8 | ucgcccugaaacucCCGGUCAa | ggaagaagcgguucGGCCAGUg |
| NM_053042 | ZNF518B | circRNA | chr4 | 10447287 | 10447308 | - | 1 | 0 | AGO1-4 | 7mer-m8 | ucgcccugaaacUCCCGGUCAa | ggcaggugcgaaACGGCCAGUc |
| NM_001166139 | LCORL | circRNA | chr4 | 17963588 | 17963609 | - | 1 | 0 | AGO2 | 7mer-m8 | ucgcccugAAACUCCCGGUCAa | agcuaucaUCUCAGGGCCAGUc |
| NM_001085400 | RELL1 | circRNA | chr4 | 37613253 | 37613277 | - | 2 | 0 | AGO1-4 | 7mer-m8 | ucgcCCUGAAACUC---CCGGUCAa | ggguGAGCUGUGGGGGAGGCCAGUu |
| NM_015173 | TBC1D1 | circRNA | chr4 | 38019975 | 38019985 | + | 2 | 0 | AGO1-4,AGO2 | 7mer-m8 | ucgcccugaaacucCCGGUCAa | #NAME? |
| NM_001204747 | RFC1 | circRNA | chr4 | 39322149 | 39322170 | - | 1 | 1 | AGO2 | 7mer-m8 | ucgcccuGAAACUCCCGGUCAa | gucucuuCUCCCAAGGCCAGUu |
| NM_018177 | N4BP2 | circRNA | chr4 | 40122088 | 40122110 | + | 1 | 0 | AGO2 | 7mer-m8 | ucgcccugaaACU-CCCGGUCAa | auuuuguuggUGACUGGCCAGUu |
| NM_001098634 | RBM47 | circRNA | chr4 | 40428082 | 40428103 | - | 7 | 0 | AGO1,AGO1-4 | 7mer-m8 | ucGCCCUGAAACUCCCGGUCAa | gcCGGGAUCUACGGGGCCAGUu |
| NM_001098634 | RBM47 | circRNA | chr4 | 40440105 | 40440126 | - | 1 | 0 | AGO1-4 | 7mer-m8 | ucgcccugaaacucCCGGUCAa | ucaagaagagcuucGGCCAGUu |
| NM_004181 | UCHL1 | circRNA | chr4 | 41259636 | 41259657 | + | 2 | 0 | AGO1-4 | 7mer-m8 | ucGCCCUGAAACUCCCGGUCAa | ggCUGGGGGUCGCCGGCCAGUg |
| NM_001040402 | DCUN1D4 | circRNA | chr4 | 52779695 | 52779702 | + | 2 | 0 | AGO1-4 | 7mer-m8 | ucgcccugaaacucCCGGUCAa | #NAME? |
| NM_002703 | PPAT | circRNA | chr4 | 57260466 | 57260487 | - | 2 | 0 | AGO1-4,AGO2 | 7mer-m8 | ucgcccUGAAACUCCCGGUCAa | acagacACAAUAGGGGCCAGUu |
| NM_000938 | POLR2B | circRNA | chr4 | 57883211 | 57883231 | + | 1 | 0 | AGO1-4 | 7mer-m8 | ucGCCCUGAAACUCCCGGUCAa | ggCAGGAUCUUG-UGGCCAGUg |
| NM_001134 | AFP | circRNA | chr4 | 74318167 | 74318188 | + | 1 | 0 | AGO1-4 | 7mer-m8 | ucgcccugaaACUCCCGGUCAa | uaaacccuggUGUUGGCCAGUg |
| NM_003715 | USO1 | circRNA | chr4 | 76714850 | 76714873 | + | 4 | 0 | AGO1-4,AGO2 | 7mer-m8 | ucgcccUGAAACUC--CCGGUCAa | uaauucAGUUUCAGCUGGCCAGUu |
| NM_025074 | FRAS1 | circRNA | chr4 | 78978813 | 78978834 | + | 1 | 0 | AGO2 | 7mer-m8 | ucgcccugaaacucCCGGUCAa | uggaagaucccaucGGCCAGUg |
| NM_080685 | PTPN13 | circRNA | chr4 | 87638237 | 87638258 | + | 1 | 0 | AGO2 | 7mer-m8 | ucgcccugaaACUCCCGGUCAa | aaaccuuaagUCAAGGCCAGUc |
| NM_015143 | METAP1 | circRNA | chr4 | 99956551 | 99956568 | + | 1 | 0 | AGO1-4 | 7mer-m8 | ucgcccugaaacUCCCGGUCAa | #NAME? |
| NM_001127208 | TET2 | circRNA | chr4 | 106158264 | 106158285 | + | 8 | 0 | AGO1-4,AGO2 | 7mer-m8 | ucgcCCUGAAACUCCCGGUCAa | aguuGAAAUGUCAGGGCCAGUc |
| NM_021227 | OSTC | circRNA | chr4 | 109576775 | 109576796 | + | 2 | 0 | AGO1-4 | 8mer | ucgcCCUGAAACUCCCGGUCAa | acauGGGCAUCAGAGGCCAGUa |
| NM_006323 | SEC24B | circRNA | chr4 | 110384569 | 110384589 | + | 9 | 0 | AGO1-4,AGO2 | 7mer-m8 | ucgccCUGAAACUCCCGGUCAa | gcuccGACUGUUA-GGCCAGUu |
| NM_003619 | PRSS12 | circRNA | chr4 | 119237406 | 119237429 | - | 1 | 0 | AGO2 | 7mer-m8 | ucgCCCUG-AAACUC-CCGGUCAa | ggaGGUAUAUUACAGAGGCCAGUg |
| NM_176824 | BBS7 | circRNA | chr4 | 122754518 | 122754540 | - | 1 | 0 | AGO1-4 | 7mer-m8 | ucgccCUGAAACU-CCCGGUCAa | acacuGACCCUAACAGGCCAGUu |
| NM_176824 | BBS7 | circRNA | chr4 | 122756416 | 122756437 | - | 1 | 0 | AGO1-4 | 8mer | ucgcccugaAACUCCCGGUCAa | uucgcucaaUUGAAGGCCAGUa |
| NM_015130 | TBC1D9 | circRNA | chr4 | 141543378 | 141543399 | - | 10 | 0 | AGO1-4,AGO2 | 7mer-m8 | ucgcccugaaacucCCGGUCAa | aagccccucaccucGGCCAGUg |
| NM_207123 | GAB1 | circRNA | chr4 | 144361456 | 144361477 | + | 2 | 1 | AGO1-4 | 7mer-m8 | ucgcccugaaaCUCCCGGUCAa | aaccccucccaGAAGGCCAGUu |
| NM_001102653 | OTUD4 | circRNA | chr4 | 146059601 | 146059619 | - | 4 | 0 | AGO1-4 | 7mer-m8 | ucgccCUGAAACUCCCGGUCAa | augcaGAC--UGA-GGCCAGUg |
| NM_001199282 | LRBA | circRNA | chr4 | 151773078 | 151773098 | - | 1 | 0 | AGO1-4 | 7mer-m8 | ucGCCCUGAAACUCCCGGUCAa | ggCUGGA-GUUGAAGGCCAGUc |
| NM_006726 | LRBA | circRNA | chr4 | 151773078 | 151773098 | - | 1 | 0 | AGO1-4 | 7mer-m8 | ucGCCCUGAAACUCCCGGUCAa | ggCUGGA-GUUGAAGGCCAGUc |
| NM_001109977 | FAM160A1 | circRNA | chr4 | 152498663 | 152498683 | + | 2 | 0 | AGO1-4,AGO2 | 7mer-m8 | ucgcccugaaACUCCCGGUCAa | ucccuccagaUGA-GGCCAGUg |
| NM_001130067 | TRIM2 | circRNA | chr4 | 154236985 | 154237006 | + | 1 | 0 | AGO1-4 | 7mer-m8 | ucgcccugaaACUCCCGGUCAa | uauuuuccaaUGAUGGCCAGUu |
| NM_001131007 | KIAA0922 | circRNA | chr4 | 154555358 | 154555379 | + | 2 | 0 | AGO1-4 | 7mer-m8 | ucgcccugaaaCUCCCGGUCAa | gugcaacaugcGAAGGCCAGUu |
| NM_014247 | RAPGEF2 | circRNA | chr4 | 160253669 | 160253692 | + | 7 | 0 | AGO1-4,AGO2 | 7mer-m8 | ucgCCCUGAAA--CUCCCGGUCAa | uugGUGACAUUAAAAAGGCCAGUc |
| NM_001166108 | PALLD | circRNA | chr4 | 169812073 | 169812095 | + | 4 | 0 | AGO1-4,AGO2 | 8mer | ucgcCCUGAA----ACUCCCGGUCAa | #NAME? |
| NM_032783 | CBR4 | circRNA | chr4 | 169923320 | 169923341 | - | 2 | 0 | AGO1-4 | 7mer-m8 | ucgcccugaaacucCCGGUCAa | uaaaaggcaacucuGGCCAGUc |
| NM_005245 | FAT1 | circRNA | chr4 | 187629887 | 187629908 | - | 16 | 0 | AGO1,AGO1-4,AGO2 | 7mer-m8 | ucgcccugaaacuCCCGGUCAa | acaguucaaagccGGGCCAGUc |
| hsa_circ_0001461 | hsa_circ_000713 | circRNA | chr4 | 187629887 | 187629908 | - | 16 | 0 | AGO1,AGO1-4,AGO2 | 7mer-m8 | ucgcccugaaacuCCCGGUCAa | acaguucaaagccGGGCCAGUc |
| NR_027633 | BRD9 | circRNA | chr5 | 891292 | 891313 | - | 1 | 0 | AGO1-4 | 7mer-m8 | ucgcccugaaacucCCGGUCAa | gccgcccccagaucGGCCAGUc |
| NM_006598 | SLC12A7 | circRNA | chr5 | 1083872 | 1083893 | - | 1 | 0 | AGO1-4 | 7mer-m8 | ucgcccugaaacucCCGGUCAa | ggcaucccgggcgcGGCCAGUg |
| NM_015325 | KIAA0947 | circRNA | chr5 | 5463848 | 5463870 | + | 1 | 0 | AGO1-4 | 7mer-m8 | ucgcccugaaacUC-CCGGUCAa | ugagaaguccccAGAGGCCAGUc |
| NM_017755 | NSUN2 | circRNA | chr5 | 6632769 | 6632791 | - | 3 | 0 | AGO1-4 | 7mer-m8 | ucGC-CCUGAAACUCCCGGUCAa | ccCGAGGGCGAGUGGGGCCAGUu |
| NM_012073 | CCT5 | circRNA | chr5 | 10253131 | 10253153 | + | 1 | 0 | AGO2 | 7mer-m8 | ucgcccuGAA-ACUCCCGGUCAa | uaccuucCUUAAGAGGGCCAGUu |
| NM_007118 | TRIO | circRNA | chr5 | 14244580 | 14244600 | + | 1 | 0 | AGO1-4 | 7mer-m8 | ucgcCCUGAAACUCCCGGUCAa | aaaaGCACCUU-AUGGCCAGUu |
| NM_138348 | FAM105B | circRNA | chr5 | 14693064 | 14693085 | + | 2 | 0 | AGO2 | 7mer-m8 | ucgcccugaaacucCCGGUCAa | cccacccaaggacuGGCCAGUg |
| NM_152295 | TARS | circRNA | chr5 | 33462213 | 33462234 | + | 10 | 0 | AGO1,AGO1-4,AGO2 | 7mer-m8 | ucgcccugaaacUCCCGGUCAa | ugaugauaagaaAAGGCCAGUg |
| NM_001145525 | RAI14 | circRNA | chr5 | 34757651 | 34757672 | + | 5 | 0 | AGO1-4,AGO2 | 7mer-m8 | ucGCCCUGAAACUCCCGGUCAa | cuCGGCAAGAAGGGGGCCAGUg |
| NM_015577 | RAI14 | circRNA | chr5 | 34757651 | 34757672 | + | 5 | 0 | AGO1-4,AGO2 | 7mer-m8 | ucGCCCUGAAACUCCCGGUCAa | cuCGGCAAGAAGGGGGCCAGUg |
| NM_023073 | C5orf42 | circRNA | chr5 | 37206480 | 37206503 | - | 1 | 0 | AGO2 | 7mer-m8 | ucgcccUGAAACUC--CCGGUCAa | ugcaacACUCUAAGGUGGCCAGUg |
| NM_181501 | ITGA1 | circRNA | chr5 | 52211322 | 52211343 | + | 1 | 0 | AGO1-4 | 7mer-m8 | ucGCCCUGAAACUCCCGGUCAa | caCUGAAAUUUUUUGGCCAGUc |
| NM_001008397 | GPX8 | circRNA | chr5 | 54456828 | 54456849 | + | 2 | 0 | AGO1-4,AGO2 | 7mer-m8 | ucgcccugaaacucCCGGUCAa | cuaguuguaaacguGGCCAGUg |
| NM_018369 | DEPDC1B | circRNA | chr5 | 59943231 | 59943251 | - | 1 | 0 | AGO1-4 | 7mer-m8 | ucgcccugaaACUCCCGGUCAa | aacaucccagUGA-GGCCAGUu |
| NM_020928 | ZSWIM6 | circRNA | chr5 | 60817128 | 60817149 | + | 2 | 0 | AGO1-4,AGO2 | 7mer-m8 | ucgcccugaaacUCCCGGUCAa | aaguuggagcaaAAGGCCAGUu |
| NM_001253699 | ERBB2IP | circRNA | chr5 | 65307918 | 65307939 | + | 2 | 0 | AGO1-4,AGO2 | 7mer-m8 | ucgcccUGAAACUCCCGGUCAa | uugacaAUUGUGGAGGCCAGUg |
| hsa_circ_0001493 | hsa_circ_001427 | circRNA | chr5 | 65307918 | 65307939 | + | 2 | 0 | AGO1-4,AGO2 | 7mer-m8 | ucgcccUGAAACUCCCGGUCAa | uugacaAUUGUGGAGGCCAGUg |
| hsa_circ_0001494 | hsa_circ_000730 | circRNA | chr5 | 65307918 | 65307939 | + | 2 | 0 | AGO1-4,AGO2 | 7mer-m8 | ucgcccUGAAACUCCCGGUCAa | uugacaAUUGUGGAGGCCAGUg |
| NM_181523 | PIK3R1 | circRNA | chr5 | 67522323 | 67522344 | + | 3 | 0 | AGO1-4,AGO2 | 7mer-m8 | ucgcccugaaacUCCCGGUCAa | auggacagccguAUGGCCAGUc |
| NM_001098728 | GTF2H2C | circRNA | chr5 | 69422122 | 69422140 | + | 1 | 0 | AGO1-4 | 7mer-m8 | ucgcccUGAAACUCCCGGUCAa | agaccuGCUUU---GGCCAGUg |
| NM_017411 | SMN2 | circRNA | chr5 | 69422122 | 69422140 | + | 1 | 0 | AGO1-4 | 7mer-m8 | ucgcccUGAAACUCCCGGUCAa | agaccuGCUUU---GGCCAGUg |
| NR_033417 | GTF2H2B | circRNA | chr5 | 69422122 | 69422140 | + | 1 | 0 | AGO1-4 | 7mer-m8 | ucgcccUGAAACUCCCGGUCAa | agaccuGCUUU---GGCCAGUg |
| TCONS_l2_00023407 | TCONS_l2_00023407 | circRNA | chr5 | 69422122 | 69422140 | + | 1 | 0 | AGO1-4 | 7mer-m8 | ucgcccUGAAACUCCCGGUCAa | agaccuGCUUU---GGCCAGUg |
| NM_001098728 | GTF2H2C | circRNA | chr5 | 69422153 | 69422174 | + | 1 | 0 | AGO1-4 | 7mer-m8 | ucgccCUGAAACUCCCGGUCAa | cagcuGAUUCCCGGGGCCAGUc |
| NM_017411 | SMN2 | circRNA | chr5 | 69422153 | 69422174 | + | 1 | 0 | AGO1-4 | 7mer-m8 | ucgccCUGAAACUCCCGGUCAa | cagcuGAUUCCCGGGGCCAGUc |
| NR_033417 | GTF2H2B | circRNA | chr5 | 69422153 | 69422174 | + | 1 | 0 | AGO1-4 | 7mer-m8 | ucgccCUGAAACUCCCGGUCAa | cagcuGAUUCCCGGGGCCAGUc |
| TCONS_l2_00023407 | TCONS_l2_00023407 | circRNA | chr5 | 69422153 | 69422174 | + | 1 | 0 | AGO1-4 | 7mer-m8 | ucgccCUGAAACUCCCGGUCAa | cagcuGAUUCCCGGGGCCAGUc |
| NR_029426 | SMA4 | circRNA | chr5 | 69771364 | 69771385 | - | 1 | 0 | AGO1-4 | 7mer-m8 | ucgccCUGAAACUCCCGGUCAa | cagcuGAUUCCCGGGGCCAGUc |
| NR_034021 | SMA5 | circRNA | chr5 | 69771364 | 69771385 | - | 1 | 0 | AGO1-4 | 7mer-m8 | ucgccCUGAAACUCCCGGUCAa | cagcuGAUUCCCGGGGCCAGUc |
| NR_029426 | SMA4 | circRNA | chr5 | 69771398 | 69771416 | - | 1 | 0 | AGO1-4 | 7mer-m8 | ucgcccUGAAACUCCCGGUCAa | agaccuGCUUU---GGCCAGUg |
| NR_034021 | SMA5 | circRNA | chr5 | 69771398 | 69771416 | - | 1 | 0 | AGO1-4 | 7mer-m8 | ucgcccUGAAACUCCCGGUCAa | agaccuGCUUU---GGCCAGUg |
| NM_005909 | MAP1B | circRNA | chr5 | 71492148 | 71492168 | + | 6 | 0 | AGO1-4 | 7mer-m8 | ucgCCCUGAAACUCCCGGUCAa | agaGGGAGUCUG-UGGCCAGUg |
| NM_003633 | ENC1 | circRNA | chr5 | 73936141 | 73936161 | - | 1 | 0 | AGO1-4 | 7mer-m8 | ucgcCCUGAAACUCCCGGUCAa | aguuGUGCGUUG-CGGCCAGUc |
| NM_003664 | AP3B1 | circRNA | chr5 | 77536697 | 77536718 | - | 7 | 0 | AGO1-4,AGO2 | 8mer | ucgcccugaaacucCCGGUCAa | guugugaagaauguGGCCAGUa |
| NM_152405 | JMY | circRNA | chr5 | 78617710 | 78617732 | + | 5 | 1 | AGO1,AGO1-4,AGO2 | 7mer-m8 | ucgcccuGAA-ACUCCCGGUCAa | uuacaucCUUGUAAUGGCCAGUu |
| NM_004272 | HOMER1 | circRNA | chr5 | 78746877 | 78746895 | - | 3 | 0 | AGO2 | 7mer-m8 | ucGCCCUGAAACUCCCGGUCAa | cuCAGAAGUUU---GGCCAGUg |
| NM_001131028 | ATG10 | circRNA | chr5 | 81549156 | 81549178 | + | 4 | 0 | AGO1-4,AGO2 | 7mer-m8 | ucGCCCUGAAAC-UCCCGGUCAa | ggCUGAGCAUUGUAGGGCCAGUu |
| NM_004385 | VCAN | circRNA | chr5 | 82833101 | 82833123 | + | 2 | 0 | AGO1-4,AGO2 | 7mer-m8 | ucgcccUGAAACUC-CCGGUCAa | gcagaaGCUAGGCGUGGCCAGUu |
| NM_004385 | VCAN | circRNA | chr5 | 82868305 | 82868326 | + | 2 | 0 | AGO1-4 | 7mer-m8 | ucGCCCUGAAACUCCCGGUCAa | uuUGGCAUGAGAAUGGCCAGUg |
| NM_153354 | TMEM161B | circRNA | chr5 | 87536688 | 87536709 | - | 1 | 0 | AGO2 | 7mer-m8 | ucgcccugaaACUCCCGGUCAa | guuaccauggUGAUGGCCAGUg |
| NM_153354 | TMEM161B | circRNA | chr5 | 87537435 | 87537455 | - | 1 | 0 | AGO2 | 7mer-m8 | ucgCCCUGAAACUCCCGGUCAa | uuaGUGACUUUCA-GGCCAGUu |
| NM_014899 | RHOBTB3 | circRNA | chr5 | 95067724 | 95067744 | + | 6 | 1 | AGO1-4,AGO2 | 7mer-m8 | ucgccCUGAAACUCCCGGUCAa | ucaccGAGUAUCA-GGCCAGUg |
| NM_000919 | PAM | circRNA | chr5 | 102343172 | 102343194 | + | 1 | 0 | AGO1 | 7mer-m8 | ucGCCCUGAAAC-UCCCGGUCAa | agCAGUCCUCUGCCAGGCCAGUu |
| NM_001127510 | APC | circRNA | chr5 | 112154865 | 112154882 | + | 2 | 0 | AGO1-4 | 7mer-m8 | ucGCCCUGAAACUCCCGGUCAa | cuCGGGCC----AGGGCCAGUg |
| NM_152624 | DCP2 | circRNA | chr5 | 112351078 | 112351096 | + | 16 | 0 | AGO1,AGO1-4,AGO2 | 7mer-m8 | ucgcccUGAAACUCCCGGUCAa | gugcauACUUU---GGCCAGUc |
| NM_018700 | TRIM36 | circRNA | chr5 | 114480373 | 114480398 | - | 9 | 0 | AGO1,AGO1-4,AGO2,AGO3 | 7mer-m8 | ucGCCCUGAA-AC-UC--CCGGUCAa | acUGUGAAUUAUGUAGGAGGCCAGUu |
| NM_005509 | DMXL1 | circRNA | chr5 | 118465058 | 118465079 | + | 1 | 0 | AGO1-4 | 7mer-m8 | ucgcccuGAA-ACUCCCGGUCAa | caaccauCUUCUGA-GGCCAGUg |
| NM_020747 | ZNF608 | circRNA | chr5 | 123982829 | 123982852 | - | 10 | 0 | AGO1,AGO1-4,AGO2,AGO3 | 8mer | ucgcccUGAA--ACUCCCGGUCAa | ucagucACUUUAUUAUGGCCAGUa |
| NM_020747 | ZNF608 | circRNA | chr5 | 123983187 | 123983208 | - | 1 | 0 | AGO2 | 7mer-m8 | ucgccCUGAAACUCCCGGUCAa | gguauGAGAUCAAAGGCCAGUu |
| NM_020747 | ZNF608 | circRNA | chr5 | 123983442 | 123983463 | - | 1 | 0 | AGO2 | 8mer | ucgcccugaaacUCCCGGUCAa | caggagagccgcAUGGCCAGUa |
| NR_015360 | FLJ33630 | circRNA | chr5 | 127396900 | 127396920 | - | 12 | 0 | AGO1,AGO1-4,AGO2 | 8mer | ucgcccuGAAACUCCCGGUCAa | cugucuuCUUU-AUGGCCAGUa |
| NM_001999 | FBN2 | circRNA | chr5 | 127670939 | 127670960 | - | 4 | 0 | AGO1-4,AGO2 | 7mer-m8 | ucgcccugaaACUCCCGGUCAa | auaucugugaUGGCGGCCAGUg |
| NM_133372 | FNIP1 | circRNA | chr5 | 130987611 | 130987635 | - | 20 | 0 | AGO1-4,AGO2 | 8mer | ucgcCCUGA-AA-CUC-CCGGUCAa | aaauGGACUGUUCAAGUGGCCAGUa |
| NM_002198 | IRF1 | circRNA | chr5 | 131817354 | 131817378 | - | 5 | 0 | AGO1-4,AGO2 | 7mer-m8 | ucgcCCUGAAACU--C-CCGGUCAa | cuaaGCACUUGAAAUGUGGCCAGUg |
| NM_002198 | IRF1 | circRNA | chr5 | 131819464 | 131819485 | - | 4 | 0 | AGO1-4,AGO2 | 7mer-m8 | ucGCCCUGAAACUCCCGGUCAa | cuCAGGGCCUGGCAGGCCAGUg |
| NM_014423 | AFF4 | circRNA | chr5 | 132211824 | 132211845 | - | 5 | 3 | AGO1-4,AGO2 | 7mer-m8 | ucgcccuGAAACUCCCGGUCAa | gcaccacCUUUAAGGGCCAGUu |
| NM_014423 | AFF4 | circRNA | chr5 | 132211862 | 132211883 | - | 2 | 0 | AGO1-4 | 7mer-m8 | ucgcccugaaacucCCGGUCAa | ucaccaacagaacuGGCCAGUu |
| NM_014423 | AFF4 | circRNA | chr5 | 132213367 | 132213388 | - | 6 | 0 | AGO1-4,AGO2 | 8mer | ucgccCUGAAACUCCCGGUCAa | guucuGGCUCUAGAGGCCAGUa |
| NM_014423 | AFF4 | circRNA | chr5 | 132219173 | 132219193 | - | 8 | 0 | AGO1-4,AGO2 | 8mer | ucgcccugAAACUCCCGGUCAa | auuauucaUCUG-GGGCCAGUa |
| NM_015288 | PHF15 | circRNA | chr5 | 133915371 | 133915393 | + | 10 | 0 | AGO1-4,AGO2 | 7mer-m8 | ucgcccugaaaCU-CCCGGUCAa | auuucauggcaGAUGGGCCAGUc |
| NM_015288 | PHF15 | circRNA | chr5 | 133915768 | 133915793 | + | 2 | 0 | AGO1-4,AGO2 | 7mer-m8 | ucgcCCUGAAA----CUCCCGGUCAa | uggaGGGCUGUAGGGGAGGGCCAGUg |
| NM_015288 | PHF15 | circRNA | chr5 | 133915858 | 133915879 | + | 1 | 0 | AGO2 | 7mer-m8 | ucgcccuGAAACUCCCGGUCAa | cuuccuuCCUUCGGGGCCAGUc |
| NM_015288 | PHF15 | circRNA | chr5 | 133918719 | 133918741 | + | 6 | 0 | AGO1-4,AGO2 | 7mer-m8 | ucgcccuGAAACUC-CCGGUCAa | uggucacCUGGGUGUGGCCAGUg |
| TCONS_00009789 | TCONS_00009789 | circRNA | chr5 | 134453161 | 134453181 | + | 1 | 0 | AGO1-4 | 7mer-m8 | ucgcccUGAAACUCCCGGUCAa | ggucacACAGUUA-GGCCAGUg |
| TCONS_00010097 | TCONS_00010097 | circRNA | chr5 | 134453161 | 134453181 | + | 1 | 0 | AGO1-4 | 7mer-m8 | ucgcccUGAAACUCCCGGUCAa | ggucacACAGUUA-GGCCAGUg |
| TCONS_00010097 | TCONS_00010097 | circRNA | chr5 | 134631203 | 134631221 | + | 1 | 0 | AGO1-4 | 7mer-m8 | ucgcccUGAAACUCCCGGUCAa | guuucaGCUUU---GGCCAGUg |
| NM_000358 | TGFBI | circRNA | chr5 | 135385159 | 135385180 | + | 4 | 0 | AGO1-4 | 8mer | ucgccCUGAAACUCCCGGUCAa | ugcuuGAAGGUAACGGCCAGUa |
| NM_016604 | KDM3B | circRNA | chr5 | 137728860 | 137728869 | + | 2 | 0 | AGO1-4,AGO2 | 7mer-m8 | ucgcccugaaacucCCGGUCAa | #NAME? |
| hsa_circ_0001537 | hsa_circ_001423 | circRNA | chr5 | 137728860 | 137728869 | + | 2 | 0 | AGO1-4,AGO2 | 7mer-m8 | ucgcccugaaacucCCGGUCAa | #NAME? |
| NM_004730 | ETF1 | circRNA | chr5 | 137843313 | 137843332 | - | 1 | 0 | AGO1-4 | 7mer-m8 | ucgcccugAAACUCCCGGUCAa | augaagcaUUU--GGGCCAGUg |
| NM_018834 | MATR3 | circRNA | chr5 | 138643411 | 138643432 | + | 12 | 0 | AGO1,AGO1-4 | 8mer | ucgcccugaaacucCCGGUCAa | ggagaugcagaccaGGCCAGUa |
| NM_199189 | MATR3 | circRNA | chr5 | 138643411 | 138643432 | + | 12 | 0 | AGO1,AGO1-4 | 8mer | ucgcccugaaacucCCGGUCAa | ggagaugcagaccaGGCCAGUa |
| NM_198282 | TMEM173 | circRNA | chr5 | 138855678 | 138855699 | - | 2 | 0 | AGO1-4,AGO2 | 7mer-m8 | ucgcccugaaacuCCCGGUCAa | gucuuguccccuuGGGCCAGUc |
| NM_003883 | HDAC3 | circRNA | chr5 | 141000768 | 141000789 | - | 2 | 0 | AGO2 | 7mer-m8 | ucgcCCUGAAACUCCCGGUCAa | caguGGGCCCUGGAGGCCAGUc |
| NM_002587 | PCDH1 | circRNA | chr5 | 141244557 | 141244578 | - | 1 | 0 | AGO1-4 | 7mer-m8 | ucgcccugaaacucCCGGUCAa | uuccagcugcgccaGGCCAGUg |
| NM_032420 | PCDH1 | circRNA | chr5 | 141244557 | 141244578 | - | 1 | 0 | AGO1-4 | 7mer-m8 | ucgcccugaaacucCCGGUCAa | uuccagcugcgccaGGCCAGUg |
| NM_205836 | FBXO38 | circRNA | chr5 | 147795548 | 147795569 | + | 2 | 0 | AGO1-4,AGO2 | 7mer-m8 | ucGCCCUGAAACUCCCGGUCAa | agCUGGACUCUUUUGGCCAGUu |
| NM_001543 | NDST1 | circRNA | chr5 | 149935656 | 149935676 | + | 2 | 0 | AGO1-4,AGO2 | 7mer-m8 | ucGCCCUGAAACUCCCGGUCAa | agUGGGCCAGCGA-GGCCAGUc |
| NM_001543 | NDST1 | circRNA | chr5 | 149936708 | 149936729 | + | 2 | 1 | AGO1-4 | 7mer-m8 | ucgcccugaaacUCCCGGUCAa | caaaacagcagaGGGGCCAGUu |
| NM_018047 | RBM22 | circRNA | chr5 | 150076238 | 150076259 | - | 4 | 0 | AGO1,AGO1-4,AGO2 | 7mer-m8 | ucgcccugaaacUCCCGGUCAa | cucugauggaacACGGCCAGUu |
| NM_001155 | ANXA6 | circRNA | chr5 | 150506027 | 150506041 | - | 3 | 0 | AGO1-4 | 7mer-m8 | ucgcccugaaACUCCCGGUCAa | #NAME? |
| NM_015315 | LARP1 | circRNA | chr5 | 154188028 | 154188046 | + | 1 | 0 | AGO1-4 | 7mer-m8 | ucgcCCUGAAACUCCCGGUCAa | gcuuGGGCAUU---GGCCAGUc |
| NM_004779 | CNOT8 | circRNA | chr5 | 154242872 | 154242893 | + | 5 | 0 | AGO1-4,AGO2 | 8mer | ucgcccugaaACUCCCGGUCAa | aucugugaagUGUGGGCCAGUa |
| NM_001037738 | NPM1 | circRNA | chr5 | 170818757 | 170818778 | + | 8 | 0 | AGO1,AGO1-4 | 7mer-m8 | ucgcccugaAACUCCCGGUCAa | gaaguguggUUCAGGGCCAGUg |
| NM_002520 | NPM1 | circRNA | chr5 | 170818757 | 170818778 | + | 8 | 0 | AGO1,AGO1-4 | 7mer-m8 | ucgcccugaAACUCCCGGUCAa | gaaguguggUUCAGGGCCAGUg |
| NM_003714 | STC2 | circRNA | chr5 | 172755171 | 172755192 | - | 4 | 0 | AGO1,AGO1-4 | 7mer-m8 | ucgcccugaaacuCCCGGUCAa | gugccgagcggcuGGGCCAGUu |
| NM_020444 | KIAA1191 | circRNA | chr5 | 175773675 | 175773695 | - | 7 | 0 | AGO1-4,AGO2 | 7mer-m8 | ucgCCCUGAAACUCCCGGUCAa | gugGGGCCUCUUA-GGCCAGUg |
| NM_012171 | TSPAN17 | circRNA | chr5 | 176085963 | 176085984 | + | 3 | 0 | AGO1-4,AGO2 | 7mer-m8 | ucgccCUGAAACUCCCGGUCAa | uccuuGGCCCCGCUGGCCAGUg |
| NM_012279 | ZNF346 | circRNA | chr5 | 176492123 | 176492144 | + | 1 | 0 | AGO2 | 7mer-m8 | ucgcccugaaacUCCCGGUCAa | cuuagaccagauAUGGCCAGUu |
| NM_002082 | GRK6 | circRNA | chr5 | 176863142 | 176863164 | + | 2 | 0 | AGO2 | 7mer-m8 | ucGCCCUG-AAACUCCCGGUCAa | uaCGAGAUGAUCGCAGGCCAGUc |
| NM_001142298 | SQSTM1 | circRNA | chr5 | 179263661 | 179263684 | + | 20 | 1 | AGO1,AGO1-4,AGO2,AGO3 | 7mer-m8 | ucGCCCUGAAAC-U-CCCGGUCAa | ugCAGGUCUCUGUACGGGCCAGUu |
| NM_001142298 | SQSTM1 | circRNA | chr5 | 179264032 | 179264053 | + | 14 | 0 | AGO1-4,AGO2 | 7mer-m8 | ucgccCUGAAACUCCCGGUCAa | aggcaGGCUGGGGAGGCCAGUg |
| NM_015455 | CNOT6 | circRNA | chr5 | 180001543 | 180001563 | + | 9 | 0 | AGO1,AGO1-4,AGO2 | 8mer | ucgcccuGAAACUCCCGGUCAa | acugcuuUUUUGA-GGCCAGUa |
| NM_001114618 | MGAT1 | circRNA | chr5 | 180235989 | 180236011 | - | 1 | 0 | AGO1-4 | 7mer-m8 | ucgcccugaaaCU-CCCGGUCAa | agcacaagacaGAUGGGCCAGUu |
| NM_006098 | GNB2L1 | circRNA | chr5 | 180669225 | 180669246 | - | 33 | 0 | AGO1,AGO1-4,AGO2,AGO3 | 7mer-m8 | ucgcccuGAAACUCCCGGUCAa | uuaucucCUCAGAUGGCCAGUu |
| NM_018303 | EXOC2 | circRNA | chr6 | 556484 | 556505 | - | 2 | 0 | AGO1-4,AGO2 | 7mer-m8 | ucgcccugaaaCUC-CCGGUCAa | gugcaagccggGAGAGGCCAGU- |
| NM_030666 | SERPINB1 | circRNA | chr6 | 2838185 | 2838206 | - | 9 | 0 | AGO1-4,AGO2 | 7mer-m8 | ucGCCCUGAAACUCCCGGUCAa | uaUGGUGCUGACCUGGCCAGUg |
| NM_004155 | SERPINB9 | circRNA | chr6 | 2892205 | 2892226 | - | 1 | 0 | AGO1-4 | 7mer-m8 | ucgcccugaaacUCCCGGUCAa | ccaggaggagcaAAGGCCAGUg |
| NM_015482 | SLC22A23 | circRNA | chr6 | 3323807 | 3323828 | - | 2 | 0 | AGO1-4,AGO2 | 7mer-m8 | ucgcccugaaacucCCGGUCAa | ucugcaaaugcacuGGCCAGUc |
| NM_015482 | SLC22A23 | circRNA | chr6 | 3324161 | 3324183 | - | 1 | 0 | AGO1-4 | 7mer-m8 | ucGC-CCUGAAACUCCCGGUCAa | uuCGUGGCCAUGGCGGGCCAGUu |
| NM_006567 | FARS2 | circRNA | chr6 | 5368843 | 5368867 | + | 1 | 0 | AGO1-4 | 7mer-m8 | ucgcccugaaACUC---CCGGUCAa | gucuaccuggUGAGUAAGGCCAGUc |
| NM_001003699 | RREB1 | circRNA | chr6 | 7125819 | 7125841 | + | 1 | 0 | AGO1-4 | 7mer-m8 | ucgcCCUGAAAC-UCCCGGUCAa | uuuuGCAUUGGGUAGGGCCAGUg |
| NM_001003699 | RREB1 | circRNA | chr6 | 7189506 | 7189528 | + | 1 | 0 | AGO2 | 7mer-m8 | ucgcCCUGAAACUC-CCGGUCAa | aaguGCACUGUGUGUGGCCAGUc |
| hsa_circ_0001573 | hsa_circ_001179 | circRNA | chr6 | 7189506 | 7189528 | + | 1 | 0 | AGO2 | 7mer-m8 | ucgcCCUGAAACUC-CCGGUCAa | aaguGCACUGUGUGUGGCCAGUc |
| NM_016495 | TBC1D7 | circRNA | chr6 | 13292634 | 13292652 | - | 1 | 0 | AGO2 | 7mer-m8 | ucGCCCUGAAACUCCCGGUCAa | agCAGGAUU---AUGGCCAGUu |
| NM_016495 | TBC1D7 | circRNA | chr6 | 13304813 | 13304832 | - | 1 | 0 | AGO1-4 | 7mer-m8 | ucgcccuGAAACUCCCGGUCAa | uuuuucuCUUU--GGGCCAGUu |
| NM_000332 | ATXN1 | circRNA | chr6 | 16299784 | 16299805 | - | 3 | 0 | AGO2 | 8mer | ucgccCUGAAACUCCCGGUCAa | uaccaGACUGACAUGGCCAGUa |
| NM_003472 | DEK | circRNA | chr6 | 18236724 | 18236745 | - | 10 | 0 | AGO1-4,AGO2 | 7mer-m8 | ucgccCUGAAACUCCCGGUCAa | auaaaGAAAUUACUGGCCAGUg |
| NM_017640 | LRRC16A | circRNA | chr6 | 25472659 | 25472680 | + | 2 | 0 | AGO1-4 | 7mer-m8 | ucgcccugaaacucCCGGUCAa | uuugcacaaaaacuGGCCAGUg |
| NM_013375 | ABT1 | circRNA | chr6 | 26598252 | 26598273 | + | 1 | 0 | AGO1 | 7mer-m8 | ucgcccugaaACUCCCGGUCAa | gccaagcgcgUGGCGGCCAGUc |
| hsa_circ_0001594 | hsa_circ_000966 | circRNA | chr6 | 27781788 | 27781809 | - | 1 | 0 | AGO1-4 | 7mer-m8 | ucgcccugaaacucCCGGUCAa | gagccacugcgccaGGCCAGUu |
| hsa_circ_0075924 | hsa_circ_0075924 | circRNA | chr6 | 30593963 | 30593984 | + | 5 | 0 | AGO1,AGO1-4,AGO2 | 7mer-m8 | ucGCCCUGAAACUCCCGGUCAa | guUGUGUCUUUUGUGGCCAGUg |
| hsa_circ_0075944 | hsa_circ_0075944 | circRNA | chr6 | 32136678 | 32136699 | - | 5 | 1 | AGO1-4,AGO2 | 7mer-m8 | ucgcccugaaacucCCGGUCAa | cucuaccccacauuGGCCAGUg |
| hsa_circ_0075969 | hsa_circ_0075969 | circRNA | chr6 | 33423227 | 33423247 | + | 1 | 0 | AGO1-4 | 7mer-m8 | ucgcccuGAAACUCCCGGUCAa | cucaucuCCUUG-UGGCCAGUg |
| NM_001188 | BAK1 | circRNA | chr6 | 33541420 | 33541441 | - | 3 | 0 | AGO1-4,AGO2 | 7mer-m8 | ucgcccugaaACUCCCGGUCAa | ggccccugcgUGGGGGCCAGUc |
| NM_001188 | BAK1 | circRNA | chr6 | 33541608 | 33541630 | - | 1 | 0 | AGO1-4 | 7mer-m8 | ucGCCCUGAAAC-UCCCGGUCAa | ggUGUGGUUCUGUUGGGCCAGUu |
| NM_002224 | ITPR3 | circRNA | chr6 | 33632923 | 33632944 | + | 1 | 0 | AGO1 | 7mer-m8 | ucgcccugaaACUCCCGGUCAa | gccagcuccaUGCUGGCCAGUg |
| NM_024294 | C6orf106 | circRNA | chr6 | 34558346 | 34558367 | - | 18 | 0 | AGO1-4,AGO2 | 7mer-m8 | ucgcccugaaacucCCGGUCAa | ggccuuaccccuucGGCCAGUc |
| NM_017754 | UHRF1BP1 | circRNA | chr6 | 34824054 | 34824073 | + | 1 | 0 | AGO1-4 | 7mer-m8 | ucgCCCUGAAACUCCCGGUCAa | augGAGAC-CCGA-GGCCAGUg |
| NM_015695 | BRPF3 | circRNA | chr6 | 36168839 | 36168860 | + | 1 | 0 | AGO1-4 | 7mer-m8 | ucgcccugaaACUCCCGGUCAa | cauacaucccUGAGGGCCAGUg |
| NR_036610 | SRSF3 | circRNA | chr6 | 36567710 | 36567731 | + | 1 | 0 | AGO1 | 7mer-m8 | ucgcccugaaacucCCGGUCAa | acaugacccaggcuGGCCAGUc |
| NM_001012426 | FOXP4 | circRNA | chr6 | 41555608 | 41555629 | + | 1 | 0 | AGO1-4 | 7mer-m8 | ucgcccugaaacuCCCGGUCAa | ugugugaagaccuGGGCCAGUu |
| NM_003131 | SRF | circRNA | chr6 | 43141618 | 43141639 | + | 1 | 0 | AGO1-4 | 7mer-m8 | ucgcccugaaACUCCCGGUCAa | gugcuguugcUGGUGGCCAGUg |
| NM_015388 | YIPF3 | circRNA | chr6 | 43479880 | 43479901 | - | 4 | 0 | AGO1-4,AGO2 | 7mer-m8 | ucgccCUGAAACUCCCGGUCAa | caccuGAAAUUCUUGGCCAGUc |
| NM_018135 | MRPS18A | circRNA | chr6 | 43646298 | 43646319 | - | 3 | 0 | AGO1-4 | 7mer-m8 | ucgcccugaaacucCCGGUCAa | cuccuaaccccucuGGCCAGUg |
| NM_001078175 | SLC29A1 | circRNA | chr6 | 44201532 | 44201554 | + | 4 | 0 | AGO2 | 7mer-m8 | ucgcccUGAA-ACUCCCGGUCAa | aucccuGCUUGUGCAGGCCAGUg |
| NM_001078177 | SLC29A1 | circRNA | chr6 | 44201532 | 44201554 | + | 4 | 0 | AGO2 | 7mer-m8 | ucgcccUGAA-ACUCCCGGUCAa | aucccuGCUUGUGCAGGCCAGUg |
| NM_004955 | SLC29A1 | circRNA | chr6 | 44201532 | 44201554 | + | 4 | 0 | AGO2 | 7mer-m8 | ucgcccUGAA-ACUCCCGGUCAa | aucccuGCUUGUGCAGGCCAGUg |
| NM_014452 | TNFRSF21 | circRNA | chr6 | 47200339 | 47200360 | - | 7 | 0 | AGO1-4,AGO2 | 7mer-m8 | ucgcccugaaacUCCCGGUCAa | uuaacagagaauAUGGCCAGUg |
| NM_015548 | DST | circRNA | chr6 | 56394508 | 56394533 | - | 3 | 0 | AGO1-4,AGO2 | 7mer-m8 | ucgccCUGAAACUC----CCGGUCAa | caccaGGCUGUUAGCAUUGGCCAGUc |
| NM_004282 | BAG2 | circRNA | chr6 | 57048784 | 57048805 | + | 5 | 0 | AGO1-4,AGO2 | 7mer-m8 | ucgcccugaaacuCCCGGUCAa | ugaggugccacauGGGCCAGUu |
| NM_015153 | PHF3 | circRNA | chr6 | 64395236 | 64395258 | + | 2 | 0 | AGO1-4,AGO2 | 7mer-m8 | ucgcccUGAAACU-CCCGGUCAa | agcaaaAUUUUCAUAGGCCAGUc |
| NM_004370 | COL12A1 | circRNA | chr6 | 75839861 | 75839886 | - | 6 | 0 | AGO1-4 | 7mer-m8 | ucGCCCU-GAA--ACU-CCCGGUCAa | ccUGGGAUCAUGCUGAUGGGCCAGUu |
| NM_018247 | TMEM30A | circRNA | chr6 | 75965771 | 75965792 | - | 24 | 0 | AGO1-4,AGO2 | 7mer-m8 | ucgcccugaaACUCCCGGUCAa | ugcaugugcaUCAAGGCCAGUc |
| NM_002526 | NT5E | circRNA | chr6 | 86200327 | 86200349 | + | 2 | 0 | AGO1-4 | 7mer-m8 | ucGC-CCUGAAACUCCCGGUCAa | agCGUGCACCGCUACGGCCAGUc |
| NM_002526 | NT5E | circRNA | chr6 | 86205337 | 86205358 | + | 8 | 0 | AGO1-4,AGO2 | 8mer | ucgcccugaaacucCCGGUCAa | uauuuuuuacacuuGGCCAGUa |
| NM_016021 | UBE2J1 | circRNA | chr6 | 90042810 | 90042830 | - | 7 | 0 | AGO1,AGO1-4,AGO2 | 8mer | ucGCCCUGAAACUCCCGGUCAa | ucCAGGGUGCU-ACGGCCAGUa |
| NM_014611 | MDN1 | circRNA | chr6 | 90426466 | 90426487 | - | 1 | 0 | AGO2 | 7mer-m8 | ucgcccugaaacucCCGGUCAa | aagcuuacgcaguuGGCCAGUg |
| NM_032870 | PNISR | circRNA | chr6 | 99858635 | 99858660 | - | 3 | 0 | AGO1-4,AGO2 | 7mer-m8 | ucgcccUGAAAC----UCCCGGUCAa | ccgauaAUUUUGCAGUGGGGCCAGUg |
| NM_005190 | CCNC | circRNA | chr6 | 99990526 | 99990546 | - | 2 | 0 | AGO1-4,AGO2 | 7mer-m8 | ucgcccugAAACUCCCGGUCAa | caaucaggUUUCA-GGCCAGUu |
| NM_020771 | HACE1 | circRNA | chr6 | 105225112 | 105225133 | - | 1 | 0 | AGO1-4 | 7mer-m8 | ucgcccugaaacucCCGGUCAa | agauauggugcacaGGCCAGUg |
| NM_020771 | HACE1 | circRNA | chr6 | 105228323 | 105228332 | - | 2 | 0 | AGO1-4 | 8mer | ucgcccugaaacucCCGGUCAa | #NAME? |
| NM_020771 | HACE1 | circRNA | chr6 | 105228323 | 105228346 | - | 2 | 0 | AGO1-4 | 8mer | ucgCCCUGAAACUC--CCGGUCAa | uugGUGGUUUUCAGGUGGCCAGUa |
| NM_001105206 | LAMA4 | circRNA | chr6 | 112463463 | 112463485 | - | 2 | 0 | AGO1-4,AGO2 | 7mer-m8 | ucgccCUG-AAACUCCCGGUCAa | uccauGAUGUUUGAUGGCCAGUc |
| NM_001105206 | LAMA4 | circRNA | chr6 | 112512930 | 112512951 | - | 4 | 0 | AGO1-4 | 7mer-m8 | ucgcccugaaacucCCGGUCAa | gugaugaagucacuGGCCAGUg |
| NM_152730 | C6orf170 | circRNA | chr6 | 121513033 | 121513058 | - | 2 | 0 | AGO1-4 | 7mer-m8 | ucgccCUGAAACUC----CCGGUCAa | auguaGGCUGGGAGCCUAGGCCAGUc |
| NM_001135648 | PTPRK | circRNA | chr6 | 128841412 | 128841433 | - | 1 | 0 | AGO2 | 7mer-m8 | ucgcccugaaacUCCCGGUCAa | ugggaucggcccAAGGCCAGUu |
| NM_000426 | LAMA2 | circRNA | chr6 | 129670499 | 129670520 | + | 1 | 0 | AGO1-4 | 8mer | ucGCCCUGAAACUCCCGGUCAa | caCGGGGAUAUGAAGGCCAGUa |
| NM_020464 | NHSL1 | circRNA | chr6 | 138753043 | 138753066 | - | 1 | 0 | AGO1-4 | 7mer-m8 | ucgcccUGAAACU--CCCGGUCAa | gugcccACUGGGAACGGGCCAGUc |
| NM_015439 | CCDC28A | circRNA | chr6 | 139114368 | 139114391 | + | 5 | 0 | AGO1-4,AGO2 | 7mer-m8 | ucgcCCUGAAA--CUCCCGGUCAa | ugguGUGUUUUCCAAUGGCCAGUg |
| hsa_circ_0078031 | hsa_circ_0078031 | circRNA | chr6 | 141958817 | 141958838 | - | 1 | 0 | AGO2 | 7mer-m8 | ucgcccUGAAACUCCCGGUCAa | aaacucAAUUGCUGGGCCAGUc |
| NM_015093 | TAB2 | circRNA | chr6 | 149699378 | 149699401 | + | 5 | 0 | AGO1-4,AGO2 | 7mer-m8 | ucgcccUGAAACUC--CCGGUCAa | uggacaACUUCAAGGUGGCCAGUc |
| NM_001242767 | MTHFD1L | circRNA | chr6 | 151281495 | 151281516 | + | 3 | 0 | AGO1-4 | 7mer-m8 | ucgcCCUGAAACUCCCGGUCAa | ggaaGGAUGGUGGUGGCCAGUg |
| NM_001122740 | ESR1 | circRNA | chr6 | 152163760 | 152163781 | + | 5 | 0 | AGO1-4 | 8mer | ucgcccugaaacucCCGGUCAa | ggcagagaaagauuGGCCAGUa |
| NM_014892 | SCAF8 | circRNA | chr6 | 155066320 | 155066341 | + | 1 | 0 | AGO1-4 | 8mer | ucgcccugaaacucCCGGUCAa | auuugauuaaaauaGGCCAGUa |
| NM_020732 | ARID1B | circRNA | chr6 | 157521975 | 157521996 | + | 1 | 0 | AGO2 | 8mer | ucgCCCUGAAACUCCCGGUCAa | gcaGGCCCAUCCAGGGCCAGUa |
| NM_016224 | SNX9 | circRNA | chr6 | 158296161 | 158296182 | + | 1 | 0 | AGO2 | 7mer-m8 | ucgcccugaaacucCCGGUCAa | gccagcacagcucaGGCCAGUu |
| NM_020823 | TMEM181 | circRNA | chr6 | 159053395 | 159053417 | + | 5 | 0 | AGO1-4,AGO2 | 7mer-m8 | ucgcccuGAAACUC-CCGGUCAa | ccuuccuCGUUGAGUGGCCAGUg |
| NM_000876 | IGF2R | circRNA | chr6 | 160480033 | 160480054 | + | 2 | 0 | AGO1-4 | 7mer-m8 | ucgcccugaaacUCCCGGUCAa | uuggaagccagcAAGGCCAGUc |
| NM_005922 | MAP3K4 | circRNA | chr6 | 161470560 | 161470582 | + | 4 | 0 | AGO2 | 7mer-m8 | ucgccCUGAAACU-CCCGGUCAa | uaucaGACAUUGGCUGGCCAGUg |
| NM_001040000 | MLLT4 | circRNA | chr6 | 168369515 | 168369536 | + | 1 | 0 | AGO2 | 7mer-m8 | ucgcccugaaacuCCCGGUCAa | cagaggcagagcuGGGCCAGUg |
| NM_001130965 | SUN1 | circRNA | chr7 | 930990 | 931011 | + | 1 | 0 | AGO1-4 | 7mer-m8 | ucGCCCUGAAACUCCCGGUCAa | cuCAGGCCUGCCUCGGCCAGUu |
| NM_001040167 | LFNG | circRNA | chr7 | 2567064 | 2567085 | + | 1 | 0 | AGO1-4 | 7mer-m8 | ucgcccUGAAACUCCCGGUCAa | gccucuGCUCCGAGGGCCAGUg |
| NM_002304 | LFNG | circRNA | chr7 | 2567064 | 2567085 | + | 1 | 0 | AGO1-4 | 7mer-m8 | ucgcccUGAAACUCCCGGUCAa | gccucuGCUCCGAGGGCCAGUg |
| NM_152743 | BRAT1 | circRNA | chr7 | 2583531 | 2583552 | - | 1 | 0 | AGO2 | 7mer-m8 | ucGCCCUGAAACUCCCGGUCAa | uuUGUGGCCUCGGCGGCCAGUc |
| NM_001037165 | FOXK1 | circRNA | chr7 | 4795090 | 4795111 | + | 1 | 0 | AGO2 | 7mer-m8 | ucgcccugaaaCUCCCGGUCAa | uuaaaacagaaGAGGGCCAGUg |
| NM_001204456 | RBAK | circRNA | chr7 | 5096926 | 5096934 | + | 1 | 0 | AGO1-4 | 7mer-m8 | ucgcccugaaacuCCCGGUCAa | #NAME? |
| NM_001204513 | RBAK-LOC389458 | circRNA | chr7 | 5096926 | 5096934 | + | 1 | 0 | AGO1-4 | 7mer-m8 | ucgcccugaaacuCCCGGUCAa | #NAME? |
| NM_021163 | RBAK | circRNA | chr7 | 5096926 | 5096934 | + | 1 | 0 | AGO1-4 | 7mer-m8 | ucgcccugaaacuCCCGGUCAa | #NAME? |
| NM_015610 | WIPI2 | circRNA | chr7 | 5270991 | 5271016 | + | 7 | 0 | AGO1-4,AGO2 | 7mer-m8 | ucGCCCUGA--AAC--UCCCGGUCAa | agUGGGUCUGCUUGUCAAGGCCAGUu |
| TCONS_l2_00025633 | TCONS_l2_00025633 | circRNA | chr7 | 5270991 | 5271016 | + | 7 | 0 | AGO1-4,AGO2 | 7mer-m8 | ucGCCCUGA--AAC--UCCCGGUCAa | agUGGGUCUGCUUGUCAAGGCCAGUu |
| TCONS_l2_00025633 | TCONS_l2_00025633 | circRNA | chr7 | 5475541 | 5475561 | + | 1 | 0 | AGO1-4 | 7mer-m8 | ucgcccuGAAACUCCCGGUCAa | aaauuacCUGUCA-GGCCAGUu |
| TCONS_l2_00025633 | TCONS_l2_00025633 | circRNA | chr7 | 5478468 | 5478489 | + | 2 | 0 | AGO1-4 | 7mer-m8 | ucgcccugaaacUCCCGGUCAa | aaaauaaaggccAGGGCCAGUg |
| TCONS_l2_00025633 | TCONS_l2_00025633 | circRNA | chr7 | 5953399 | 5953421 | + | 1 | 0 | AGO1-4 | 7mer-m8 | ucgcccugaaaCUC-CCGGUCAa | gaaguguggcaGAGAGGCCAGUc |
| NM_006303 | AIMP2 | circRNA | chr7 | 6063413 | 6063433 | + | 9 | 1 | AGO1,AGO2 | 7mer-m8 | ucgcccugaAACUCCCGGUCAa | gucuuuuuaUUUA-GGCCAGUu |
| TCONS_l2_00025633 | TCONS_l2_00025633 | circRNA | chr7 | 6063413 | 6063433 | + | 9 | 1 | AGO1,AGO2 | 7mer-m8 | ucgcccugaAACUCCCGGUCAa | gucuuuuuaUUUA-GGCCAGUu |
| TCONS_l2_00025633 | TCONS_l2_00025633 | circRNA | chr7 | 6075105 | 6075126 | + | 2 | 0 | AGO1-4 | 7mer-m8 | ucgcccugaaacucCCGGUCAa | aaugcuacugcacuGGCCAGUc |
| TCONS_l2_00025633 | TCONS_l2_00025633 | circRNA | chr7 | 6103908 | 6103934 | + | 1 | 0 | AGO1-4 | 7mer-m8 | ucGCCCUGAA-----ACUCCCGGUCAa | gaCGGGGUUUCACCAUGUUGGCCAGUc |
| NM_032172 | USP42 | circRNA | chr7 | 6187408 | 6187429 | + | 3 | 0 | AGO1-4,AGO2 | 7mer-m8 | ucgcccugaaacucCCGGUCAa | ccacccauagccccGGCCAGUc |
| TCONS_l2_00025633 | TCONS_l2_00025633 | circRNA | chr7 | 6187408 | 6187429 | + | 3 | 0 | AGO1-4,AGO2 | 7mer-m8 | ucgcccugaaacucCCGGUCAa | ccacccauagccccGGCCAGUc |
| TCONS_l2_00025633 | TCONS_l2_00025633 | circRNA | chr7 | 6389474 | 6389495 | + | 1 | 0 | AGO1-4 | 7mer-m8 | ucgcccugaaacucCCGGUCAa | aaucaaauaaaauaGGCCAGUu |
| TCONS_l2_00025633 | TCONS_l2_00025633 | circRNA | chr7 | 6434879 | 6434900 | + | 1 | 0 | AGO1-4 | 7mer-m8 | ucgcccugaaacucCCGGUCAa | gagccacuacgccuGGCCAGUg |
| TCONS_l2_00025633 | TCONS_l2_00025633 | circRNA | chr7 | 6526672 | 6526694 | + | 1 | 0 | AGO1-4 | 7mer-m8 | ucgcccugaaACU-CCCGGUCAa | uccugccaagUGACCGGCCAGUg |
| NM_016265 | ZNF12 | circRNA | chr7 | 6737434 | 6737442 | - | 1 | 0 | AGO1-4 | 7mer-m8 | ucgcccugaaacuCCCGGUCAa | #NAME? |
| NM_001159767 | BZW2 | circRNA | chr7 | 16737695 | 16737716 | + | 6 | 0 | AGO1-4,AGO2 | 7mer-m8 | ucgcccUGAAACUCCCGGUCAa | uguucaGCUCCCAAGGCCAGUc |
| NM_001002926 | TWISTNB | circRNA | chr7 | 19737380 | 19737401 | - | 9 | 0 | AGO1,AGO1-4,AGO2 | 8mer | ucgcccugaaacucCCGGUCAa | gcucuuaaaagccaGGCCAGUa |
| NM_182762 | MACC1 | circRNA | chr7 | 20180067 | 20180087 | - | 2 | 0 | AGO2 | 7mer-m8 | ucgcccUGAAACUCCCGGUCAa | gcacacACCCUG-UGGCCAGUc |
| NM_003112 | SP4 | circRNA | chr7 | 21469802 | 21469823 | + | 2 | 0 | AGO1-4 | 8mer | ucgcccugaaacucCCGGUCAa | gcucagcagauacuGGCCAGUa |
| NM_003930 | SKAP2 | circRNA | chr7 | 26707381 | 26707402 | - | 1 | 0 | AGO2 | 8mer | ucgcccugaaacucCCGGUCAa | aguuuuuaguccuaGGCCAGUa |
| NM_002047 | GARS | circRNA | chr7 | 30640687 | 30640706 | + | 13 | 0 | AGO1-4,AGO2 | 7mer-m8 | ucgccCUGAAACUCCCGGUCAa | uguauGACUUU--GGGCCAGUu |
| NM_015060 | AVL9 | circRNA | chr7 | 32885521 | 32885542 | + | 3 | 0 | AGO1-4 | 7mer-m8 | ucgcccugaaacucCCGGUCAa | auaguguuaaaauaGGCCAGUg |
| NM_015283 | DPY19L1 | circRNA | chr7 | 35051047 | 35051062 | - | 2 | 0 | AGO1-4 | 7mer-m8 | ucgcccUGAAACUCCCGGUCAa | #NAME? |
| NM_018685 | ANLN | circRNA | chr7 | 36466525 | 36466536 | + | 2 | 0 | AGO1-4 | 7mer-m8 | ucgcccugaaACUCCCGGUCAa | #NAME? |
| NM_006555 | YKT6 | circRNA | chr7 | 44247014 | 44247034 | + | 3 | 0 | AGO1-4 | 8mer | ucGCCCUGAAACU-CCCGGUCAa | gaCAGGA--UAGACUGGCCAGUa |
| NM_015332 | NUDCD3 | circRNA | chr7 | 44507845 | 44507866 | - | 1 | 0 | AGO1-4 | 7mer-m8 | ucgCCCUGAAACUCCCGGUCAa | uugGUGACUCUGAUGGCCAGUu |
| NM_015332 | NUDCD3 | circRNA | chr7 | 44530254 | 44530278 | - | 1 | 0 | AGO1-4 | 7mer-m8 | ucgcCCUGAAAC--UC-CCGGUCAa | cugcGGACGCCGGAAGUGGCCAGUc |
| NM_002541 | OGDH | circRNA | chr7 | 44737209 | 44737232 | + | 2 | 0 | AGO1-4,AGO2 | 7mer-m8 | ucGCCCUGAAAC--UCCCGGUCAa | agCUGGGCUUCGCCAUGGCCAGUc |
| NM_138635 | H2AFV | circRNA | chr7 | 44868155 | 44868176 | - | 11 | 0 | AGO1-4,AGO2 | 7mer-m8 | ucGCCCUGAAACUCCCGGUCAa | uuCAGAUCUUUGUUGGCCAGUc |
| NM_022748 | TNS3 | circRNA | chr7 | 47578005 | 47578029 | - | 1 | 0 | AGO1-4 | 7mer-m8 | ucGCCCUGA---AACUCCCGGUCAa | gcUGGGCCUGGAGUGAAGGCCAGUg |
| NM_001001550 | GRB10 | circRNA | chr7 | 50659001 | 50659022 | - | 10 | 0 | AGO1,AGO1-4 | 7mer-m8 | ucgcccugaaacUCCCGGUCAa | acaagcuucaguAAGGCCAGUc |
| NM_001001550 | GRB10 | circRNA | chr7 | 50686926 | 50686947 | - | 2 | 0 | AGO2 | 7mer-m8 | ucgCCCUGAAACUCCCGGUCAa | gugGAGAGUACCAUGGCCAGUg |
| NM_001001555 | GRB10 | circRNA | chr7 | 50686926 | 50686947 | - | 2 | 0 | AGO2 | 7mer-m8 | ucgCCCUGAAACUCCCGGUCAa | gugGAGAGUACCAUGGCCAGUg |
| NM_001001550 | GRB10 | circRNA | chr7 | 50798697 | 50798718 | - | 1 | 0 | AGO1-4 | 7mer-m8 | ucgcccugaaacucCCGGUCAa | uccaguucucguguGGCCAGUu |
| NM_030796 | VOPP1 | circRNA | chr7 | 55604431 | 55604452 | - | 2 | 0 | AGO1-4 | 7mer-m8 | ucgcccugaaacuCCCGGUCAa | acgcuccgugccuGGGCCAGUg |
| NM_152626 | ZNF92 | circRNA | chr7 | 64778058 | 64778079 | + | 1 | 0 | AGO1-4 | 8mer | ucgcccugaaacucCCGGUCAa | cauaauccugccuaGGCCAGUa |
| NR_027393 | INTS4L1 | circRNA | chr7 | 64778058 | 64778079 | + | 1 | 0 | AGO1-4 | 8mer | ucgcccugaaacucCCGGUCAa | cauaauccugccuaGGCCAGUa |
| NR_033416 | CCT6P3 | circRNA | chr7 | 64778058 | 64778079 | + | 1 | 0 | AGO1-4 | 8mer | ucgcccugaaacucCCGGUCAa | cauaauccugccuaGGCCAGUa |
| NR_027393 | INTS4L1 | circRNA | chr7 | 64861970 | 64861991 | + | 1 | 0 | AGO1-4 | 8mer | ucgcccugaaACUCCCGGUCAa | auuuaauugaUGGUGGCCAGUa |
| NR_033416 | CCT6P3 | circRNA | chr7 | 64861970 | 64861991 | + | 1 | 0 | AGO1-4 | 8mer | ucgcccugaaACUCCCGGUCAa | auuuaauugaUGGUGGCCAGUa |
| hsa_circ_0001711 | hsa_circ_001947 | circRNA | chr7 | 65420046 | 65420067 | + | 1 | 0 | AGO1-4 | 7mer-m8 | ucgcccugaaacuCCCGGUCAa | cagucauaaaacuGGGCCAGUu |
| TCONS_l2_00027391 | TCONS_l2_00027391 | circRNA | chr7 | 66021791 | 66021812 | - | 1 | 0 | AGO1-4 | 7mer-m8 | ucgcCCUGAAACUCCCGGUCAa | cuucGGGCACGGGGGGCCAGUc |
| NM_153033 | KCTD7 | circRNA | chr7 | 66165822 | 66165843 | + | 1 | 0 | AGO1-4 | 7mer-m8 | ucgcccugaaacucCCGGUCAa | gccccagcggcccaGGCCAGUg |
| NM_017994 | C7orf42 | circRNA | chr7 | 66423392 | 66423413 | + | 11 | 0 | AGO1-4,AGO2 | 7mer-m8 | ucGCCCUGAAACUCCCGGUCAa | ugUGGGGUCCUGAGGGCCAGUg |
| NM_172020 | POM121 | circRNA | chr7 | 72361166 | 72361174 | + | 1 | 0 | AGO1-4 | 7mer-m8 | ucgcccugaaacuCCCGGUCAa | #NAME? |
| hsa_circ_0080464 | hsa_circ_0080464 | circRNA | chr7 | 72506213 | 72506234 | + | 1 | 0 | AGO1-4 | 7mer-m8 | ucgcccugaaaCUCCCGGUCAa | gcaauauagggGACGGCCAGUu |
| hsa_circ_0080465 | hsa_circ_0080465 | circRNA | chr7 | 72506213 | 72506234 | + | 1 | 0 | AGO1-4 | 7mer-m8 | ucgcccugaaaCUCCCGGUCAa | gcaauauagggGACGGCCAGUu |
| NM_003388 | CLIP2 | circRNA | chr7 | 72607056 | 72607078 | + | 3 | 0 | AGO1-4 | 7mer-m8 | ucGC-CCUGAAACUCCCGGUCAa | acCGUGGAAGGUAAGGGCCAGUc |
| NM_032999 | GTF2I | circRNA | chr7 | 72607056 | 72607078 | + | 3 | 0 | AGO1-4 | 7mer-m8 | ucGC-CCUGAAACUCCCGGUCAa | acCGUGGAAGGUAAGGGCCAGUc |
| NM_003388 | CLIP2 | circRNA | chr7 | 72607065 | 72607078 | + | 3 | 0 | AGO1-4 | 7mer-m8 | ucgcccugaaACUCCCGGUCAa | #NAME? |
| NM_032999 | GTF2I | circRNA | chr7 | 72607065 | 72607078 | + | 3 | 0 | AGO1-4 | 7mer-m8 | ucgcccugaaACUCCCGGUCAa | #NAME? |
| NM_032408 | BAZ1B | circRNA | chr7 | 72873988 | 72874008 | - | 5 | 0 | AGO1-4,AGO2 | 7mer-m8 | ucgccCUGAAACUCCCGGUCAa | ugauuGCCCUUCA-GGCCAGUg |
| NR_040582 | STAG3L3 | circRNA | chr7 | 72873988 | 72874008 | - | 5 | 0 | AGO1-4,AGO2 | 7mer-m8 | ucgccCUGAAACUCCCGGUCAa | ugauuGCCCUUCA-GGCCAGUg |
| hsa_circ_0001714 | hsa_circ_001192 | circRNA | chr7 | 72873988 | 72874008 | - | 5 | 0 | AGO1-4,AGO2 | 7mer-m8 | ucgccCUGAAACUCCCGGUCAa | ugauuGCCCUUCA-GGCCAGUg |
| NM_003388 | CLIP2 | circRNA | chr7 | 72943629 | 72943650 | + | 1 | 0 | AGO1-4 | 7mer-m8 | ucgcccugaaacucCCGGUCAa | gagccaccaugcccGGCCAGUu |
| NM_032999 | GTF2I | circRNA | chr7 | 72943629 | 72943650 | + | 1 | 0 | AGO1-4 | 7mer-m8 | ucgcccugaaacucCCGGUCAa | gagccaccaugcccGGCCAGUu |
| NM_032408 | BAZ1B | circRNA | chr7 | 72952449 | 72952470 | - | 3 | 0 | AGO1-4,AGO2 | 8mer | ucgcccUGAA-ACUCCCGGUCAa | auguuuGCUUCUCA-GGCCAGUa |
| NR_040582 | STAG3L3 | circRNA | chr7 | 72952449 | 72952470 | - | 3 | 0 | AGO1-4,AGO2 | 8mer | ucgcccUGAA-ACUCCCGGUCAa | auguuuGCUUCUCA-GGCCAGUa |
| NM_032408 | BAZ1B | circRNA | chr7 | 72985130 | 72985153 | - | 2 | 0 | AGO1-4 | 7mer-m8 | ucGCCCUGAAAC--UCCCGGUCAa | ccCAGGUCUUGGCCUUGGCCAGUg |
| NR_040582 | STAG3L3 | circRNA | chr7 | 72985130 | 72985153 | - | 2 | 0 | AGO1-4 | 7mer-m8 | ucGCCCUGAAAC--UCCCGGUCAa | ccCAGGUCUUGGCCUUGGCCAGUg |
| NM_032408 | BAZ1B | circRNA | chr7 | 72985355 | 72985376 | - | 2 | 0 | AGO1-4 | 7mer-m8 | ucgcccugaaacuCCCGGUCAa | agcugcucaaguuGGGCCAGUg |
| NR_040582 | STAG3L3 | circRNA | chr7 | 72985355 | 72985376 | - | 2 | 0 | AGO1-4 | 7mer-m8 | ucgcccugaaacuCCCGGUCAa | agcugcucaaguuGGGCCAGUg |
| NM_032408 | BAZ1B | circRNA | chr7 | 73023506 | 73023526 | - | 1 | 0 | AGO2 | 7mer-m8 | ucGCCCUGAAACUCCCGGUCAa | ccCAGGAAUUUGA-GGCCAGUc |
| NR_040582 | STAG3L3 | circRNA | chr7 | 73023506 | 73023526 | - | 1 | 0 | AGO2 | 7mer-m8 | ucGCCCUGAAACUCCCGGUCAa | ccCAGGAAUUUGA-GGCCAGUc |
| NM_003388 | CLIP2 | circRNA | chr7 | 73111856 | 73111880 | + | 1 | 0 | AGO2 | 7mer-m8 | ucgcCCUGA---AACUCCCGGUCAa | ccauGGAUUCACAUGGGGGCCAGUc |
| NM_032999 | GTF2I | circRNA | chr7 | 73111856 | 73111880 | + | 1 | 0 | AGO2 | 7mer-m8 | ucgcCCUGA---AACUCCCGGUCAa | ccauGGAUUCACAUGGGGGCCAGUc |
| NM_002314 | LIMK1 | circRNA | chr7 | 73500110 | 73500132 | + | 4 | 0 | AGO1-4 | 8mer | ucgcCCU-GAAACUCCCGGUCAa | cagaGGAUCUAUGAUGGCCAGUa |
| NM_003388 | CLIP2 | circRNA | chr7 | 73500110 | 73500132 | + | 4 | 0 | AGO1-4 | 8mer | ucgcCCU-GAAACUCCCGGUCAa | cagaGGAUCUAUGAUGGCCAGUa |
| NM_032999 | GTF2I | circRNA | chr7 | 73500110 | 73500132 | + | 4 | 0 | AGO1-4 | 8mer | ucgcCCU-GAAACUCCCGGUCAa | cagaGGAUCUAUGAUGGCCAGUa |
| NM_003388 | CLIP2 | circRNA | chr7 | 73505081 | 73505102 | + | 4 | 0 | AGO1-4 | 7mer-m8 | ucgcccugaaaCUCCCGGUCAa | cccacuggggaGGUGGCCAGUg |
| NM_032999 | GTF2I | circRNA | chr7 | 73505081 | 73505102 | + | 4 | 0 | AGO1-4 | 7mer-m8 | ucgcccugaaaCUCCCGGUCAa | cccacuggggaGGUGGCCAGUg |
| NM_003388 | CLIP2 | circRNA | chr7 | 73596803 | 73596824 | + | 1 | 0 | AGO1-4 | 7mer-m8 | ucgcccugaaacucCCGGUCAa | gagccauggcguccGGCCAGUc |
| NM_032999 | GTF2I | circRNA | chr7 | 73596803 | 73596824 | + | 1 | 0 | AGO1-4 | 7mer-m8 | ucgcccugaaacucCCGGUCAa | gagccauggcguccGGCCAGUc |
| NM_032408 | BAZ1B | circRNA | chr7 | 73646438 | 73646459 | - | 6 | 0 | AGO1-4,AGO2 | 7mer-m8 | ucgccCUGAAACUCCCGGUCAa | acaauGGCCCCGGUGGCCAGUu |
| NM_181471 | RFC2 | circRNA | chr7 | 73646438 | 73646459 | - | 6 | 0 | AGO1-4,AGO2 | 7mer-m8 | ucgccCUGAAACUCCCGGUCAa | acaauGGCCCCGGUGGCCAGUu |
| NR_040582 | STAG3L3 | circRNA | chr7 | 73646438 | 73646459 | - | 6 | 0 | AGO1-4,AGO2 | 7mer-m8 | ucgccCUGAAACUCCCGGUCAa | acaauGGCCCCGGUGGCCAGUu |
| NM_032408 | BAZ1B | circRNA | chr7 | 73663676 | 73663702 | - | 1 | 0 | AGO1-4 | 7mer-m8 | ucGCCCUGAA-----ACUCCCGGUCAa | gaCGGGGUUUCGCCAUGUUGGCCAGUc |
| NR_040582 | STAG3L3 | circRNA | chr7 | 73663676 | 73663702 | - | 1 | 0 | AGO1-4 | 7mer-m8 | ucGCCCUGAA-----ACUCCCGGUCAa | gaCGGGGUUUCGCCAUGUUGGCCAGUc |
| NM_032408 | BAZ1B | circRNA | chr7 | 73666807 | 73666828 | - | 1 | 0 | AGO1-4 | 8mer | ucgcccugaaacucCCGGUCAa | gguugaaaaauauaGGCCAGUa |
| NM_181471 | RFC2 | circRNA | chr7 | 73666807 | 73666828 | - | 1 | 0 | AGO1-4 | 8mer | ucgcccugaaacucCCGGUCAa | gguugaaaaauauaGGCCAGUa |
| NR_040582 | STAG3L3 | circRNA | chr7 | 73666807 | 73666828 | - | 1 | 0 | AGO1-4 | 8mer | ucgcccugaaacucCCGGUCAa | gguugaaaaauauaGGCCAGUa |
| NM_003388 | CLIP2 | circRNA | chr7 | 74012630 | 74012653 | + | 1 | 0 | AGO1-4 | 7mer-m8 | ucgccCUGAAACUC--CCGGUCAa | ucaaaGGCAGAGAGGAGGCCAGUc |
| NM_032999 | GTF2I | circRNA | chr7 | 74012630 | 74012653 | + | 1 | 0 | AGO1-4 | 7mer-m8 | ucgccCUGAAACUC--CCGGUCAa | ucaaaGGCAGAGAGGAGGCCAGUc |
| NM_003388 | CLIP2 | circRNA | chr7 | 74160741 | 74160763 | + | 3 | 0 | AGO1-4 | 7mer-m8 | ucGC-CCUGAAACUCCCGGUCAa | acCGUGGAAGGUAAGGGCCAGUc |
| NM_032999 | GTF2I | circRNA | chr7 | 74160741 | 74160763 | + | 3 | 0 | AGO1-4 | 7mer-m8 | ucGC-CCUGAAACUCCCGGUCAa | acCGUGGAAGGUAAGGGCCAGUc |
| NR_040582 | STAG3L3 | circRNA | chr7 | 74273336 | 74273357 | - | 1 | 0 | AGO1-4 | 7mer-m8 | ucgcccugaaacucCCGGUCAa | ggucucaccacauuGGCCAGUu |
| NR_040582 | STAG3L3 | circRNA | chr7 | 74277659 | 74277680 | - | 2 | 0 | AGO1-4 | 7mer-m8 | ucgcccUGAAACUCCCGGUCAa | cugccuAUUUUUUAGGCCAGUg |
| NM_001099415 | POM121C | circRNA | chr7 | 75068447 | 75068468 | - | 2 | 0 | AGO1-4 | 7mer-m8 | ucGCCCUGAAACUCCCGGUCAa | uuUGAGCCCCUGGUGGCCAGUg |
| NM_001099415 | POM121C | circRNA | chr7 | 75104446 | 75104454 | - | 1 | 0 | AGO1-4 | 7mer-m8 | ucgcccugaaacuCCCGGUCAa | #NAME? |
| NM_001099415 | POM121C | circRNA | chr7 | 75104446 | 75104467 | - | 1 | 0 | AGO1-4 | 7mer-m8 | ucgcccUGAAACUCCCGGUCAa | guugccAUUACAGGGGCCAGUg |
| NM_001099415 | POM121C | circRNA | chr7 | 75145538 | 75145546 | - | 1 | 0 | AGO1-4 | 7mer-m8 | ucgcccugaaacuCCCGGUCAa | #NAME? |
| NM_005338 | HIP1 | circRNA | chr7 | 75145538 | 75145546 | - | 1 | 0 | AGO1-4 | 7mer-m8 | ucgcccugaaacuCCCGGUCAa | #NAME? |
| NR_028059 | PMS2P3 | circRNA | chr7 | 75145538 | 75145546 | - | 1 | 0 | AGO1-4 | 7mer-m8 | ucgcccugaaacuCCCGGUCAa | #NAME? |
| NM_001099415 | POM121C | circRNA | chr7 | 75145538 | 75145559 | - | 1 | 0 | AGO1-4 | 7mer-m8 | ucgcccUGAAACUCCCGGUCAa | guugccAUUACAGGGGCCAGUg |
| NM_005338 | HIP1 | circRNA | chr7 | 75145538 | 75145559 | - | 1 | 0 | AGO1-4 | 7mer-m8 | ucgcccUGAAACUCCCGGUCAa | guugccAUUACAGGGGCCAGUg |
| NR_028059 | PMS2P3 | circRNA | chr7 | 75145538 | 75145559 | - | 1 | 0 | AGO1-4 | 7mer-m8 | ucgcccUGAAACUCCCGGUCAa | guugccAUUACAGGGGCCAGUg |
| NM_001099415 | POM121C | circRNA | chr7 | 75663391 | 75663415 | - | 2 | 0 | AGO1-4 | 8mer | ucgccCUGAAAC---UCCCGGUCAa | aaguaGGUUAUGCCCAUGGCCAGUa |
| NM_005338 | HIP1 | circRNA | chr7 | 75663391 | 75663415 | - | 2 | 0 | AGO1-4 | 8mer | ucgccCUGAAAC---UCCCGGUCAa | aaguaGGUUAUGCCCAUGGCCAGUa |
| NM_001099415 | POM121C | circRNA | chr7 | 75677196 | 75677217 | - | 1 | 0 | AGO2 | 7mer-m8 | ucgcCCUGAAACUCCCGGUCAa | ggucGGAGUGGGAGGGCCAGUc |
| NM_005338 | HIP1 | circRNA | chr7 | 75677196 | 75677217 | - | 1 | 0 | AGO2 | 7mer-m8 | ucgcCCUGAAACUCCCGGUCAa | ggucGGAGUGGGAGGGCCAGUc |
| NM_016086 | STYXL1 | circRNA | chr7 | 75677196 | 75677217 | - | 1 | 0 | AGO2 | 7mer-m8 | ucgcCCUGAAACUCCCGGUCAa | ggucGGAGUGGGAGGGCCAGUc |
| NM_005918 | MDH2 | circRNA | chr7 | 75695732 | 75695751 | + | 8 | 0 | AGO1,AGO1-4,AGO2 | 7mer-m8 | ucGCCCUGAAACUCCCGGUCAa | gcUGUGAC--GGGUGGCCAGUu |
| NM_001099415 | POM121C | circRNA | chr7 | 75728745 | 75728766 | - | 1 | 0 | AGO2 | 7mer-m8 | ucgcCCUGAAACUCCCGGUCAa | cuguGGAAGGUAAGGGCCAGUc |
| NM_005338 | HIP1 | circRNA | chr7 | 75728745 | 75728766 | - | 1 | 0 | AGO2 | 7mer-m8 | ucgcCCUGAAACUCCCGGUCAa | cuguGGAAGGUAAGGGCCAGUc |
| NM_005751 | AKAP9 | circRNA | chr7 | 91709018 | 91709039 | + | 5 | 0 | AGO1-4 | 7mer-m8 | ucgcccugaaacUCCCGGUCAa | acaugcaagaacAAGGCCAGUu |
| NM_001145306 | CDK6 | circRNA | chr7 | 92238265 | 92238287 | - | 15 | 0 | AGO1,AGO1-4,AGO2,AGO3 | 7mer-m8 | ucgcccugaaacUC-CCGGUCAa | guuuguuauaacAGUGGCCAGUc |
| NM_001259 | CDK6 | circRNA | chr7 | 92238265 | 92238287 | - | 15 | 0 | AGO1,AGO1-4,AGO2,AGO3 | 7mer-m8 | ucgcccugaaacUC-CCGGUCAa | guuuguuauaacAGUGGCCAGUc |
| NM_001099401 | SGCE | circRNA | chr7 | 94248200 | 94248221 | - | 2 | 0 | AGO2 | 7mer-m8 | ucgcccugaaACUCCCGGUCAa | guagaagaaaUGUUGGCCAGUg |
| NM_033017 | TRIM4 | circRNA | chr7 | 99490269 | 99490290 | - | 2 | 0 | AGO1-4 | 7mer-m8 | ucgcccugaAACUCCCGGUCAa | aucagccagUUCUUGGCCAGUg |
| NM_022574 | GIGYF1 | circRNA | chr7 | 100281241 | 100281255 | - | 2 | 0 | AGO1-4 | 7mer-m8 | ucgcccugaaACUCCCGGUCAa | #NAME? |
| NM_004444 | EPHB4 | circRNA | chr7 | 100401154 | 100401175 | - | 1 | 0 | AGO2 | 7mer-m8 | ucgcccugaaacucCCGGUCAa | cagaagaaaaucuuGGCCAGUg |
| NM_004444 | EPHB4 | circRNA | chr7 | 100410475 | 100410500 | - | 2 | 0 | AGO1-4 | 7mer-m8 | ucGCCCUGAA---ACU-CCCGGUCAa | agCGAGGCCUCCAUCAUGGGCCAGUu |
| hsa_circ_0001730 | hsa_circ_000001 | circRNA | chr7 | 100410475 | 100410500 | - | 2 | 0 | AGO1-4 | 7mer-m8 | ucGCCCUGAA---ACU-CCCGGUCAa | agCGAGGCCUCCAUCAUGGGCCAGUu |
| NM_004444 | EPHB4 | circRNA | chr7 | 100419967 | 100419988 | - | 1 | 0 | AGO1-4 | 7mer-m8 | ucgcccugaaaCUCCCGGUCAa | acugccgugagGAUGGCCAGUg |
| NM_000602 | SERPINE1 | circRNA | chr7 | 100775222 | 100775243 | + | 9 | 0 | AGO1-4,AGO2 | 7mer-m8 | ucgcccUGAAACUCCCGGUCAa | cccucuACUUCAACGGCCAGUg |
| NM_001165413 | SERPINE1 | circRNA | chr7 | 100775222 | 100775243 | + | 9 | 0 | AGO1-4,AGO2 | 7mer-m8 | ucgcccUGAAACUCCCGGUCAa | cccucuACUUCAACGGCCAGUg |
| NM_000602 | SERPINE1 | circRNA | chr7 | 100781189 | 100781210 | + | 13 | 0 | AGO1-4 | 7mer-m8 | ucgcccugaaacUCCCGGUCAa | ggugacaggccaAAGGCCAGUg |
| NM_001084 | PLOD3 | circRNA | chr7 | 100852153 | 100852173 | - | 1 | 0 | AGO2 | 7mer-m8 | ucGCCCUGAAACUCCCGGUCAa | gaUGGAGCACU-ACGGCCAGUg |
| NM_001202543 | CUX1 | circRNA | chr7 | 101898038 | 101898057 | + | 1 | 0 | AGO1-4 | 7mer-m8 | ucgCCCUGAAACUCCCGGUCAa | gugGUGAC-CUGA-GGCCAGUg |
| NM_001242713 | LOC100289561 | circRNA | chr7 | 102016270 | 102016278 | + | 1 | 0 | AGO1-4 | 7mer-m8 | ucgcccugaaacuCCCGGUCAa | #NAME? |
| NR_038967 | LOC100630923 | circRNA | chr7 | 102016270 | 102016278 | + | 1 | 0 | AGO1-4 | 7mer-m8 | ucgcccugaaacuCCCGGUCAa | #NAME? |
| NM_001242713 | LOC100289561 | circRNA | chr7 | 102027465 | 102027486 | + | 1 | 0 | AGO1-4 | 7mer-m8 | ucgcccugaaacUCCCGGUCAa | cugcagauagaaAAGGCCAGUc |
| NR_038967 | LOC100630923 | circRNA | chr7 | 102027465 | 102027486 | + | 1 | 0 | AGO1-4 | 7mer-m8 | ucgcccugaaacUCCCGGUCAa | cugcagauagaaAAGGCCAGUc |
| NM_001242713 | LOC100289561 | circRNA | chr7 | 102027534 | 102027556 | + | 1 | 0 | AGO1-4 | 7mer-m8 | ucGCCCUG-AAACUCCCGGUCAa | ucUGGGAUGAAAGGCGGCCAGUg |
| NR_038967 | LOC100630923 | circRNA | chr7 | 102027534 | 102027556 | + | 1 | 0 | AGO1-4 | 7mer-m8 | ucGCCCUG-AAACUCCCGGUCAa | ucUGGGAUGAAAGGCGGCCAGUg |
| NM_001097615 | POLR2J3 | circRNA | chr7 | 102172735 | 102172756 | - | 1 | 0 | AGO1-4 | 7mer-m8 | ucgccCUGAAACUCCCGGUCAa | gucccGGCCAUCAAGGCCAGUc |
| NM_005045 | RELN | circRNA | chr7 | 103276755 | 103276776 | - | 1 | 0 | AGO1-4 | 7mer-m8 | ucgcccugaaacucCCGGUCAa | gguuguggugucuuGGCCAGUg |
| NM_002291 | LAMB1 | circRNA | chr7 | 107580743 | 107580764 | - | 5 | 0 | AGO1-4,AGO2 | 7mer-m8 | ucgcccugaaacuCCCGGUCAa | gugaccaguccacGGGCCAGUg |
| NM_002291 | LAMB1 | circRNA | chr7 | 107592516 | 107592540 | - | 8 | 0 | AGO1-4,AGO2 | 7mer-m8 | ucgcccUGAAAC-UC--CCGGUCAa | cccaauACCUGGCAGCUGGCCAGUg |
| NM_002291 | LAMB1 | circRNA | chr7 | 107599865 | 107599886 | - | 3 | 0 | AGO1-4,AGO2 | 7mer-m8 | ucgcccugaaacucCCGGUCAa | gcaaucccgucacuGGCCAGUg |
| NM_002291 | LAMB1 | circRNA | chr7 | 107600219 | 107600240 | - | 3 | 0 | AGO1-4 | 7mer-m8 | ucgcccugaaaCUCCCGGUCAa | gugaucccaacGGAGGCCAGUg |
| NM_002291 | LAMB1 | circRNA | chr7 | 107605109 | 107605132 | - | 3 | 0 | AGO1-4 | 7mer-m8 | ucgcccUGAAA---CUC---CCGGUCAa | #NAME? |
| NM_002291 | LAMB1 | circRNA | chr7 | 107615771 | 107615794 | - | 3 | 0 | AGO1-4,AGO2 | 7mer-m8 | ucGCCCUGA--AACUCCCGGUCAa | uaCUGGUCUCAUUGCUGGCCAGUg |
| NM_032549 | IMMP2L | circRNA | chr7 | 110303616 | 110303638 | - | 1 | 0 | AGO1-4 | 7mer-m8 | ucgccCUGAAACUC-CCGGUCAa | uugcuGGCAUUGGGAGGCCAGUu |
| NM_001199672 | CALU | circRNA | chr7 | 128409906 | 128409927 | + | 7 | 0 | AGO1-4,AGO2 | 7mer-m8 | ucgCCCUGAAACUCCCGGUCAa | gugGGAAGAGUUAGGGCCAGUg |
| NM_001458 | FLNC | circRNA | chr7 | 128484982 | 128485003 | + | 1 | 0 | AGO1-4 | 7mer-m8 | ucgcccugaaACUCCCGGUCAa | ccgagcaaggUGCGGGCCAGUg |
| NR_034053 | TNPO3 | circRNA | chr7 | 128619166 | 128619177 | - | 3 | 0 | AGO1-4,AGO2 | 7mer-m8 | ucgcccugaaacUCCCGGUCAa | #NAME? |
| NR_034120 | LOC646329 | circRNA | chr7 | 130570060 | 130570081 | - | 1 | 0 | AGO1-4 | 8mer | ucgcccugaaacucCCGGUCAa | acaucauccccacuGGCCAGUa |
| NM_030647 | JHDM1D | circRNA | chr7 | 139788626 | 139788646 | - | 2 | 0 | AGO1,AGO1-4 | 8mer | ucgcccugaAACUCCCGGUCAa | gaauuaaaaUUG-UGGCCAGUa |
| NM_030647 | JHDM1D | circRNA | chr7 | 139791689 | 139791710 | - | 1 | 0 | AGO2 | 7mer-m8 | ucgcccugaaacUCCCGGUCAa | ucuaagcccagaAAGGCCAGUu |
| NM_052853 | ADCK2 | circRNA | chr7 | 140373561 | 140373582 | + | 2 | 0 | AGO1-4,AGO2 | 7mer-m8 | ucgcccugaaacuCCCGGUCAa | ccuacaucaaacuGGGCCAGUg |
| NM_001080392 | KIAA1147 | circRNA | chr7 | 141357367 | 141357389 | - | 3 | 0 | AGO1,AGO2 | 7mer-m8 | ucgcccUGAAA-CUCCCGGUCAa | uacuuuGCUUUCUAAGGCCAGUc |
| NM_001080392 | KIAA1147 | circRNA | chr7 | 141386387 | 141386408 | - | 1 | 0 | AGO1-4 | 7mer-m8 | ucgcccugaaacUCCCGGUCAa | gaguucaagucuAUGGCCAGUg |
| NM_001163474 | ZNF746 | circRNA | chr7 | 149171074 | 149171095 | - | 1 | 0 | AGO1-4 | 7mer-m8 | ucgcccugaaacucCCGGUCAa | uguguuggaagguuGGCCAGUu |
| NM_001142928 | LRRC61 | circRNA | chr7 | 150020594 | 150020619 | + | 1 | 0 | AGO1-4 | 7mer-m8 | ucGCCCUGAA-ACUC---CCGGUCAa | cgCCGGACUUCCCAGCUUGGCCAGUg |
| NM_005542 | INSIG1 | circRNA | chr7 | 155093332 | 155093353 | + | 9 | 0 | AGO1,AGO1-4,AGO2 | 7mer-m8 | ucgcccugaaacuCCCGGUCAa | uuuaagagagaauGGGCCAGUg |
| NM_005494 | DNAJB6 | circRNA | chr7 | 157178264 | 157178285 | + | 1 | 0 | AGO1-4 | 7mer-m8 | ucgcccugaaaCUCCCGGUCAa | aaguugaagaaGAUGGCCAGUu |
| NM_058246 | DNAJB6 | circRNA | chr7 | 157178264 | 157178285 | + | 1 | 0 | AGO1-4 | 7mer-m8 | ucgcccugaaaCUCCCGGUCAa | aaguugaagaaGAUGGCCAGUu |
| NM_018361 | AGPAT5 | circRNA | chr8 | 6618216 | 6618236 | + | 1 | 0 | AGO2 | 7mer-m8 | ucgcccuGAAACUCCCGGUCAa | cucccucCUUU-UGGGCCAGUu |
| NM_004462 | FDFT1 | circRNA | chr8 | 11665798 | 11665819 | + | 1 | 0 | AGO1-4 | 7mer-m8 | ucgcccuGAAACUCCCGGUCAa | uggguucCCUUAGCGGCCAGUg |
| NM_001174159 | SH2D4A | circRNA | chr8 | 19176991 | 19177013 | + | 1 | 0 | AGO1-4 | 7mer-m8 | ucgCCCUG-AAACUCCCGGUCAa | ggaGGGACACCUGGAGGCCAGUu |
| NM_022071 | SH2D4A | circRNA | chr8 | 19176991 | 19177013 | + | 1 | 0 | AGO1-4 | 7mer-m8 | ucgCCCUG-AAACUCCCGGUCAa | ggaGGGACACCUGGAGGCCAGUu |
| NM_022749 | FAM160B2 | circRNA | chr8 | 21960828 | 21960849 | + | 1 | 0 | AGO1-4 | 7mer-m8 | ucgcccugaaacucCCGGUCAa | uggucaggagcccaGGCCAGUg |
| NM_018688 | BIN3 | circRNA | chr8 | 22481753 | 22481774 | - | 1 | 0 | AGO1 | 7mer-m8 | ucgcccugaaacUCCCGGUCAa | aaaggagaagacGGGGCCAGUg |
| NM_003842 | TNFRSF10B | circRNA | chr8 | 22879259 | 22879280 | - | 5 | 0 | AGO1-4,AGO2 | 7mer-m8 | ucgcccuGAAACUCCCGGUCAa | gcaacuuCUACAGGGGCCAGUc |
| NM_152272 | CHMP7 | circRNA | chr8 | 23106743 | 23106763 | + | 1 | 0 | AGO1-4 | 7mer-m8 | ucgccCUGAAACUCCCGGUCAa | agucaGAC-UUCAUGGCCAGUg |
| NM_002318 | LOXL2 | circRNA | chr8 | 23167235 | 23167256 | - | 2 | 0 | AGO1-4,AGO2 | 7mer-m8 | ucgcccugaaacUCCCGGUCAa | agauccacaacaAUGGCCAGUc |
| NM_002318 | LOXL2 | circRNA | chr8 | 23185961 | 23185984 | - | 3 | 0 | AGO1-4 | 7mer-m8 | ucGCCCUGAA--ACUCCCGGUCAa | agUGGGACCUGGUGUCGGCCAGUg |
| NM_024940 | DOCK5 | circRNA | chr8 | 25203020 | 25203042 | + | 2 | 0 | AGO1-4,AGO2 | 7mer-m8 | ucgcccugaaACUC-CCGGUCAa | acagaccagcUCAGCGGCCAGUu |
| NM_152562 | CDCA2 | circRNA | chr8 | 25327507 | 25327528 | + | 1 | 0 | AGO1-4 | 7mer-m8 | ucgcccugaaacUCCCGGUCAa | uuuucuuaguacAUGGCCAGUu |
| NM_002717 | PPP2R2A | circRNA | chr8 | 26218512 | 26218532 | + | 8 | 0 | AGO1,AGO1-4,AGO2,AGO3 | 7mer-m8 | ucgcccugaAACUCCCGGUCAa | aucuaauggUUGA-GGCCAGUc |
| NM_001440 | EXTL3 | circRNA | chr8 | 28575285 | 28575310 | + | 1 | 0 | AGO1-4 | 7mer-m8 | ucgCCCUGAAACU----CCCGGUCAa | acgGGGACCUGGACCUGGGGCCAGUg |
| NM_018310 | BRF2 | circRNA | chr8 | 37702208 | 37702229 | - | 1 | 0 | AGO1-4 | 7mer-m8 | ucgCCCUGAAACUCCCGGUCAa | cagGGGAAGCGGCCGGCCAGUc |
| NM_021623 | PLEKHA2 | circRNA | chr8 | 38830115 | 38830134 | + | 2 | 0 | AGO2 | 7mer-m8 | ucgcccUGAAACUCCCGGUCAa | uucuuaACUU--CUGGCCAGUu |
| NM_001002296 | GOLGA7 | circRNA | chr8 | 41355086 | 41355107 | + | 1 | 0 | AGO1-4 | 7mer-m8 | ucgcCCUGAAACUCCCGGUCAa | cagaGAAGCUCGGCGGCCAGUc |
| NM_006904 | PRKDC | circRNA | chr8 | 48748957 | 48748978 | - | 6 | 0 | AGO1-4,AGO2 | 7mer-m8 | ucgcccugaAACUCCCGGUCAa | ccucucagcUCGCUGGCCAGUg |
| NM_006904 | PRKDC | circRNA | chr8 | 48848317 | 48848338 | - | 9 | 0 | AGO1-4,AGO2 | 7mer-m8 | ucgcccugaaacUCCCGGUCAa | uuuggcagcaaaAGGGCCAGUu |
| NM_005914 | MCM4 | circRNA | chr8 | 48883210 | 48883231 | + | 4 | 0 | AGO1-4 | 8mer | ucgcccugaaacUCCCGGUCAa | accucguccccaGGGGCCAGUa |
| NM_024831 | TGS1 | circRNA | chr8 | 56686038 | 56686059 | + | 2 | 0 | AGO1-4 | 7mer-m8 | ucgcccugaaaCUCCCGGUCAa | cgcgagcggccGCGGGCCAGUu |
| NM_017780 | CHD7 | circRNA | chr8 | 61768578 | 61768599 | + | 3 | 0 | AGO2 | 8mer | ucgcccugaaacUCCCGGUCAa | guugaaaggcaaAUGGCCAGUa |
| NM_006540 | NCOA2 | circRNA | chr8 | 71053519 | 71053542 | - | 2 | 0 | AGO2 | 7mer-m8 | ucGCCCUGAAACU--CCCGGUCAa | acCAGUGCCAUGAACCGGCCAGUc |
| NM_024721 | ZFHX4 | circRNA | chr8 | 77767130 | 77767151 | + | 1 | 0 | AGO1-4 | 7mer-m8 | ucGCCCUGAAACUCCCGGUCAa | cgCGGGAGAGGAAAGGCCAGUu |
| NM_003821 | RIPK2 | circRNA | chr8 | 90782117 | 90782138 | + | 8 | 0 | AGO1-4,AGO2 | 8mer | ucgcccugaaacUCCCGGUCAa | ggacaaaaaucaAGGGCCAGUa |
| NM_015496 | KIAA1429 | circRNA | chr8 | 95539180 | 95539201 | - | 4 | 0 | AGO1-4,AGO2 | 7mer-m8 | ucgccCUGAAACUCCCGGUCAa | aacaaGACUCCCUUGGCCAGUu |
| NM_001135733 | TP53INP1 | circRNA | chr8 | 95941313 | 95941334 | - | 11 | 0 | AGO1-4,AGO2 | 7mer-m8 | ucgcccugaaacucCCGGUCAa | ucgauucacacguaGGCCAGUu |
| NM_001145860 | POP1 | circRNA | chr8 | 99168577 | 99168597 | + | 1 | 0 | AGO2 | 7mer-m8 | ucgccCUGAAACUCCCGGUCAa | ucacaGACCAGGA-GGCCAGUg |
| NM_030780 | SLC25A32 | circRNA | chr8 | 104427146 | 104427163 | - | 1 | 0 | AGO1-4 | 7mer-m8 | ucGCCCUGAAACUCCCGGUCAa | gaCGGGCC----AGGGCCAGUc |
| NM_001100117 | RIMS2 | circRNA | chr8 | 105161269 | 105161287 | + | 1 | 0 | AGO1-4 | 7mer-m8 | ucgcCCUGAAACUCCCGGUCAa | aaguGAACCUU---GGCCAGUg |
| NM_001100117 | RIMS2 | circRNA | chr8 | 105188068 | 105188091 | + | 1 | 0 | AGO1-4 | 7mer-m8 | ucgcccugaAACUC--CCGGUCAa | cacaguuucUAGAGGUGGCCAGUc |
| NM_001100117 | RIMS2 | circRNA | chr8 | 105223928 | 105223954 | + | 1 | 0 | AGO1-4 | 7mer-m8 | ucGCCCUGAA-----ACUCCCGGUCAa | gaUGGGAUUUCAUCAUGUUGGCCAGUc |
| NM_014109 | ATAD2 | circRNA | chr8 | 124408608 | 124408629 | - | 5 | 0 | AGO1-4,AGO2 | 8mer | ucGC-CCUGAAACUCCCGGUCAa | guCGCGCACGCCGA-GGCCAGUa |
| NM_014751 | MTSS1 | circRNA | chr8 | 125580623 | 125580646 | - | 1 | 0 | AGO1-4 | 7mer-m8 | ucgcccuGAAAC--UCCCGGUCAa | uucaucuCUAUGCUGCGGCCAGUg |
| NM_014846 | KIAA0196 | circRNA | chr8 | 126044513 | 126044535 | - | 1 | 0 | AGO1-4 | 7mer-m8 | ucgccCUGAAACU-CCCGGUCAa | uuccuGGCGCUGAUUGGCCAGUu |
| NM_001247996 | ASAP1 | circRNA | chr8 | 131364806 | 131364827 | - | 2 | 0 | AGO1-4,AGO2 | 7mer-m8 | ucgcccugaaACUCCCGGUCAa | caaguccucaUGCAGGCCAGUc |
| NM_016018 | PHF20L1 | circRNA | chr8 | 133817640 | 133817660 | + | 1 | 0 | AGO2 | 7mer-m8 | ucgcccugaAACUCCCGGUCAa | ggauuagaaUUG-GGGCCAGUc |
| NM_001135242 | NDRG1 | circRNA | chr8 | 134249783 | 134249805 | - | 2 | 0 | AGO1-4 | 7mer-m8 | ucgccCUGAAACUC-CCGGUCAa | cugacGUUUCUGGGCGGCCAGUg |
| NM_012154 | EIF2C2 | circRNA | chr8 | 141551270 | 141551292 | - | 7 | 0 | AGO1,AGO2 | 7mer-m8 | ucGCC-CUGAAACUCCCGGUCAa | gaCGGUGUCUCUGAAGGCCAGUu |
| NM_001199649 | PTK2 | circRNA | chr8 | 141900674 | 141900695 | - | 3 | 0 | AGO1-4 | 8mer | ucgcccugaaacuCCCGGUCAa | gagccaaccaccuGGGCCAGUa |
| NM_005607 | PTK2 | circRNA | chr8 | 141900674 | 141900695 | - | 3 | 0 | AGO1-4 | 8mer | ucgcccugaaacuCCCGGUCAa | gagccaaccaccuGGGCCAGUa |
| NM_153831 | PTK2 | circRNA | chr8 | 141900674 | 141900695 | - | 3 | 0 | AGO1-4 | 8mer | ucgcccugaaacuCCCGGUCAa | gagccaaccaccuGGGCCAGUa |
| NM_001080431 | SLC45A4 | circRNA | chr8 | 142225874 | 142225895 | - | 1 | 0 | AGO1-4 | 8mer | ucgcccugaaacuCCCGGUCAa | cguacgcccugcuGGGCCAGUa |
| NM_052924 | RHPN1 | circRNA | chr8 | 144464677 | 144464699 | + | 1 | 0 | AGO1 | 7mer-m8 | ucGCCCU-GAAACUCCCGGUCAa | ccCGGCAUCCACGUGGGCCAGUc |
| NM_198488 | FAM83H | circRNA | chr8 | 144812459 | 144812480 | - | 2 | 0 | AGO1-4,AGO2 | 7mer-m8 | ucGCCC-UGAAACUCCCGGUCAa | cuCGGGUAC-AUACUGGCCAGUg |
| NM_078480 | PUF60 | circRNA | chr8 | 144899883 | 144899904 | - | 7 | 0 | AGO1-4,AGO2 | 8mer | ucgccCUGAAACUCCCGGUCAa | ucuuuGACCUGGGUGGCCAGUa |
| hsa_circ_0085994 | hsa_circ_0085994 | circRNA | chr8 | 145318606 | 145318627 | - | 1 | 0 | AGO1-4 | 7mer-m8 | ucgcccugaaACUCCCGGUCAa | gugucucuccUGGGGGCCAGUg |
| hsa_circ_0085995 | hsa_circ_0085995 | circRNA | chr8 | 145318606 | 145318627 | - | 1 | 0 | AGO1-4 | 7mer-m8 | ucgcccugaaACUCCCGGUCAa | gugucucuccUGGGGGCCAGUg |
| hsa_circ_0085996 | hsa_circ_0085996 | circRNA | chr8 | 145318606 | 145318627 | - | 1 | 0 | AGO1-4 | 7mer-m8 | ucgcccugaaACUCCCGGUCAa | gugucucuccUGGGGGCCAGUg |
| hsa_circ_0085997 | hsa_circ_0085997 | circRNA | chr8 | 145318606 | 145318627 | - | 1 | 0 | AGO1-4 | 7mer-m8 | ucgcccugaaACUCCCGGUCAa | gugucucuccUGGGGGCCAGUg |
| hsa_circ_0085998 | hsa_circ_0085998 | circRNA | chr8 | 145318606 | 145318627 | - | 1 | 0 | AGO1-4 | 7mer-m8 | ucgcccugaaACUCCCGGUCAa | gugucucuccUGGGGGCCAGUg |
| hsa_circ_0085999 | hsa_circ_0085999 | circRNA | chr8 | 145318606 | 145318627 | - | 1 | 0 | AGO1-4 | 7mer-m8 | ucgcccugaaACUCCCGGUCAa | gugucucuccUGGGGGCCAGUg |
| hsa_circ_0086000 | hsa_circ_0086000 | circRNA | chr8 | 145318606 | 145318627 | - | 1 | 0 | AGO1-4 | 7mer-m8 | ucgcccugaaACUCCCGGUCAa | gugucucuccUGGGGGCCAGUg |
| hsa_circ_0086001 | hsa_circ_0086001 | circRNA | chr8 | 145318606 | 145318627 | - | 1 | 0 | AGO1-4 | 7mer-m8 | ucgcccugaaACUCCCGGUCAa | gugucucuccUGGGGGCCAGUg |
| hsa_circ_0086003 | hsa_circ_0086003 | circRNA | chr8 | 145318606 | 145318627 | - | 1 | 0 | AGO1-4 | 7mer-m8 | ucgcccugaaACUCCCGGUCAa | gugucucuccUGGGGGCCAGUg |
| hsa_circ_0086004 | hsa_circ_0086004 | circRNA | chr8 | 145318606 | 145318627 | - | 1 | 0 | AGO1-4 | 7mer-m8 | ucgcccugaaACUCCCGGUCAa | gugucucuccUGGGGGCCAGUg |
| NM_015201 | BOP1 | circRNA | chr8 | 145488731 | 145488755 | - | 2 | 0 | AGO1-4 | 7mer-m8 | ucGCC-CUGA-AACUC-CCGGUCAa | ugCGGCGGCUGCAGAGUGGCCAGUu |
| NM_013291 | CPSF1 | circRNA | chr8 | 145625001 | 145625022 | - | 1 | 0 | AGO1-4 | 7mer-m8 | ucgcccugaaacucCCGGUCAa | cugcaggagcccccGGCCAGUg |
| NM_000973 | RPL8 | circRNA | chr8 | 146017178 | 146017203 | - | 18 | 0 | AGO1,AGO1-4,AGO2 | 7mer-m8 | ucGC-CCUGAAACU---CCCGGUCAa | gcCGAGGGCAUUCACACGGGCCAGUu |
| NM_033301 | RPL8 | circRNA | chr8 | 146017178 | 146017203 | - | 18 | 0 | AGO1,AGO1-4,AGO2 | 7mer-m8 | ucGC-CCUGAAACU---CCCGGUCAa | gcCGAGGGCAUUCACACGGGCCAGUu |
| NM_182905 | WASH1 | circRNA | chr9 | 16802 | 16823 | - | 1 | 0 | AGO1 | 7mer-m8 | ucgcccugaaACUCCCGGUCAa | gcuccugaggUGCUGGCCAGUg |
| NM_153186 | KANK1 | circRNA | chr9 | 617731 | 617752 | + | 1 | 0 | AGO2 | 7mer-m8 | ucgcccugaaacucCCGGUCAa | cauuccgaucagcaGGCCAGUu |
| NM_001042413 | GLIS3 | circRNA | chr9 | 4258444 | 4258465 | - | 1 | 0 | AGO2 | 7mer-m8 | ucgcccuGAAACUCCCGGUCAa | aacacuuCCUAGCAGGCCAGUu |
| NM_004170 | SLC1A1 | circRNA | chr9 | 4586615 | 4586637 | + | 1 | 0 | AGO2 | 8mer | ucgcccUGAAACUC-CCGGUCAa | auucauACUCAAAGAGGCCAGUa |
| NM_017645 | HAUS6 | circRNA | chr9 | 19058813 | 19058831 | - | 1 | 0 | AGO1-4 | 7mer-m8 | ucgccCUGAAACUCCCGGUCAa | uaucaGAUUUU---GGCCAGUc |
| NM_017794 | KIAA1797 | circRNA | chr9 | 20990232 | 20990253 | + | 1 | 0 | AGO1-4 | 8mer | ucgcccugaaacuCCCGGUCAa | gaauggcccggcuGGGCCAGUa |
| NM_001539 | DNAJA1 | circRNA | chr9 | 33038890 | 33038911 | + | 16 | 0 | AGO1,AGO1-4,AGO2 | 7mer-m8 | ucgcccuGAAACUCCCGGUCAa | cagaccuCUUAAUGGGCCAGUg |
| NM_018449 | UBAP2 | circRNA | chr9 | 33948479 | 33948498 | - | 2 | 0 | AGO1-4 | 7mer-m8 | ucgccCUGAAACUCCCGGUCAa | gcuccGAGUUU--GGGCCAGUu |
| hsa_circ_0001846 | hsa_circ_001335 | circRNA | chr9 | 33948479 | 33948498 | - | 2 | 0 | AGO1-4 | 7mer-m8 | ucgccCUGAAACUCCCGGUCAa | gcuccGAGUUU--GGGCCAGUu |
| NM_006289 | TLN1 | circRNA | chr9 | 35697463 | 35697484 | - | 18 | 2 | AGO1-4,AGO2 | 7mer-m8 | ucgcccugaaacucCCGGUCAa | ugccagcagcuuccGGCCAGUc |
| NM_006289 | TLN1 | circRNA | chr9 | 35712924 | 35712946 | - | 10 | 0 | AGO1-4,AGO2 | 7mer-m8 | ucgcCCUGAA-ACUCCCGGUCAa | cauuGUACUUGAUACGGCCAGUg |
| NM_006289 | TLN1 | circRNA | chr9 | 35718859 | 35718883 | - | 10 | 0 | AGO1-4,AGO2 | 7mer-m8 | ucGCCCUGAAACUC---CCGGUCAa | gcUGGGAACGUGGGCCAGGCCAGUg |
| NM_020944 | GBA2 | circRNA | chr9 | 35744352 | 35744373 | - | 1 | 0 | AGO1-4 | 7mer-m8 | ucGCCCUGAAACUC-CCGGUCAa | ccCGUGGC-UGGAGAGGCCAGUu |
| NM_022781 | RNF38 | circRNA | chr9 | 36356331 | 36356352 | - | 6 | 0 | AGO1-4,AGO2 | 7mer-m8 | ucgcccugaaacucCCGGUCAa | auuugccaccaccaGGCCAGUu |
| NM_194328 | RNF38 | circRNA | chr9 | 36356331 | 36356352 | - | 6 | 0 | AGO1-4,AGO2 | 7mer-m8 | ucgcccugaaacucCCGGUCAa | auuugccaccaccaGGCCAGUu |
| NM_194330 | RNF38 | circRNA | chr9 | 36356331 | 36356352 | - | 6 | 0 | AGO1-4,AGO2 | 7mer-m8 | ucgcccugaaacucCCGGUCAa | auuugccaccaccaGGCCAGUu |
| hsa_circ_0001859 | hsa_circ_001783 | circRNA | chr9 | 37087247 | 37087269 | + | 2 | 0 | AGO1-4 | 7mer-m8 | ucgcccugaaAC-UCCCGGUCAa | ucaguuauaaUGCAGGGCCAGUg |
| NM_032226 | ZCCHC7 | circRNA | chr9 | 37164144 | 37164164 | + | 1 | 0 | AGO1-4 | 7mer-m8 | ucgcCCUGAAACUCCCGGUCAa | aagaGGAUCUU-CUGGCCAGUc |
| NM_032226 | ZCCHC7 | circRNA | chr9 | 37197777 | 37197798 | + | 1 | 0 | AGO2 | 7mer-m8 | ucgcccugaaacuCCCGGUCAa | aucugugagaucuGGGCCAGUg |
| NM_012203 | GRHPR | circRNA | chr9 | 37432034 | 37432051 | + | 3 | 0 | AGO1-4 | 7mer-m8 | ucGCCCUGAAACUCCCGGUCAa | acCAGGCCUU----GGCCAGUg |
| NM_016042 | EXOSC3 | circRNA | chr9 | 37782109 | 37782130 | - | 6 | 0 | AGO1,AGO2,AGO3 | 7mer-m8 | ucgcccuGAAACUCCCGGUCAa | gagaucuCAUCUAUGGCCAGUu |
| NM_000692 | ALDH1B1 | circRNA | chr9 | 38396677 | 38396698 | + | 2 | 0 | AGO1-4 | 7mer-m8 | ucgcccugaaacuCCCGGUCAa | uguucuucaacauGGGCCAGUg |
| NM_001099666 | PTAR1 | circRNA | chr9 | 72325975 | 72325996 | - | 1 | 0 | AGO2 | 7mer-m8 | ucgcccugaaacUCCCGGUCAa | gggagaaaaaaaAAGGCCAGUu |
| NM_000689 | ALDH1A1 | circRNA | chr9 | 75531966 | 75531987 | - | 2 | 0 | AGO1-4,AGO2 | 7mer-m8 | ucgcccugaaacUCCCGGUCAa | uauucuaccaccAGGGCCAGUg |
| NM_058179 | PSAT1 | circRNA | chr9 | 80919684 | 80919707 | + | 10 | 0 | AGO1,AGO1-4,AGO2 | 7mer-m8 | ucgcCCUGAAACU-C-CCGGUCAa | gcaaGGAGGUGGGUGCGGCCAGUu |
| NM_007005 | TLE4 | circRNA | chr9 | 82187463 | 82187484 | + | 1 | 0 | AGO1-4 | 7mer-m8 | ucgcccugaaaCUCCCGGUCAa | caaugcccgcgGCGGGCCAGUg |
| NM_007005 | TLE4 | circRNA | chr9 | 82337921 | 82337942 | + | 1 | 0 | AGO2 | 8mer | ucgcccuGAAACUCCCGGUCAa | agaacacCUUAUGGGGCCAGUa |
| NM_014612 | FAM120A | circRNA | chr9 | 96214388 | 96214409 | + | 4 | 0 | AGO1-4 | 7mer-m8 | ucGCCCUGAAACUCCCGGUCAa | acUGGGUCAGCGGCGGCCAGUg |
| NM_014612 | FAM120A | circRNA | chr9 | 96294496 | 96294517 | + | 12 | 0 | AGO1-4,AGO2 | 7mer-m8 | ucgcccUGAAACUCCCGGUCAa | cgcucuGCUCUAUAGGCCAGUu |
| NM_177995 | PTPDC1 | circRNA | chr9 | 96859897 | 96859925 | + | 1 | 0 | AGO2 | 7mer-m8 | ucGCCCUGAA---ACUC----CCGGUCAa | ugCUGGACUUAGCGGAGAACAGGCCAGUg |
| NM_014788 | TRIM14 | circRNA | chr9 | 100847513 | 100847531 | - | 4 | 0 | AGO1-4,AGO2 | 7mer-m8 | ucGCCCUGAAACUCCCGGUCAa | agCUGGUCUCU---GGCCAGUg |
| NR_024532 | ALG2 | circRNA | chr9 | 101980580 | 101980598 | - | 12 | 0 | AGO1-4,AGO2 | 8mer | ucgccCUGAAACUCCCGGUCAa | aguccGACCUU---GGCCAGUa |
| NM_001859 | SLC31A1 | circRNA | chr9 | 116025443 | 116025464 | + | 1 | 0 | AGO1-4 | 7mer-m8 | ucgcCCUGAAACUCCCGGUCAa | aaaaGAAAAUUCAAGGCCAGUu |
| NM_031219 | HDHD3 | circRNA | chr9 | 116137942 | 116137963 | - | 1 | 0 | AGO1-4 | 7mer-m8 | ucgcccugaaacucCCGGUCAa | cagcucuccuccuaGGCCAGUg |
| NM_001633 | AMBP | circRNA | chr9 | 116822483 | 116822504 | - | 2 | 0 | AGO1-4,AGO2 | 7mer-m8 | ucgcccugaaaCUCCCGGUCAa | gcaagucagagGAUGGCCAGUg |
| NM_002160 | TNC | circRNA | chr9 | 117836019 | 117836040 | - | 1 | 0 | AGO1-4 | 7mer-m8 | ucgcccugaaacucCCGGUCAa | acagucuccccacaGGCCAGUg |
| NM_002581 | PAPPA | circRNA | chr9 | 118949583 | 118949604 | + | 3 | 0 | AGO1-4 | 7mer-m8 | ucgcccugaaacucCCGGUCAa | gcagcuaccucccaGGCCAGUg |
| NM_002581 | PAPPA | circRNA | chr9 | 119124935 | 119124956 | + | 4 | 0 | AGO1-4 | 7mer-m8 | ucGCCCUGAAACUCCCGGUCAa | gcCAGGAGAUGCAAGGCCAGUg |
| NM_018249 | CDK5RAP2 | circRNA | chr9 | 123163039 | 123163060 | - | 1 | 0 | AGO1-4 | 7mer-m8 | ucgcccugaaaCUCCCGGUCAa | cacuccccgagGAUGGCCAGUg |
| NM_001009936 | PHF19 | circRNA | chr9 | 123636886 | 123636907 | - | 19 | 0 | AGO1,AGO1-4,AGO2 | 8mer | ucgcccugaaaCUCCCGGUCAa | ccaaacugacgGAGGGCCAGUa |
| NM_015651 | PHF19 | circRNA | chr9 | 123636886 | 123636907 | - | 19 | 0 | AGO1,AGO1-4,AGO2 | 8mer | ucgcccugaaaCUCCCGGUCAa | ccaaacugacgGAGGGCCAGUa |
| NM_001735 | C5 | circRNA | chr9 | 123737060 | 123737081 | - | 1 | 0 | AGO1-4 | 8mer | ucgcccuGAAACUCCCGGUCAa | gaauuucCUUGGGAGGCCAGUa |
| NM_138777 | MRRF | circRNA | chr9 | 125085481 | 125085502 | + | 1 | 0 | AGO1-4 | 7mer-m8 | ucgcccuGAAACUCCCGGUCAa | uccccucCUUACCUGGCCAGUc |
| NM_012197 | RABGAP1 | circRNA | chr9 | 125832652 | 125832675 | + | 3 | 0 | AGO1-4,AGO2 | 7mer-m8 | ucgcCCUGAAAC--UCCCGGUCAa | gauuGGUUAUUGCCAGGGCCAGUc |
| NM_020946 | DENND1A | circRNA | chr9 | 126193750 | 126193771 | - | 1 | 0 | AGO2 | 7mer-m8 | ucgcCCUGAAACUCCCGGUCAa | gguuGUAGUGAGAGGGCCAGUg |
| NM_005833 | RABEPK | circRNA | chr9 | 127996034 | 127996055 | + | 2 | 0 | AGO1-4,AGO2 | 7mer-m8 | ucgcccugAAACUCCCGGUCAa | guguaucaUUCCAUGGCCAGUg |
| NM_001006618 | MAPKAP1 | circRNA | chr9 | 128388830 | 128388854 | - | 1 | 0 | AGO1-4 | 7mer-m8 | ucgcCCUGAAACU--C-CCGGUCAa | uugaGUACUUGAAAUGUGGCCAGUg |
| NM_022833 | FAM129B | circRNA | chr9 | 130269117 | 130269137 | - | 4 | 0 | AGO1,AGO1-4,AGO2 | 7mer-m8 | ucgccCUGAAACUCCCGGUCAa | agacuGAGUUCUA-GGCCAGUg |
| NM_005094 | SLC27A4 | circRNA | chr9 | 131114920 | 131114941 | + | 4 | 0 | AGO1-4,AGO2 | 7mer-m8 | ucgcccugaaacucCCGGUCAa | acaucgugggaaucGGCCAGUg |
| NM_001127244 | LRRC8A | circRNA | chr9 | 131679404 | 131679425 | + | 10 | 0 | AGO1-4,AGO2 | 7mer-m8 | ucgccCUGAAACUCCCGGUCAa | aaaaaGACACUAACGGCCAGUg |
| NM_019594 | LRRC8A | circRNA | chr9 | 131679404 | 131679425 | + | 10 | 0 | AGO1-4,AGO2 | 7mer-m8 | ucgccCUGAAACUCCCGGUCAa | aaaaaGACACUAACGGCCAGUg |
| NM_020145 | SH3GLB2 | circRNA | chr9 | 131783394 | 131783415 | - | 2 | 0 | AGO1-4 | 7mer-m8 | ucgcccugaaacucCCGGUCAa | uacauggcagacgcGGCCAGUg |
| NM_007313 | ABL1 | circRNA | chr9 | 133729451 | 133729470 | + | 2 | 0 | AGO1-4 | 8mer | ucgccCUGAAACUC-CCGGUCAa | #NAME? |
| NM_007313 | ABL1 | circRNA | chr9 | 133729577 | 133729597 | + | 8 | 0 | AGO1-4,AGO2 | 7mer-m8 | ucgccCUGAAACUCCCGGUCAa | uguauGAUUUUG-UGGCCAGUg |
| NM_007313 | ABL1 | circRNA | chr9 | 133738340 | 133738358 | + | 2 | 0 | AGO1-4 | 8mer | ucGCCCUGAAACUCCCGGUCAa | agCUGGGC---GGGGGCCAGUa |
| NM_007313 | ABL1 | circRNA | chr9 | 133762218 | 133762239 | + | 4 | 0 | AGO1,AGO1-4,AGO2 | 7mer-m8 | ucgcccugaaacucCCGGUCAa | ggaggaaagggccuGGCCAGUc |
| NM_006059 | LAMC3 | circRNA | chr9 | 133901810 | 133901831 | + | 1 | 0 | AGO2 | 8mer | ucgcccugaaaCUCCCGGUCAa | acggccggcccGAGGGCCAGUa |
| NM_006059 | LAMC3 | circRNA | chr9 | 133932403 | 133932424 | + | 1 | 0 | AGO1-4 | 7mer-m8 | ucgccCUGAAACUCCCGGUCAa | ccacuGGCUACACGGGCCAGUu |
| NM_005085 | NUP214 | circRNA | chr9 | 134050978 | 134050999 | + | 2 | 0 | AGO1-4 | 7mer-m8 | ucgcccugaaaCUCCCGGUCAa | cucaggcggcaGAUGGCCAGUc |
| NM_005085 | NUP214 | circRNA | chr9 | 134090704 | 134090728 | + | 1 | 0 | AGO2 | 7mer-m8 | ucgcccuGAAACUC---CCGGUCAa | aaaaaccCAUUCAGCUCGGCCAGUg |
| NM_013318 | PRRC2B | circRNA | chr9 | 134278264 | 134278287 | + | 1 | 0 | AGO1-4 | 7mer-m8 | ucgcCCUGA-AAC-UCCCGGUCAa | gcaaGCAUUCUUGUAUGGCCAGUg |
| NM_013318 | PRRC2B | circRNA | chr9 | 134358809 | 134358830 | + | 2 | 0 | AGO1-4 | 8mer | ucgcccugaaacucCCGGUCAa | cuucaggaugccuuGGCCAGUa |
| NM_198679 | RAPGEF1 | circRNA | chr9 | 134591465 | 134591487 | - | 2 | 0 | AGO1-4 | 7mer-m8 | ucgcccugaaacUC-CCGGUCAa | ggauccagcagcAGUGGCCAGUg |
| NM_198679 | RAPGEF1 | circRNA | chr9 | 134608938 | 134608959 | - | 1 | 0 | AGO1-4 | 7mer-m8 | ucgcccugaaacucCCGGUCAa | ucugucauccagcaGGCCAGUc |
| NM_003172 | SURF1 | circRNA | chr9 | 136220759 | 136220778 | - | 3 | 0 | AGO1-4,AGO2 | 7mer-m8 | ucGCCCUGAAACUCCCGGUCAa | auCUGGAGUAU-A-GGCCAGUg |
| NM_033161 | SURF4 | circRNA | chr9 | 136237332 | 136237353 | - | 3 | 0 | AGO1-4 | 7mer-m8 | ucgcccugaaACUCCCGGUCAa | gcucauuaaaUGGUGGCCAGUu |
| NM_020385 | REXO4 | circRNA | chr9 | 136279906 | 136279927 | - | 1 | 0 | AGO2 | 7mer-m8 | ucgcccugaaacUCCCGGUCAa | guaccucgcaccAAGGCCAGUg |
| NM_001134398 | VAV2 | circRNA | chr9 | 136793979 | 136794000 | - | 3 | 0 | AGO1-4 | 7mer-m8 | ucgcccugaaacucCCGGUCAa | ugccagccucacccGGCCAGUg |
| NM_017588 | WDR5 | circRNA | chr9 | 137017113 | 137017134 | + | 1 | 0 | AGO1-4 | 7mer-m8 | ucgcccugaaacucCCGGUCAa | gggacaccgccucaGGCCAGUg |
| NM_017588 | WDR5 | circRNA | chr9 | 137024128 | 137024148 | + | 4 | 0 | AGO1-4,AGO2 | 7mer-m8 | ucgcCCUGAAACUCCCGGUCAa | augaGGGC-UGCAGGGCCAGUg |
| NM_015447 | CAMSAP1 | circRNA | chr9 | 138773559 | 138773580 | - | 2 | 0 | AGO1-4,AGO2 | 7mer-m8 | ucGCCCUGAAACUCCCGGUCAa | auCGAGAAGGUGGUGGCCAGUg |
| hsa_circ_0001900 | hsa_circ_000004 | circRNA | chr9 | 138773559 | 138773580 | - | 2 | 0 | AGO1-4,AGO2 | 7mer-m8 | ucGCCCUGAAACUCCCGGUCAa | auCGAGAAGGUGGUGGCCAGUg |
| NM_014866 | SEC16A | circRNA | chr9 | 139348744 | 139348762 | - | 1 | 0 | AGO1-4 | 7mer-m8 | ucGCCCUGAAACUCCCGGUCAa | gaCGGCCCAUU---GGCCAGUc |
| NM_017617 | NOTCH1 | circRNA | chr9 | 139399765 | 139399786 | - | 2 | 0 | AGO1-4 | 7mer-m8 | ucgcccugaaaCUCCCGGUCAa | gccagcgugcgGAAGGCCAGUg |
| NM_001606 | ABCA2 | circRNA | chr9 | 139915203 | 139915226 | - | 1 | 0 | AGO2 | 7mer-m8 | ucGCC-CUGAAAC-UCCCGGUCAa | ccCGGACACGCUGCAGGGCCAGUg |
| NM_017723 | C9orf167 | circRNA | chr9 | 140175125 | 140175151 | + | 3 | 0 | AGO1-4,AGO2 | 7mer-m8 | ucGCCCUGAAACUC-----CCGGUCAa | cgUGGGACCCUUAGCCCGUGGCCAGUc |
| NM_152285 | ARRDC1 | circRNA | chr9 | 140508637 | 140508658 | + | 3 | 0 | AGO1-4,AGO2 | 7mer-m8 | ucGCCCUGAAACUCCCGGUCAa | acCAGCCCUGUGGUGGCCAGUc |
| NM_152285 | ARRDC1 | circRNA | chr9 | 140509372 | 140509394 | + | 5 | 0 | AGO1,AGO1-4 | 7mer-m8 | ucgcCCUGA-AACUCCCGGUCAa | cagaGGGCUCCGGGGGGCCAGUg |
| NM_015155 | LARP4B | circRNA | chr10 | 858421 | 858442 | - | 3 | 0 | AGO1-4,AGO2 | 7mer-m8 | ucgcccugaaACUCCCGGUCAa | cgcagaugaaUGAGGGCCAGUg |
| NM_001242339 | PFKP | circRNA | chr10 | 3150010 | 3150032 | + | 1 | 0 | AGO1-4 | 7mer-m8 | ucGCCCUGAA--ACUCCCGGUCAa | ccUGGGACUUGCUCA-GGCCAGUg |
| NM_017782 | FAM208B | circRNA | chr10 | 5790440 | 5790461 | + | 1 | 0 | AGO1-4 | 7mer-m8 | ucgcccugaaacUCCCGGUCAa | ccagcaggcagaAUGGCCAGUu |
| NM_031923 | TAF3 | circRNA | chr10 | 8051222 | 8051243 | + | 1 | 0 | AGO1-4 | 7mer-m8 | ucGCCCUGAAACUCCCGGUCAa | ccCGCCGCCUCCGGGGCCAGUg |
| NM_015542 | UPF2 | circRNA | chr10 | 11997286 | 11997307 | - | 8 | 0 | AGO1-4,AGO2 | 8mer | ucGCCCUGAAACUCCCGGUCAa | uuCUGGACACAUGUGGCCAGUa |
| NM_080599 | UPF2 | circRNA | chr10 | 11997286 | 11997307 | - | 8 | 0 | AGO1-4,AGO2 | 8mer | ucGCCCUGAAACUCCCGGUCAa | uuCUGGACACAUGUGGCCAGUa |
| hsa_circ_0017914 | hsa_circ_0017914 | circRNA | chr10 | 19787931 | 19787952 | + | 1 | 0 | AGO1-4 | 7mer-m8 | ucgcccugaaacUCCCGGUCAa | ccuguugugacaAUGGCCAGUc |
| hsa_circ_0017915 | hsa_circ_0017915 | circRNA | chr10 | 19787931 | 19787952 | + | 1 | 0 | AGO1-4 | 7mer-m8 | ucgcccugaaacUCCCGGUCAa | ccuguugugacaAUGGCCAGUc |
| NM_014915 | ANKRD26 | circRNA | chr10 | 27301946 | 27301966 | - | 3 | 0 | AGO1-4,AGO2 | 7mer-m8 | ucgcccUGAAACUCCCGGUCAa | acucucACUACCA-GGCCAGUc |
| NM_021738 | SVIL | circRNA | chr10 | 29935382 | 29935403 | - | 2 | 0 | AGO1-4,AGO2 | 8mer | ucgcccUGAAACUCCCGGUCAa | uagcuuACCAUACGGGCCAGUa |
| NM_145012 | CCNY | circRNA | chr10 | 35858482 | 35858499 | + | 2 | 0 | AGO1-4 | 7mer-m8 | ucGCCCUGAAACUCCCGGUCAa | ccCAGGGCU----GGGCCAGUg |
| NM_145012 | CCNY | circRNA | chr10 | 35859555 | 35859577 | + | 2 | 0 | AGO1-4 | 7mer-m8 | ucgccCUGAAACUC-CCGGUCAa | uucaaGUCUCUGGGAGGCCAGUg |
| NM_015262 | FAM21C | circRNA | chr10 | 46287826 | 46287847 | + | 1 | 0 | AGO1-4 | 8mer | ucgccCUGAAACUCCCGGUCAa | ugaauGCCUUUGGAGGCCAGUa |
| TCONS_00018502 | TCONS_00018502 | circRNA | chr10 | 47600685 | 47600706 | - | 1 | 0 | AGO1-4 | 8mer | ucgcccugaaACUCCCGGUCAa | caguucaaccUGCAGGCCAGUa |
| TCONS_l2_00004179 | TCONS_l2_00004179 | circRNA | chr10 | 47600685 | 47600706 | - | 1 | 0 | AGO1-4 | 8mer | ucgcccugaaACUCCCGGUCAa | caguucaaccUGCAGGCCAGUa |
| NM_001145260 | NCOA4 | circRNA | chr10 | 51383156 | 51383177 | + | 1 | 0 | AGO1-4 | 7mer-m8 | ucgcccugaaacUCCCGGUCAa | caaccaucaaccAUGGCCAGUu |
| hsa_circ_0002224 | hsa_circ_0002224 | circRNA | chr10 | 51383156 | 51383177 | + | 1 | 0 | AGO1-4 | 7mer-m8 | ucgcccugaaacUCCCGGUCAa | caaccaucaaccAUGGCCAGUu |
| hsa_circ_0006820 | hsa_circ_0006820 | circRNA | chr10 | 51383156 | 51383177 | + | 1 | 0 | AGO1-4 | 7mer-m8 | ucgcccugaaacUCCCGGUCAa | caaccaucaaccAUGGCCAGUu |
| hsa_circ_0018356 | hsa_circ_0018356 | circRNA | chr10 | 51383156 | 51383177 | + | 1 | 0 | AGO1-4 | 7mer-m8 | ucgcccugaaacUCCCGGUCAa | caaccaucaaccAUGGCCAGUu |
| hsa_circ_0018357 | hsa_circ_0018357 | circRNA | chr10 | 51383156 | 51383177 | + | 1 | 0 | AGO1-4 | 7mer-m8 | ucgcccugaaacUCCCGGUCAa | caaccaucaaccAUGGCCAGUu |
| hsa_circ_0018359 | hsa_circ_0018359 | circRNA | chr10 | 51383156 | 51383177 | + | 1 | 0 | AGO1-4 | 7mer-m8 | ucgcccugaaacUCCCGGUCAa | caaccaucaaccAUGGCCAGUu |
| NR_029388 | LOC728407 | circRNA | chr10 | 51611573 | 51611594 | - | 1 | 0 | AGO1-4 | 7mer-m8 | ucgcccugaaacUCCCGGUCAa | caaccaucaaccAUGGCCAGUu |
| NM_022079 | HERC4 | circRNA | chr10 | 69805328 | 69805349 | - | 3 | 0 | AGO1-4 | 8mer | ucgcccugaaacUCCCGGUCAa | gcuacugguaguAGGGCCAGUa |
| NM_138357 | MCU | circRNA | chr10 | 74566635 | 74566656 | + | 1 | 0 | AGO1-4 | 7mer-m8 | ucgcccugaaacucCCGGUCAa | ccagaagcaagcuuGGCCAGUu |
| NM_001135752 | ECD | circRNA | chr10 | 74908069 | 74908088 | - | 2 | 0 | AGO1-4 | 7mer-m8 | ucgcccUGAAACUCCCGGUCAa | cucaccACUCU--GGGCCAGUu |
| NM_004922 | SEC24C | circRNA | chr10 | 75510929 | 75510950 | + | 1 | 0 | AGO2 | 7mer-m8 | ucgcccugaaacUCCCGGUCAa | gccaggcugcauAUGGCCAGUu |
| NM_015037 | KIAA0913 | circRNA | chr10 | 75554040 | 75554061 | + | 1 | 3 | AGO1 | 7mer-m8 | ucgcccugaaACUCCCGGUCAa | uugccucucaUGCUGGCCAGUu |
| hsa_circ_0000249 | hsa_circ_001838 | circRNA | chr10 | 76259880 | 76259905 | + | 1 | 0 | AGO1-4 | 7mer-m8 | ucGCCCUGA-AACUC---CCGGUCAa | agCAGAACUGUUGUGUCAGGCCAGUg |
| NM_004747 | DLG5 | circRNA | chr10 | 79632177 | 79632197 | - | 1 | 0 | AGO1-4 | 7mer-m8 | ucgcCCUGAAACUCCCGGUCAa | guuaGGCCCCAGA-GGCCAGUc |
| NM_020338 | ZMIZ1 | circRNA | chr10 | 81056268 | 81056289 | + | 7 | 0 | AGO1,AGO1-4,AGO2 | 8mer | ucgcccugaaacucCCGGUCAa | uccccacccagccaGGCCAGUa |
| NM_005729 | PPIF | circRNA | chr10 | 81113573 | 81113591 | + | 9 | 1 | AGO1-4,AGO2 | 7mer-m8 | ucgccCUGAAACUCCCGGUCAa | ucacaGACUGU---GGCCAGUu |
| NM_030927 | TSPAN14 | circRNA | chr10 | 82232573 | 82232594 | + | 1 | 0 | AGO1-4 | 7mer-m8 | ucgcccugaaacucCCGGUCAa | auuguuccagauuuGGCCAGUg |
| NM_030927 | TSPAN14 | circRNA | chr10 | 82233934 | 82233956 | + | 1 | 0 | AGO2 | 7mer-m8 | ucgcccugaaACUC-CCGGUCAa | ugugaucaggUCAGAGGCCAGUu |
| NM_018999 | FAM190B | circRNA | chr10 | 86274479 | 86274500 | + | 6 | 0 | AGO1-4,AGO2 | 7mer-m8 | ucgcccugaaacucCCGGUCAa | guaagcagaguucuGGCCAGUg |
| NM_003972 | BTAF1 | circRNA | chr10 | 93742412 | 93742433 | + | 3 | 0 | AGO1-4,AGO2 | 7mer-m8 | ucgcccugaaacuCCCGGUCAa | cagcugaaucccuGGGCCAGUu |
| NM_013451 | MYOF | circRNA | chr10 | 95107448 | 95107472 | - | 5 | 0 | AGO1-4,AGO2 | 7mer-m8 | ucGCC--CUGA-AACUCCCGGUCAa | ggCGGAAGCCUGUCGUCGGCCAGUg |
| NM_013451 | MYOF | circRNA | chr10 | 95169420 | 95169441 | - | 3 | 0 | AGO1-4 | 7mer-m8 | ucGCCCUGAAACUCCCGGUCAa | ccCUGGGCCCAAGGGGCCAGUu |
| NM_002860 | ALDH18A1 | circRNA | chr10 | 97373744 | 97373764 | - | 2 | 1 | AGO1-4,AGO2 | 7mer-m8 | ucgcCCUGAAACUCCCGGUCAa | auguGGAUUCCGA-GGCCAGUg |
| NM_001134375 | CCNJ | circRNA | chr10 | 97810023 | 97810044 | + | 2 | 0 | AGO1-4 | 7mer-m8 | ucgcccuGAAACUCCCGGUCAa | ugcccucCUAUAAAGGCCAGUc |
| NM_138413 | HOGA1 | circRNA | chr10 | 99416039 | 99416060 | + | 2 | 0 | AGO1-4 | 7mer-m8 | ucgcccugaaacucCCGGUCAa | uagguaguauaccuGGCCAGUg |
| NM_018425 | PI4K2A | circRNA | chr10 | 99416042 | 99416060 | + | 2 | 0 | AGO1-4 | 7mer-m8 | ucgcccugaaacucCCGGUCAa | #NAME? |
| NM_138413 | HOGA1 | circRNA | chr10 | 99416042 | 99416060 | + | 2 | 0 | AGO1-4 | 7mer-m8 | ucgcccugaaacucCCGGUCAa | #NAME? |
| NM_017902 | HIF1AN | circRNA | chr10 | 102312828 | 102312850 | + | 2 | 0 | AGO1-4,AGO2 | 7mer-m8 | ucgCCCU-GAAACUCCCGGUCAa | gugGGCACCUACAAGGGCCAGUu |
| NM_018121 | FAM178A | circRNA | chr10 | 102724472 | 102724488 | + | 2 | 0 | AGO1-4,AGO2 | 8mer | ucgCCCUGAAACUCCCGGUCAa | gugGGGACU-----GGCCAGUa |
| NM_032112 | MRPL43 | circRNA | chr10 | 102746582 | 102746603 | - | 1 | 0 | AGO1-4 | 7mer-m8 | ucgcccUGAAACUCCCGGUCAa | acccuaGCAUCCAGGGCCAGUg |
| NM_032429 | LZTS2 | circRNA | chr10 | 102763461 | 102763486 | + | 3 | 0 | AGO1-4,AGO2 | 7mer-m8 | ucGCCCUGAA--ACU--CCCGGUCAa | ccUGGCAUUUAGUGGCUGGGCCAGUg |
| NM_030929 | KAZALD1 | circRNA | chr10 | 102824915 | 102824936 | + | 1 | 0 | AGO2 | 8mer | ucgcccugaaacucCCGGUCAa | gcaggagaugcccuGGCCAGUa |
| NM_033637 | BTRC | circRNA | chr10 | 103314180 | 103314203 | + | 1 | 0 | AGO2 | 7mer-m8 | ucgcccUGAAAC--UCCCGGUCAa | uguuuuGUUUUGUUUUGGCCAGUu |
| NM_033637 | BTRC | circRNA | chr10 | 103316805 | 103316826 | + | 3 | 0 | AGO1-4,AGO2 | 7mer-m8 | ucgcccugaaaCUCCCGGUCAa | caaaucugaggGAUGGCCAGUg |
| NM_015062 | PPRC1 | circRNA | chr10 | 103900972 | 103900992 | + | 7 | 0 | AGO1-4,AGO2 | 8mer | ucgccCUGAAACUCCCGGUCAa | ccauuGUCUAUG-GGGCCAGUa |
| NM_024326 | FBXL15 | circRNA | chr10 | 104182823 | 104182849 | + | 1 | 0 | AGO1-4 | 7mer-m8 | ucgcCCUGAA----ACUC-CCGGUCAa | gugaGGACCUCUGGUGAGAGGCCAGUg |
| NM_030912 | TRIM8 | circRNA | chr10 | 104416594 | 104416617 | + | 1 | 0 | AGO1-4 | 7mer-m8 | ucgcCCUGAA-ACUC-CCGGUCAa | caacGGCCUUCCCAGAGGCCAGUu |
| NM_020383 | XPNPEP1 | circRNA | chr10 | 111624644 | 111624665 | - | 4 | 0 | AGO1-4,AGO2 | 7mer-m8 | ucgccCUGAAACUCCCGGUCAa | gccccGACUUCUUUGGCCAGUg |
| NM_022494 | ZDHHC6 | circRNA | chr10 | 114194085 | 114194106 | - | 6 | 0 | AGO1-4,AGO2 | 8mer | ucgcCCUGAAACUC-CCGGUCAa | agauGGAC-UUGAGUGGCCAGUa |
| NM_020940 | FAM160B1 | circRNA | chr10 | 116595376 | 116595397 | + | 1 | 0 | AGO2 | 7mer-m8 | ucgcccugaaacucCCGGUCAa | cauuaacgugcacaGGCCAGUg |
| NM_139169 | TRUB1 | circRNA | chr10 | 116731960 | 116731981 | + | 5 | 0 | AGO1-4,AGO2 | 7mer-m8 | ucgcccugaaacucCCGGUCAa | agcaaaaccugccaGGCCAGUg |
| NM_025015 | HSPA12A | circRNA | chr10 | 118592640 | 118592661 | - | 1 | 0 | AGO2 | 7mer-m8 | ucgcCCUGAAACUCCCGGUCAa | ccauGGGCAUUUGCGGCCAGUg |
| NM_173791 | PDZD8 | circRNA | chr10 | 119044850 | 119044868 | - | 8 | 0 | AGO1-4,AGO2 | 7mer-m8 | ucgcccUGAAACUCCCGGUCAa | aagauaACUUU---GGCCAGUu |
| NM_001380 | DOCK1 | circRNA | chr10 | 129207616 | 129207638 | + | 12 | 0 | AGO1,AGO1-4,AGO2 | 7mer-m8 | ucgcccUGAAACU-CCCGGUCAa | cuccuaAGUUUCACAGGCCAGUg |
| NM_006659 | TUBGCP2 | circRNA | chr10 | 135106064 | 135106085 | - | 1 | 0 | AGO1-4 | 7mer-m8 | ucgcccUGAAACUCCCGGUCAa | uaccuaACCAAGGCGGCCAGUg |
| NM_021932 | RIC8A | circRNA | chr11 | 212909 | 212930 | + | 1 | 1 | AGO1-4 | 8mer | ucgcccugaaaCUCCCGGUCAa | gaggccggcccGAGGGCCAGUa |
| NM_001242837 | AP2A2 | circRNA | chr11 | 986808 | 986829 | + | 2 | 0 | AGO1-4 | 7mer-m8 | ucgcccugaaacuCCCGGUCAa | ccugcaaccaguuGGGCCAGUu |
| NM_012305 | AP2A2 | circRNA | chr11 | 986808 | 986829 | + | 2 | 0 | AGO1-4 | 7mer-m8 | ucgcccugaaacuCCCGGUCAa | ccugcaaccaguuGGGCCAGUu |
| NM_001242932 | LSP1 | circRNA | chr11 | 1908013 | 1908031 | + | 1 | 0 | AGO1-4 | 8mer | ucgccCUGAAACUCCCGGUCAa | agcauGGCUGU---GGCCAGUa |
| TCONS_00063837_H19 | TCONS_00063837_H19 | circRNA | chr11 | 2018870 | 2018889 | - | 4 | 0 | AGO1-4 | 7mer-m8 | ucGCCCUGAAACUCCCGGUCAa | ggUGGGGC-CUGA-GGCCAGUg |
| NM_003311 | PHLDA2 | circRNA | chr11 | 2949660 | 2949681 | - | 9 | 0 | AGO1,AGO1-4,AGO2 | 7mer-m8 | ucgcccugaaacucCCGGUCAa | caaauaaaucacuuGGCCAGUu |
| NM_016320 | NUP98 | circRNA | chr11 | 3707276 | 3707297 | - | 1 | 0 | AGO1-4 | 7mer-m8 | ucgcccugaaacucCCGGUCAa | gaaggugugcuacaGGCCAGUu |
| NM_032127 | FAM160A2 | circRNA | chr11 | 6245913 | 6245935 | - | 1 | 0 | AGO1-4 | 7mer-m8 | ucgcccUGAAACU-CCCGGUCAa | uuagaaAUCUGGACUGGCCAGUg |
| NM_144666 | DNHD1 | circRNA | chr11 | 6584738 | 6584759 | + | 1 | 0 | AGO1-4 | 7mer-m8 | ucgcccugaaacuCCCGGUCAa | caugaaacaggcuGGGCCAGUg |
| NM_000391 | TPP1 | circRNA | chr11 | 6634129 | 6634152 | - | 16 | 0 | AGO1,AGO1-4,AGO2 | 7mer-m8 | ucGCCCUGAAAC--UCCCGGUCAa | caCGUAGUAUUGAAAUGGCCAGUu |
| NM_000391 | TPP1 | circRNA | chr11 | 6637955 | 6637973 | - | 1 | 0 | AGO1-4 | 7mer-m8 | ucGCCCUGAAACUCCCGGUCAa | gcCGGGA--UUGA-GGCCAGUc |
| NM_000990 | RPL27A | circRNA | chr11 | 8707533 | 8707551 | + | 2 | 0 | AGO1,AGO1-4 | 7mer-m8 | ucgccCUGAAACUCCCGGUCAa | acacaGACGUU---GGCCAGUu |
| NM_005418 | ST5 | circRNA | chr11 | 8715332 | 8715355 | - | 3 | 0 | AGO1-4 | 7mer-m8 | ucgCCCUGA-AACU-CCCGGUCAa | cugGGUGCUCGUGACUGGCCAGUu |
| NM_005418 | ST5 | circRNA | chr11 | 8752242 | 8752266 | - | 1 | 0 | AGO1-4 | 7mer-m8 | ucGCCCU-G-AAACUC-CCGGUCAa | ucUGGGAGCGAGUGGGCGGCCAGUg |
| NM_015012 | TMEM41B | circRNA | chr11 | 9304032 | 9304053 | - | 2 | 0 | AGO2 | 7mer-m8 | ucgcccugaaacucCCGGUCAa | aaauuuaaaguucuGGCCAGUc |
| NM_032867 | MICALCL | circRNA | chr11 | 12315467 | 12315488 | + | 1 | 0 | AGO1-4 | 7mer-m8 | ucgcccugaaaCUCCCGGUCAa | ggaagaagaggGAGGGCCAGUg |
| NM_021961 | TEAD1 | circRNA | chr11 | 12961608 | 12961629 | + | 4 | 0 | AGO1-4 | 7mer-m8 | ucgccCUGAAACUCCCGGUCAa | cugcaGAGUUUGGAGGCCAGUu |
| NM_016451 | COPB1 | circRNA | chr11 | 14497414 | 14497434 | - | 4 | 0 | AGO1-4,AGO2 | 7mer-m8 | ucgcccUGAAACUCCCGGUCAa | gaaauaACUGU-AGGGCCAGUu |
| NM_148976 | PSMA1 | circRNA | chr11 | 14632643 | 14632665 | - | 1 | 0 | AGO1-4 | 8mer | ucGCCCUGAAAC-UCCCGGUCAa | guCAGCUCUCAGCAAGGCCAGUa |
| NM_012139 | SERGEF | circRNA | chr11 | 18026037 | 18026055 | - | 3 | 0 | AGO2 | 7mer-m8 | ucgcccuGAAACUCCCGGUCAa | ccaacucCUUU---GGCCAGUu |
| NM_138421 | SAAL1 | circRNA | chr11 | 18111797 | 18111819 | - | 1 | 0 | AGO2 | 7mer-m8 | ucgcccugaaacUC-CCGGUCAa | uucccaggcagaAGUGGCCAGUg |
| NM_001098522 | HTATIP2 | circRNA | chr11 | 20404594 | 20404614 | + | 2 | 0 | AGO1-4,AGO2 | 7mer-m8 | ucgccCUGAAACUCCCGGUCAa | uaccaGACUCU-UGGGCCAGUg |
| NM_018393 | TCP11L1 | circRNA | chr11 | 33090357 | 33090378 | + | 1 | 0 | AGO1-4 | 7mer-m8 | ucgcccugaaacucCCGGUCAa | cagauccaggccguGGCCAGUc |
| NM_005734 | HIPK3 | circRNA | chr11 | 33308985 | 33309008 | + | 3 | 0 | AGO1-4,AGO2 | 7mer-m8 | ucgccCUGAAACUC--CCGGUCAa | uaauaGACUUUGGGUCGGCCAGUc |
| hsa_circ_0000284 | hsa_circ_000016 | circRNA | chr11 | 33308985 | 33309008 | + | 3 | 0 | AGO1-4,AGO2 | 7mer-m8 | ucgccCUGAAACUC--CCGGUCAa | uaauaGACUUUGGGUCGGCCAGUc |
| NM_005898 | CAPRIN1 | circRNA | chr11 | 34113510 | 34113531 | + | 11 | 0 | AGO1,AGO1-4,AGO2,AGO3 | 7mer-m8 | ucgcccugaaacucCCGGUCAa | caaaaucaguaccaGGCCAGUu |
| NM_203364 | CAPRIN1 | circRNA | chr11 | 34113510 | 34113531 | + | 11 | 0 | AGO1,AGO1-4,AGO2,AGO3 | 7mer-m8 | ucgcccugaaacucCCGGUCAa | caaaaucaguaccaGGCCAGUu |
| hsa_circ_0000287 | hsa_circ_001353 | circRNA | chr11 | 34113510 | 34113531 | + | 11 | 0 | AGO1,AGO1-4,AGO2,AGO3 | 7mer-m8 | ucgcccugaaacucCCGGUCAa | caaaaucaguaccaGGCCAGUu |
| NM_005898 | CAPRIN1 | circRNA | chr11 | 34120941 | 34120962 | + | 9 | 0 | AGO1-4,AGO2 | 7mer-m8 | ucgcccugaaacucCCGGUCAa | gccaaaaacacacuGGCCAGUg |
| NM_024662 | NAT10 | circRNA | chr11 | 34162694 | 34162715 | + | 1 | 0 | AGO1-4 | 7mer-m8 | ucgcccugaaacuCCCGGUCAa | uugagcugcccucGGGCCAGUu |
| NM_001752 | CAT | circRNA | chr11 | 34478327 | 34478347 | + | 1 | 0 | AGO1-4 | 7mer-m8 | ucgccCUGAAACUCCCGGUCAa | caccuGGCAUUGA-GGCCAGUc |
| NM_018259 | TTC17 | circRNA | chr11 | 43441767 | 43441788 | + | 1 | 0 | AGO1-4 | 7mer-m8 | ucgccCUGAAACUCCCGGUCAa | ccucuGUCUUCCAGGGCCAGUg |
| NM_001101802 | PHF21A | circRNA | chr11 | 45959814 | 45959836 | - | 1 | 0 | AGO2 | 7mer-m8 | ucgcccugaaacUC-CCGGUCAa | guuugcagaaaaAGUGGCCAGUu |
| NM_017749 | AMBRA1 | circRNA | chr11 | 46465110 | 46465135 | - | 2 | 0 | AGO1-4 | 8mer | ucgcCCUG--AAAC--UCCCGGUCAa | ugauGGACAGUCUGCUGUGGCCAGUa |
| NM_004308 | ARHGAP1 | circRNA | chr11 | 46700209 | 46700234 | - | 5 | 0 | AGO1-4,AGO2 | 7mer-m8 | ucGCCCUGA--AAC--UCCCGGUCAa | ggCUGGGCUGGUAGCAGGGGCCAGUc |
| NM_001184975 | PACSIN3 | circRNA | chr11 | 47199540 | 47199564 | - | 1 | 0 | AGO1-4 | 7mer-m8 | ucGCCCUGA-AAC--UCCCGGUCAa | agCAGGGCUGGUGCCAAGGCCAGUu |
| NM_175732 | PTPMT1 | circRNA | chr11 | 47591355 | 47591376 | + | 5 | 0 | AGO1-4,AGO2 | 7mer-m8 | ucgcccugaaacuCCCGGUCAa | aguaccagucgcuGGGCCAGUg |
| NM_014342 | MTCH2 | circRNA | chr11 | 47663999 | 47664020 | - | 5 | 0 | AGO1-4,AGO2 | 7mer-m8 | ucgcccugaaacucCCGGUCAa | aucauggcggacgcGGCCAGUc |
| NM_033396 | TNKS1BP1 | circRNA | chr11 | 57087603 | 57087625 | - | 2 | 0 | AGO2 | 8mer | ucGCCCUGAAACU-CCCGGUCAa | gaUGGUCCCAGGAGGGGCCAGUa |
| NM_198183 | UBE2L6 | circRNA | chr11 | 57319549 | 57319569 | - | 1 | 0 | AGO1-4 | 7mer-m8 | ucgcCCUGAAACUCCCGGUCAa | guuuGCAGUUACA-GGCCAGUu |
| NM_015457 | ZDHHC5 | circRNA | chr11 | 57466212 | 57466236 | + | 1 | 0 | AGO1-4 | 7mer-m8 | ucgccCUGAAACU---CCCGGUCAa | gcacaGGCUUUGAGCUGGGCCAGUu |
| NM_152716 | PATL1 | circRNA | chr11 | 59426731 | 59426752 | - | 1 | 0 | AGO2 | 7mer-m8 | ucgcccugaaacucCCGGUCAa | ggcagugcagaccaGGCCAGUu |
| NM_001161454 | CYBASC3 | circRNA | chr11 | 61125231 | 61125256 | - | 4 | 0 | AGO1,AGO2 | 7mer-m8 | ucGCCCUGAAACUC----CCGGUCAa | cgCAGGAAUUGGAGCCCAGGCCAGUu |
| NM_024811 | CPSF7 | circRNA | chr11 | 61187948 | 61187969 | - | 4 | 0 | AGO1-4,AGO2 | 7mer-m8 | ucgcccugaaacUCCCGGUCAa | agaaucgagcaaAUGGCCAGUc |
| NM_022830 | TUT1 | circRNA | chr11 | 62356523 | 62356542 | - | 3 | 0 | AGO2 | 7mer-m8 | ucgcCCUGAAACUCCCGGUCAa | auuuGGACCU--GUGGCCAGUg |
| NM_017490 | MARK2 | circRNA | chr11 | 63677923 | 63677945 | + | 3 | 0 | AGO1-4 | 7mer-m8 | ucgcccUGAAACUC-CCGGUCAa | cuugcuACCCUCAGUGGCCAGUg |
| NM_006795 | EHD1 | circRNA | chr11 | 64620501 | 64620524 | - | 3 | 0 | AGO1-4,AGO2 | 7mer-m8 | ucGCCC-UG-AAACUCCCGGUCAa | ccCGGGCGCGUUUGCAGGCCAGUg |
| NM_006795 | EHD1 | circRNA | chr11 | 64620576 | 64620600 | - | 5 | 0 | AGO1-4,AGO2 | 7mer-m8 | ucGCCCUGAAACU---CCCGGUCAa | ugCGGCCCAGUGGCCCGGGCCAGUu |
| NM_001997 | FAU | circRNA | chr11 | 64889022 | 64889043 | - | 15 | 1 | AGO1-4,AGO2 | 7mer-m8 | ucgcCCUGAAACUCCCGGUCAa | augaGGCCACUCUGGGCCAGUg |
| NM_002689 | POLA2 | circRNA | chr11 | 65029608 | 65029631 | + | 1 | 0 | AGO1-4 | 7mer-m8 | ucGCCCUGAAAC--UCCCGGUCAa | ucUGAGGUCUUGCUUGGGCCAGUc |
| NM_006779 | CDC42EP2 | circRNA | chr11 | 65089101 | 65089122 | + | 7 | 0 | AGO1-4,AGO2 | 7mer-m8 | ucgcccugaAACUCCCGGUCAa | ccuguauggUCGCUGGCCAGUg |
| hsa_circ_0003812 | hsa_circ_0003812 | circRNA | chr11 | 65204008 | 65204029 | + | 9 | 0 | AGO1-4 | 7mer-m8 | ucgcccUGAAACUCCCGGUCAa | gaggccACCGUCAUGGCCAGUu |
| hsa_circ_0006417 | hsa_circ_0006417 | circRNA | chr11 | 65204008 | 65204029 | + | 9 | 0 | AGO1-4 | 7mer-m8 | ucgcccUGAAACUCCCGGUCAa | gaggccACCGUCAUGGCCAGUu |
| hsa_circ_0007185 | hsa_circ_0007185 | circRNA | chr11 | 65204008 | 65204029 | + | 9 | 0 | AGO1-4 | 7mer-m8 | ucgcccUGAAACUCCCGGUCAa | gaggccACCGUCAUGGCCAGUu |
| hsa_circ_0002791 | hsa_circ_0002791 | circRNA | chr11 | 65204009 | 65204029 | + | 9 | 0 | AGO1-4 | 7mer-m8 | ucgcccUGAAACUCCCGGUCAa | #NAME? |
| hsa_circ_0003812 | hsa_circ_0003812 | circRNA | chr11 | 65204009 | 65204029 | + | 9 | 0 | AGO1-4 | 7mer-m8 | ucgcccUGAAACUCCCGGUCAa | #NAME? |
| hsa_circ_0006417 | hsa_circ_0006417 | circRNA | chr11 | 65204009 | 65204029 | + | 9 | 0 | AGO1-4 | 7mer-m8 | ucgcccUGAAACUCCCGGUCAa | #NAME? |
| hsa_circ_0007185 | hsa_circ_0007185 | circRNA | chr11 | 65204009 | 65204029 | + | 9 | 0 | AGO1-4 | 7mer-m8 | ucgcccUGAAACUCCCGGUCAa | #NAME? |
| hsa_circ_0005929 | hsa_circ_0005929 | circRNA | chr11 | 65211182 | 65211203 | + | 6 | 0 | AGO1-4,AGO2 | 7mer-m8 | ucgcccugaaacUCCCGGUCAa | uggccagagggaAGGGCCAGUc |
| NM_001130144 | LTBP3 | circRNA | chr11 | 65321815 | 65321836 | - | 1 | 0 | AGO1-4 | 7mer-m8 | ucgcccugaaACUCCCGGUCAa | ccugcaugaaUGGCGGCCAGUg |
| NM_001130144 | LTBP3 | circRNA | chr11 | 65325192 | 65325216 | - | 4 | 0 | AGO1-4,AGO2 | 7mer-m8 | ucGCCCUGA---AACUCCCGGUCAa | agCGGACCUGUCUCAAGGGCCAGUg |
| NM_003860 | BANF1 | circRNA | chr11 | 65771098 | 65771119 | + | 6 | 0 | AGO1-4,AGO2 | 7mer-m8 | ucgcccugaaacucCCGGUCAa | ccuauguuguccuuGGCCAGUu |
| NM_006842 | SF3B2 | circRNA | chr11 | 65825809 | 65825830 | + | 3 | 1 | AGO1-4,AGO3 | 7mer-m8 | ucgcccugaaacuCCCGGUCAa | cucgcucgucccuGGGCCAGUc |
| NM_018026 | PACS1 | circRNA | chr11 | 66001269 | 66001289 | + | 2 | 0 | AGO1-4,AGO2 | 7mer-m8 | ucgcccugaaaCUCCCGGUCAa | ucuuccaugccGA-GGCCAGUg |
| NM_005125 | CCS | circRNA | chr11 | 66366698 | 66366722 | + | 1 | 0 | AGO1-4 | 7mer-m8 | ucgcCCUGAAAC--UC-CCGGUCAa | ucaaGGGCAUGGGCAGCGGCCAGUu |
| NM_001198845 | RBM14-RBM4 | circRNA | chr11 | 66411274 | 66411295 | + | 14 | 0 | AGO1,AGO1-4,AGO2 | 7mer-m8 | ucgcccugaaacUCCCGGUCAa | cagaauacagccAUGGCCAGUc |
| NM_002896 | RBM4 | circRNA | chr11 | 66411274 | 66411295 | + | 14 | 0 | AGO1,AGO1-4,AGO2 | 7mer-m8 | ucgcccugaaacUCCCGGUCAa | cagaauacagccAUGGCCAGUc |
| NM_006946 | SPTBN2 | circRNA | chr11 | 66488656 | 66488677 | - | 3 | 0 | AGO1,AGO1-4,AGO2 | 8mer | ucgcCCUGAAACUCCCGGUCAa | gcuuGGAAAUCCAGGGCCAGUa |
| NM_001040716 | PC | circRNA | chr11 | 66617797 | 66617818 | - | 1 | 0 | AGO2 | 8mer | ucgcccugaaaCUCCCGGUCAa | augagaucccaGGGGGCCAGUa |
| NM_024036 | LRFN4 | circRNA | chr11 | 66625231 | 66625252 | + | 4 | 0 | AGO1-4,AGO2 | 7mer-m8 | ucgcccugaaACUCCCGGUCAa | cugcugcugcUGCUGGCCAGUg |
| NM_001177880 | SYT12 | circRNA | chr11 | 66816226 | 66816247 | + | 1 | 0 | AGO1-4 | 7mer-m8 | ucgcccugaaaCUCCCGGUCAa | uagcaaccaggGCGGGCCAGUu |
| NR_030767 | ANKRD13D | circRNA | chr11 | 67059572 | 67059593 | + | 1 | 0 | AGO2 | 7mer-m8 | ucGC-CCUGAAACUCCCGGUCAa | agCGAGGAGCAUG-UGGCCAGUc |
| NM_004910 | PITPNM1 | circRNA | chr11 | 67265018 | 67265039 | - | 1 | 0 | AGO1 | 7mer-m8 | ucgcccugaaacUCCCGGUCAa | auccccagcgacAUGGCCAGUc |
| NM_053056 | CCND1 | circRNA | chr11 | 69467482 | 69467505 | + | 24 | 0 | AGO1-4,AGO2 | 8mer | ucgcCCUGAAA--CUCCCGGUCAa | cagaGGAUGUUCAUAAGGCCAGUa |
| NM_012309 | SHANK2 | circRNA | chr11 | 70332905 | 70332925 | - | 2 | 0 | AGO1,AGO2 | 7mer-m8 | ucgCCCUGAAACUCCCGGUCAa | uggGUGGCGCCGA-GGCCAGUg |
| NM_006185 | NUMA1 | circRNA | chr11 | 71714248 | 71714272 | - | 2 | 0 | AGO1-4 | 7mer-m8 | ucGCCCUGAAACUC---CCGGUCAa | gcUGGGUCCUUGGGCCUGGCCAGUc |
| NM_006185 | NUMA1 | circRNA | chr11 | 71714563 | 71714583 | - | 4 | 0 | AGO1-4,AGO2 | 8mer | ucgcCCUGAAACUCCCGGUCAa | caaaGCAC-UAAAGGGCCAGUa |
| NM_017907 | LAMTOR1 | circRNA | chr11 | 71809364 | 71809385 | - | 4 | 0 | AGO1-4,AGO2 | 7mer-m8 | ucgcccugaaACUCCCGGUCAa | ccccaccaagUGCUGGCCAGUg |
| NM_001567 | INPPL1 | circRNA | chr11 | 71948612 | 71948634 | + | 3 | 0 | AGO1-4,AGO2 | 7mer-m8 | ucgcccugaaacUC-CCGGUCAa | uuuccugggggaAGUGGCCAGUg |
| NM_001040118 | ARAP1 | circRNA | chr11 | 72423565 | 72423588 | - | 1 | 0 | AGO1-4 | 7mer-m8 | ucGCCCUG-AAACUC-CCGGUCAa | caCGGGCCGUGCGCGUGGCCAGUc |
| NM_003355 | UCP2 | circRNA | chr11 | 73686602 | 73686626 | - | 4 | 0 | AGO1-4,AGO2 | 8mer | ucgcCCUGAAAC---UCCCGGUCAa | acauGAACUCUGCCCUGGGCCAGUa |
| NM_003355 | UCP2 | circRNA | chr11 | 73689115 | 73689136 | - | 1 | 0 | AGO1-4 | 7mer-m8 | ucgcccugaaacUCCCGGUCAa | aggagaaagucaGGGGCCAGUg |
| NM_001098638 | RNF169 | circRNA | chr11 | 74546909 | 74546930 | + | 2 | 0 | AGO1-4,AGO2 | 7mer-m8 | ucgcccuGAA-ACUCCCGGUCAa | cagacuuCUUAUGA-GGCCAGUc |
| NM_030792 | GDPD5 | circRNA | chr11 | 75154191 | 75154214 | - | 1 | 0 | AGO1 | 7mer-m8 | ucgCCCUGA--AACUCCCGGUCAa | gcaGAGACUCAACGCUGGCCAGUg |
| NM_016578 | RSF1 | circRNA | chr11 | 77377900 | 77377921 | - | 4 | 0 | AGO1-4,AGO2 | 7mer-m8 | ucgcccugaaaCUCCCGGUCAa | cucucacaccaGCGGGCCAGUu |
| NM_016578 | RSF1 | circRNA | chr11 | 77412295 | 77412317 | - | 6 | 0 | AGO1-4,AGO2 | 7mer-m8 | ucgcCCUGA-AACUCCCGGUCAa | acaaGUACUGUUGGUGGCCAGUc |
| hsa_circ_0000345 | hsa_circ_001227 | circRNA | chr11 | 77412295 | 77412317 | - | 6 | 0 | AGO1-4,AGO2 | 7mer-m8 | ucgcCCUGA-AACUCCCGGUCAa | acaaGUACUGUUGGUGGCCAGUc |
| NM_182603 | ANKRD42 | circRNA | chr11 | 82923503 | 82923525 | + | 1 | 0 | AGO1-4 | 7mer-m8 | ucgcccugaaacUC-CCGGUCAa | aaccuacaguaaAGUGGCCAGUg |
| NM_021825 | CCDC90B | circRNA | chr11 | 82991257 | 82991281 | - | 2 | 2 | AGO1-4,AGO2 | 7mer-m8 | ucgCCCUG-A--AACUCCCGGUCAa | ggaGGGAUAUGAUAGGCGGCCAGUg |
| NM_001142699 | DLG2 | circRNA | chr11 | 83180299 | 83180320 | - | 1 | 0 | AGO2 | 8mer | ucgcccugaaacucCCGGUCAa | aguuuauagaagccGGCCAGUa |
| NM_007173 | PRSS23 | circRNA | chr11 | 85991393 | 85991413 | + | 1 | 0 | AGO1-4 | 7mer-m8 | ucGCCCUGAAACUCCCGGUCAa | guCAGAACUCACA-GGCCAGUu |
| NM_007173 | PRSS23 | circRNA | chr11 | 86750483 | 86750508 | + | 1 | 0 | AGO2 | 8mer | ucgccCUGAAACUC----CCGGUCAa | auaaaGAUCUAGAGUAGAGGCCAGUa |
| NM_024116 | TAF1D | circRNA | chr11 | 93474559 | 93474580 | - | 3 | 0 | AGO1-4,AGO2 | 7mer-m8 | ucgcccugaaacucCCGGUCAa | gcucuaggacaccaGGCCAGUc |
| NM_015036 | ENDOD1 | circRNA | chr11 | 94865032 | 94865053 | + | 2 | 0 | AGO1-4,AGO2 | 7mer-m8 | ucgcccuGAAACUCCCGGUCAa | auuuauuCUUGGGUGGCCAGUu |
| NM_032427 | MAML2 | circRNA | chr11 | 95825675 | 95825697 | - | 1 | 0 | AGO1-4 | 7mer-m8 | ucgcccugaaacUC-CCGGUCAa | guagcuggcggcAGCGGCCAGUc |
| NM_020886 | USP28 | circRNA | chr11 | 113700031 | 113700050 | - | 1 | 0 | AGO1-4 | 8mer | ucGCCCUGAAACUCCCGGUCAa | aaUGAGACCUU--CGGCCAGUa |
| NM_001197104 | MLL | circRNA | chr11 | 118395497 | 118395518 | + | 21 | 0 | AGO1-4,AGO2 | 7mer-m8 | ucgcccugaaacUCCCGGUCAa | gagaaagacccaAAGGCCAGUc |
| NM_001197104 | MLL | circRNA | chr11 | 118396033 | 118396056 | + | 21 | 0 | AGO1,AGO1-4,AGO2 | 7mer-m8 | ucGCCCUGAA--ACUCCCGGUCAa | ggCUGCAUUUCCUGGUGGCCAGUg |
| NM_001164277 | SLC37A4 | circRNA | chr11 | 118897685 | 118897703 | - | 3 | 0 | AGO1-4 | 7mer-m8 | ucgccCUGAAACUCCCGGUCAa | guacuGACU---GGGGCCAGUu |
| NM_006389 | HYOU1 | circRNA | chr11 | 118915861 | 118915881 | - | 10 | 0 | AGO1-4,AGO2 | 7mer-m8 | ucgcccuGAAACUCCCGGUCAa | gagcaucCUUUCA-GGCCAGUg |
| NM_001382 | DPAGT1 | circRNA | chr11 | 118971022 | 118971046 | - | 2 | 0 | AGO1-4,AGO2 | 7mer-m8 | ucgccCUGAAACUC---CCGGUCAa | uuaacGGCCUAGAGGCUGGCCAGUc |
| NM_014619 | GRIK4 | circRNA | chr11 | 120525354 | 120525375 | + | 1 | 0 | AGO1-4 | 8mer | ucgcccugaaacucCCGGUCAa | uagccaccacaccuGGCCAGUa |
| NM_006597 | HSPA8 | circRNA | chr11 | 122930457 | 122930478 | - | 34 | 1 | AGO1,AGO1-4,AGO2,AGO3 | 8mer | ucgcccugaaacucCCGGUCAa | ucuuccagcacccaGGCCAGUa |
| NM_153201 | HSPA8 | circRNA | chr11 | 122930457 | 122930478 | - | 34 | 1 | AGO1,AGO1-4,AGO2,AGO3 | 8mer | ucgcccugaaacucCCGGUCAa | ucuuccagcacccaGGCCAGUa |
| NM_001114122 | CHEK1 | circRNA | chr11 | 125525716 | 125525737 | + | 3 | 0 | AGO1,AGO1-4,AGO2 | 7mer-m8 | ucgcccugaaacucCCGGUCAa | uuucaaaagggccuGGCCAGUu |
| NM_001143835 | NFRKB | circRNA | chr11 | 129739838 | 129739859 | - | 2 | 0 | AGO1-4,AGO2 | 7mer-m8 | ucgcccuGAAACUCCCGGUCAa | gauagccCUGCCAAGGCCAGUu |
| NM_014155 | ZBTB44 | circRNA | chr11 | 130100093 | 130100113 | - | 1 | 0 | AGO2 | 7mer-m8 | ucgcccugaAACUCCCGGUCAa | uaaucuaaaUAGA-GGCCAGUu |
| NR_027948 | ERC1 | circRNA | chr12 | 1600068 | 1600089 | + | 1 | 0 | AGO2 | 7mer-m8 | ucgcccugaaacucCCGGUCAa | ucacucuguuccucGGCCAGUu |
| NM_003213 | TEAD4 | circRNA | chr12 | 3131025 | 3131044 | + | 15 | 0 | AGO1-4,AGO2 | 7mer-m8 | ucGC-CCUGAAACUCCCGGUCAa | uuCGUGCACAUU---GGCCAGUc |
| NM_000552 | VWF | circRNA | chr12 | 6085386 | 6085407 | - | 3 | 0 | AGO1-4 | 7mer-m8 | ucgcccUGAAACUCCCGGUCAa | ccaucuACCCUGUGGGCCAGUu |
| NM_014865 | NCAPD2 | circRNA | chr12 | 6626615 | 6626636 | + | 4 | 0 | AGO1-4,AGO2 | 7mer-m8 | ucgcccugaaACUCCCGGUCAa | gccauccagcUGCUGGCCAGUu |
| NM_001273 | CHD4 | circRNA | chr12 | 6715603 | 6715617 | - | 1 | 0 | AGO1-4 | 7mer-m8 | ucgcccugaaacuCCCGGUCAa | #NAME? |
| NM_006331 | EMG1 | circRNA | chr12 | 7080217 | 7080239 | + | 3 | 0 | AGO1-4,AGO2 | 7mer-m8 | ucGCCCUGAAACU-CCCGGUCAa | ugUGGUGCUGGAAGGGGCCAGUc |
| NM_001131023 | PEX5 | circRNA | chr12 | 7354372 | 7354393 | + | 2 | 0 | AGO2 | 7mer-m8 | ucgcccugaaacUCCCGGUCAa | gaucugcagcacACGGCCAGUg |
| NM_001131025 | PEX5 | circRNA | chr12 | 7354372 | 7354393 | + | 2 | 0 | AGO2 | 7mer-m8 | ucgcccugaaacUCCCGGUCAa | gaucugcagcacACGGCCAGUg |
| NM_176884 | TAS2R43 | circRNA | chr12 | 11262882 | 11262907 | - | 1 | 0 | AGO1-4 | 7mer-m8 | ucgccCUGAAACU--C--CCGGUCAa | ucccuGAAUUUGAAUGUUGGCCAGUc |
| NR_037918 | PRH1-PRR4 | circRNA | chr12 | 11262882 | 11262907 | - | 1 | 0 | AGO1-4 | 7mer-m8 | ucgccCUGAAACU--C--CCGGUCAa | ucccuGAAUUUGAAUGUUGGCCAGUc |
| NM_001987 | ETV6 | circRNA | chr12 | 12048243 | 12048264 | + | 5 | 0 | AGO1-4,AGO2 | 7mer-m8 | ucgcccuGAAACUCCCGGUCAa | augccuuUUCUCAGGGCCAGUg |
| NM_002336 | LRP6 | circRNA | chr12 | 12270872 | 12270893 | - | 6 | 0 | AGO1,AGO2 | 8mer | ucgcccugaaacucCCGGUCAa | uuuaaucuaauauaGGCCAGUa |
| NM_015954 | DERA | circRNA | chr12 | 16115856 | 16115876 | + | 4 | 0 | AGO1-4,AGO2 | 7mer-m8 | ucGCCCUGAAACUCCCGGUCAa | cuUGGUGCUGACA-GGCCAGUg |
| NM_019844 | SLCO1B3 | circRNA | chr12 | 21078540 | 21078562 | + | 1 | 0 | AGO2 | 7mer-m8 | ucgcccUGAAACUC-CCGGUCAa | aguuuuAUUUCGUGUGGCCAGUu |
| NM_016551 | TM7SF3 | circRNA | chr12 | 27148178 | 27148199 | - | 2 | 0 | AGO1-4,AGO2 | 7mer-m8 | ucgcccugaaACUCCCGGUCAa | gugccccaggUGAAGGCCAGUg |
| NM_175861 | TMTC1 | circRNA | chr12 | 29830068 | 29830088 | - | 1 | 0 | AGO1-4 | 7mer-m8 | ucgcccUGAAACUCCCGGUCAa | cagcaaAAUUAGA-GGCCAGUg |
| NM_001242397 | TWF1 | circRNA | chr12 | 44189093 | 44189114 | - | 6 | 0 | AGO1,AGO1-4,AGO2 | 7mer-m8 | ucgcccugaaacuCCCGGUCAa | auuuuauacaguuGGGCCAGUu |
| NM_152641 | ARID2 | circRNA | chr12 | 46245193 | 46245216 | + | 4 | 0 | AGO1-4,AGO2 | 8mer | ucgcCCUGAAACUC--CCGGUCAa | agguGGUCUAUCAGGUGGCCAGUa |
| NM_030674 | SLC38A1 | circRNA | chr12 | 46590933 | 46590954 | - | 1 | 0 | AGO1-4 | 7mer-m8 | ucgcccugaaacUCCCGGUCAa | cugauccagggcAUGGCCAGUc |
| NM_005480 | TROAP | circRNA | chr12 | 49723966 | 49723989 | + | 1 | 0 | AGO1-4 | 7mer-m8 | ucgCCCUGA--AACUCCCGGUCAa | agaGGGGCUGGUAGGGGGCCAGUg |
| NM_023071 | SPATS2 | circRNA | chr12 | 49838581 | 49838604 | + | 2 | 0 | AGO1-4 | 7mer-m8 | ucgcccUGAAACU--CCCGGUCAa | uucaccAUGUUGGCCAGGCCAGUc |
| NM_006337 | MCRS1 | circRNA | chr12 | 49959368 | 49959389 | - | 2 | 0 | AGO1,AGO2 | 7mer-m8 | ucGCCCUGAAACUCCCGGUCAa | acCCGGGCAAAGGGGGCCAGUg |
| NM_006337 | MCRS1 | circRNA | chr12 | 49960665 | 49960689 | - | 1 | 0 | AGO2 | 7mer-m8 | ucgcccugaaACUC---CCGGUCAa | uauaaggaagUGAGGCUGGCCAGUu |
| NM_001174126 | SLC11A2 | circRNA | chr12 | 51385405 | 51385425 | - | 4 | 0 | AGO1-4 | 8mer | ucgccCUGAAACUCCCGGUCAa | uuuacGAGCUUG-CGGCCAGUa |
| NM_001174129 | SLC11A2 | circRNA | chr12 | 51385405 | 51385425 | - | 4 | 0 | AGO1-4 | 8mer | ucgccCUGAAACUCCCGGUCAa | uuuacGAGCUUG-CGGCCAGUa |
| NM_001174126 | SLC11A2 | circRNA | chr12 | 51386603 | 51386626 | - | 6 | 0 | AGO1,AGO2 | 7mer-m8 | ucGCCCU--GAAACUCCCGGUCAa | gaCAGGAACCUAUUCUGGCCAGUu |
| NM_001174129 | SLC11A2 | circRNA | chr12 | 51386603 | 51386626 | - | 6 | 0 | AGO1,AGO2 | 7mer-m8 | ucGCCCU--GAAACUCCCGGUCAa | gaCAGGAACCUAUUCUGGCCAGUu |
| NM_000020 | ACVRL1 | circRNA | chr12 | 52315320 | 52315341 | + | 3 | 0 | AGO1-4 | 7mer-m8 | ucgcccugaaacucCCGGUCAa | guggcaggaucacaGGCCAGUg |
| NM_001417 | EIF4B | circRNA | chr12 | 53435597 | 53435619 | + | 4 | 0 | AGO1,AGO1-4 | 8mer | ucgccCUGAAACU-CCCGGUCAa | uaauuGGUGUGGAUUGGCCAGUa |
| NM_012291 | ESPL1 | circRNA | chr12 | 53681984 | 53682005 | + | 1 | 0 | AGO1-4 | 7mer-m8 | ucgcccugaaaCUCCCGGUCAa | cggcguccccaGAGGGCCAGUg |
| NM_001127322 | CBX5 | circRNA | chr12 | 54632852 | 54632872 | - | 5 | 0 | AGO1-4,AGO2 | 8mer | ucgCCCUGAAACUCCCGGUCAa | guaGGGAGCCAGA-GGCCAGUa |
| NM_002205 | ITGA5 | circRNA | chr12 | 54801918 | 54801939 | - | 1 | 0 | AGO1-4 | 7mer-m8 | ucgccCUGAAACUCCCGGUCAa | cugcaGACUCGCCAGGCCAGUu |
| NR_037656 | BLOC1S1 | circRNA | chr12 | 56112949 | 56112970 | + | 2 | 2 | AGO2 | 7mer-m8 | ucgcccugaaacucCCGGUCAa | uugccaagcagacaGGCCAGUg |
| NM_001184796 | ESYT1 | circRNA | chr12 | 56524588 | 56524609 | + | 2 | 0 | AGO1-4 | 8mer | ucGCCCUGAAACUCCCGGUCAa | ucUGGCCCUUCCUGGGCCAGUa |
| NM_001184796 | ESYT1 | circRNA | chr12 | 56537936 | 56537958 | + | 7 | 0 | AGO1-4,AGO2 | 7mer-m8 | ucgcccuGAAAC-UCCCGGUCAa | ucuguauCUGUGCCUGGCCAGUg |
| NM_015292 | ESYT1 | circRNA | chr12 | 56537936 | 56537958 | + | 7 | 0 | AGO1-4,AGO2 | 7mer-m8 | ucgcccuGAAAC-UCCCGGUCAa | ucuguauCUGUGCCUGGCCAGUg |
| NM_001184796 | ESYT1 | circRNA | chr12 | 56538184 | 56538211 | + | 7 | 3 | AGO1-4,AGO2 | 7mer-m8 | ucgcccUGAAACU------CCCGGUCAa | ucuacuGCUUUGAUGGCUGGGGCCAGUc |
| NM_015292 | ESYT1 | circRNA | chr12 | 56538184 | 56538211 | + | 7 | 3 | AGO1-4,AGO2 | 7mer-m8 | ucgcccUGAAACU------CCCGGUCAa | ucuacuGCUUUGAUGGCUGGGGCCAGUc |
| NM_006601 | PTGES3 | circRNA | chr12 | 57065564 | 57065585 | - | 5 | 0 | AGO1-4,AGO2 | 7mer-m8 | ucgcccugaaacucCCGGUCAa | gaaaaggagaaucuGGCCAGUc |
| NM_001166358 | SHMT2 | circRNA | chr12 | 57628187 | 57628208 | + | 9 | 0 | AGO1,AGO1-4,AGO2 | 7mer-m8 | ucgcccugaaacuCCCGGUCAa | cuucccccuaccuGGGCCAGUg |
| NM_020762 | SRGAP1 | circRNA | chr12 | 64521412 | 64521433 | + | 4 | 0 | AGO1-4 | 7mer-m8 | ucgcccugaaaCUCCCGGUCAa | ugaggccagcaGUGGGCCAGUc |
| NM_152440 | C12orf66 | circRNA | chr12 | 64587886 | 64587907 | - | 1 | 0 | AGO1-4 | 7mer-m8 | ucgcccugaaacucCCGGUCAa | ucugcccagcgacaGGCCAGUc |
| NM_006482 | DYRK2 | circRNA | chr12 | 68056296 | 68056323 | + | 20 | 0 | AGO1,AGO1-4,AGO2,AGO3 | 7mer-m8 | ucgcCCUGAAA-----CUC-CCGGUCAa | uaguGGAUUUUAAUCCUAGUGGCCAGUu |
| NM_002392 | MDM2 | circRNA | chr12 | 69210593 | 69210614 | + | 35 | 0 | AGO1,AGO1-4,AGO2,AGO3,AGO4 | 8mer | ucgcccugaaacucCCGGUCAa | uucuuuuuuaucuuGGCCAGUa |
| NM_001874 | CPM | circRNA | chr12 | 69264019 | 69264040 | - | 1 | 0 | AGO2 | 7mer-m8 | ucGCCCUGAAACUCCCGGUCAa | ggUGGUGCCCUCGUGGCCAGUu |
| NM_007007 | CPSF6 | circRNA | chr12 | 69646907 | 69646928 | + | 3 | 0 | AGO1,AGO2 | 7mer-m8 | ucgcccugaaacUCCCGGUCAa | aaaaucgggcaaAUGGCCAGUc |
| hsa_circ_0000417 | hsa_circ_001472 | circRNA | chr12 | 69646907 | 69646928 | + | 3 | 0 | AGO1,AGO2 | 7mer-m8 | ucgcccugaaacUCCCGGUCAa | aaaaucgggcaaAUGGCCAGUc |
| NM_144982 | ZFC3H1 | circRNA | chr12 | 72026707 | 72026730 | - | 2 | 0 | AGO1-4,AGO2 | 8mer | ucGCCC-UGAAACUC-CCGGUCAa | cuCGGGCAAAGGCAGUGGCCAGUa |
| NM_014903 | NAV3 | circRNA | chr12 | 78604792 | 78604814 | + | 7 | 0 | AGO1-4,AGO2 | 7mer-m8 | ucgcccugaaacUC-CCGGUCAa | agaacaaauccaAGAGGCCAGUc |
| NM_002480 | PPP1R12A | circRNA | chr12 | 80190707 | 80190728 | - | 2 | 0 | AGO1-4,AGO2 | 8mer | ucgcccUGAAACUCCCGGUCAa | uuaccaGCGUUAUAGGCCAGUa |
| NM_152588 | TMTC2 | circRNA | chr12 | 83251118 | 83251141 | + | 2 | 0 | AGO1-4,AGO2 | 7mer-m8 | ucGCCCUG--AAACUCCCGGUCAa | gaCGAGCCGAUGUCGGGGCCAGUc |
| NM_172240 | POC1B | circRNA | chr12 | 89885818 | 89885839 | - | 1 | 0 | AGO1-4 | 7mer-m8 | ucgcccugaaACUCCCGGUCAa | acuuuucagcUGAUGGCCAGUu |
| NM_024312 | GNPTAB | circRNA | chr12 | 102151014 | 102151035 | - | 1 | 0 | AGO2 | 7mer-m8 | ucgcccUGAAACUCCCGGUCAa | uuucucAUGUGGUUGGCCAGUu |
| NM_024312 | GNPTAB | circRNA | chr12 | 102164911 | 102164933 | - | 1 | 0 | AGO1-4 | 7mer-m8 | ucgcccugaAACUC-CCGGUCAa | gcaguuguaUUCAGAGGCCAGUg |
| NM_001031701 | NT5DC3 | circRNA | chr12 | 104166801 | 104166822 | - | 4 | 0 | AGO1-4,AGO2 | 7mer-m8 | ucgcccUGAAACUCCCGGUCAa | auaguuACAUGUAUGGCCAGUg |
| NM_001031701 | NT5DC3 | circRNA | chr12 | 104171097 | 104171118 | - | 1 | 0 | AGO1-4 | 7mer-m8 | ucgcccugaaACUCCCGGUCAa | guuccccaggUGAUGGCCAGUu |
| NM_003299 | HSP90B1 | circRNA | chr12 | 104327861 | 104327882 | + | 12 | 0 | AGO1,AGO1-4 | 7mer-m8 | ucgcccugaaaCUCCCGGUCAa | aagcacaggaaGAUGGCCAGUc |
| NM_003299 | HSP90B1 | circRNA | chr12 | 104327884 | 104327906 | + | 17 | 0 | AGO1,AGO1-4,AGO2 | 7mer-m8 | ucgcccugaAACU-CCCGGUCAa | acuucugaaUUGAUUGGCCAGUu |
| NM_014706 | SART3 | circRNA | chr12 | 108939069 | 108939090 | - | 1 | 0 | AGO2 | 8mer | ucgcccugaaacUCCCGGUCAa | uuuggcuagaguAUGGCCAGUa |
| NM_014325 | CORO1C | circRNA | chr12 | 109042383 | 109042404 | - | 8 | 0 | AGO1-4,AGO2 | 7mer-m8 | ucgcccugaaacUCCCGGUCAa | aaaaccacagacACGGCCAGUg |
| NM_014325 | CORO1C | circRNA | chr12 | 109097504 | 109097525 | - | 1 | 0 | AGO1-4 | 8mer | ucgCCCUGAAACUCCCGGUCAa | cugGGGAGAGGGCAGGCCAGUa |
| NM_021625 | TRPV4 | circRNA | chr12 | 110234420 | 110234439 | - | 1 | 0 | AGO1-4 | 7mer-m8 | ucGCCCUGAAACUCCCGGUCAa | acUGGGCCUAU--GGGCCAGUg |
| NM_033121 | ANKRD13A | circRNA | chr12 | 110476908 | 110476927 | + | 3 | 0 | AGO2 | 8mer | ucGCCCUGAAACUCCCGGUCAa | uuCUGCAC-AUGA-GGCCAGUa |
| NM_170665 | ATP2A2 | circRNA | chr12 | 110786233 | 110786254 | + | 1 | 0 | AGO1-4 | 8mer | ucgcccUGAAACUCCCGGUCAa | cugguuACUGGGAUGGCCAGUa |
| NM_001082538 | TCTN1 | circRNA | chr12 | 111064722 | 111064746 | + | 1 | 0 | AGO1-4 | 7mer-m8 | ucGCCC--UGAAAC-UCCCGGUCAa | caUGGGCCACCAUGCCUGGCCAGUu |
| NM_002973 | ATXN2 | circRNA | chr12 | 111894018 | 111894039 | - | 1 | 0 | AGO1-4 | 7mer-m8 | ucgcccugaaacucCCGGUCAa | caggcucuccaucuGGCCAGUc |
| NM_000690 | ALDH2 | circRNA | chr12 | 112230435 | 112230456 | + | 4 | 0 | AGO1-4,AGO2 | 7mer-m8 | ucgcccugaaacUCCCGGUCAa | uguucuucaaccAGGGCCAGUg |
| NM_001143906 | TRAFD1 | circRNA | chr12 | 112591068 | 112591089 | + | 6 | 0 | AGO1-4,AGO2 | 7mer-m8 | ucgcccugaaacucCCGGUCAa | ugguaaggugcucaGGCCAGUu |
| NM_001109662 | C12orf51 | circRNA | chr12 | 112650309 | 112650330 | - | 1 | 0 | AGO2 | 7mer-m8 | ucgcccugaaacucCCGGUCAa | cucugguggcauguGGCCAGUg |
| NM_015335 | MED13L | circRNA | chr12 | 116664270 | 116664290 | - | 5 | 0 | AGO1-4,AGO2 | 8mer | ucgcccuGAAACUCCCGGUCAa | ugaauauCAUU-AUGGCCAGUa |
| NM_002442 | MSI1 | circRNA | chr12 | 120806049 | 120806070 | - | 2 | 0 | AGO2 | 7mer-m8 | ucGCCCUGAAACUCCCGGUCAa | ugCGCGAAUACUUCGGCCAGUu |
| NM_001080533 | UNC119B | circRNA | chr12 | 121157813 | 121157834 | + | 5 | 0 | AGO1-4,AGO2 | 8mer | ucgcccugaaACUCCCGGUCAa | augccuauaaUGGAGGCCAGUa |
| NM_001080533 | UNC119B | circRNA | chr12 | 121160962 | 121160984 | + | 9 | 0 | AGO1-4,AGO2 | 7mer-m8 | ucgcccuGAA-ACUCCCGGUCAa | ucacuuuCUUCUGAUGGCCAGUg |
| NM_001080825 | TMEM120B | circRNA | chr12 | 122213877 | 122213900 | + | 3 | 0 | AGO1-4 | 7mer-m8 | ucgCCCUGAAACU--CCCGGUCAa | aagGUGGCUGAGGCCGGGCCAGUc |
| NM_001247997 | CLIP1 | circRNA | chr12 | 122862333 | 122862357 | - | 3 | 0 | AGO1-4 | 7mer-m8 | ucgcccUGAAAC-U--CCCGGUCAa | aaacccAGUUUGCACCAGGCCAGUg |
| hsa_circ_0000456 | hsa_circ_000811 | circRNA | chr12 | 123336746 | 123336767 | + | 1 | 0 | AGO2 | 7mer-m8 | ucgcccugaaacucCCGGUCAa | gaguccccaggucuGGCCAGUg |
| NM_020845 | PITPNM2 | circRNA | chr12 | 123468866 | 123468886 | - | 4 | 0 | AGO1-4,AGO2 | 7mer-m8 | ucgCCCUGAAACUCCCGGUCAa | ucaGGGGCCCUG-UGGCCAGUg |
| NM_020845 | PITPNM2 | circRNA | chr12 | 123469888 | 123469909 | - | 1 | 0 | AGO2 | 7mer-m8 | ucgcccugaaacucCCGGUCAa | ccgccuggagcccaGGCCAGUg |
| NM_012463 | ATP6V0A2 | circRNA | chr12 | 124244363 | 124244387 | + | 4 | 0 | AGO1-4,AGO2 | 7mer-m8 | ucGCCCUGAAA---CUCCCGGUCAa | gaUGAGAUGUUACCCAAGGCCAGUu |
| NM_006231 | POLE | circRNA | chr12 | 133219249 | 133219275 | - | 3 | 0 | AGO1-4,AGO2 | 7mer-m8 | ucGCCCU-GA-AACUC---CCGGUCAa | gcUGGGAGCUGAAGAGGCUGGCCAGUg |
| NM_005895 | GOLGA3 | circRNA | chr12 | 133384582 | 133384603 | - | 2 | 0 | AGO1-4 | 7mer-m8 | ucgcCCUGAAACUCCCGGUCAa | cugcGGAUACCCUGGGCCAGUu |
| NM_005895 | GOLGA3 | circRNA | chr12 | 133385048 | 133385071 | - | 4 | 0 | AGO1-4 | 8mer | ucgcccUGAA--ACUCCCGGUCAa | cagaaaACUUACCAAGGGCCAGUa |
| TCONS_l2_00007055 | TCONS_l2_00007055 | circRNA | chr13 | 20323201 | 20323223 | - | 1 | 0 | AGO1-4 | 7mer-m8 | ucgcccUGAAAC-UCCCGGUCAa | ugagccACUGUGCCUGGCCAGUu |
| NM_003453 | ZMYM2 | circRNA | chr13 | 20605472 | 20605493 | + | 10 | 0 | AGO1,AGO1-4,AGO2 | 7mer-m8 | ucgcccugaaacUCCCGGUCAa | agucaucuccaaAUGGCCAGUu |
| NM_022459 | XPO4 | circRNA | chr13 | 21383274 | 21383295 | - | 2 | 0 | AGO1-4,AGO2 | 7mer-m8 | ucgcccugaaacucCCGGUCAa | uuuucugaucaacuGGCCAGUg |
| NM_153251 | ZDHHC20 | circRNA | chr13 | 22009636 | 22009657 | - | 1 | 0 | AGO1-4 | 8mer | ucgcccugaaacucCCGGUCAa | uuugucccagauuuGGCCAGUa |
| NM_175854 | PAN3 | circRNA | chr13 | 28719316 | 28719337 | + | 1 | 0 | AGO2 | 8mer | ucgcccUGAAACUCCCGGUCAa | uaguacAGUAUCAUGGCCAGUa |
| NM_003045 | SLC7A1 | circRNA | chr13 | 30087838 | 30087859 | - | 14 | 0 | AGO1-4,AGO2 | 7mer-m8 | ucgcccugaaacuCCCGGUCAa | ggccuccaggccuGGGCCAGUu |
| NM_003045 | SLC7A1 | circRNA | chr13 | 30091884 | 30091905 | - | 3 | 0 | AGO1-4,AGO2 | 8mer | ucgcccugaaaCUCCCGGUCAa | cugguauaccaGAUGGCCAGUa |
| NM_000059 | BRCA2 | circRNA | chr13 | 32912586 | 32912607 | + | 3 | 0 | AGO2 | 7mer-m8 | ucgcccugaaacucCCGGUCAa | gucuuaaauuaucuGGCCAGUu |
| NM_000059 | BRCA2 | circRNA | chr13 | 32972830 | 32972851 | + | 2 | 0 | AGO1-4,AGO2 | 8mer | ucgCCCUGAAACUCCCGGUCAa | cagGAGAGUUCCCAGGCCAGUa |
| NM_005780 | LHFP | circRNA | chr13 | 39952573 | 39952592 | - | 2 | 0 | AGO1,AGO2 | 7mer-m8 | ucgcccUGAAACUCCCGGUCAa | ggcuacACUU--CUGGCCAGUu |
| NM_031431 | COG3 | circRNA | chr13 | 46099117 | 46099138 | + | 2 | 0 | AGO1,AGO2 | 7mer-m8 | ucgcccugaaacUCCCGGUCAa | gcguuaaaaacaAUGGCCAGUc |
| NM_052950 | WDFY2 | circRNA | chr13 | 52334053 | 52334070 | + | 5 | 0 | AGO1-4,AGO2 | 7mer-m8 | ucgCCCUGAAACUCCCGGUCAa | gugGGGACCU----GGCCAGUg |
| NM_001004127 | ALG11 | circRNA | chr13 | 52598771 | 52598792 | + | 3 | 0 | AGO1-4 | 7mer-m8 | ucGCCCUGAAACUCCCGGUCAa | ugCUGGUUUCUGUUGGCCAGUu |
| hsa_circ_0000492 | hsa_circ_001561 | circRNA | chr13 | 67801820 | 67801841 | - | 1 | 0 | AGO2 | 7mer-m8 | ucgcccugaaacucCCGGUCAa | uguaaaugacaacaGGCCAGUg |
| NM_005358 | LMO7 | circRNA | chr13 | 76246809 | 76246830 | + | 1 | 0 | AGO1-4 | 7mer-m8 | ucgcccugaaacucCCGGUCAa | cacucuagaugcuuGGCCAGUu |
| NM_002271 | IPO5 | circRNA | chr13 | 98634764 | 98634785 | + | 6 | 0 | AGO1,AGO1-4,AGO2 | 7mer-m8 | ucgcccugaaacucCCGGUCAa | augagaauaucccaGGCCAGUc |
| NM_001130048 | DOCK9 | circRNA | chr13 | 99538849 | 99538870 | - | 1 | 0 | AGO2 | 7mer-m8 | ucgccCUGAAACUCCCGGUCAa | uggcaGACCUGGUGGGCCAGUu |
| NM_003749 | IRS2 | circRNA | chr13 | 110408330 | 110408350 | - | 2 | 0 | AGO1-4,AGO2 | 7mer-m8 | ucgcccugaAACUCCCGGUCAa | cuucuuaaaUUG-UGGCCAGUg |
| NM_001846 | COL4A2 | circRNA | chr13 | 111077081 | 111077098 | + | 1 | 0 | AGO2 | 7mer-m8 | ucgcccugaaacuCCCGGUCAa | #NAME? |
| NM_017664 | ANKRD10 | circRNA | chr13 | 111532342 | 111532363 | - | 2 | 0 | AGO1-4,AGO2 | 7mer-m8 | ucgcccugaaacUCCCGGUCAa | uggaaagcaggaAUGGCCAGUg |
| NM_001113511 | ARHGEF7 | circRNA | chr13 | 111862318 | 111862339 | + | 5 | 0 | AGO1-4,AGO2 | 8mer | ucgcccugAAACUCCCGGUCAa | caaaacugUUCCAGGGCCAGUa |
| NM_003899 | ARHGEF7 | circRNA | chr13 | 111862318 | 111862339 | + | 5 | 0 | AGO1-4,AGO2 | 8mer | ucgcccugAAACUCCCGGUCAa | caaaacugUUCCAGGGCCAGUa |
| hsa_circ_0000503 | hsa_circ_001050 | circRNA | chr13 | 111862318 | 111862339 | + | 5 | 0 | AGO1-4,AGO2 | 8mer | ucgcccugAAACUCCCGGUCAa | caaaacugUUCCAGGGCCAGUa |
| NM_001113511 | ARHGEF7 | circRNA | chr13 | 111951501 | 111951522 | + | 1 | 0 | AGO1-4 | 7mer-m8 | ucgcccugaaacucCCGGUCAa | aaggggcagcaccuGGCCAGUg |
| NM_005561 | LAMP1 | circRNA | chr13 | 113976013 | 113976034 | + | 8 | 0 | AGO1,AGO1-4,AGO2 | 7mer-m8 | ucgcccugaaaCUCCCGGUCAa | ucaagguggaaGGUGGCCAGUu |
| NM_024658 | IPO4 | circRNA | chr14 | 24655575 | 24655596 | - | 1 | 1 | AGO2 | 7mer-m8 | ucgcccugaaacucCCGGUCAa | cugagcccccaccaGGCCAGUu |
| NM_004581 | RABGGTA | circRNA | chr14 | 24735708 | 24735723 | - | 1 | 0 | AGO1-4 | 7mer-m8 | ucgcccugaaACUCCCGGUCAa | #NAME? |
| NM_004554 | NFATC4 | circRNA | chr14 | 24845216 | 24845237 | + | 2 | 0 | AGO1-4 | 7mer-m8 | ucgCCCUGAAACUCCCGGUCAa | caaGAGGGUUUCCCGGCCAGUc |
| NM_002742 | PRKD1 | circRNA | chr14 | 30194781 | 30194803 | - | 5 | 0 | AGO1,AGO1-4,AGO2 | 7mer-m8 | ucgcccugaaacUC-CCGGUCAa | ucagcuggugaaAGCGGCCAGUg |
| NM_015382 | HECTD1 | circRNA | chr14 | 31598397 | 31598419 | - | 8 | 0 | AGO1-4,AGO2 | 8mer | ucGCCCUGAAACUC-CCGGUCAa | uuCUGUGUGUAGCGUGGCCAGUa |
| NM_015473 | HEATR5A | circRNA | chr14 | 31771641 | 31771662 | - | 1 | 0 | AGO2 | 7mer-m8 | ucgcccugaaACUCCCGGUCAa | ugaaguuaccUGGGGGCCAGUu |
| NM_001663 | ARF6 | circRNA | chr14 | 50360546 | 50360567 | + | 15 | 0 | AGO1-4,AGO2 | 7mer-m8 | ucgcccugaaacuCCCGGUCAa | acaaguugaagcuGGGCCAGUc |
| NM_007361 | NID2 | circRNA | chr14 | 52472529 | 52472549 | - | 3 | 0 | AGO1-4 | 7mer-m8 | ucgcccUGAAACUCCCGGUCAa | aaauaaACAUAG-UGGCCAGUu |
| NM_015589 | SAMD4A | circRNA | chr14 | 55167345 | 55167367 | + | 1 | 0 | AGO1-4 | 7mer-m8 | ucgCCCUG-AAACUCCCGGUCAa | guaGGGUCAUCAGUGGGCCAGUg |
| NM_014924 | ATG14 | circRNA | chr14 | 55836060 | 55836085 | - | 2 | 0 | AGO1-4 | 7mer-m8 | ucgcCCU-GAAACUC---CCGGUCAa | aggaGGACCUGUGCGUGUGGCCAGUu |
| NM_018229 | MUDENG | circRNA | chr14 | 57741157 | 57741175 | + | 5 | 0 | AGO1-4,AGO2 | 7mer-m8 | ucgcCCUGAAACUCCCGGUCAa | ugaaGAACUCU---GGCCAGUu |
| NM_017420 | SIX4 | circRNA | chr14 | 61177468 | 61177489 | - | 12 | 0 | AGO1-4,AGO2 | 7mer-m8 | ucgcccugaaACUCCCGGUCAa | uucaguugaaUAAGGGCCAGUu |
| NM_006255 | PRKCH | circRNA | chr14 | 61788660 | 61788681 | + | 1 | 0 | AGO2 | 7mer-m8 | ucgcccugaaacUCCCGGUCAa | ggaggaggcagaAUGGCCAGUc |
| NM_005956 | MTHFD1 | circRNA | chr14 | 64906851 | 64906871 | + | 2 | 0 | AGO1-4 | 7mer-m8 | ucgcccuGAAACUCCCGGUCAa | uugauauCUCUG-UGGCCAGUg |
| NM_005956 | MTHFD1 | circRNA | chr14 | 64926495 | 64926521 | + | 3 | 0 | AGO1-4 | 7mer-m8 | ucgcccUGAAAC--UC---CCGGUCAa | gaagcuACUUUGAAAGUCUGGCCAGUg |
| NM_022474 | MPP5 | circRNA | chr14 | 67768759 | 67768780 | + | 2 | 0 | AGO1-4 | 8mer | ucgcccugaaacucCCGGUCAa | uuuaugaaaguauuGGCCAGUa |
| NM_015994 | ATP6V1D | circRNA | chr14 | 67826477 | 67826498 | - | 3 | 0 | AGO1-4,AGO2 | 7mer-m8 | ucgcccUGAAACUCCCGGUCAa | gaagacACUGUGGAGGCCAGUu |
| NM_015346 | ZFYVE26 | circRNA | chr14 | 68214919 | 68214940 | - | 19 | 0 | AGO1-4,AGO2 | 7mer-m8 | ucgcccugaaaCUCCCGGUCAa | guuugucuggaGAUGGCCAGUu |
| hsa_circ_0000547 | hsa_circ_001250 | circRNA | chr14 | 68391601 | 68391622 | + | 2 | 0 | AGO1-4 | 7mer-m8 | ucgcccugaaacUCCCGGUCAa | cucaucagagacAAGGCCAGUc |
| hsa_circ_0000547 | hsa_circ_001250 | circRNA | chr14 | 68416772 | 68416798 | + | 1 | 0 | AGO1-4 | 7mer-m8 | ucgcCCUGAAA--CUC---CCGGUCAa | ccuaGGAUUUUCAGAGAAUGGCCAGUg |
| NM_001130004 | ACTN1 | circRNA | chr14 | 69360374 | 69360400 | - | 9 | 0 | AGO1-4,AGO2 | 7mer-m8 | ucgccCUGAAACUC-----CCGGUCAa | uggaaGACUACGAGAAGCUGGCCAGUg |
| NM_014734 | KIAA0247 | circRNA | chr14 | 70175621 | 70175642 | + | 1 | 0 | AGO1-4 | 7mer-m8 | ucgcccugaaacucCCGGUCAa | aagaugaggccccaGGCCAGUc |
| hsa_circ_0000552 | hsa_circ_000594 | circRNA | chr14 | 71899205 | 71899225 | + | 1 | 0 | AGO1-4 | 8mer | ucgcccuGAAACUCCCGGUCAa | auaauuuCUUU-AUGGCCAGUa |
| hsa_circ_0006783 | hsa_circ_0006783 | circRNA | chr14 | 71899205 | 71899225 | + | 1 | 0 | AGO1-4 | 8mer | ucgcccuGAAACUCCCGGUCAa | auaauuuCUUU-AUGGCCAGUa |
| hsa_circ_0000552 | hsa_circ_000594 | circRNA | chr14 | 71902624 | 71902645 | + | 1 | 0 | AGO1-4 | 7mer-m8 | ucgcccuGAAACUCCCGGUCAa | ugcauuuCUCUGAUGGCCAGUg |
| hsa_circ_0006783 | hsa_circ_0006783 | circRNA | chr14 | 71902624 | 71902645 | + | 1 | 0 | AGO1-4 | 7mer-m8 | ucgcccuGAAACUCCCGGUCAa | ugcauuuCUCUGAUGGCCAGUg |
| NM_021260 | ZFYVE1 | circRNA | chr14 | 73441560 | 73441581 | - | 1 | 0 | AGO1-4 | 7mer-m8 | ucgcccugaaacucCCGGUCAa | uucaucaaagacucGGCCAGUg |
| NM_000021 | PSEN1 | circRNA | chr14 | 73686680 | 73686701 | + | 5 | 0 | AGO2 | 7mer-m8 | ucgcccugaaacUCCCGGUCAa | ccauuucuucccAAGGCCAGUc |
| NM_001005743 | NUMB | circRNA | chr14 | 73822399 | 73822421 | - | 2 | 0 | AGO1-4 | 7mer-m8 | ucgcccugAAACUC-CCGGUCAa | uguuuaugUUCCAGAGGCCAGUc |
| hsa_circ_0000553 | hsa_circ_001753 | circRNA | chr14 | 73822399 | 73822421 | - | 2 | 0 | AGO1-4 | 7mer-m8 | ucgcccugAAACUC-CCGGUCAa | uguuuaugUUCCAGAGGCCAGUc |
| NM_152444 | PTGR2 | circRNA | chr14 | 74353762 | 74353784 | + | 1 | 0 | AGO2 | 7mer-m8 | ucGCCCUGAAACUC-CCGGUCAa | ggCGGGCCCGGGAGCGGCCAGUc |
| NM_000428 | LTBP2 | circRNA | chr14 | 75022305 | 75022326 | - | 1 | 0 | AGO1-4 | 8mer | ucgccCUGAAACUCCCGGUCAa | cacccGACUGCCACGGCCAGUa |
| NM_001039479 | KIAA0317 | circRNA | chr14 | 75128196 | 75128214 | - | 14 | 0 | AGO1,AGO2,AGO3 | 7mer-m8 | ucgcccUGAAACUCCCGGUCAa | uuucuuAUUUU---GGCCAGUu |
| NM_001039479 | KIAA0317 | circRNA | chr14 | 75134201 | 75134222 | - | 1 | 0 | AGO2 | 7mer-m8 | ucgcccugaaacucCCGGUCAa | gcccaauaucggcuGGCCAGUc |
| NM_001040108 | MLH3 | circRNA | chr14 | 75516043 | 75516064 | - | 2 | 0 | AGO1-4 | 7mer-m8 | ucgcccUGAAACUCCCGGUCAa | aauauuGCUGACAUGGCCAGUg |
| NM_183387 | EML5 | circRNA | chr14 | 89128106 | 89128124 | - | 1 | 0 | AGO1-4 | 8mer | ucgcCCUGAAACUCCCGGUCAa | ugaaGGAAUUU---GGCCAGUa |
| hsa_circ_0000559 | hsa_circ_001754 | circRNA | chr14 | 89749362 | 89749383 | - | 1 | 0 | AGO2 | 7mer-m8 | ucgCCCUGAAACUCCCGGUCAa | aaaGAGAAGGUGGUGGCCAGUc |
| NM_018319 | TDP1 | circRNA | chr14 | 90488780 | 90488801 | + | 1 | 0 | AGO1-4 | 7mer-m8 | ucgcccugaaACUCCCGGUCAa | gcagcccaggUGCUGGCCAGUc |
| NM_006329 | FBLN5 | circRNA | chr14 | 92361299 | 92361322 | - | 1 | 0 | AGO1-4 | 7mer-m8 | ucgccCUGAA-A-CUCCCGGUCAa | auauuGGCUUCUGGAAGGCCAGUg |
| NM_004239 | TRIP11 | circRNA | chr14 | 92505986 | 92506003 | - | 2 | 0 | AGO1-4,AGO2 | 7mer-m8 | ucGCCCUGAAACUCCCGGUCAa | cuCCGGAUU----GGGCCAGUc |
| NM_017437 | CPSF2 | circRNA | chr14 | 92624099 | 92624121 | + | 2 | 0 | AGO1-4 | 7mer-m8 | ucgcccugaaacUC-CCGGUCAa | ccauggcccaccAGAGGCCAGUc |
| NM_177438 | DICER1 | circRNA | chr14 | 95562372 | 95562393 | - | 6 | 0 | AGO1-4,AGO2 | 7mer-m8 | ucgcccUGAAACUCCCGGUCAa | gcugcuGCUUCUGUGGCCAGUu |
| NM_138576 | BCL11B | circRNA | chr14 | 99724053 | 99724074 | - | 3 | 0 | AGO1-4 | 7mer-m8 | ucgcccugaaacucCCGGUCAa | accugcucaccuguGGCCAGUg |
| NM_001376 | DYNC1H1 | circRNA | chr14 | 102516512 | 102516533 | + | 4 | 0 | AGO1-4 | 7mer-m8 | ucgcccugaaaCUCCCGGUCAa | aacaccgagaaGAAGGCCAGUg |
| NM_001017963 | HSP90AA1 | circRNA | chr14 | 102552223 | 102552245 | - | 21 | 0 | AGO1,AGO1-4,AGO2 | 7mer-m8 | ucgcccuGAAACU-CCCGGUCAa | gauaucuCUAUGAUUGGCCAGUu |
| NM_005348 | HSP90AA1 | circRNA | chr14 | 102552223 | 102552245 | - | 21 | 0 | AGO1,AGO1-4,AGO2 | 7mer-m8 | ucgcccuGAAACU-CCCGGUCAa | gauaucuCUAUGAUUGGCCAGUu |
| NM_018335 | ZNF839 | circRNA | chr14 | 102793034 | 102793055 | + | 2 | 0 | AGO1-4,AGO2 | 7mer-m8 | ucgcccugaaacUCCCGGUCAa | uuaacccaggccACGGCCAGUu |
| NM_001128918 | MARK3 | circRNA | chr14 | 103934437 | 103934458 | + | 4 | 0 | AGO1-4 | 7mer-m8 | ucgcccugaaacucCCGGUCAa | ucaacaacaguacuGGCCAGUc |
| NM_001128920 | MARK3 | circRNA | chr14 | 103934437 | 103934458 | + | 4 | 0 | AGO1-4 | 7mer-m8 | ucgcccugaaacucCCGGUCAa | ucaacaacaguacuGGCCAGUc |
| NM_032374 | APOPT1 | circRNA | chr14 | 104127717 | 104127738 | + | 1 | 0 | AGO1-4 | 7mer-m8 | ucgcccugaaACUCCCGGUCAa | uuuugaagaaUGCAGGCCAGUu |
| NM_015656 | KIF26A | circRNA | chr14 | 104642174 | 104642197 | + | 1 | 0 | AGO1-4 | 7mer-m8 | ucgCCCUGAAAC-UC-CCGGUCAa | acaGUGACCCUGCAGCGGCCAGUg |
| NM_022489 | INF2 | circRNA | chr14 | 105185421 | 105185442 | + | 1 | 0 | AGO1-4 | 7mer-m8 | ucgcccugaaacUCCCGGUCAa | ugcagccugccaAGGGCCAGUc |
| NM_138420 | AHNAK2 | circRNA | chr14 | 105403806 | 105403827 | - | 2 | 0 | AGO1-4,AGO2 | 7mer-m8 | ucgcccugaaACUCCCGGUCAa | ggcaaaagggUGAUGGCCAGUg |
| NM_002226 | JAG2 | circRNA | chr14 | 105612207 | 105612228 | - | 1 | 0 | AGO1-4 | 7mer-m8 | ucgcccugaaacucCCGGUCAa | uggagaaggccccaGGCCAGUg |
| NM_177533 | NUDT14 | circRNA | chr14 | 105639329 | 105639353 | - | 2 | 0 | AGO1-4 | 7mer-m8 | ucgCCCUGAAAC--UC-CCGGUCAa | ccaGGGUUCUAGACAGAGGCCAGUc |
| NM_001242786 | BRF1 | circRNA | chr14 | 105684095 | 105684118 | - | 2 | 0 | AGO1-4 | 8mer | ucGCCCU--G-AAACUCCCGGUCAa | gaCGGGAGCCAAUUCA-GGCCAGUa |
| NM_001519 | BRF1 | circRNA | chr14 | 105684095 | 105684118 | - | 2 | 0 | AGO1-4 | 8mer | ucGCCCU--G-AAACUCCCGGUCAa | gaCGGGAGCCAAUUCA-GGCCAGUa |
| NM_001242790 | BRF1 | circRNA | chr14 | 105766797 | 105766818 | - | 1 | 0 | AGO1-4 | 7mer-m8 | ucgcccugaaaCUCCCGGUCAa | gcuccucggccGUGGGCCAGUu |
| NM_001519 | BRF1 | circRNA | chr14 | 105766797 | 105766818 | - | 1 | 0 | AGO1-4 | 7mer-m8 | ucgcccugaaaCUCCCGGUCAa | gcuccucggccGUGGGCCAGUu |
| NM_001100913 | PACS2 | circRNA | chr14 | 105863877 | 105863898 | + | 2 | 0 | AGO1-4,AGO2 | 7mer-m8 | ucgcccugaaACUCCCGGUCAa | ccaggccagcUGUGGGCCAGUc |
| NR_002224 | ADAM6 | circRNA | chr14 | 107109763 | 107109786 | - | 1 | 0 | AGO1-4 | 7mer-m8 | ucgcccUGAA--ACUCCCGGUCAa | uugaauAUUUGCUAAAGGCCAGUu |
| NR_027457 | LINC00221 | circRNA | chr14 | 107109763 | 107109786 | - | 1 | 0 | AGO1-4 | 7mer-m8 | ucgcccUGAA--ACUCCCGGUCAa | uugaauAUUUGCUAAAGGCCAGUu |
| hsa_circ_0034021 | hsa_circ_0034021 | circRNA | chr14 | 107109763 | 107109786 | - | 1 | 0 | AGO1-4 | 7mer-m8 | ucgcccUGAA--ACUCCCGGUCAa | uugaauAUUUGCUAAAGGCCAGUu |
| NR_027992 | NBEAP1 | circRNA | chr15 | 21112982 | 21113003 | - | 1 | 0 | AGO1-4 | 7mer-m8 | ucgcccuGAAACUCCCGGUCAa | ugcauuuCUCUGAUGGCCAGUg |
| NM_144599 | NIPA1 | circRNA | chr15 | 23047101 | 23047121 | - | 1 | 0 | AGO1-4 | 7mer-m8 | ucgcCCUGAAACUCCCGGUCAa | ucauGUAC-UUCAUGGCCAGUg |
| NR_003325 | SNORD116-10 | circRNA | chr15 | 25344389 | 25344410 | + | 1 | 0 | AGO2 | 7mer-m8 | ucgcccuGAAACUCCCGGUCAa | cucccauCCUCAAGGGCCAGUg |
| NR_003340 | SNORD116-26 | circRNA | chr15 | 25344389 | 25344410 | + | 1 | 0 | AGO2 | 7mer-m8 | ucgcccuGAAACUCCCGGUCAa | cucccauCCUCAAGGGCCAGUg |
| TCONS_00023883 | TCONS_00023883 | circRNA | chr15 | 25344389 | 25344410 | + | 1 | 0 | AGO2 | 7mer-m8 | ucgcccuGAAACUCCCGGUCAa | cucccauCCUCAAGGGCCAGUg |
| hsa_circ_0034145 | hsa_circ_0034145 | circRNA | chr15 | 25344389 | 25344410 | + | 1 | 0 | AGO2 | 7mer-m8 | ucgcccuGAAACUCCCGGUCAa | cucccauCCUCAAGGGCCAGUg |
| NM_000814 | GABRB3 | circRNA | chr15 | 27184327 | 27184348 | - | 1 | 0 | AGO1-4 | 7mer-m8 | ucgcccugaaacuCCCGGUCAa | ugugaggcccgcuGGGCCAGUc |
| NM_004667 | HERC2 | circRNA | chr15 | 28483267 | 28483289 | - | 1 | 0 | AGO2 | 8mer | ucGCCCUGAA-ACUCCCGGUCAa | caCGCGUUUUGUGUUGGCCAGUa |
| NM_130901 | OTUD7A | circRNA | chr15 | 32074817 | 32074838 | - | 1 | 0 | AGO1-4 | 8mer | ucgcccugaaacucCCGGUCAa | gucccagcgccucuGGCCAGUa |
| NM_001191323 | GREM1 | circRNA | chr15 | 33023235 | 33023256 | + | 5 | 0 | AGO1-4 | 7mer-m8 | ucGCCCUGAAACUCCCGGUCAa | acCGCUUCUGUUACGGCCAGUg |
| NM_013372 | GREM1 | circRNA | chr15 | 33023235 | 33023256 | + | 5 | 0 | AGO1-4 | 7mer-m8 | ucGCCCUGAAACUCCCGGUCAa | acCGCUUCUGUUACGGCCAGUg |
| NM_001220482 | MEIS2 | circRNA | chr15 | 37390220 | 37390241 | - | 1 | 0 | AGO1-4 | 8mer | ucgcccugaaACUCCCGGUCAa | cccaaugucaUGCCGGCCAGUa |
| NM_003246 | THBS1 | circRNA | chr15 | 39874763 | 39874784 | + | 14 | 0 | AGO1-4 | 7mer-m8 | ucgcccugaaacucCCGGUCAa | cucuccuggcaaccGGCCAGUg |
| NM_007223 | GPR176 | circRNA | chr15 | 40094358 | 40094379 | - | 1 | 0 | AGO1-4 | 7mer-m8 | ucgcccugaaACUCCCGGUCAa | gcccaugcagUGGUGGCCAGUg |
| NM_001013703 | EIF2AK4 | circRNA | chr15 | 40268936 | 40268957 | + | 1 | 0 | AGO1-4 | 7mer-m8 | ucgcccugaaacucCCGGUCAa | ucgggcgagcgcucGGCCAGUg |
| NM_001013703 | EIF2AK4 | circRNA | chr15 | 40311312 | 40311332 | + | 1 | 0 | AGO1-4 | 7mer-m8 | ucgcccUGAAACUCCCGGUCAa | ccacaaGCUCUG-GGGCCAGUu |
| NM_002225 | IVD | circRNA | chr15 | 40707440 | 40707461 | + | 1 | 0 | AGO1-4 | 7mer-m8 | ucgcccugaaacucCCGGUCAa | gccccugcaccucuGGCCAGUc |
| NM_014952 | BAHD1 | circRNA | chr15 | 40754114 | 40754135 | + | 2 | 0 | AGO1,AGO1-4 | 7mer-m8 | ucgcccugaaacuCCCGGUCAa | gcugcagaucccuGGGCCAGUu |
| NM_020990 | CKMT1B | circRNA | chr15 | 43887194 | 43887216 | + | 1 | 0 | AGO1-4 | 7mer-m8 | ucgcccugaaAC-UCCCGGUCAa | gcacaacaacUGCAUGGCCAGUc |
| NM_001015001 | CKMT1A | circRNA | chr15 | 43987026 | 43987048 | + | 1 | 0 | AGO1-4 | 7mer-m8 | ucgcccugaaAC-UCCCGGUCAa | gcacaacaacUGCAUGGCCAGUc |
| NM_016396 | CTDSPL2 | circRNA | chr15 | 44816792 | 44816813 | + | 23 | 0 | AGO1,AGO1-4,AGO2 | 8mer | ucgcccugaaacUCCCGGUCAa | auuucaacaggaAUGGCCAGUa |
| NM_025137 | SPG11 | circRNA | chr15 | 44903115 | 44903136 | - | 14 | 0 | AGO1-4 | 7mer-m8 | ucgcccugaaacucCCGGUCAa | auucccaccaaucaGGCCAGUg |
| NM_000138 | FBN1 | circRNA | chr15 | 48764825 | 48764846 | - | 2 | 0 | AGO1-4 | 7mer-m8 | ucgcccugaaacUCCCGGUCAa | aucucuguggcaAUGGCCAGUg |
| NM_000138 | FBN1 | circRNA | chr15 | 48780390 | 48780414 | - | 3 | 0 | AGO1-4 | 7mer-m8 | ucgcccuGAAAC--UC-CCGGUCAa | cugaccuCUGUGGCAGAGGCCAGUg |
| NM_000138 | FBN1 | circRNA | chr15 | 48796088 | 48796110 | - | 1 | 0 | AGO2 | 7mer-m8 | ucgcccugaaaCUC-CCGGUCAa | gguggauacaaGAGAGGCCAGUg |
| NM_017672 | TRPM7 | circRNA | chr15 | 50978940 | 50978960 | - | 1 | 0 | AGO1-4 | 7mer-m8 | ucGCCCUGAAACUCCCGGUCAa | ggCGCGUCCCUG-UGGCCAGUc |
| NM_016194 | GNB5 | circRNA | chr15 | 52425620 | 52425644 | - | 2 | 0 | AGO1-4 | 7mer-m8 | ucGCCCUGAAACUC----CCGGUCAa | ugUGGGAC-AUGCGCUCCGGCCAGUg |
| NM_001018088 | VPS13C | circRNA | chr15 | 62161721 | 62161742 | - | 2 | 0 | AGO1,AGO2 | 8mer | ucgcccugaaacUCCCGGUCAa | ggaaucguagauAUGGCCAGUa |
| NM_003922 | HERC1 | circRNA | chr15 | 63972910 | 63972934 | - | 4 | 0 | AGO1-4,AGO2 | 8mer | ucgcCCUGAAA---CUCCCGGUCAa | uguuGGAGUUUCUCGCUGGCCAGUa |
| NM_022048 | CSNK1G1 | circRNA | chr15 | 64463977 | 64463998 | - | 1 | 0 | AGO2 | 7mer-m8 | ucgcccugaaacUCCCGGUCAa | augcccccagaaGGGGCCAGUg |
| NM_025049 | PIF1 | circRNA | chr15 | 65111372 | 65111395 | - | 1 | 0 | AGO2 | 8mer | ucgcccUGAAACUC--CCGGUCAa | acagcaACCCUGAGCUGGCCAGUa |
| NM_025201 | PLEKHO2 | circRNA | chr15 | 65158247 | 65158270 | + | 2 | 0 | AGO2 | 7mer-m8 | ucgcccUGAAAC--UCCCGGUCAa | cuuccuACUCUGCUCUGGCCAGUg |
| NM_006660 | CLPX | circRNA | chr15 | 65456489 | 65456510 | - | 10 | 0 | AGO1-4,AGO2 | 7mer-m8 | ucgcCCUGAAACUCCCGGUCAa | acaaGUAUGUUGUUGGCCAGUc |
| NM_004884 | IGDCC3 | circRNA | chr15 | 65623909 | 65623931 | - | 1 | 0 | AGO2 | 7mer-m8 | ucGCCCUGAAACU-CCCGGUCAa | ugCGGGCUCAUCACAGGCCAGUg |
| NM_002755 | MAP2K1 | circRNA | chr15 | 66717855 | 66717876 | + | 1 | 0 | AGO2 | 7mer-m8 | ucgcccUGAAACUCCCGGUCAa | agacccACUUCUAGGGCCAGUg |
| NM_001008224 | UACA | circRNA | chr15 | 71018123 | 71018145 | - | 2 | 0 | AGO1-4,AGO2 | 7mer-m8 | ucgccCUGAAAC-UCCCGGUCAa | ggauuGUUUCUGCAUGGCCAGUg |
| NM_001206796 | PKM2 | circRNA | chr15 | 72499593 | 72499614 | - | 16 | 2 | AGO1,AGO1-4,AGO2 | 7mer-m8 | ucgcccugaaACUCCCGGUCAa | gaugaaauccUGGAGGCCAGUg |
| NM_001206797 | PKM2 | circRNA | chr15 | 72499593 | 72499614 | - | 16 | 2 | AGO1,AGO1-4,AGO2 | 7mer-m8 | ucgcccugaaACUCCCGGUCAa | gaugaaauccUGGAGGCCAGUg |
| NM_001206799 | PKM2 | circRNA | chr15 | 72499593 | 72499614 | - | 16 | 2 | AGO1,AGO1-4,AGO2 | 7mer-m8 | ucgcccugaaACUCCCGGUCAa | gaugaaauccUGGAGGCCAGUg |
| NM_001206796 | PKM2 | circRNA | chr15 | 72523409 | 72523430 | - | 5 | 0 | AGO1-4 | 7mer-m8 | ucgcccugaaacUCCCGGUCAa | caguccccaccaAGGGCCAGUc |
| hsa_circ_0000630 | hsa_circ_001255 | circRNA | chr15 | 73027067 | 73027088 | + | 1 | 0 | AGO1-4 | 7mer-m8 | ucgccCUGAAACUCCCGGUCAa | uuauaGAUGUGGGGGGCCAGUg |
| NR_023318 | ADPGK | circRNA | chr15 | 73047935 | 73047958 | - | 1 | 0 | AGO1-4 | 8mer | ucgcccUGAAACUC--CCGGUCAa | caguucACCUAGAGCUGGCCAGUa |
| NM_005576 | LOXL1 | circRNA | chr15 | 74239475 | 74239496 | + | 3 | 0 | AGO1,AGO1-4 | 7mer-m8 | ucgcccugaaacUCCCGGUCAa | gccgagggccacAAGGCCAGUu |
| NM_033240 | PML | circRNA | chr15 | 74315483 | 74315509 | + | 2 | 0 | AGO1-4 | 7mer-m8 | ucGCCCUGAAACUC-----CCGGUCAa | agCGCGACUACGAGGAGAUGGCCAGUc |
| NM_006465 | ARID3B | circRNA | chr15 | 74889995 | 74890014 | + | 3 | 0 | AGO1-4,AGO2 | 8mer | ucgccCUGAAACUCCCGGUCAa | ucucaGACAUU--UGGCCAGUa |
| NM_001145358 | SIN3A | circRNA | chr15 | 75704051 | 75704072 | - | 3 | 0 | AGO1-4 | 7mer-m8 | ucgcccugaaacucCCGGUCAa | caagcacauacuccGGCCAGUc |
| hsa_circ_0000636 | hsa_circ_000857 | circRNA | chr15 | 75704051 | 75704072 | - | 3 | 0 | AGO1-4 | 7mer-m8 | ucgcccugaaacucCCGGUCAa | caagcacauacuccGGCCAGUc |
| NM_002833 | PTPN9 | circRNA | chr15 | 75760911 | 75760932 | - | 5 | 0 | AGO1-4,AGO2 | 8mer | ucgcccugaaacUCCCGGUCAa | uugccacuagaaAGGGCCAGUa |
| NM_024776 | PEAK1 | circRNA | chr15 | 77472810 | 77472831 | - | 3 | 0 | AGO1-4 | 7mer-m8 | ucgcccugaaacUCCCGGUCAa | gugucagcagccAUGGCCAGUg |
| NM_014630 | ZNF592 | circRNA | chr15 | 85327251 | 85327275 | + | 3 | 0 | AGO1-4,AGO2 | 7mer-m8 | ucGCCCUGAAAC---UCCCGGUCAa | gaCGAGAGCAUGACAAAGGCCAGUg |
| NM_006738 | AKAP13 | circRNA | chr15 | 86122230 | 86122249 | + | 2 | 0 | AGO1-4,AGO2 | 7mer-m8 | ucgCCCUGAAACUCCCGGUCAa | ccaGAGAC--AGAUGGCCAGUu |
| NM_005928 | MFGE8 | circRNA | chr15 | 89449943 | 89449964 | - | 3 | 0 | AGO1-4 | 7mer-m8 | ucgcccugaaacucCCGGUCAa | ggugccagccgcuuGGCCAGUc |
| NM_001199058 | C15orf38-AP3S2 | circRNA | chr15 | 90376491 | 90376516 | - | 2 | 0 | AGO2 | 7mer-m8 | ucgccCUGAAA----CUCCCGGUCAa | ggcuuGAUUUUCUAAGGUGGCCAGUg |
| NR_023361 | AP3S2 | circRNA | chr15 | 90376491 | 90376516 | - | 2 | 0 | AGO2 | 7mer-m8 | ucgccCUGAAA----CUCCCGGUCAa | ggcuuGAUUUUCUAAGGUGGCCAGUg |
| NM_022769 | CRTC3 | circRNA | chr15 | 91150666 | 91150687 | + | 1 | 0 | AGO1-4 | 7mer-m8 | ucgcccugaaaCUCCCGGUCAa | ccuauggaggaGGGGGCCAGUc |
| NM_013272 | SLCO3A1 | circRNA | chr15 | 92414463 | 92414484 | + | 1 | 0 | AGO1-4 | 7mer-m8 | ucgcccugaaacuCCCGGUCAa | uaugaaaugucuuGGGCCAGUu |
| NR_037601 | LOC100507217 | circRNA | chr15 | 93477799 | 93477823 | + | 2 | 0 | AGO1-4 | 8mer | ucgcCCUGAA--ACUC-CCGGUCAa | auuuGCAUUUUAUAAGAGGCCAGUa |
| NM_000875 | IGF1R | circRNA | chr15 | 99467111 | 99467133 | + | 3 | 0 | AGO1-4 | 7mer-m8 | ucgccCUG-AAACUCCCGGUCAa | cagauGACAUUCCUGGGCCAGUg |
| NM_198243 | ASB7 | circRNA | chr15 | 101190379 | 101190403 | + | 2 | 0 | AGO1-4 | 7mer-m8 | ucgcccUGAAACUC---CCGGUCAa | gcagccACUCUGAGUGUGGCCAGUu |
| NM_003502 | AXIN1 | circRNA | chr16 | 339473 | 339494 | - | 1 | 0 | AGO2 | 7mer-m8 | ucgcccugaaacuCCCGGUCAa | gcgcugucacccuGGGCCAGUu |
| NM_006428 | MRPL28 | circRNA | chr16 | 417676 | 417698 | - | 1 | 1 | AGO1-4 | 7mer-m8 | ucgcccugaaacUC-CCGGUCAa | cagaagagagccAGUGGCCAGUg |
| NM_021259 | TMEM8A | circRNA | chr16 | 424809 | 424829 | - | 1 | 0 | AGO1-4 | 7mer-m8 | ucgcCCUGAAACUCCCGGUCAa | uuguGGACCCU-AUGGCCAGUg |
| hsa_circ_0000663 | hsa_circ_001482 | circRNA | chr16 | 424809 | 424829 | - | 1 | 0 | AGO1-4 | 7mer-m8 | ucgcCCUGAAACUCCCGGUCAa | uuguGGACCCU-AUGGCCAGUg |
| NM_005632 | SOLH | circRNA | chr16 | 598064 | 598085 | + | 2 | 0 | AGO2 | 7mer-m8 | ucgcccugaaacuCCCGGUCAa | ugcccgagcgcccGGGCCAGUg |
| NM_001172663 | RAB40C | circRNA | chr16 | 677678 | 677699 | + | 1 | 0 | AGO1 | 7mer-m8 | ucgcccugaaacucCCGGUCAa | cuggcuggacgccaGGCCAGUg |
| NM_032520 | GNPTG | circRNA | chr16 | 1402250 | 1402271 | + | 2 | 0 | AGO1-4,AGO2 | 7mer-m8 | ucgcccugaaacucCCGGUCAa | ccguucuugccucaGGCCAGUc |
| NM_001009944 | PKD1 | circRNA | chr16 | 2139805 | 2139828 | - | 1 | 0 | AGO1-4 | 7mer-m8 | ucgcccUGAAAC--UCCCGGUCAa | ccagccGCCUUGCCCGGGCCAGUc |
| NM_001089 | ABCA3 | circRNA | chr16 | 2376148 | 2376169 | - | 2 | 0 | AGO1-4 | 7mer-m8 | ucgcccugaaacuCCCGGUCAa | ccaccaucuacccGGGCCAGUc |
| NM_016292 | TRAP1 | circRNA | chr16 | 3727601 | 3727622 | - | 5 | 0 | AGO1-4,AGO2 | 7mer-m8 | ucgcccugaaacucCCGGUCAa | gcagcaagaucaucGGCCAGUu |
| NM_032569 | GLYR1 | circRNA | chr16 | 4861695 | 4861717 | - | 7 | 0 | AGO1,AGO1-4,AGO2 | 7mer-m8 | ucgcccugaaACU-CCCGGUCAa | cuggcccacgUGACAGGCCAGUc |
| NM_016256 | NAGPA | circRNA | chr16 | 5078897 | 5078915 | - | 3 | 0 | AGO2 | 7mer-m8 | ucGCCCUGAAACUCCCGGUCAa | aaCGGGACCUU---GGCCAGUu |
| NM_014048 | MKL2 | circRNA | chr16 | 14355804 | 14355825 | + | 1 | 0 | AGO1-4 | 7mer-m8 | ucgcccugaaacucCCGGUCAa | caguggagagccuuGGCCAGUu |
| NM_014287 | NOMO1 | circRNA | chr16 | 14946314 | 14946335 | + | 2 | 0 | AGO1-4 | 7mer-m8 | ucgcccugaaacucCCGGUCAa | uccaaugccaaugcGGCCAGUc |
| NM_004996 | ABCC1 | circRNA | chr16 | 14973864 | 14973885 | + | 1 | 0 | AGO1-4 | 8mer | ucgcccugaaacucCCGGUCAa | cuugguagagcccuGGCCAGUa |
| NM_014287 | NOMO1 | circRNA | chr16 | 14973864 | 14973885 | + | 1 | 0 | AGO1-4 | 8mer | ucgcccugaaacucCCGGUCAa | cuugguagagcccuGGCCAGUa |
| NM_004996 | ABCC1 | circRNA | chr16 | 14973872 | 14973885 | + | 1 | 0 | AGO1-4 | 8mer | ucgcccugaaacucCCGGUCAa | #NAME? |
| NM_014287 | NOMO1 | circRNA | chr16 | 14973872 | 14973885 | + | 1 | 0 | AGO1-4 | 8mer | ucgcccugaaacucCCGGUCAa | #NAME? |
| NM_004996 | ABCC1 | circRNA | chr16 | 14980658 | 14980679 | + | 4 | 1 | AGO1-4 | 7mer-m8 | ucgcccugaaacucCCGGUCAa | agacaguuucccuuGGCCAGUc |
| NM_014287 | NOMO1 | circRNA | chr16 | 14980658 | 14980679 | + | 4 | 1 | AGO1-4 | 7mer-m8 | ucgcccugaaacucCCGGUCAa | agacaguuucccuuGGCCAGUc |
| hsa_circ_0038032 | hsa_circ_0038032 | circRNA | chr16 | 15217914 | 15217939 | - | 2 | 0 | AGO1-4 | 7mer-m8 | ucgcCCUGAA---ACUC-CCGGUCAa | ccuuGGACGUCCCUGAGAGGCCAGUc |
| NM_004996 | ABCC1 | circRNA | chr16 | 15358915 | 15358936 | + | 1 | 0 | AGO1-4 | 8mer | ucGCCCUGAAACUCCCGGUCAa | uuCGAGACCAGCCUGGCCAGUa |
| NM_014287 | NOMO1 | circRNA | chr16 | 15358915 | 15358936 | + | 1 | 0 | AGO1-4 | 8mer | ucGCCCUGAAACUCCCGGUCAa | uuCGAGACCAGCCUGGCCAGUa |
| NM_004996 | ABCC1 | circRNA | chr16 | 15403158 | 15403179 | + | 1 | 0 | AGO1-4 | 7mer-m8 | ucgcccugaaacucCCGGUCAa | caaauguucugccaGGCCAGUg |
| NM_014287 | NOMO1 | circRNA | chr16 | 15403158 | 15403179 | + | 1 | 0 | AGO1-4 | 7mer-m8 | ucgcccugaaacucCCGGUCAa | caaauguucugccaGGCCAGUg |
| NM_004996 | ABCC1 | circRNA | chr16 | 15645893 | 15645914 | + | 1 | 0 | AGO1-4 | 7mer-m8 | ucgCCCUGAAACUCCCGGUCAa | uuaGGGACACCAAGGGCCAGUc |
| NM_014287 | NOMO1 | circRNA | chr16 | 15645893 | 15645914 | + | 1 | 0 | AGO1-4 | 7mer-m8 | ucgCCCUGAAACUCCCGGUCAa | uuaGGGACACCAAGGGCCAGUc |
| NM_014287 | NOMO1 | circRNA | chr16 | 16056987 | 16057008 | + | 1 | 0 | AGO1-4 | 7mer-m8 | ucgcccugaaacucCCGGUCAa | ucugaaauccaauaGGCCAGUc |
| NM_001004067 | NOMO3 | circRNA | chr16 | 16344812 | 16344833 | + | 2 | 0 | AGO1-4 | 7mer-m8 | ucgcccugaaacucCCGGUCAa | uccaaugccaaugcGGCCAGUc |
| NM_004996 | ABCC1 | circRNA | chr16 | 16344812 | 16344833 | + | 2 | 0 | AGO1-4 | 7mer-m8 | ucgcccugaaacucCCGGUCAa | uccaaugccaaugcGGCCAGUc |
| NM_014287 | NOMO1 | circRNA | chr16 | 16344812 | 16344833 | + | 2 | 0 | AGO1-4 | 7mer-m8 | ucgcccugaaacucCCGGUCAa | uccaaugccaaugcGGCCAGUc |
| NM_001004067 | NOMO3 | circRNA | chr16 | 16372511 | 16372532 | + | 1 | 0 | AGO1-4 | 8mer | ucgcccugaaacucCCGGUCAa | cuuggcagagcccuGGCCAGUa |
| NM_004996 | ABCC1 | circRNA | chr16 | 16372511 | 16372532 | + | 1 | 0 | AGO1-4 | 8mer | ucgcccugaaacucCCGGUCAa | cuuggcagagcccuGGCCAGUa |
| NM_014287 | NOMO1 | circRNA | chr16 | 16372511 | 16372532 | + | 1 | 0 | AGO1-4 | 8mer | ucgcccugaaacucCCGGUCAa | cuuggcagagcccuGGCCAGUa |
| NM_001004067 | NOMO3 | circRNA | chr16 | 16372519 | 16372532 | + | 1 | 0 | AGO1-4 | 8mer | ucgcccugaaacucCCGGUCAa | #NAME? |
| NM_004996 | ABCC1 | circRNA | chr16 | 16372519 | 16372532 | + | 1 | 0 | AGO1-4 | 8mer | ucgcccugaaacucCCGGUCAa | #NAME? |
| NM_014287 | NOMO1 | circRNA | chr16 | 16372519 | 16372532 | + | 1 | 0 | AGO1-4 | 8mer | ucgcccugaaacucCCGGUCAa | #NAME? |
| NM_001004067 | NOMO3 | circRNA | chr16 | 16379302 | 16379323 | + | 2 | 1 | AGO1-4 | 7mer-m8 | ucgcccugaaacucCCGGUCAa | agacaguuucccuuGGCCAGUc |
| NM_004996 | ABCC1 | circRNA | chr16 | 16379302 | 16379323 | + | 2 | 1 | AGO1-4 | 7mer-m8 | ucgcccugaaacucCCGGUCAa | agacaguuucccuuGGCCAGUc |
| NM_014287 | NOMO1 | circRNA | chr16 | 16379302 | 16379323 | + | 2 | 1 | AGO1-4 | 7mer-m8 | ucgcccugaaacucCCGGUCAa | agacaguuucccuuGGCCAGUc |
| NM_173614 | NOMO2 | circRNA | chr16 | 18520527 | 18520548 | - | 3 | 1 | AGO1-4 | 7mer-m8 | ucgcccugaaacucCCGGUCAa | agacaguuucccuuGGCCAGUc |
| NM_173614 | NOMO2 | circRNA | chr16 | 18555043 | 18555064 | - | 2 | 0 | AGO1-4 | 7mer-m8 | ucgcccugaaacucCCGGUCAa | uccaaugccaaugcGGCCAGUc |
| NM_001128301 | LYRM1 | circRNA | chr16 | 20931518 | 20931539 | + | 3 | 0 | AGO1-4 | 8mer | ucgcccugaaacUCCCGGUCAa | gauuccuuacccAAGGCCAGUa |
| NR_002594 | SLC7A5P2 | circRNA | chr16 | 21521706 | 21521727 | - | 1 | 0 | AGO1-4 | 7mer-m8 | ucGCC-CUGAAACUCCCGGUCAa | caCGGUGACCCUG-GGGCCAGUc |
| NR_027155 | LOC100271836 | circRNA | chr16 | 21521706 | 21521727 | - | 1 | 0 | AGO1-4 | 7mer-m8 | ucGCC-CUGAAACUCCCGGUCAa | caCGGUGACCCUG-GGGCCAGUc |
| NM_013302 | EEF2K | circRNA | chr16 | 22237079 | 22237103 | + | 1 | 0 | AGO1-4 | 7mer-m8 | ucgccCUGAAACU--C-CCGGUCAa | uggaaGGCGUUGAUGGCGGCCAGUc |
| NM_020718 | USP31 | circRNA | chr16 | 23125495 | 23125517 | - | 1 | 0 | AGO1-4 | 7mer-m8 | ucgcccuGA-AACUCCCGGUCAa | cgaccuuCUGUUUAAGGCCAGUu |
| NM_020718 | USP31 | circRNA | chr16 | 23160110 | 23160131 | - | 1 | 0 | AGO1-4 | 8mer | ucgcccugaaacuCCCGGUCAa | aguaccuggcgcuGGGCCAGUa |
| NM_001083614 | EARS2 | circRNA | chr16 | 23533577 | 23533598 | - | 18 | 0 | AGO1,AGO1-4,AGO2,AGO3 | 8mer | ucgcccugaaacucCCGGUCAa | ugcuaccauuccuuGGCCAGUa |
| NR_003501 | EARS2 | circRNA | chr16 | 23533577 | 23533598 | - | 18 | 0 | AGO1,AGO1-4,AGO2,AGO3 | 8mer | ucgcccugaaacucCCGGUCAa | ugcuaccauuccuuGGCCAGUa |
| NM_019116 | UBFD1 | circRNA | chr16 | 23583054 | 23583075 | + | 7 | 0 | AGO1-4,AGO2 | 7mer-m8 | ucgcccugaaacucCCGGUCAa | gacccuagcagguuGGCCAGUg |
| NM_014494 | TNRC6A | circRNA | chr16 | 24801058 | 24801079 | + | 8 | 0 | AGO1-4,AGO2 | 8mer | ucgcccugaaacucCCGGUCAa | caaccauggugccuGGCCAGUa |
| NM_000418 | IL4R | circRNA | chr16 | 27374876 | 27374897 | + | 1 | 0 | AGO1-4 | 7mer-m8 | ucgcccuGAAACUCCCGGUCAa | cagacccCUGUCAUGGCCAGUc |
| NM_015171 | XPO6 | circRNA | chr16 | 28115929 | 28115950 | - | 3 | 0 | AGO1,AGO1-4 | 7mer-m8 | ucgcccUGAAACUCCCGGUCAa | aaguccACCGUGCUGGCCAGUg |
| NM_001142450 | SPNS1 | circRNA | chr16 | 28986516 | 28986535 | + | 1 | 0 | AGO1-4 | 7mer-m8 | ucGCCCUGAAACU-CCCGGUCAa | acCCGGAC---GACGGGCCAGUg |
| NM_032038 | SPNS1 | circRNA | chr16 | 28995588 | 28995609 | + | 1 | 0 | AGO2 | 7mer-m8 | ucgcccugaaacucCCGGUCAa | acccgcgugcccguGGCCAGUg |
| NM_001031827 | BOLA2 | circRNA | chr16 | 29872446 | 29872470 | - | 3 | 0 | AGO1-4,AGO2 | 7mer-m8 | ucgccCUGAAACU--C-CCGGUCAa | agcauGAGUUUGGAUGUGGCCAGUc |
| NM_006319 | CDIPT | circRNA | chr16 | 29872446 | 29872470 | - | 3 | 0 | AGO1-4,AGO2 | 7mer-m8 | ucgccCUGAAACU--C-CCGGUCAa | agcauGAGUUUGGAUGUGGCCAGUc |
| NR_002453 | LOC595101 | circRNA | chr16 | 29872446 | 29872470 | - | 3 | 0 | AGO1-4,AGO2 | 7mer-m8 | ucgccCUGAAACU--C-CCGGUCAa | agcauGAGUUUGGAUGUGGCCAGUc |
| TCONS_l2_00010452 | TCONS_l2_00010452 | circRNA | chr16 | 29872446 | 29872470 | - | 3 | 0 | AGO1-4,AGO2 | 7mer-m8 | ucgccCUGAAACU--C-CCGGUCAa | agcauGAGUUUGGAUGUGGCCAGUc |
| NM_004783 | TAOK2 | circRNA | chr16 | 30003029 | 30003052 | + | 1 | 0 | AGO1-4 | 7mer-m8 | ucgcccuGAAACUC--CCGGUCAa | ugagcuuCUUGGGGCUGGCCAGUg |
| NM_001031827 | BOLA2 | circRNA | chr16 | 30019782 | 30019803 | - | 1 | 0 | AGO1-4 | 7mer-m8 | ucgcccugaaacucCCGGUCAa | cccuucuuccaacuGGCCAGUu |
| NR_002453 | LOC595101 | circRNA | chr16 | 30019782 | 30019803 | - | 1 | 0 | AGO1-4 | 7mer-m8 | ucgcccugaaacucCCGGUCAa | cccuucuuccaacuGGCCAGUu |
| TCONS_l2_00010452 | TCONS_l2_00010452 | circRNA | chr16 | 30019782 | 30019803 | - | 1 | 0 | AGO1-4 | 7mer-m8 | ucgcccugaaacucCCGGUCAa | cccuucuuccaacuGGCCAGUu |
| NM_001031827 | BOLA2 | circRNA | chr16 | 30100570 | 30100591 | - | 1 | 0 | AGO1-4 | 7mer-m8 | ucgcccugaaacucCCGGUCAa | cugggcccaccccuGGCCAGUg |
| NR_002453 | LOC595101 | circRNA | chr16 | 30100570 | 30100591 | - | 1 | 0 | AGO1-4 | 7mer-m8 | ucgcccugaaacucCCGGUCAa | cugggcccaccccuGGCCAGUg |
| TCONS_l2_00010452 | TCONS_l2_00010452 | circRNA | chr16 | 30100570 | 30100591 | - | 1 | 0 | AGO1-4 | 7mer-m8 | ucgcccugaaacucCCGGUCAa | cugggcccaccccuGGCCAGUg |
| NM_001031827 | BOLA2 | circRNA | chr16 | 30128204 | 30128225 | - | 4 | 0 | AGO1-4,AGO2 | 7mer-m8 | ucgcCCUGAAACUCCCGGUCAa | cgacGGAUGAGGUGGGCCAGUc |
| NR_002453 | LOC595101 | circRNA | chr16 | 30128204 | 30128225 | - | 4 | 0 | AGO1-4,AGO2 | 7mer-m8 | ucgcCCUGAAACUCCCGGUCAa | cgacGGAUGAGGUGGGCCAGUc |
| TCONS_l2_00010452 | TCONS_l2_00010452 | circRNA | chr16 | 30128204 | 30128225 | - | 4 | 0 | AGO1-4,AGO2 | 7mer-m8 | ucgcCCUGAAACUCCCGGUCAa | cgacGGAUGAGGUGGGCCAGUc |
| NM_001031827 | BOLA2 | circRNA | chr16 | 30198646 | 30198667 | - | 1 | 0 | AGO1-4 | 7mer-m8 | ucgcccuGAAACUCCCGGUCAa | uacauacCCUACGGGGCCAGUc |
| NR_002453 | LOC595101 | circRNA | chr16 | 30198646 | 30198667 | - | 1 | 0 | AGO1-4 | 7mer-m8 | ucgcccuGAAACUCCCGGUCAa | uacauacCCUACGGGGCCAGUc |
| TCONS_l2_00010452 | TCONS_l2_00010452 | circRNA | chr16 | 30198646 | 30198667 | - | 1 | 0 | AGO1-4 | 7mer-m8 | ucgcccuGAAACUCCCGGUCAa | uacauacCCUACGGGGCCAGUc |
| NM_001105079 | FBRS | circRNA | chr16 | 30675156 | 30675177 | + | 1 | 0 | AGO2 | 7mer-m8 | ucgcccugaaacucCCGGUCAa | ugacucccaggccaGGCCAGUg |
| NM_006662 | SRCAP | circRNA | chr16 | 30735399 | 30735417 | + | 6 | 0 | AGO1-4,AGO2 | 7mer-m8 | ucgccCUGAAACUCCCGGUCAa | gcuucGGCUCU---GGCCAGUc |
| NM_006662 | SRCAP | circRNA | chr16 | 30749054 | 30749075 | + | 6 | 1 | AGO1-4,AGO2 | 7mer-m8 | ucgccCUGAAACUCCCGGUCAa | gagcuGGCUUCUGUGGCCAGUu |
| NM_000294 | PHKG2 | circRNA | chr16 | 30767767 | 30767789 | + | 1 | 0 | AGO2 | 8mer | ucgccCU-GAAACUCCCGGUCAa | cgcauGAUCAUGGAGGGCCAGUa |
| NM_152288 | ORAI3 | circRNA | chr16 | 30960714 | 30960737 | + | 4 | 0 | AGO1-4,AGO2 | 7mer-m8 | ucGCCCUGAA--ACUCCCGGUCAa | acCUGGACCUCAUGGGGGCCAGUc |
| NM_025193 | HSD3B7 | circRNA | chr16 | 30997473 | 30997495 | + | 1 | 0 | AGO1-4 | 7mer-m8 | ucgcCCUGAAAC-UCCCGGUCAa | agacGUGUUUGGCAGGGCCAGUc |
| NM_024006 | VKORC1 | circRNA | chr16 | 31102700 | 31102717 | - | 1 | 0 | AGO1-4 | 7mer-m8 | ucGCCCUGAAACUCCCGGUCAa | uuCGGGA----GGUGGCCAGUg |
| NM_031490 | LONP2 | circRNA | chr16 | 48381506 | 48381527 | + | 2 | 0 | AGO1,AGO1-4 | 7mer-m8 | ucgcCCUGAAACUCCCGGUCAa | gaauGGAUGGCGAGGGCCAGUu |
| NM_005611 | RBL2 | circRNA | chr16 | 53504493 | 53504514 | + | 2 | 0 | AGO1-4 | 7mer-m8 | ucgcccugaaacUCCCGGUCAa | aacagcagaagcAAGGCCAGUc |
| NM_007006 | NUDT21 | circRNA | chr16 | 56463366 | 56463390 | - | 6 | 0 | AGO1-4,AGO2,AGO3 | 7mer-m8 | ucgcccUGA-AACU--CCCGGUCAa | uagguaACUCUAGAUUAGGCCAGUu |
| NM_152727 | CPNE2 | circRNA | chr16 | 57147339 | 57147360 | + | 2 | 0 | AGO1-4 | 7mer-m8 | ucgccCUGAAACUCCCGGUCAa | agcauGACUUCCUGGGCCAGUu |
| NM_001130100 | KIFC3 | circRNA | chr16 | 57799518 | 57799539 | - | 4 | 0 | AGO1-4,AGO2 | 7mer-m8 | ucgcccugaaacucCCGGUCAa | gauugcucguguccGGCCAGUc |
| NM_024598 | C16orf57 | circRNA | chr16 | 58055186 | 58055209 | + | 1 | 0 | AGO1 | 7mer-m8 | ucgcccUGAAACU--CCCGGUCAa | uguuucAUUUUGGCCUGGCCAGUg |
| NM_001795 | CDH5 | circRNA | chr16 | 66437107 | 66437128 | + | 2 | 0 | AGO1-4 | 7mer-m8 | ucgcccugaaacucCCGGUCAa | cccccugcagcccaGGCCAGUc |
| NM_020786 | PDP2 | circRNA | chr16 | 66924693 | 66924711 | + | 1 | 0 | AGO1-4 | 7mer-m8 | ucgCCCUGAAACUCCCGGUCAa | cugGGGA---UAAAGGCCAGUg |
| NM_032140 | C16orf48 | circRNA | chr16 | 67696942 | 67696963 | - | 1 | 0 | AGO1 | 7mer-m8 | ucgcccugaaaCUCCCGGUCAa | aggagcaggggGGUGGCCAGUg |
| NM_001076785 | SLC7A6 | circRNA | chr16 | 68331347 | 68331366 | + | 2 | 0 | AGO1,AGO2 | 7mer-m8 | ucGCCCUGAAACUCCCGGUCAa | agUGGGGC--UCAGGGCCAGUg |
| NM_032382 | COG8 | circRNA | chr16 | 69368791 | 69368815 | - | 2 | 0 | AGO1-4 | 7mer-m8 | ucGCCCUGA-A-AC-UCCCGGUCAa | acCUGGACUCUCUGCUGGGCCAGUg |
| NM_007014 | WWP2 | circRNA | chr16 | 69974411 | 69974433 | + | 2 | 0 | AGO1-4,AGO2 | 7mer-m8 | ucGCCCUG-AAACUCCCGGUCAa | cgCAGGACACAUGCCGGCCAGUu |
| NM_199424 | WWP2 | circRNA | chr16 | 69974411 | 69974433 | + | 2 | 0 | AGO1-4,AGO2 | 7mer-m8 | ucGCCCUG-AAACUCCCGGUCAa | cgCAGGACACAUGCCGGCCAGUu |
| NM_012426 | SF3B3 | circRNA | chr16 | 70563043 | 70563064 | + | 7 | 0 | AGO1-4,AGO2 | 7mer-m8 | ucGCCCUGAAACUCCCGGUCAa | guCGCAUCGUUCCUGGCCAGUu |
| NM_001030007 | AP1G1 | circRNA | chr16 | 71783819 | 71783840 | - | 4 | 0 | AGO1-4 | 7mer-m8 | ucgcccugaaacucCCGGUCAa | aucuucuuguaucuGGCCAGUg |
| NM_018124 | RFWD3 | circRNA | chr16 | 74657080 | 74657102 | - | 4 | 0 | AGO1-4,AGO2 | 7mer-m8 | ucgcccugaaacUC-CCGGUCAa | aaagguaaggaaAGAGGCCAGUu |
| NM_030581 | WDR59 | circRNA | chr16 | 74907796 | 74907816 | - | 2 | 0 | AGO1-4,AGO2 | 7mer-m8 | ucgccCUGAAACUCCCGGUCAa | caccuGGC-UCCAGGGCCAGUg |
| NM_198390 | CMIP | circRNA | chr16 | 81725396 | 81725417 | + | 1 | 0 | AGO1-4 | 7mer-m8 | ucgcccugaaacucCCGGUCAa | uggagaccgucucuGGCCAGUu |
| NM_001537 | HSBP1 | circRNA | chr16 | 83846322 | 83846345 | + | 2 | 0 | AGO1-4 | 8mer | ucgcccuGAAACU--CCCGGUCAa | gaaccucCUUUGAUCAGGCCAGUa |
| NM_182981 | OSGIN1 | circRNA | chr16 | 83994601 | 83994626 | + | 1 | 0 | AGO1-4 | 7mer-m8 | ucgCCCUGAAACUC----CCGGUCAa | augGUGAUCCUGAGCCAAGGCCAGUg |
| NM_005679 | TAF1C | circRNA | chr16 | 84218619 | 84218643 | - | 2 | 0 | AGO1-4 | 7mer-m8 | ucgccCUGA-AACUC--CCGGUCAa | uuccaGUCUCUCGAGGUGGCCAGUg |
| NM_020947 | KIAA1609 | circRNA | chr16 | 84511366 | 84511384 | - | 1 | 0 | AGO2 | 7mer-m8 | ucGCCCUGAAACUCCCGGUCAa | ugCAGGGCU---GGGGCCAGUg |
| NM_024731 | KLHL36 | circRNA | chr16 | 84695720 | 84695738 | + | 1 | 0 | AGO1 | 7mer-m8 | ucGCCCUGAAACUCCCGGUCAa | acCAGGAC---CGGGGCCAGUg |
| NM_031476 | CRISPLD2 | circRNA | chr16 | 84879416 | 84879437 | + | 2 | 0 | AGO1-4 | 7mer-m8 | ucgcccugaaaCUCCCGGUCAa | ucugcugcagcGUGGGCCAGUc |
| NM_003486 | SLC7A5 | circRNA | chr16 | 87866530 | 87866552 | - | 13 | 1 | AGO1-4,AGO2 | 7mer-m8 | ucgcccugaaacUC-CCGGUCAa | aggagcaugcgcAGAGGCCAGUu |
| NM_013275 | ANKRD11 | circRNA | chr16 | 89348775 | 89348793 | - | 2 | 0 | AGO1-4,AGO2 | 8mer | ucgcCCUGAAACUCCCGGUCAa | ggaaGGACU---CCGGCCAGUa |
| NM_014972 | TCF25 | circRNA | chr16 | 89977697 | 89977718 | + | 5 | 0 | AGO1-4,AGO2 | 7mer-m8 | ucgcCCUGAAACUCCCGGUCAa | uguuGGUCGGAGUCGGCCAGUu |
| NM_001242822 | DEF8 | circRNA | chr16 | 90025662 | 90025683 | + | 1 | 0 | AGO1-4 | 7mer-m8 | ucgcccugaaacUCCCGGUCAa | cacuagccaaacAUGGCCAGUc |
| NM_017702 | DEF8 | circRNA | chr16 | 90025662 | 90025683 | + | 1 | 0 | AGO1-4 | 7mer-m8 | ucgcccugaaacUCCCGGUCAa | cacuagccaaacAUGGCCAGUc |
| NM_018146 | RNMTL1 | circRNA | chr17 | 685795 | 685817 | + | 1 | 0 | AGO1-4 | 7mer-m8 | ucgCCCUG--AAACUCCCGGUCAa | caaGGCACCAUCUGA-GGCCAGUg |
| NM_016823 | CRK | circRNA | chr17 | 1325943 | 1325964 | - | 5 | 0 | AGO1-4,AGO2 | 8mer | ucgcccugaaaCUCCCGGUCAa | ccauuucuccaGAGGGCCAGUa |
| NM_001080779 | MYO1C | circRNA | chr17 | 1383912 | 1383919 | - | 2 | 0 | AGO1-4 | 7mer-m8 | ucgcccugaaacucCCGGUCAa | #NAME? |
| NM_006224 | PITPNA | circRNA | chr17 | 1456325 | 1456349 | - | 4 | 0 | AGO1-4 | 8mer | ucgcCCUGAAACUC---CCGGUCAa | agaaGGACGGUGAGAAAGGCCAGUa |
| NM_006224 | PITPNA | circRNA | chr17 | 1456404 | 1456422 | - | 11 | 0 | AGO1-4,AGO2 | 8mer | ucGCCCUGAAACUCCCGGUCAa | ucUGUGGC--UGA-GGCCAGUa |
| NM_152346 | SLC43A2 | circRNA | chr17 | 1477975 | 1477996 | - | 1 | 0 | AGO2 | 7mer-m8 | ucgcccugaaacuCCCGGUCAa | caggccugcagcuGGGCCAGUg |
| hsa_circ_0000731 | hsa_circ_001263 | circRNA | chr17 | 1477975 | 1477996 | - | 1 | 0 | AGO2 | 7mer-m8 | ucgcccugaaacuCCCGGUCAa | caggccugcagcuGGGCCAGUg |
| NM_006445 | PRPF8 | circRNA | chr17 | 1577857 | 1577879 | - | 13 | 0 | AGO1-4,AGO2 | 7mer-m8 | ucGCCCUGAAACU--CCCGGUCAa | auUGGGA-UUGCACCGGGCCAGUg |
| NM_015113 | ZZEF1 | circRNA | chr17 | 3954192 | 3954213 | - | 1 | 0 | AGO1-4 | 7mer-m8 | ucgcccugaaacucCCGGUCAa | uauagcgcccaccuGGCCAGUg |
| NM_016376 | ANKFY1 | circRNA | chr17 | 4069773 | 4069799 | - | 13 | 0 | AGO1-4,AGO2 | 7mer-m8 | ucgcccuGAAACU--C---CCGGUCAa | acuuuucUUUUGAAGGUUUGGCCAGUu |
| NM_001212 | C1QBP | circRNA | chr17 | 5336286 | 5336304 | - | 15 | 1 | AGO1-4,AGO2 | 7mer-m8 | ucgccCUGAAACUCCCGGUCAa | uggcaGGCUUU---GGCCAGUg |
| NM_020162 | DHX33 | circRNA | chr17 | 5356972 | 5356988 | - | 1 | 0 | AGO1 | 7mer-m8 | ucgcccugaaacucCCGGUCAa | #NAME? |
| NM_016041 | DERL2 | circRNA | chr17 | 5386161 | 5386182 | - | 2 | 0 | AGO2 | 7mer-m8 | ucgcccUGAAACUCCCGGUCAa | cuucuuAUUUUUUGGGCCAGUu |
| NM_032442 | NEURL4 | circRNA | chr17 | 7224557 | 7224579 | - | 1 | 0 | AGO1-4 | 7mer-m8 | ucgcCCU-GAAACUCCCGGUCAa | uguuGGAUCUCUACGGGCCAGUc |
| NM_001128833 | ZBTB4 | circRNA | chr17 | 7365604 | 7365624 | - | 4 | 0 | AGO1-4 | 7mer-m8 | ucGCCCUGAAACUCCCGGUCAa | ucUGGGA-GUGAAGGGCCAGUg |
| NM_000937 | POLR2A | circRNA | chr17 | 7411745 | 7411769 | + | 8 | 0 | AGO1-4,AGO2 | 7mer-m8 | ucgccCUGAA--AC-UCCCGGUCAa | uuacuGUCUUCCUGUUGGGCCAGUc |
| NM_004860 | FXR2 | circRNA | chr17 | 7507149 | 7507167 | - | 2 | 0 | AGO2 | 7mer-m8 | ucgCCCUGAAACUCCCGGUCAa | ggaGCGACUU---CGGCCAGUu |
| NM_025099 | CTC1 | circRNA | chr17 | 8138550 | 8138570 | - | 1 | 0 | AGO1-4 | 7mer-m8 | ucgCCCUGAAACUCCCGGUCAa | gggGGGAC-AAGAAGGCCAGUg |
| NM_153210 | USP43 | circRNA | chr17 | 9613279 | 9613303 | + | 1 | 0 | AGO1-4 | 7mer-m8 | ucGCCCUGA---AACUCCCGGUCAa | gcCGGAACUCUCUGGAUGGCCAGUg |
| NR_037985 | TTC19 | circRNA | chr17 | 15907536 | 15907557 | + | 1 | 0 | AGO1-4 | 8mer | ucgcccugaaacucCCGGUCAa | auuucccuaaagcuGGCCAGUa |
| NM_201274 | MPRIP | circRNA | chr17 | 17095680 | 17095700 | + | 1 | 0 | AGO1-4 | 7mer-m8 | ucgcccUGAAACUCCCGGUCAa | ccauccACUGUCA-GGCCAGUg |
| NM_017758 | ALKBH5 | circRNA | chr17 | 18111960 | 18111982 | + | 8 | 0 | AGO1,AGO1-4,AGO2 | 8mer | ucgcccugaAACUC-CCGGUCAa | ggcugaugcUGGAGUGGCCAGUa |
| NM_001243439 | SPECC1 | circRNA | chr17 | 20109048 | 20109070 | + | 1 | 0 | AGO2 | 7mer-m8 | ucgcccugaaacUC-CCGGUCAa | gcagaaagccacAGAGGCCAGUg |
| NM_152904 | SPECC1 | circRNA | chr17 | 20109048 | 20109070 | + | 1 | 0 | AGO2 | 7mer-m8 | ucgcccugaaacUC-CCGGUCAa | gcagaaagccacAGAGGCCAGUg |
| hsa_circ_0000745 | hsa_circ_000013 | circRNA | chr17 | 20109048 | 20109070 | + | 1 | 0 | AGO2 | 7mer-m8 | ucgcccugaaacUC-CCGGUCAa | gcagaaagccacAGAGGCCAGUg |
| NM_015584 | POLDIP2 | circRNA | chr17 | 26674568 | 26674589 | - | 6 | 0 | AGO1-4,AGO2 | 7mer-m8 | ucgcccugaaaCUCCCGGUCAa | aaugcagagagGGAGGCCAGUg |
| NM_014680 | KIAA0100 | circRNA | chr17 | 26960700 | 26960722 | - | 5 | 0 | AGO1-4,AGO2 | 7mer-m8 | ucGCCCUGAAACU-CCCGGUCAa | gcCUGGACACUAAUUGGCCAGUg |
| NM_144683 | DHRS13 | circRNA | chr17 | 27228586 | 27228609 | - | 1 | 0 | AGO2 | 7mer-m8 | ucgcCCUGAAACU--CCCGGUCAa | ucauGGCCUUGGACUUGGCCAGUc |
| NM_001033561 | PHF12 | circRNA | chr17 | 27239966 | 27239989 | - | 1 | 0 | AGO2 | 7mer-m8 | ucGCCCUGAAACU--CCCGGUCAa | ugUGGGACUGCCAAUGGGCCAGUg |
| NM_020889 | PHF12 | circRNA | chr17 | 27239966 | 27239989 | - | 1 | 0 | AGO2 | 7mer-m8 | ucGCCCUGAAACU--CCCGGUCAa | ugUGGGACUGCCAAUGGGCCAGUg |
| NM_020791 | TAOK1 | circRNA | chr17 | 27877017 | 27877038 | + | 2 | 0 | AGO2 | 8mer | ucgcccuGAAACUCCCGGUCAa | aaaagucCUCUUAAGGCCAGUa |
| NM_004871 | GOSR1 | circRNA | chr17 | 28852950 | 28852971 | + | 1 | 0 | AGO2 | 7mer-m8 | ucgcccugaaacucCCGGUCAa | cauguagacagcuaGGCCAGUg |
| NM_024857 | ATAD5 | circRNA | chr17 | 29076292 | 29076313 | + | 3 | 0 | AGO1-4 | 7mer-m8 | ucgcccugaaacucCCGGUCAa | caccauguugcccaGGCCAGUu |
| NR_015341 | LRRC37BP1 | circRNA | chr17 | 29076292 | 29076313 | + | 3 | 0 | AGO1-4 | 7mer-m8 | ucgcccugaaacucCCGGUCAa | caccauguugcccaGGCCAGUu |
| NM_015355 | SUZ12 | circRNA | chr17 | 29117592 | 29117613 | + | 1 | 0 | AGO2 | 8mer | ucgcccugaAACUCCCGGUCAa | caaaacaaaUUCUUGGCCAGUa |
| NM_024857 | ATAD5 | circRNA | chr17 | 29117592 | 29117613 | + | 1 | 0 | AGO2 | 8mer | ucgcccugaAACUCCCGGUCAa | caaaacaaaUUCUUGGCCAGUa |
| NR_015341 | LRRC37BP1 | circRNA | chr17 | 29117592 | 29117613 | + | 1 | 0 | AGO2 | 8mer | ucgcccugaAACUCCCGGUCAa | caaaacaaaUUCUUGGCCAGUa |
| NM_015355 | SUZ12 | circRNA | chr17 | 29158098 | 29158121 | + | 2 | 0 | AGO1-4 | 8mer | ucgcCCUGAAACU--CCCGGUCAa | aagcGGAUUCAGAGGGGGCCAGUa |
| NM_024857 | ATAD5 | circRNA | chr17 | 29158098 | 29158121 | + | 2 | 0 | AGO1-4 | 8mer | ucgcCCUGAAACU--CCCGGUCAa | aagcGGAUUCAGAGGGGGCCAGUa |
| NR_015341 | LRRC37BP1 | circRNA | chr17 | 29158098 | 29158121 | + | 2 | 0 | AGO1-4 | 8mer | ucgcCCUGAAACU--CCCGGUCAa | aagcGGAUUCAGAGGGGGCCAGUa |
| NM_015355 | SUZ12 | circRNA | chr17 | 29286092 | 29286113 | + | 3 | 0 | AGO1,AGO2 | 7mer-m8 | ucgcCCUGAAACUCCCGGUCAa | gguuGUGCAUUAAGGGCCAGUu |
| NM_024857 | ATAD5 | circRNA | chr17 | 29286092 | 29286113 | + | 3 | 0 | AGO1,AGO2 | 7mer-m8 | ucgcCCUGAAACUCCCGGUCAa | gguuGUGCAUUAAGGGCCAGUu |
| NR_015341 | LRRC37BP1 | circRNA | chr17 | 29286092 | 29286113 | + | 3 | 0 | AGO1,AGO2 | 7mer-m8 | ucgcCCUGAAACUCCCGGUCAa | gguuGUGCAUUAAGGGCCAGUu |
| NM_001042492 | NF1 | circRNA | chr17 | 29657358 | 29657379 | + | 2 | 0 | AGO1-4,AGO2 | 7mer-m8 | ucgcccugaAACUCCCGGUCAa | auuuaaaaaUCGAGGGCCAGUu |
| NM_015355 | SUZ12 | circRNA | chr17 | 29657358 | 29657379 | + | 2 | 0 | AGO1-4,AGO2 | 7mer-m8 | ucgcccugaAACUCCCGGUCAa | auuuaaaaaUCGAGGGCCAGUu |
| NM_001042492 | NF1 | circRNA | chr17 | 29684336 | 29684359 | + | 2 | 0 | AGO1-4 | 7mer-m8 | ucgcccUGAAAC-UC-CCGGUCAa | augaauACUUAGCAGAGGCCAGUg |
| NM_015355 | SUZ12 | circRNA | chr17 | 29684336 | 29684359 | + | 2 | 0 | AGO1-4 | 7mer-m8 | ucgcccUGAAAC-UC-CCGGUCAa | augaauACUUAGCAGAGGCCAGUg |
| NM_001042492 | NF1 | circRNA | chr17 | 29701494 | 29701515 | + | 14 | 0 | AGO1-4,AGO2 | 7mer-m8 | ucgcccugaaacucCCGGUCAa | auacugcuacuacuGGCCAGUg |
| NM_015355 | SUZ12 | circRNA | chr17 | 29701494 | 29701515 | + | 14 | 0 | AGO1-4,AGO2 | 7mer-m8 | ucgcccugaaacucCCGGUCAa | auacugcuacuacuGGCCAGUg |
| NM_015355 | SUZ12 | circRNA | chr17 | 29889650 | 29889671 | + | 1 | 0 | AGO1-4 | 7mer-m8 | ucgcccugaaacuCCCGGUCAa | uaauuccgcugcuGGGCCAGUc |
| NM_015355 | SUZ12 | circRNA | chr17 | 29889689 | 29889710 | + | 1 | 0 | AGO1-4 | 7mer-m8 | ucgcccugaaacucCCGGUCAa | gcugguaaauauuuGGCCAGUc |
| NR_037713 | RFFL | circRNA | chr17 | 33338785 | 33338806 | - | 1 | 0 | AGO1-4 | 7mer-m8 | ucgcccugaaacUCCCGGUCAa | cuggcaaagcccAAGGCCAGUg |
| NM_198839 | ACACA | circRNA | chr17 | 35443455 | 35443476 | - | 1 | 0 | AGO1-4 | 7mer-m8 | ucGCCCUGAAACUCCCGGUCAa | gcUGGGAGCUGCAAGGCCAGUg |
| NM_198836 | ACACA | circRNA | chr17 | 35603758 | 35603779 | - | 6 | 0 | AGO1-4,AGO2 | 7mer-m8 | ucgcccugAAACUCCCGGUCAa | gucaugugUUUGCCGGCCAGUg |
| NM_198838 | ACACA | circRNA | chr17 | 35603758 | 35603779 | - | 6 | 0 | AGO1-4,AGO2 | 7mer-m8 | ucgcccugAAACUCCCGGUCAa | gucaugugUUUGCCGGCCAGUg |
| NM_198839 | ACACA | circRNA | chr17 | 35603758 | 35603779 | - | 6 | 0 | AGO1-4,AGO2 | 7mer-m8 | ucgcccugAAACUCCCGGUCAa | gucaugugUUUGCCGGCCAGUg |
| NM_001488 | TADA2A | circRNA | chr17 | 35825593 | 35825614 | + | 2 | 0 | AGO2 | 7mer-m8 | ucgccCUGAAACUCCCGGUCAa | ugcaaGAAUUGUGGGGCCAGUg |
| NM_007247 | SYNRG | circRNA | chr17 | 35937568 | 35937589 | - | 1 | 0 | AGO1-4 | 8mer | ucgcccUGAAACUCCCGGUCAa | uaucccAGUUUAAUGGCCAGUa |
| NM_007247 | SYNRG | circRNA | chr17 | 35939359 | 35939380 | - | 2 | 0 | AGO1-4,AGO2 | 7mer-m8 | ucgcccugaaacucCCGGUCAa | ccuccaccccacuuGGCCAGUu |
| NM_005937 | MLLT6 | circRNA | chr17 | 36872387 | 36872407 | + | 1 | 0 | AGO1-4 | 8mer | ucgcccugaaACUCCCGGUCAa | agucaucuggUGA-GGCCAGUa |
| NM_005937 | MLLT6 | circRNA | chr17 | 36876047 | 36876071 | + | 6 | 0 | AGO1-4,AGO2 | 7mer-m8 | ucgcccuGAAACUC---CCGGUCAa | cugcaccCUGUGGGGGCGGCCAGUu |
| NM_032339 | MIEN1 | circRNA | chr17 | 37886501 | 37886524 | - | 3 | 0 | AGO1-4,AGO2 | 7mer-m8 | ucgcccUGAAACUC--CCGGUCAa | cgaccuACCUGGAGCUGGCCAGUg |
| NM_001096 | ACLY | circRNA | chr17 | 40065308 | 40065321 | - | 4 | 0 | AGO1-4 | 7mer-m8 | ucgcccugaaacucCCGGUCAa | #NAME? |
| NM_001070 | TUBG1 | circRNA | chr17 | 40761772 | 40761793 | + | 1 | 0 | AGO1-4 | 7mer-m8 | ucgcccugaaacuCCCGGUCAa | ucacccuacaguuGGGCCAGUg |
| NM_007300 | BRCA1 | circRNA | chr17 | 41246245 | 41246265 | - | 2 | 0 | AGO1-4,AGO2 | 7mer-m8 | ucgccCUGAAACUCCCGGUCAa | aaauaGAC-UUACUGGCCAGUg |
| NM_133373 | PLCD3 | circRNA | chr17 | 43190260 | 43190281 | - | 2 | 0 | AGO1-4,AGO2 | 7mer-m8 | ucgccCUGAAACUCCCGGUCAa | ccaauGACUUUGUGGGCCAGUu |
| hsa_circ_0044175 | hsa_circ_0044175 | circRNA | chr17 | 43514590 | 43514611 | - | 2 | 0 | AGO1,AGO1-4 | 7mer-m8 | ucgcccugaaacUCCCGGUCAa | ccucagcagggcAGGGCCAGUc |
| hsa_circ_0003434 | hsa_circ_0003434 | circRNA | chr17 | 43552614 | 43552631 | - | 3 | 0 | AGO1-4 | 7mer-m8 | ucGCCCUGAAACUCCCGGUCAa | gcCUGGAC----ACGGCCAGUu |
| hsa_circ_0008067 | hsa_circ_0008067 | circRNA | chr17 | 43552614 | 43552631 | - | 3 | 0 | AGO1-4 | 7mer-m8 | ucGCCCUGAAACUCCCGGUCAa | gcCUGGAC----ACGGCCAGUu |
| hsa_circ_0044175 | hsa_circ_0044175 | circRNA | chr17 | 43552614 | 43552631 | - | 3 | 0 | AGO1-4 | 7mer-m8 | ucGCCCUGAAACUCCCGGUCAa | gcCUGGAC----ACGGCCAGUu |
| hsa_circ_0044176 | hsa_circ_0044176 | circRNA | chr17 | 43552614 | 43552631 | - | 3 | 0 | AGO1-4 | 7mer-m8 | ucGCCCUGAAACUCCCGGUCAa | gcCUGGAC----ACGGCCAGUu |
| hsa_circ_0044177 | hsa_circ_0044177 | circRNA | chr17 | 43552614 | 43552631 | - | 3 | 0 | AGO1-4 | 7mer-m8 | ucGCCCUGAAACUCCCGGUCAa | gcCUGGAC----ACGGCCAGUu |
| NM_030753 | WNT3 | circRNA | chr17 | 44223751 | 44223772 | - | 1 | 0 | AGO1-4 | 7mer-m8 | ucgcccugaaacucCCGGUCAa | cugaguaccagcuuGGCCAGUc |
| hsa_circ_0044187 | hsa_circ_0044187 | circRNA | chr17 | 44223751 | 44223772 | - | 1 | 0 | AGO1-4 | 7mer-m8 | ucgcccugaaacucCCGGUCAa | cugaguaccagcuuGGCCAGUc |
| hsa_circ_0044189 | hsa_circ_0044189 | circRNA | chr17 | 44223751 | 44223772 | - | 1 | 0 | AGO1-4 | 7mer-m8 | ucgcccugaaacucCCGGUCAa | cugaguaccagcuuGGCCAGUc |
| hsa_circ_0044190 | hsa_circ_0044190 | circRNA | chr17 | 44223751 | 44223772 | - | 1 | 0 | AGO1-4 | 7mer-m8 | ucgcccugaaacucCCGGUCAa | cugaguaccagcuuGGCCAGUc |
| hsa_circ_0044191 | hsa_circ_0044191 | circRNA | chr17 | 44223751 | 44223772 | - | 1 | 0 | AGO1-4 | 7mer-m8 | ucgcccugaaacucCCGGUCAa | cugaguaccagcuuGGCCAGUc |
| NM_004287 | GOSR2 | circRNA | chr17 | 45016770 | 45016790 | + | 6 | 0 | AGO1-4,AGO2 | 7mer-m8 | ucgcccugaaACUCCCGGUCAa | uuuucuaaagUGA-GGCCAGUg |
| NM_002265 | KPNB1 | circRNA | chr17 | 45760097 | 45760116 | + | 10 | 0 | AGO1-4,AGO2 | 7mer-m8 | ucgccCUGAAACUCCCGGUCAa | uuucuGUCUUU--UGGCCAGUg |
| NM_024015 | HOXB4 | circRNA | chr17 | 46653235 | 46653257 | - | 2 | 0 | AGO2 | 7mer-m8 | ucGCCCUGAAACU-CCCGGUCAa | cuUGGCAGGGUGAGGGGCCAGUu |
| NM_006546 | IGF2BP1 | circRNA | chr17 | 47129516 | 47129537 | + | 1 | 0 | AGO2 | 8mer | ucgcccugaaacucCCGGUCAa | uagggauaacaucaGGCCAGUa |
| NM_030802 | FAM117A | circRNA | chr17 | 47809968 | 47809989 | - | 1 | 0 | AGO1-4 | 7mer-m8 | ucgcccUGAAACUCCCGGUCAa | gcuccuACCUUCUGGGCCAGUg |
| NM_017643 | MBTD1 | circRNA | chr17 | 49257491 | 49257518 | - | 12 | 0 | AGO1-4,AGO2 | 7mer-m8 | ucGCCCUGAAACUC------CCGGUCAa | uuUGGGAUUUUUAGAAGUUAGGCCAGUc |
| NM_005082 | TRIM25 | circRNA | chr17 | 54965498 | 54965519 | - | 1 | 0 | AGO1-4 | 7mer-m8 | ucgcccugaaacucCCGGUCAa | gaguuuaaucaucuGGCCAGUg |
| NM_003488 | AKAP1 | circRNA | chr17 | 55183576 | 55183596 | + | 3 | 0 | AGO1-4,AGO2 | 7mer-m8 | ucgccCUGAAACUCCCGGUCAa | ucccaGGUGGUG-GGGCCAGUg |
| NM_003168 | SUPT4H1 | circRNA | chr17 | 56422899 | 56422918 | - | 3 | 0 | AGO1-4,AGO2 | 7mer-m8 | ucgcccUGAAACUCCCGGUCAa | uccuauACCUU-A-GGCCAGUc |
| NM_017763 | RNF43 | circRNA | chr17 | 56440735 | 56440756 | - | 1 | 0 | AGO1-4 | 7mer-m8 | ucGCCCUGAAACUCCCGGUCAa | gcUGGGGCUGACCUGGCCAGUg |
| NM_004687 | MTMR4 | circRNA | chr17 | 56572645 | 56572666 | - | 1 | 0 | AGO1 | 7mer-m8 | ucgcccUGAAACUCCCGGUCAa | ggcccuGCUUUGGGGGCCAGUg |
| NM_006039 | MRC2 | circRNA | chr17 | 60742241 | 60742263 | + | 2 | 0 | AGO1-4 | 7mer-m8 | ucgccCUGAAACUC-CCGGUCAa | gaccaGACCCGCAGUGGCCAGUg |
| NM_025185 | TANC2 | circRNA | chr17 | 61106194 | 61106214 | + | 1 | 0 | AGO1-4 | 7mer-m8 | ucgcccUGAAACUCCCGGUCAa | ucauauGUUCUCA-GGCCAGUg |
| NM_025185 | TANC2 | circRNA | chr17 | 61117107 | 61117131 | + | 1 | 0 | AGO1-4 | 8mer | ucgcccugAAACU---CCCGGUCAa | uuuuaauaUUUGAUUCUGGCCAGUa |
| NM_025185 | TANC2 | circRNA | chr17 | 61498249 | 61498270 | + | 4 | 0 | AGO1-4,AGO2 | 8mer | ucgcccugAAACUCCCGGUCAa | guagcccaUUCAAUGGCCAGUa |
| NM_025185 | TANC2 | circRNA | chr17 | 61504463 | 61504483 | + | 1 | 0 | AGO1-4 | 7mer-m8 | ucgcccugaaACUCCCGGUCAa | uccugcugagUGA-GGCCAGUu |
| NM_005828 | DCAF7 | circRNA | chr17 | 61666608 | 61666630 | + | 31 | 0 | AGO1,AGO1-4,AGO2,AGO3,AGO4 | 8mer | ucgcccUGAAACUC-CCGGUCAa | aaacauGUUUCCAGUGGCCAGUa |
| NM_005828 | DCAF7 | circRNA | chr17 | 61666671 | 61666691 | + | 16 | 0 | AGO1-4,AGO2 | 7mer-m8 | ucgcCCUGAAACUCCCGGUCAa | ucuaGGA-GUUCCUGGCCAGUc |
| NM_020198 | CCDC47 | circRNA | chr17 | 61838286 | 61838306 | - | 7 | 0 | AGO1-4,AGO2 | 7mer-m8 | ucGCCCUGAAACUCCCGGUCAa | gcCCGGAUGAUGA-GGCCAGUg |
| NM_004396 | DDX5 | circRNA | chr17 | 62500161 | 62500182 | - | 28 | 0 | AGO1,AGO1-4,AGO2 | 7mer-m8 | ucgcccugaaaCUCCCGGUCAa | ucaagcucaggGAUGGCCAGUu |
| NR_024386 | PLEKHM1P | circRNA | chr17 | 62818033 | 62818050 | - | 2 | 0 | AGO1-4 | 7mer-m8 | ucGCCCUGAAACUCCCGGUCAa | gcCUGGAC----ACGGCCAGUu |
| NM_002737 | PRKCA | circRNA | chr17 | 64620383 | 64620404 | + | 1 | 0 | AGO1-4 | 7mer-m8 | ucgcccugaaacUCCCGGUCAa | gguuagcaugaaAGGGCCAGUu |
| NM_002737 | PRKCA | circRNA | chr17 | 64620423 | 64620449 | + | 1 | 0 | AGO1-4 | 7mer-m8 | ucgcCCUG--AAACU---CCCGGUCAa | acauGGACAGUUUCAUCUUGGCCAGUu |
| NM_014877 | HELZ | circRNA | chr17 | 65134180 | 65134202 | - | 1 | 0 | AGO2 | 8mer | ucgcccugaaacUC-CCGGUCAa | aguuaagaaggaAGUGGCCAGUa |
| NM_014877 | HELZ | circRNA | chr17 | 65214805 | 65214826 | - | 5 | 0 | AGO1,AGO1-4,AGO2 | 8mer | ucgcccugaaacucCCGGUCAa | cccuucuuucucuuGGCCAGUa |
| NM_004252 | SLC9A3R1 | circRNA | chr17 | 72745079 | 72745098 | + | 4 | 0 | AGO1-4 | 8mer | ucgCCCUGAAACUCCCGGUCAa | aagGGCAAGUU--GGGCCAGUa |
| NM_004252 | SLC9A3R1 | circRNA | chr17 | 72758221 | 72758242 | + | 6 | 0 | AGO1-4 | 7mer-m8 | ucgcccugaaacucCCGGUCAa | acaaguccaagccaGGCCAGUu |
| NM_207346 | TSEN54 | circRNA | chr17 | 73520588 | 73520610 | + | 4 | 0 | AGO1-4,AGO2 | 7mer-m8 | ucGCCCUGAAACUC-CCGGUCAa | acCUGUAGCUUCAGAGGCCAGUc |
| NM_001145297 | EXOC7 | circRNA | chr17 | 74077922 | 74077944 | - | 4 | 0 | AGO1,AGO1-4,AGO2 | 7mer-m8 | ucgCCCUGAAACU-CCCGGUCAa | aggGAGAUGCUCAGGGGCCAGUc |
| NM_001145297 | EXOC7 | circRNA | chr17 | 74097785 | 74097805 | - | 1 | 0 | AGO1-4 | 7mer-m8 | ucgcccUGAAACUCCCGGUCAa | gcuacuACCAUG-UGGCCAGUg |
| NM_052916 | RNF157 | circRNA | chr17 | 74163838 | 74163863 | - | 1 | 0 | AGO2 | 8mer | ucgCCCUGAAACUC----CCGGUCAa | gaaGAGCCCUGGAGAAGAGGCCAGUa |
| NM_003016 | SRSF2 | circRNA | chr17 | 74731920 | 74731941 | - | 4 | 5 | AGO1,AGO1-4,AGO2 | 8mer | ucgcccugaaacucCCGGUCAa | gauaaaagaauauuGGCCAGUa |
| NM_001127198 | TMC6 | circRNA | chr17 | 76120774 | 76120795 | - | 3 | 0 | AGO1-4 | 7mer-m8 | ucgcCCUGAAACUCCCGGUCAa | ugaaGCGCAUCGGGGGCCAGUu |
| NM_025090 | USP36 | circRNA | chr17 | 76799870 | 76799894 | - | 1 | 0 | AGO1-4 | 7mer-m8 | ucgcccugaAAC--UC-CCGGUCAa | ccacaccagUUGCCAGAGGCCAGUg |
| NM_005567 | LGALS3BP | circRNA | chr17 | 76972157 | 76972182 | - | 6 | 0 | AGO1,AGO1-4,AGO2 | 7mer-m8 | ucgCCCU-GAA-A-CUC-CCGGUCAa | gugGAGAUCUUCUACAGAGGCCAGUg |
| NM_001159772 | CANT1 | circRNA | chr17 | 76989001 | 76989022 | - | 6 | 0 | AGO1-4,AGO2 | 7mer-m8 | ucgcccugaaacUCCCGGUCAa | ucuccgcaucauAUGGCCAGUg |
| NM_138793 | CANT1 | circRNA | chr17 | 76989001 | 76989022 | - | 6 | 0 | AGO1-4,AGO2 | 7mer-m8 | ucgcccugaaacUCCCGGUCAa | ucuccgcaucauAUGGCCAGUg |
| NM_001042573 | ENGASE | circRNA | chr17 | 77083706 | 77083727 | + | 2 | 0 | AGO1-4 | 7mer-m8 | ucGCCCUGAAACUCCCGGUCAa | ccCAGGCCACAUAGGGCCAGUg |
| NM_016538 | SIRT7 | circRNA | chr17 | 79869964 | 79869985 | - | 2 | 0 | AGO1-4,AGO2 | 7mer-m8 | ucgcccugaaacucCCGGUCAa | uuccucgcucaccaGGCCAGUc |
| NM_016538 | SIRT7 | circRNA | chr17 | 79870246 | 79870270 | - | 1 | 0 | AGO1-4 | 7mer-m8 | ucGCCCUGAAAC-UC--CCGGUCAa | guUGGCACUUUGCAGAUGGCCAGUg |
| NM_004104 | FASN | circRNA | chr17 | 80036498 | 80036519 | - | 8 | 0 | AGO1-4,AGO2 | 7mer-m8 | ucGCCCUGAAACUCCCGGUCAa | agUGGGGGGUCGUGGGCCAGUc |
| NM_004104 | FASN | circRNA | chr17 | 80043590 | 80043614 | - | 9 | 0 | AGO1-4,AGO2 | 7mer-m8 | ucgccCUGAAAC---UCCCGGUCAa | agcacGACGUUGCCCAGGGCCAGUg |
| NM_139062 | CSNK1D | circRNA | chr17 | 80200627 | 80200648 | - | 14 | 1 | AGO1-4,AGO2 | 7mer-m8 | ucGCCCUGAAACUCCCGGUCAa | agCUGUGCACUGACGGCCAGUc |
| NM_005993 | TBCD | circRNA | chr17 | 80887008 | 80887029 | + | 2 | 0 | AGO1-4,AGO3 | 7mer-m8 | ucGCCCUGAAACUCCCGGUCAa | ugUGUGGCCCAGCAGGCCAGUg |
| NM_001105244 | PTPRM | circRNA | chr18 | 7926597 | 7926620 | + | 1 | 0 | AGO2 | 7mer-m8 | ucGCCCUGAA--ACUCCCGGUCAa | ugUGGAAGUUAAUGCUGGCCAGUu |
| NM_001042388 | PPP4R1 | circRNA | chr18 | 9562947 | 9562968 | - | 1 | 0 | AGO2 | 7mer-m8 | ucgcccugaaaCUCCCGGUCAa | gccauguugcgGAGGGCCAGUg |
| NM_001042388 | PPP4R1 | circRNA | chr18 | 9577116 | 9577137 | - | 1 | 0 | AGO2 | 8mer | ucgcccugaaacucCCGGUCAa | auccaucuagcucaGGCCAGUa |
| NM_005134 | PPP4R1 | circRNA | chr18 | 9577116 | 9577137 | - | 1 | 0 | AGO2 | 8mer | ucgcccugaaacucCCGGUCAa | auccaucuagcucaGGCCAGUa |
| NM_032525 | TUBB6 | circRNA | chr18 | 12308305 | 12308326 | + | 2 | 0 | AGO1-4 | 7mer-m8 | ucgcccugaaaCUCCCGGUCAa | ugcacauccagGCGGGCCAGUg |
| NM_018030 | OSBPL1A | circRNA | chr18 | 21761138 | 21761159 | - | 5 | 0 | AGO1-4,AGO2 | 7mer-m8 | ucgcccugaaacUCCCGGUCAa | uaccucauccacAAGGCCAGUu |
| NM_080597 | OSBPL1A | circRNA | chr18 | 21761138 | 21761159 | - | 5 | 0 | AGO1-4,AGO2 | 7mer-m8 | ucgcccugaaacUCCCGGUCAa | uaccucauccacAAGGCCAGUu |
| NM_020474 | GALNT1 | circRNA | chr18 | 33168140 | 33168161 | + | 1 | 0 | AGO1-4 | 7mer-m8 | ucgcCCUGAAACUCCCGGUCAa | cuguGGAGAGAGAAGGCCAGUu |
| NM_012319 | SLC39A6 | circRNA | chr18 | 33694251 | 33694272 | - | 9 | 0 | AGO1-4,AGO2 | 7mer-m8 | ucgcccugaaacucCCGGUCAa | uccacgauacacucGGCCAGUc |
| NR_002838 | KC6 | circRNA | chr18 | 39039467 | 39039489 | - | 1 | 0 | AGO2 | 7mer-m8 | ucgcccuGAAACUC-CCGGUCAa | aauguauCUAUCUGUGGCCAGUg |
| NM_020964 | EPG5 | circRNA | chr18 | 43502548 | 43502566 | - | 3 | 0 | AGO1-4 | 8mer | ucgcccugaaacucCCGGUCAa | #NAME? |
| NM_001242804 | LOC100505549 | circRNA | chr18 | 55386602 | 55386627 | + | 1 | 0 | AGO1-4 | 7mer-m8 | ucgccCUGAAACUC----CCGGUCAa | ugccaGACAGUGGGCGCAGGCCAGUg |
| NM_021127 | PMAIP1 | circRNA | chr18 | 57570679 | 57570700 | + | 22 | 0 | AGO1,AGO1-4,AGO2,AGO3 | 8mer | ucgCCCUGAAACUCCCGGUCAa | ggaGGGUUACUUGGGGCCAGUa |
| NM_020854 | KIAA1468 | circRNA | chr18 | 59894616 | 59894642 | + | 2 | 0 | AGO1-4,AGO2 | 7mer-m8 | ucgccCUGAAAC-UC----CCGGUCAa | gaaaaGAUCUUGUAGAUGUGGCCAGUg |
| NM_001728 | BSG | circRNA | chr19 | 581397 | 581418 | + | 9 | 0 | AGO1-4,AGO2 | 8mer | ucgcccugaaacucCCGGUCAa | uggaggccgaccccGGCCAGUa |
| NM_198591 | BSG | circRNA | chr19 | 581397 | 581418 | + | 9 | 0 | AGO1-4,AGO2 | 8mer | ucgcccugaaacucCCGGUCAa | uggaggccgaccccGGCCAGUa |
| NM_004368 | CNN2 | circRNA | chr19 | 1036208 | 1036229 | + | 3 | 0 | AGO1-4,AGO2 | 7mer-m8 | ucgcccugaaacucCCGGUCAa | ccaccaugaaggcuGGCCAGUg |
| NM_019112 | ABCA7 | circRNA | chr19 | 1055128 | 1055145 | + | 1 | 0 | AGO2 | 7mer-m8 | ucgcCCUGAAACUCCCGGUCAa | ccaaGGUCUU----GGCCAGUg |
| NM_002695 | POLR2E | circRNA | chr19 | 1087928 | 1087948 | - | 2 | 0 | AGO2 | 7mer-m8 | ucGCCCUGAAACUCCCGGUCAa | agCUGUGGUUUG-CGGCCAGUu |
| NM_014963 | SBNO2 | circRNA | chr19 | 1117419 | 1117439 | - | 1 | 0 | AGO2 | 7mer-m8 | ucgcccuGAAACUCCCGGUCAa | caaguccCUGUG-GGGCCAGUu |
| NM_000156 | GAMT | circRNA | chr19 | 1397096 | 1397118 | - | 1 | 0 | AGO2 | 7mer-m8 | ucGCCCUGAAAC-UCCCGGUCAa | gcCGGCGCUGGGCUUGGCCAGUc |
| NM_003926 | MBD3 | circRNA | chr19 | 1577187 | 1577207 | - | 5 | 0 | AGO1-4,AGO2 | 7mer-m8 | ucgcCCUGAAACUCCCGGUCAa | gaguGGACUCUG-CGGCCAGUc |
| NM_001178002 | ATP8B3 | circRNA | chr19 | 1789041 | 1789065 | - | 1 | 0 | AGO1-4 | 7mer-m8 | ucgCCCUGAAACUC---CCGGUCAa | acaGCGACUUCGUGCUCGGCCAGUu |
| NM_032737 | LMNB2 | circRNA | chr19 | 2434863 | 2434884 | - | 2 | 0 | AGO1-4 | 7mer-m8 | ucgcccugaaaCUCCCGGUCAa | cagaacgacaaGGCGGCCAGUg |
| NM_003260 | TLE2 | circRNA | chr19 | 3011068 | 3011089 | - | 1 | 0 | AGO1-4 | 7mer-m8 | ucGCCCUGAAACUCCCGGUCAa | ccCGGGCCCAGCUCGGCCAGUc |
| NM_198969 | AES | circRNA | chr19 | 3057709 | 3057729 | - | 13 | 4 | AGO1-4,AGO2 | 7mer-m8 | ucGCCCUGAAACUCCCGGUCAa | aaUGUGAC-AAGUUGGCCAGUg |
| NM_020170 | NCLN | circRNA | chr19 | 3208590 | 3208613 | + | 2 | 0 | AGO1-4,AGO2 | 7mer-m8 | ucgccCUGAAAC--UCCCGGUCAa | auccaGGCUUUGCCACGGCCAGUu |
| NM_001080543 | C19orf29 | circRNA | chr19 | 3624032 | 3624053 | - | 2 | 0 | AGO1,AGO2 | 7mer-m8 | ucgcccugaaacUCCCGGUCAa | aggagcagucacGGGGCCAGUg |
| NM_001961 | EEF2 | circRNA | chr19 | 3982304 | 3982325 | - | 25 | 0 | AGO1,AGO1-4,AGO2 | 7mer-m8 | ucgcccugaaaCUCCCGGUCAa | ccgccaaggggGAGGGCCAGUu |
| NM_015898 | ZBTB7A | circRNA | chr19 | 4064191 | 4064212 | - | 1 | 0 | AGO2 | 7mer-m8 | ucgcccugaaACUCCCGGUCAa | auuggcugcgUGGCGGCCAGUc |
| NM_003025 | SH3GL1 | circRNA | chr19 | 4361649 | 4361671 | - | 7 | 0 | AGO1-4,AGO2 | 7mer-m8 | ucgCCCUG-AAACUCCCGGUCAa | gagGGCAUGCUGGACGGCCAGUc |
| NM_052972 | LRG1 | circRNA | chr19 | 4538651 | 4538672 | - | 1 | 0 | AGO1-4 | 7mer-m8 | ucGCCCUGAAACUCCCGGUCAa | gcCCGAAUUCCUGCGGCCAGUg |
| NM_014649 | SAFB2 | circRNA | chr19 | 5592779 | 5592797 | - | 1 | 0 | AGO1-4 | 8mer | ucGCCCUGAAACUCCCGGUCAa | auCGAGAC---CGGGGCCAGUa |
| NM_000064 | C3 | circRNA | chr19 | 6709831 | 6709853 | - | 1 | 0 | AGO2 | 7mer-m8 | ucgcccugaaacUC-CCGGUCAa | cuggugguaaaaAGCGGCCAGUc |
| NM_024690 | MUC16 | circRNA | chr19 | 9067762 | 9067782 | - | 1 | 0 | AGO2 | 7mer-m8 | ucGCCCUGAAACUCCCGGUCAa | ugCUGGCAUCUG-UGGCCAGUu |
| NM_001130031 | ZNF562 | circRNA | chr19 | 9762164 | 9762188 | - | 1 | 0 | AGO2 | 7mer-m8 | ucgCCCUGAAAC---UCCCGGUCAa | gcaGAGAUUUUGUUUAUGGCCAGUu |
| NM_152289 | ZNF561 | circRNA | chr19 | 9762164 | 9762188 | - | 1 | 0 | AGO2 | 7mer-m8 | ucgCCCUGAAAC---UCCCGGUCAa | gcaGAGAUUUUGUUUAUGGCCAGUu |
| NM_002566 | P2RY11 | circRNA | chr19 | 10222275 | 10222296 | + | 4 | 0 | AGO2 | 8mer | ucgcccugaaacuCCCGGUCAa | ugcucugcuggcuGGGCCAGUa |
| NM_002566 | P2RY11 | circRNA | chr19 | 10225024 | 10225046 | + | 2 | 0 | AGO2 | 7mer-m8 | ucGCCCU-GAAACUCCCGGUCAa | ugUGGCAGCGUUGGUGGCCAGUg |
| NM_002566 | P2RY11 | circRNA | chr19 | 10225924 | 10225945 | + | 8 | 0 | AGO1-4,AGO2 | 7mer-m8 | ucgcCCUGAAACUCCCGGUCAa | gcacGGAGCCAGAUGGCCAGUc |
| NM_004230 | S1PR2 | circRNA | chr19 | 10334312 | 10334333 | - | 1 | 0 | AGO1-4 | 7mer-m8 | ucGCCCUGAAACUCCCGGUCAa | agUGCGGUCCUGAGGGCCAGUc |
| NM_003331 | TYK2 | circRNA | chr19 | 10476217 | 10476238 | - | 2 | 1 | AGO1-4 | 8mer | ucgcccugaaACUCCCGGUCAa | uggcauccagUGGUGGCCAGUa |
| NM_007065 | CDC37 | circRNA | chr19 | 10514074 | 10514097 | - | 5 | 0 | AGO1-4 | 7mer-m8 | ucgcccUGAAAC--UCCCGGUCAa | accccaACAUCGACACGGCCAGUc |
| NM_001005360 | DNM2 | circRNA | chr19 | 10940998 | 10941019 | + | 8 | 0 | AGO1-4,AGO2 | 7mer-m8 | ucgcccugaaacucCCGGUCAa | ucagaucccaucucGGCCAGUu |
| NM_020812 | DOCK6 | circRNA | chr19 | 11347133 | 11347153 | - | 2 | 0 | AGO1-4,AGO2 | 7mer-m8 | ucGCCCUGAAACUCCCGGUCAa | agCAGGA-GCUGCGGGCCAGUc |
| hsa_circ_0000892 | hsa_circ_001760 | circRNA | chr19 | 11969298 | 11969326 | + | 1 | 0 | AGO1-4 | 7mer-m8 | ucgCCCUGA-AAC-----UC-CCGGUCAa | aggGGGUCUAUUGCUACAAGAGGCCAGUc |
| NM_144566 | ZNF700 | circRNA | chr19 | 12054146 | 12054166 | + | 2 | 0 | AGO1-4 | 7mer-m8 | ucgcCCUGAAACUCCCGGUCAa | gcaaGUAGUCU-AGGGCCAGUc |
| NM_001164276 | ZNF44 | circRNA | chr19 | 12357696 | 12357717 | - | 1 | 0 | AGO1-4 | 7mer-m8 | ucgcccugaaacuCCCGGUCAa | gagccaccacacuGGGCCAGUu |
| NM_014975 | MAST1 | circRNA | chr19 | 12978571 | 12978593 | + | 2 | 0 | AGO2 | 7mer-m8 | ucgcccugaaaCUC-CCGGUCAa | uuuccuggaggGAGAGGCCAGUc |
| NM_004461 | FARSA | circRNA | chr19 | 13033490 | 13033511 | - | 4 | 0 | AGO1-4,AGO2 | 7mer-m8 | ucgcccugaaacucCCGGUCAa | uccuuugcaucccuGGCCAGUg |
| NM_004343 | CALR | circRNA | chr19 | 13050268 | 13050289 | + | 12 | 0 | AGO1,AGO1-4,AGO2 | 7mer-m8 | ucgcccUGAAACUCCCGGUCAa | uuuuauGCUCUGUCGGCCAGUu |
| NM_030818 | CCDC130 | circRNA | chr19 | 13862555 | 13862576 | + | 1 | 0 | AGO1-4 | 7mer-m8 | ucgcccugaaacUCCCGGUCAa | caguguguucacAAGGCCAGUu |
| NM_078481 | CD97 | circRNA | chr19 | 14519218 | 14519237 | + | 5 | 0 | AGO1-4,AGO2 | 8mer | ucGCCCUGAAACUCCCGGUCAa | ccUGGCACCU--GUGGCCAGUa |
| NM_006145 | DNAJB1 | circRNA | chr19 | 14625690 | 14625711 | - | 8 | 0 | AGO1-4,AGO2 | 7mer-m8 | ucgcccugaaACUCCCGGUCAa | gucuguucccUGGAGGCCAGUc |
| NM_000435 | NOTCH3 | circRNA | chr19 | 15289689 | 15289710 | - | 1 | 0 | AGO1-4 | 7mer-m8 | ucgcccugaaACUCCCGGUCAa | caugccagcaUGGAGGCCAGUg |
| NM_000435 | NOTCH3 | circRNA | chr19 | 15299820 | 15299841 | - | 3 | 0 | AGO1-4 | 7mer-m8 | ucgcccugaaacucCCGGUCAa | gccucgaccgcauaGGCCAGUu |
| NM_014371 | AKAP8L | circRNA | chr19 | 15512145 | 15512164 | - | 1 | 0 | AGO1 | 7mer-m8 | ucgCCCUGAAACUCCCGGUCAa | augGGGGC--CCGGGGCCAGUg |
| NM_005234 | NR2F6 | circRNA | chr19 | 17343162 | 17343183 | - | 12 | 0 | AGO1,AGO1-4,AGO2 | 7mer-m8 | ucgcccugaaacuCCCGGUCAa | ggcccuacggcucGGGCCAGUg |
| NM_020959 | ANO8 | circRNA | chr19 | 17438317 | 17438338 | - | 2 | 0 | AGO1-4 | 7mer-m8 | ucgcccUGAAACUCCCGGUCAa | acugcuACUUAAUCGGCCAGUg |
| NM_005027 | PIK3R2 | circRNA | chr19 | 18266617 | 18266638 | + | 1 | 0 | AGO1-4 | 7mer-m8 | ucgcccugaaacUCCCGGUCAa | gcuucaaccaauGGGGCCAGUg |
| NM_001033930 | UBA52 | circRNA | chr19 | 18687950 | 18687969 | + | 1 | 0 | AGO2 | 7mer-m8 | ucGCCCUGAAACUCCCGGUCAa | gcCGGUGC-ACGA-GGCCAGUg |
| NM_001033930 | UBA52 | circRNA | chr19 | 18687995 | 18688016 | + | 1 | 0 | AGO1 | 7mer-m8 | ucGCCCUGAAACUCCCGGUCAa | ggCGGGACUUCCUGGGCCAGUu |
| NM_001105570 | NUDT19 | circRNA | chr19 | 33204253 | 33204279 | + | 1 | 0 | AGO1-4 | 7mer-m8 | ucGCCCUGAA-----ACUCCCGGUCAa | gaCGGGGUUUCACCAUGUUGGCCAGUc |
| NM_014686 | KIAA0355 | circRNA | chr19 | 34832797 | 34832821 | + | 3 | 0 | AGO1,AGO2 | 7mer-m8 | ucgcCCUGAAACU---CCCGGUCAa | ucucGGGCUUUAACAUGGGCCAGUc |
| NR_038396 | LOC100506469 | circRNA | chr19 | 36032438 | 36032457 | - | 2 | 0 | AGO1-4,AGO2 | 7mer-m8 | ucGCCCUGAAACUCCCGGUCAa | ucUGUGAUUCU--UGGCCAGUg |
| NM_001083961 | WDR62 | circRNA | chr19 | 36591705 | 36591725 | + | 5 | 0 | AGO1-4,AGO2 | 7mer-m8 | ucgcccugAAACUCCCGGUCAa | aggaaucaUCUGA-GGCCAGUg |
| hsa_circ_0050779 | hsa_circ_0050779 | circRNA | chr19 | 37808828 | 37808850 | + | 1 | 0 | AGO1-4 | 7mer-m8 | ucgcccugaaacUC-CCGGUCAa | guccgagugcacAGUGGCCAGUg |
| NM_004924 | ACTN4 | circRNA | chr19 | 39220331 | 39220352 | + | 16 | 0 | AGO1-4,AGO2 | 7mer-m8 | ucgcccugaaacUCCCGGUCAa | uuuaaccaaggaGGGGCCAGUg |
| hsa_circ_0000934 | hsa_circ_002015 | circRNA | chr19 | 40573238 | 40573257 | - | 3 | 0 | AGO1,AGO2 | 8mer | ucgcccUGAAACUCCCGGUCAa | gacauaACUU--AUGGCCAGUa |
| NM_053046 | EGLN2 | circRNA | chr19 | 41306943 | 41306964 | + | 2 | 0 | AGO2 | 7mer-m8 | ucGCCCUGA-AACUCCCGGUCAa | agUGGCAGUGGUGA-GGCCAGUg |
| NR_037791 | RAB4B-EGLN2 | circRNA | chr19 | 41306943 | 41306964 | + | 2 | 0 | AGO2 | 7mer-m8 | ucGCCCUGA-AACUCCCGGUCAa | agUGGCAGUGGUGA-GGCCAGUg |
| NM_053046 | EGLN2 | circRNA | chr19 | 41313758 | 41313778 | + | 4 | 1 | AGO1-4,AGO2 | 7mer-m8 | ucgcccUGAAACUCCCGGUCAa | acgcccACCUAG-UGGCCAGUc |
| NR_037791 | RAB4B-EGLN2 | circRNA | chr19 | 41313758 | 41313778 | + | 4 | 1 | AGO1-4,AGO2 | 7mer-m8 | ucgcccUGAAACUCCCGGUCAa | acgcccACCUAG-UGGCCAGUc |
| NM_001167867 | ATP5SL | circRNA | chr19 | 41938826 | 41938846 | - | 1 | 0 | AGO2 | 8mer | ucgcCCUGAAACUCCCGGUCAa | gguuGAAAUUUG-GGGCCAGUa |
| NM_015125 | CIC | circRNA | chr19 | 42793471 | 42793492 | + | 2 | 0 | AGO1-4,AGO2 | 7mer-m8 | ucgcccugaaacucCCGGUCAa | cguucucagcgugcGGCCAGUg |
| NM_015125 | CIC | circRNA | chr19 | 42795317 | 42795338 | + | 2 | 0 | AGO2 | 8mer | ucgcccugaaacuCCCGGUCAa | gaguggccccaauGGGCCAGUa |
| NM_006297 | XRCC1 | circRNA | chr19 | 44057636 | 44057657 | - | 4 | 0 | AGO1,AGO1-4,AGO2 | 7mer-m8 | ucgcccugaaacucCCGGUCAa | cagugaccaagcuuGGCCAGUu |
| NM_003370 | VASP | circRNA | chr19 | 46021313 | 46021334 | + | 3 | 0 | AGO1-4,AGO2 | 7mer-m8 | ucgcccugaaacUCCCGGUCAa | uuugccgccggcAUGGCCAGUg |
| hsa_circ_0000942 | hsa_circ_000899 | circRNA | chr19 | 46021313 | 46021334 | + | 3 | 0 | AGO1-4,AGO2 | 7mer-m8 | ucgcccugaaacUCCCGGUCAa | uuugccgccggcAUGGCCAGUg |
| NM_175875 | SIX5 | circRNA | chr19 | 46269882 | 46269903 | - | 1 | 0 | AGO1-4 | 7mer-m8 | ucgcccugaaacUCCCGGUCAa | uccucugcccccGGGGCCAGUg |
| NM_004819 | SYMPK | circRNA | chr19 | 46341783 | 46341804 | - | 4 | 0 | AGO1-4 | 7mer-m8 | ucgcccugaaacucCCGGUCAa | cagcgcagaucuccGGCCAGUc |
| NM_006247 | PPP5C | circRNA | chr19 | 46887056 | 46887077 | + | 5 | 0 | AGO1,AGO1-4,AGO2 | 7mer-m8 | ucgCCCUGAAACUCCCGGUCAa | gugGGGACACCCAUGGCCAGUu |
| hsa_circ_0000943 | hsa_circ_001101 | circRNA | chr19 | 47422003 | 47422024 | + | 4 | 0 | AGO1-4,AGO2 | 7mer-m8 | ucgcccugaaacUCCCGGUCAa | ccgagaaggaaaAGGGCCAGUg |
| NM_004491 | ARHGAP35 | circRNA | chr19 | 47506386 | 47506413 | + | 3 | 0 | AGO2 | 7mer-m8 | ucGCCCUGAAACUC------CCGGUCAa | cuCGGUCGUUUGAGGGGCAUGGCCAGUc |
| NM_015603 | CCDC9 | circRNA | chr19 | 47769992 | 47770013 | + | 1 | 0 | AGO1-4 | 7mer-m8 | ucgcccugaaacucCCGGUCAa | accgcaagcccacuGGCCAGUg |
| NM_007059 | KPTN | circRNA | chr19 | 47987223 | 47987244 | - | 1 | 0 | AGO2 | 7mer-m8 | ucgcccugaaacucCCGGUCAa | ccgacagaaaauccGGCCAGUg |
| NM_014601 | EHD2 | circRNA | chr19 | 48220046 | 48220069 | + | 2 | 0 | AGO1-4 | 8mer | ucGCCCUGA--AACUCCCGGUCAa | caUGGUGCUGGUGGCCGGCCAGUa |
| NM_004228 | CYTH2 | circRNA | chr19 | 48983182 | 48983201 | + | 2 | 0 | AGO2 | 7mer-m8 | ucGCCCUGAAACUCCCGGUCAa | ggCAGGAC--UGUGGGCCAGUg |
| NM_014203 | AP2A1 | circRNA | chr19 | 50302607 | 50302628 | + | 1 | 0 | AGO1-4 | 7mer-m8 | ucgcccugaaacuCCCGGUCAa | ccugcaaccagcuGGGCCAGUu |
| NM_014931 | PPP6R1 | circRNA | chr19 | 55741332 | 55741356 | - | 4 | 0 | AGO1-4,AGO2 | 7mer-m8 | ucGCCCUGAAACU---CCCGGUCAa | ccCUGCACUUUCAACCAGGCCAGUc |
| NM_014931 | PPP6R1 | circRNA | chr19 | 55752675 | 55752696 | - | 3 | 0 | AGO1-4,AGO2 | 7mer-m8 | ucgcccugaaacucCCGGUCAa | guggucaagcuccuGGCCAGUg |
| NM_032430 | BRSK1 | circRNA | chr19 | 55816132 | 55816153 | + | 2 | 0 | AGO2 | 7mer-m8 | ucgcccugaaacuCCCGGUCAa | cugcacacgccccGGGCCAGUc |
| NM_001136134 | RPL28 | circRNA | chr19 | 55899434 | 55899460 | + | 3 | 0 | AGO1-4 | 7mer-m8 | ucgccCUGAAACUC-----CCGGUCAa | gaucaGGCUUGGGGAGACUGGCCAGUg |
| NM_014501 | UBE2S | circRNA | chr19 | 55912953 | 55912970 | - | 6 | 1 | AGO1-4,AGO2 | 7mer-m8 | ucGCCCUGAAACUCCCGGUCAa | guCGGGCCCU----GGCCAGUg |
| NM_014501 | UBE2S | circRNA | chr19 | 55913896 | 55913917 | - | 1 | 0 | AGO2 | 7mer-m8 | ucgcccUGAAACUCCCGGUCAa | gagcaaACUGCUGUGGCCAGUu |
| NM_016535 | ZNF581 | circRNA | chr19 | 56147550 | 56147571 | + | 3 | 0 | AGO1-4,AGO2 | 7mer-m8 | ucgcccugaaacucCCGGUCAa | aucugucagggucaGGCCAGUg |
| NM_016535 | ZNF581 | circRNA | chr19 | 56156152 | 56156172 | + | 1 | 0 | AGO1-4 | 7mer-m8 | ucgCCCUGAAACUCCCGGUCAa | agaGGGAGCCAG-GGGCCAGUg |
| NM_007279 | U2AF2 | circRNA | chr19 | 56175015 | 56175036 | + | 11 | 0 | AGO1,AGO1-4,AGO2 | 7mer-m8 | ucgcccuGAAACUCCCGGUCAa | gcaucauCUUCCAGGGCCAGUc |
| NM_001204818 | LOC100293516 | circRNA | chr19 | 58352595 | 58352616 | + | 2 | 0 | AGO1-4 | 7mer-m8 | ucgcccugaaaCUCCCGGUCAa | uuacuccagcaGGAGGCCAGUc |
| NM_032828 | ZNF587 | circRNA | chr19 | 58352595 | 58352616 | + | 2 | 0 | AGO1-4 | 7mer-m8 | ucgcccugaaaCUCCCGGUCAa | uuacuccagcaGGAGGCCAGUc |
| NM_133502 | ZNF274 | circRNA | chr19 | 58698357 | 58698378 | + | 1 | 0 | AGO2 | 7mer-m8 | ucGCCCUGAAACUCCCGGUCAa | ggCAGAAGAUUUCUGGCCAGUg |
| NM_014480 | ZNF544 | circRNA | chr19 | 58760369 | 58760390 | + | 2 | 0 | AGO1-4 | 7mer-m8 | ucgcccugaaacucCCGGUCAa | gcagaugaaaacuaGGCCAGUg |
| NM_021089 | ZNF8 | circRNA | chr19 | 58824939 | 58824960 | + | 1 | 0 | AGO1-4 | 7mer-m8 | ucgcccugaaacucCCGGUCAa | gagccaccaugcccGGCCAGUu |
| NM_024958 | NRSN2 | circRNA | chr20 | 334184 | 334206 | + | 2 | 0 | AGO1-4 | 7mer-m8 | ucGCCCUGAAACUC-CCGGUCAa | uuCCGCAAUGCCAGUGGCCAGUc |
| NM_003091 | SNRPB | circRNA | chr20 | 2444420 | 2444441 | - | 6 | 0 | AGO1-4,AGO2 | 7mer-m8 | ucGCCCUGAAACUCCCGGUCAa | ugCAGGACUUGCUGGGCCAGUc |
| NR_027700 | NOP56 | circRNA | chr20 | 2636792 | 2636811 | + | 13 | 0 | AGO1-4,AGO2 | 7mer-m8 | ucGCCCUGAAACUCCCGGUCAa | uaUGGGG--GUGAUGGCCAGUc |
| NM_021826 | FASTKD5 | circRNA | chr20 | 3129449 | 3129469 | - | 5 | 0 | AGO1,AGO1-4,AGO2 | 8mer | ucgcccuGAAACUCCCGGUCAa | cuucuucCUCU-AAGGCCAGUa |
| NM_139321 | ATRN | circRNA | chr20 | 3564679 | 3564698 | + | 1 | 0 | AGO2 | 7mer-m8 | ucgcccuGAAACUCCCGGUCAa | uccuuccCUUU--UGGCCAGUg |
| NM_000311 | PRNP | circRNA | chr20 | 4680843 | 4680864 | + | 17 | 0 | AGO1,AGO1-4,AGO2 | 8mer | ucgcccugaaacucCCGGUCAa | cguugcuaaugccaGGCCAGUa |
| NM_005116 | SLC23A2 | circRNA | chr20 | 4866533 | 4866553 | - | 1 | 0 | AGO1-4 | 7mer-m8 | ucgcccugAAACUCCCGGUCAa | uaccccugUUUCA-GGCCAGUg |
| NM_203327 | SLC23A2 | circRNA | chr20 | 4866533 | 4866553 | - | 1 | 0 | AGO1-4 | 7mer-m8 | ucgcccugAAACUCCCGGUCAa | uaccccugUUUCA-GGCCAGUg |
| NM_000214 | JAG1 | circRNA | chr20 | 10629731 | 10629752 | - | 2 | 0 | AGO2 | 7mer-m8 | ucgccCUGAAACUCCCGGUCAa | uuaauGACUGCCUUGGCCAGUg |
| NM_032985 | SEC23B | circRNA | chr20 | 18506491 | 18506510 | + | 13 | 0 | AGO1,AGO1-4,AGO2,AGO3 | 8mer | ucgCCCUGAAACUCCCGGUCAa | agaGGGAC--CCAUGGCCAGUa |
| NM_000099 | CST3 | circRNA | chr20 | 23612661 | 23612681 | - | 2 | 0 | AGO1-4,AGO2 | 7mer-m8 | ucgcccugaaACUCCCGGUCAa | ugggcccccaUGA-GGCCAGUg |
| NM_002862 | PYGB | circRNA | chr20 | 25277520 | 25277546 | + | 3 | 0 | AGO1-4,AGO2 | 7mer-m8 | ucGCCCUGAAACU-----CCCGGUCAa | cuCUGGAUUCUGGGGUCUGGGCCAGUg |
| NM_015338 | ASXL1 | circRNA | chr20 | 31023747 | 31023768 | + | 5 | 0 | AGO1-4 | 8mer | ucgcccugaaACUCCCGGUCAa | gauucccuacUGCUGGCCAGUa |
| NM_080616 | C20orf112 | circRNA | chr20 | 31034843 | 31034861 | - | 6 | 0 | AGO1-4,AGO2 | 8mer | ucgcCCUGAAACUCCCGGUCAa | auguGGGCU---GGGGCCAGUa |
| NM_080616 | C20orf112 | circRNA | chr20 | 31095318 | 31095338 | - | 2 | 0 | AGO1-4 | 7mer-m8 | ucgcccUGAAACUCCCGGUCAa | cauguuAC-UUGUAGGCCAGUu |
| NM_005093 | CBFA2T2 | circRNA | chr20 | 32194868 | 32194886 | + | 1 | 0 | AGO1-4 | 7mer-m8 | ucgcCCUGAAACUCCCGGUCAa | uggaGGAC--CGA-GGCCAGUg |
| NM_014071 | NCOA6 | circRNA | chr20 | 33345355 | 33345376 | - | 3 | 0 | AGO1-4,AGO2 | 7mer-m8 | ucgcccugaaacucCCGGUCAa | agaugagcaacccaGGCCAGUu |
| NM_001076552 | ACSS2 | circRNA | chr20 | 33515081 | 33515103 | + | 1 | 0 | AGO2 | 7mer-m8 | ucGCCCUGA-AACUCCCGGUCAa | cuCAGGAGUGCUGAGGGCCAGUg |
| NM_015638 | TRPC4AP | circRNA | chr20 | 33590913 | 33590935 | - | 3 | 1 | AGO1-4,AGO2 | 7mer-m8 | ucGCCCUGAAAC-UCCCGGUCAa | cuCGGGAGGCUGCUGGGCCAGUg |
| NM_007186 | CEP250 | circRNA | chr20 | 34092735 | 34092756 | + | 1 | 0 | AGO1-4 | 7mer-m8 | ucgcccugaaacUCCCGGUCAa | cuagcgcagaccAAGGCCAGUg |
| NR_037570 | NFS1 | circRNA | chr20 | 34286445 | 34286464 | - | 2 | 0 | AGO1-4 | 7mer-m8 | ucGCCCUGAAACUCCCGGUCAa | ccCCGGAGGU--GGGGCCAGUg |
| NM_080627 | KIAA0889 | circRNA | chr20 | 35412663 | 35412682 | - | 3 | 0 | AGO1-4,AGO2 | 7mer-m8 | ucGCCCUGAAACUCCCGGUCAa | agUGGAGC--UGUGGGCCAGUu |
| NM_014657 | TTI1 | circRNA | chr20 | 36640309 | 36640326 | - | 4 | 0 | AGO1,AGO1-4,AGO2 | 7mer-m8 | ucgcCCUGAAACUCCCGGUCAa | ggaaGGAAUU----GGCCAGUu |
| NM_004613 | TGM2 | circRNA | chr20 | 36775148 | 36775169 | - | 4 | 0 | AGO1-4 | 7mer-m8 | ucGCCCUGAAACUCCCGGUCAa | agCGCGUCAAGUAUGGCCAGUg |
| NM_004613 | TGM2 | circRNA | chr20 | 36789852 | 36789872 | - | 5 | 0 | AGO1-4 | 7mer-m8 | ucGCCCUGAAACUCCCGGUCAa | gcCGCAACUACGA-GGCCAGUg |
| NR_034009 | SRSF6 | circRNA | chr20 | 42092113 | 42092133 | + | 6 | 0 | AGO1,AGO1-4,AGO2 | 7mer-m8 | ucgCCCUGAAACUCCCGGUCAa | guaGUGCCUUU-AUGGCCAGUu |
| NM_024331 | TTPAL | circRNA | chr20 | 43118553 | 43118574 | + | 4 | 0 | AGO1-4,AGO2 | 7mer-m8 | ucgcccugaaacucCCGGUCAa | aaaaccaccuguuuGGCCAGUg |
| NM_024331 | TTPAL | circRNA | chr20 | 43120516 | 43120537 | + | 5 | 0 | AGO2 | 7mer-m8 | ucgcccUGAAACUCCCGGUCAa | uacacuGCAUUCCUGGCCAGUc |
| NM_003064 | SLPI | circRNA | chr20 | 43881705 | 43881726 | - | 2 | 0 | AGO1-4,AGO2 | 7mer-m8 | ucGCCCUGAAACUCCCGGUCAa | ucUGUGAGAUGGAUGGCCAGUg |
| NM_022104 | PCIF1 | circRNA | chr20 | 44569458 | 44569479 | + | 1 | 0 | AGO2 | 7mer-m8 | ucgcccugaaacucCCGGUCAa | cagugacacccacaGGCCAGUc |
| NM_181659 | NCOA3 | circRNA | chr20 | 46251010 | 46251031 | + | 4 | 0 | AGO1-4 | 7mer-m8 | ucgcccugaaacucCCGGUCAa | aacuuggauccacuGGCCAGUg |
| NM_001161841 | SULF2 | circRNA | chr20 | 46286422 | 46286442 | - | 11 | 0 | AGO1-4,AGO2 | 7mer-m8 | ucgcccUGAAACUCCCGGUCAa | auaaacGCUCUG-UGGCCAGUg |
| NM_001161841 | SULF2 | circRNA | chr20 | 46311828 | 46311849 | - | 2 | 0 | AGO1-4 | 7mer-m8 | ucgcccugaaacucCCGGUCAa | acgguuaccacaucGGCCAGUu |
| NM_001161841 | SULF2 | circRNA | chr20 | 46318959 | 46318980 | - | 6 | 0 | AGO1-4 | 7mer-m8 | ucgcccugaaacucCCGGUCAa | gauguacccgcacaGGCCAGUc |
| NM_020820 | PREX1 | circRNA | chr20 | 47258761 | 47258782 | - | 6 | 0 | AGO1-4 | 7mer-m8 | ucgcccuGAAACUCCCGGUCAa | ugcucucCUCAAGGGGCCAGUc |
| NM_021035 | ZNFX1 | circRNA | chr20 | 47886580 | 47886604 | - | 3 | 0 | AGO1-4 | 7mer-m8 | ucgccCUGAAACU---CCCGGUCAa | uuaauGUCUUAGAUCCUGGCCAGUg |
| NM_015339 | ADNP | circRNA | chr20 | 49510031 | 49510054 | - | 21 | 1 | AGO1,AGO1-4,AGO2,AGO3 | 7mer-m8 | ucgcccuGAAACU--CCCGGUCAa | cucuucuCUCUCAUCGGGCCAGUu |
| NM_000782 | CYP24A1 | circRNA | chr20 | 52790344 | 52790365 | - | 2 | 0 | AGO1-4 | 7mer-m8 | ucgcccugaaacucCCGGUCAa | cuuccagccccugcGGCCAGUg |
| NM_020182 | PMEPA1 | circRNA | chr20 | 56225455 | 56225477 | - | 1 | 0 | AGO1-4 | 7mer-m8 | ucGCCCU-GAAACUCCCGGUCAa | uuUGGGACCCACCGGGGCCAGUg |
| NM_199169 | PMEPA1 | circRNA | chr20 | 56225455 | 56225477 | - | 1 | 0 | AGO1-4 | 7mer-m8 | ucGCCCU-GAAACUCCCGGUCAa | uuUGGGACCCACCGGGGCCAGUg |
| NM_020673 | RAB22A | circRNA | chr20 | 56941710 | 56941730 | + | 2 | 0 | AGO2 | 7mer-m8 | ucgcccUGAAACUCCCGGUCAa | uaagaaACACAGA-GGCCAGUg |
| NM_020673 | RAB22A | circRNA | chr20 | 56941779 | 56941800 | + | 2 | 0 | AGO1-4 | 7mer-m8 | ucgcccugaaacUCCCGGUCAa | uaccgugggaacACGGCCAGUu |
| NM_004738 | VAPB | circRNA | chr20 | 57022457 | 57022482 | + | 2 | 0 | AGO1-4,AGO2 | 7mer-m8 | ucgccCUGAAA---CUC-CCGGUCAa | aaauuGAUUUUUACCAGUGGCCAGUu |
| NM_001001433 | STX16 | circRNA | chr20 | 57251976 | 57251998 | + | 3 | 0 | AGO1-4 | 7mer-m8 | ucgcccUGAAACU-CCCGGUCAa | uaacacAUUUAGAUGGGCCAGUu |
| NM_001001433 | STX16 | circRNA | chr20 | 57253969 | 57253990 | + | 1 | 0 | AGO1-4 | 7mer-m8 | ucgcccugaaacucCCGGUCAa | guaacgaucacccaGGCCAGUc |
| NM_007002 | ADRM1 | circRNA | chr20 | 60883077 | 60883091 | + | 1 | 0 | AGO1-4 | 7mer-m8 | ucgcCCUGAAACUCCCGGUCAa | #NAME? |
| NM_005560 | LAMA5 | circRNA | chr20 | 60903300 | 60903324 | - | 2 | 0 | AGO1-4 | 7mer-m8 | ucGCCCUGAAAC--UC-CCGGUCAa | ccUGUGACACAGACAGCGGCCAGUg |
| NM_005560 | LAMA5 | circRNA | chr20 | 60903977 | 60903998 | - | 1 | 0 | AGO2 | 7mer-m8 | ucgcccuGAAACUCCCGGUCAa | gugagccCUUCGGGGGCCAGUg |
| NM_005560 | LAMA5 | circRNA | chr20 | 60937601 | 60937608 | - | 2 | 0 | AGO1-4,AGO2 | 8mer | ucgcccugaaacucCCGGUCAa | #NAME? |
| NM_001024 | RPS21 | circRNA | chr20 | 60962912 | 60962933 | + | 25 | 0 | AGO1,AGO1-4,AGO2 | 7mer-m8 | ucgcccugAAACUCCCGGUCAa | caggcaggUUUAAUGGCCAGUu |
| NM_007346 | OGFR | circRNA | chr20 | 61444366 | 61444387 | + | 3 | 0 | AGO1-4,AGO2 | 7mer-m8 | ucGCCCUGAAACUCCCGGUCAa | gaCAGUGCUGCGGUGGCCAGUg |
| NM_033081 | DIDO1 | circRNA | chr20 | 61542346 | 61542366 | - | 2 | 1 | AGO1-4,AGO2 | 7mer-m8 | ucgcCCUGAAACUCCCGGUCAa | cuguGGGCUCCGA-GGCCAGUg |
| NM_080796 | DIDO1 | circRNA | chr20 | 61542346 | 61542366 | - | 2 | 1 | AGO1-4,AGO2 | 7mer-m8 | ucgcCCUGAAACUCCCGGUCAa | cuguGGGCUCCGA-GGCCAGUg |
| NM_020062 | SLC2A4RG | circRNA | chr20 | 62373297 | 62373320 | + | 2 | 0 | AGO1-4 | 7mer-m8 | ucGCCCUGAAACU--CCCGGUCAa | acUGGGGAUGGGACCUGGCCAGUg |
| NM_025219 | DNAJC5 | circRNA | chr20 | 62563747 | 62563768 | + | 5 | 0 | AGO1-4,AGO2 | 7mer-m8 | ucGCCCUGAAACUCCCGGUCAa | agCUGGAUUGAUGGGGCCAGUc |
| NM_003489 | NRIP1 | circRNA | chr21 | 16334991 | 16335012 | - | 8 | 0 | AGO1,AGO1-4,AGO2 | 7mer-m8 | ucgcccugaaacucCCGGUCAa | cauaaaauuccacuGGCCAGUu |
| TCONS_l2_00017148 | TCONS_l2_00017148 | circRNA | chr21 | 16334991 | 16335012 | - | 8 | 0 | AGO1,AGO1-4,AGO2 | 7mer-m8 | ucgcccugaaacucCCGGUCAa | cauaaaauuccacuGGCCAGUu |
| NM_004540 | NCAM2 | circRNA | chr21 | 22645451 | 22645473 | + | 1 | 0 | AGO1-4 | 8mer | ucgcccugaaACUC-CCGGUCAa | guauauuggcUGAGUGGCCAGUa |
| NM_004540 | NCAM2 | circRNA | chr21 | 22651353 | 22651372 | + | 2 | 0 | AGO1-4 | 8mer | ucgcccUGAAACUCCCGGUCAa | uauacaACUAU--UGGCCAGUa |
| NM_206866 | BACH1 | circRNA | chr21 | 30725230 | 30725251 | + | 1 | 0 | AGO1-4 | 7mer-m8 | ucgcccugaaacucCCGGUCAa | caccguguuggccaGGCCAGUc |
| NM_058187 | C21orf63 | circRNA | chr21 | 33887472 | 33887493 | + | 2 | 0 | AGO2 | 7mer-m8 | ucgcccugaaacuCCCGGUCAa | ucccaagaaacauGGGCCAGUu |
| NM_175085 | GART | circRNA | chr21 | 34896656 | 34896679 | - | 2 | 0 | AGO1-4 | 7mer-m8 | ucgcccUGAAAC--UCCCGGUCAa | uucaccAUGUUGCCAAGGCCAGUc |
| NM_001136005 | GART | circRNA | chr21 | 34904728 | 34904749 | - | 1 | 0 | AGO1-4 | 7mer-m8 | ucgccCUGAAACUCCCGGUCAa | gcuuuGGUUGUGAAGGCCAGUg |
| NM_001136006 | GART | circRNA | chr21 | 34904728 | 34904749 | - | 1 | 0 | AGO1-4 | 7mer-m8 | ucgccCUGAAACUCCCGGUCAa | gcuuuGGUUGUGAAGGCCAGUg |
| NM_175085 | GART | circRNA | chr21 | 34904728 | 34904749 | - | 1 | 0 | AGO1-4 | 7mer-m8 | ucgccCUGAAACUCCCGGUCAa | gcuuuGGUUGUGAAGGCCAGUg |
| NM_138927 | SON | circRNA | chr21 | 34941293 | 34941317 | + | 3 | 1 | AGO1,AGO1-4,AGO2 | 7mer-m8 | ucGCCCUGA---AACUCCCGGUCAa | agCUGAACUCUAUUCCUGGCCAGUu |
| NM_003024 | ITSN1 | circRNA | chr21 | 35186288 | 35186309 | + | 2 | 0 | AGO1-4,AGO2 | 7mer-m8 | ucgcccugaaACUCCCGGUCAa | ccguuccaagUGCCGGCCAGUu |
| NM_006933 | SLC5A3 | circRNA | chr21 | 35475431 | 35475452 | + | 3 | 0 | AGO1-4,AGO2 | 8mer | ucgcccugaaacUCCCGGUCAa | cccaaacuggcaAAGGCCAGUa |
| NM_032476 | MRPS6 | circRNA | chr21 | 35475431 | 35475452 | + | 3 | 0 | AGO1-4,AGO2 | 8mer | ucgcccugaaacUCCCGGUCAa | cccaaacuggcaAAGGCCAGUa |
| hsa_circ_0001186 | hsa_circ_001113 | circRNA | chr21 | 35475431 | 35475452 | + | 3 | 0 | AGO1-4,AGO2 | 8mer | ucgcccugaaacUCCCGGUCAa | cccaaacuggcaAAGGCCAGUa |
| NR_030414 | MIR802 | circRNA | chr21 | 37168843 | 37168864 | - | 1 | 0 | AGO1-4 | 7mer-m8 | ucgcCCUGAAACUCCCGGUCAa | auguGGAAGGUCCUGGCCAGUg |
| NM_004571 | PKNOX1 | circRNA | chr21 | 44448890 | 44448911 | + | 1 | 0 | AGO1-4 | 7mer-m8 | ucgcccugaaacucCCGGUCAa | aacugcucagaaccGGCCAGUu |
| NM_015056 | RRP1B | circRNA | chr21 | 45114590 | 45114611 | + | 1 | 0 | AGO2 | 7mer-m8 | ucgcccugaaacucCCGGUCAa | uuucagcaaaacgcGGCCAGUc |
| hsa_circ_0001199 | hsa_circ_001854 | circRNA | chr21 | 45114590 | 45114611 | + | 1 | 0 | AGO2 | 7mer-m8 | ucgcccugaaacucCCGGUCAa | uuucagcaaaacgcGGCCAGUc |
| NM_005049 | PWP2 | circRNA | chr21 | 45540881 | 45540902 | + | 1 | 1 | AGO1 | 7mer-m8 | ucgcccugaaacucCCGGUCAa | augaaguccguccuGGCCAGUg |
| NM_004339 | PTTG1IP | circRNA | chr21 | 46271389 | 46271407 | - | 8 | 0 | AGO1-4,AGO2 | 7mer-m8 | ucgccCUGAAACUCCCGGUCAa | cuccuGACCUU---GGCCAGUg |
| NM_058190 | FAM207A | circRNA | chr21 | 46396615 | 46396635 | + | 3 | 0 | AGO1-4,AGO2 | 7mer-m8 | ucGCCCUGAAACUCCCGGUCAa | uuCAGGA-GCUGCUGGCCAGUc |
| NR_027673 | ADARB1 | circRNA | chr21 | 46642908 | 46642929 | + | 3 | 0 | AGO1-4,AGO2 | 8mer | ucgcccugaaacUCCCGGUCAa | cacagauaauacAUGGCCAGUa |
| NM_001848 | COL6A1 | circRNA | chr21 | 47423526 | 47423547 | + | 5 | 1 | AGO1-4 | 7mer-m8 | ucgcccugaaacucCCGGUCAa | aacuacacggcccuGGCCAGUg |
| NM_001242865 | PRMT2 | circRNA | chr21 | 48069487 | 48069508 | + | 1 | 0 | AGO1-4 | 7mer-m8 | ucgcccugaaACUCCCGGUCAa | guguacgcggUGGAGGCCAGUg |
| NM_001242866 | PRMT2 | circRNA | chr21 | 48069487 | 48069508 | + | 1 | 0 | AGO1-4 | 7mer-m8 | ucgcccugaaACUCCCGGUCAa | guguacgcggUGGAGGCCAGUg |
| NM_206962 | PRMT2 | circRNA | chr21 | 48069487 | 48069508 | + | 1 | 0 | AGO1-4 | 7mer-m8 | ucgcccugaaACUCCCGGUCAa | guguacgcggUGGAGGCCAGUg |
| NM_015241 | MICAL3 | circRNA | chr22 | 18482788 | 18482809 | - | 1 | 0 | AGO1-4 | 7mer-m8 | ucgcccugaaacucCCGGUCAa | ccuggccgcacacaGGCCAGUu |
| NM_001178010 | CDC45 | circRNA | chr22 | 19470299 | 19470320 | + | 1 | 0 | AGO2 | 7mer-m8 | ucgcccugaaacucCCGGUCAa | gugugacacccauaGGCCAGUc |
| NM_006440 | TXNRD2 | circRNA | chr22 | 19882227 | 19882248 | - | 2 | 0 | AGO1-4 | 8mer | ucgcccugaaacucCCGGUCAa | gagccaccgcaccuGGCCAGUa |
| NM_152906 | C22orf25 | circRNA | chr22 | 20052670 | 20052691 | + | 2 | 0 | AGO1-4,AGO2 | 8mer | ucgcccugaaacucCCGGUCAa | ucucaugugcuucuGGCCAGUa |
| NM_022727 | TRMT2A | circRNA | chr22 | 20102321 | 20102342 | - | 1 | 0 | AGO1-4 | 7mer-m8 | ucgCCCUGAAACUCCCGGUCAa | gcaGGGCCAGGCAGGGCCAGUg |
| NM_005207 | CRKL | circRNA | chr22 | 21304024 | 21304045 | + | 9 | 0 | AGO1-4,AGO2 | 7mer-m8 | ucgcCCUGAAACUCCCGGUCAa | ggauGAAUAUAAAUGGCCAGUg |
| NM_005207 | CRKL | circRNA | chr22 | 21305334 | 21305351 | + | 7 | 0 | AGO1-4,AGO2 | 7mer-m8 | ucgCCCUGAAACUCCCGGUCAa | gagGGCACUU----GGCCAGUu |
| NM_005207 | CRKL | circRNA | chr22 | 21306336 | 21306354 | + | 1 | 0 | AGO1-4 | 7mer-m8 | ucgcccuGAAACUCCCGGUCAa | guuugccCUUU---GGCCAGUg |
| NM_006767 | LZTR1 | circRNA | chr22 | 21342359 | 21342383 | + | 1 | 0 | AGO2 | 7mer-m8 | ucgcccUGAAAC--U-CCCGGUCAa | aauacaAGUUUGCAACUGGCCAGUg |
| NM_030573 | THAP7 | circRNA | chr22 | 21354472 | 21354493 | - | 2 | 0 | AGO2 | 7mer-m8 | ucgcccugaaacUCCCGGUCAa | cccucucgaaccACGGCCAGUc |
| NM_015094 | HIC2 | circRNA | chr22 | 21803863 | 21803883 | + | 12 | 0 | AGO1-4,AGO2 | 7mer-m8 | ucgcccUGAAACUCCCGGUCAa | ucagcaGCUGU-AAGGCCAGUc |
| NM_014634 | PPM1F | circRNA | chr22 | 22288535 | 22288556 | - | 1 | 0 | AGO1-4 | 7mer-m8 | ucGCCCUGAAACUCCCGGUCAa | uuUGGGAAGUCGCCGGCCAGUg |
| NM_014634 | PPM1F | circRNA | chr22 | 22300378 | 22300399 | - | 1 | 0 | AGO1-4 | 7mer-m8 | ucgcccugaaacUCCCGGUCAa | aagagcagcccaAUGGCCAGUg |
| NM_001178126 | IGLL5 | circRNA | chr22 | 23223391 | 23223412 | + | 1 | 0 | AGO1-4 | 7mer-m8 | ucgcccugaaacucCCGGUCAa | aucagcagaagccaGGCCAGUc |
| NR_027426 | LOC648691 | circRNA | chr22 | 23223391 | 23223412 | + | 1 | 0 | AGO1-4 | 7mer-m8 | ucgcccugaaacucCCGGUCAa | aucagcagaagccaGGCCAGUc |
| NM_004327 | BCR | circRNA | chr22 | 23573169 | 23573190 | + | 1 | 0 | AGO1-4 | 7mer-m8 | ucgcccugaaacucCCGGUCAa | cucaccaugcaccaGGCCAGUu |
| NM_213720 | CHCHD10 | circRNA | chr22 | 24108165 | 24108185 | - | 17 | 0 | AGO1,AGO1-4,AGO2 | 7mer-m8 | ucgccCUGAAACUCCCGGUCAa | gugcaGAC-UCGGGGGCCAGUc |
| NM_015330 | SPECC1L | circRNA | chr22 | 24718640 | 24718662 | + | 3 | 0 | AGO1-4 | 7mer-m8 | ucgcccugaaacUC-CCGGUCAa | auacaaagccacAGUGGCCAGUg |
| NM_031444 | C22orf13 | circRNA | chr22 | 24937751 | 24937768 | - | 1 | 0 | AGO1-4 | 7mer-m8 | ucgCCCUGAAACUCCCGGUCAa | aaaGGGACUU----GGCCAGUg |
| NM_004175 | SNRPD3 | circRNA | chr22 | 24968239 | 24968261 | + | 3 | 0 | AGO2 | 7mer-m8 | ucgcccUGA-AACUCCCGGUCAa | uaagcuGCUGUUCCUGGCCAGUu |
| NM_012399 | PITPNB | circRNA | chr22 | 28248989 | 28249009 | - | 1 | 0 | AGO2 | 7mer-m8 | ucgcccUGAAACUCCCGGUCAa | gagcaaAGUUACA-GGCCAGUu |
| NM_001145418 | TTC28 | circRNA | chr22 | 28559512 | 28559533 | - | 2 | 0 | AGO1,AGO1-4 | 7mer-m8 | ucgcccugaaacucCCGGUCAa | uaccccaaugcacuGGCCAGUc |
| NM_005080 | XBP1 | circRNA | chr22 | 29192160 | 29192183 | - | 6 | 0 | AGO1-4,AGO2 | 7mer-m8 | ucgCCCUGA---AACUCCCGGUCAa | cagGGGAAUGAAGUGA-GGCCAGUg |
| NM_032204 | ASCC2 | circRNA | chr22 | 30230305 | 30230326 | - | 1 | 0 | AGO1-4 | 7mer-m8 | ucgcccuGAAACUCCCGGUCAa | cacccuuCUUCCCUGGCCAGUc |
| NM_001204240 | TBC1D10A | circRNA | chr22 | 30689707 | 30689728 | - | 2 | 0 | AGO1-4,AGO2 | 8mer | ucgccCUGAAACUCCCGGUCAa | ucaaaGCCUGCCAGGGCCAGUa |
| NR_036550 | PES1 | circRNA | chr22 | 30972879 | 30972902 | - | 4 | 0 | AGO1-4,AGO2 | 7mer-m8 | ucgcccuGAAACU--CCCGGUCAa | ucaugcuCUCUGGCUGGGCCAGUg |
| NM_004147 | DRG1 | circRNA | chr22 | 31823048 | 31823069 | + | 4 | 0 | AGO1,AGO1-4,AGO2 | 7mer-m8 | ucgcccugaaacUCCCGGUCAa | acaccaaacccaAAGGCCAGUu |
| NM_001164502 | EIF4ENIF1 | circRNA | chr22 | 31838951 | 31838972 | - | 1 | 0 | AGO1-4 | 7mer-m8 | ucgcccUGAAACUCCCGGUCAa | gaggauACUCAGAAGGCCAGUg |
| NM_019843 | EIF4ENIF1 | circRNA | chr22 | 31838951 | 31838972 | - | 1 | 0 | AGO1-4 | 7mer-m8 | ucgcccUGAAACUCCCGGUCAa | gaggauACUCAGAAGGCCAGUg |
| NM_173566 | PRR14L | circRNA | chr22 | 32077521 | 32077543 | - | 6 | 0 | AGO1-4,AGO2 | 8mer | ucgcCCUGA-AACUCCCGGUCAa | ccuuGGGCUGGUCUGGGCCAGUa |
| NM_002405 | MFNG | circRNA | chr22 | 37876242 | 37876263 | - | 1 | 0 | AGO1-4 | 7mer-m8 | ucGCCCUGAAACUCCCGGUCAa | uuCGACACCUUCUUGGCCAGUg |
| NM_016091 | EIF3L | circRNA | chr22 | 38247415 | 38247436 | + | 12 | 0 | AGO1-4,AGO2 | 7mer-m8 | ucgcccugaaacucCCGGUCAa | guguaugagcuacaGGCCAGUc |
| NM_001098504 | DDX17 | circRNA | chr22 | 38880129 | 38880150 | - | 15 | 0 | AGO1-4,AGO2 | 7mer-m8 | ucgcccugaaacucCCGGUCAa | uuugucuaagcacuGGCCAGUc |
| NM_014876 | JOSD1 | circRNA | chr22 | 39083683 | 39083702 | - | 4 | 0 | AGO1-4,AGO2 | 7mer-m8 | ucgcCCUGAAACUCCCGGUCAa | cuguGCACUU--AAGGCCAGUg |
| NM_001024843 | TNRC6B | circRNA | chr22 | 40669453 | 40669475 | + | 2 | 0 | AGO1-4 | 7mer-m8 | ucgCCCUGAAACU-CCCGGUCAa | gggGGGAGAGUGACGGGCCAGUc |
| NM_001162501 | TNRC6B | circRNA | chr22 | 40669453 | 40669475 | + | 2 | 0 | AGO1-4 | 7mer-m8 | ucgCCCUGAAACU-CCCGGUCAa | gggGGGAGAGUGACGGGCCAGUc |
| NM_000026 | ADSL | circRNA | chr22 | 40754987 | 40755007 | + | 2 | 0 | AGO1-4 | 7mer-m8 | ucgccCUGAAACUCCCGGUCAa | ccacuGGCACUCA-GGCCAGUu |
| NM_022098 | XPNPEP3 | circRNA | chr22 | 41310299 | 41310320 | + | 3 | 0 | AGO1-4,AGO2 | 7mer-m8 | ucgcccUGAAACUCCCGGUCAa | caucacACGUACGUGGCCAGUc |
| NM_022098 | XPNPEP3 | circRNA | chr22 | 41350775 | 41350796 | + | 1 | 0 | AGO1-4 | 7mer-m8 | ucgcccugaaacucCCGGUCAa | gcagccauggccccGGCCAGUu |
| NM_001429 | EP300 | circRNA | chr22 | 41545070 | 41545091 | + | 3 | 0 | AGO1-4,AGO2 | 7mer-m8 | ucgcccugaaacUCCCGGUCAa | agccuuccaaccAGGGCCAGUu |
| NM_001098 | ACO2 | circRNA | chr22 | 41919255 | 41919276 | + | 1 | 0 | AGO1-4 | 7mer-m8 | ucgcccugaaacucCCGGUCAa | guggccccugcauuGGCCAGUg |
| NM_001018050 | POLR3H | circRNA | chr22 | 41924090 | 41924110 | - | 1 | 0 | AGO2 | 7mer-m8 | ucgcccUGAAACUCCCGGUCAa | gaagcuACCCUG-GGGCCAGUg |
| hsa_circ_0001237 | hsa_circ_002074 | circRNA | chr22 | 41924090 | 41924110 | - | 1 | 0 | AGO2 | 7mer-m8 | ucgcccUGAAACUCCCGGUCAa | gaagcuACCCUG-GGGCCAGUg |
| NM_015704 | PPPDE2 | circRNA | chr22 | 41996541 | 41996562 | - | 2 | 0 | AGO1-4,AGO2 | 7mer-m8 | ucgcccugaaacUCCCGGUCAa | cuuaccccaagcAAGGCCAGUg |
| NM_004599 | SREBF2 | circRNA | chr22 | 42301784 | 42301804 | + | 2 | 0 | AGO1-4 | 7mer-m8 | ucGCCCUGAAACUCCCGGUCAa | ucUGGGCCACUCA-GGCCAGUg |
| NM_005650 | TCF20 | circRNA | chr22 | 42610896 | 42610917 | - | 3 | 0 | AGO1,AGO1-4 | 7mer-m8 | ucgcCCUGAAACUCCCGGUCAa | gugaGGGUCAUGUGGGCCAGUu |
| NM_014246 | CELSR1 | circRNA | chr22 | 46790049 | 46790070 | - | 1 | 0 | AGO2 | 7mer-m8 | ucgcccugaaacUCCCGGUCAa | guaauaagaccaACGGCCAGUg |
| NM_025204 | TRABD | circRNA | chr22 | 50637073 | 50637094 | + | 7 | 1 | AGO1-4,AGO2 | 7mer-m8 | ucgcccugaaacuCCCGGUCAa | cgccugcccuccuGGGCCAGUc |
| NM_000487 | ARSA | circRNA | chr22 | 51061591 | 51061612 | - | 1 | 0 | AGO1-4 | 7mer-m8 | ucgcccugaaaCUCCCGGUCAa | gugcaugcccaGGUGGCCAGUc |
| hsa_circ_0089761 | hsa_circ_0089761 | circRNA | chrM | 9550 | 9572 | - | 7 | 0 | AGO1,AGO1-4 | 7mer-m8 | ucgccCUGA-AACUCCCGGUCAa | gugauGCCUGUUGGGGGCCAGUg |
| hsa_circ_0089763 | hsa_circ_0089763 | circRNA | chrM | 9550 | 9572 | - | 7 | 0 | AGO1,AGO1-4 | 7mer-m8 | ucgccCUGA-AACUCCCGGUCAa | gugauGCCUGUUGGGGGCCAGUg |
| NM_001669 | ARSD | circRNA | chrX | 2823541 | 2823562 | - | 1 | 0 | AGO1-4 | 7mer-m8 | ucgcccugaaacucCCGGUCAa | aauagcuaagauuuGGCCAGUg |
| NM_005647 | TBL1X | circRNA | chrX | 9679757 | 9679778 | + | 1 | 0 | AGO1-4 | 7mer-m8 | ucGCCCUGAAACUCCCGGUCAa | gaUGGGAAGUACUUGGCCAGUg |
| NM_015691 | WWC3 | circRNA | chrX | 10092327 | 10092348 | + | 1 | 0 | AGO1-4 | 8mer | ucgcccugaaacUCCCGGUCAa | gccuacggagacACGGCCAGUa |
| NM_004586 | RPS6KA3 | circRNA | chrX | 20185757 | 20185779 | - | 2 | 0 | AGO2 | 7mer-m8 | ucgcccugaaaCUC-CCGGUCAa | uuucucugaacGAGAGGCCAGUg |
| NM_015884 | MBTPS2 | circRNA | chrX | 21901997 | 21902018 | + | 3 | 0 | AGO1-4,AGO2 | 7mer-m8 | ucgCCCUGAAACUCCCGGUCAa | uugGGGAGUCAGCAGGCCAGUg |
| NM_004010 | DMD | circRNA | chrX | 31196057 | 31196076 | - | 1 | 0 | AGO2 | 8mer | ucgcccUGAAACUCCCGGUCAa | ugaucaACUU--CUGGCCAGUa |
| NM_004229 | MED14 | circRNA | chrX | 40525987 | 40526006 | - | 1 | 0 | AGO1-4 | 7mer-m8 | ucgcCCUGAAACUCCCGGUCAa | aauaGGAC--CAGGGGCCAGUu |
| NM_021140 | KDM6A | circRNA | chrX | 44913161 | 44913182 | + | 5 | 0 | AGO1-4,AGO2 | 7mer-m8 | ucgcccugaaacucCCGGUCAa | cagauccuaauucuGGCCAGUc |
| NM_014735 | PHF16 | circRNA | chrX | 46917811 | 46917832 | + | 2 | 0 | AGO1-4,AGO2 | 7mer-m8 | ucgcccugAAACUCCCGGUCAa | aauaaccgUUUGCUGGCCAGUc |
| NM_014735 | PHF16 | circRNA | chrX | 46920248 | 46920270 | + | 1 | 0 | AGO2 | 7mer-m8 | ucgcccugAAACUC-CCGGUCAa | auuccuuaUGUCAGAGGCCAGUg |
| NM_001204468 | RBM10 | circRNA | chrX | 47030589 | 47030610 | + | 1 | 1 | AGO2 | 8mer | ucgcccugaaaCUCCCGGUCAa | gaggaggaggaGAAGGCCAGUa |
| NM_001204468 | RBM10 | circRNA | chrX | 47044734 | 47044755 | + | 5 | 0 | AGO1-4,AGO2 | 7mer-m8 | ucgcccugaaacucCCGGUCAa | gaucucccgaaauuGGCCAGUg |
| NM_004651 | USP11 | circRNA | chrX | 47107024 | 47107046 | + | 9 | 0 | AGO1,AGO1-4,AGO2 | 7mer-m8 | ucgcccugaaacUC-CCGGUCAa | ugcaacaaggacAGCGGCCAGUg |
| NM_203475 | PORCN | circRNA | chrX | 48378795 | 48378817 | + | 1 | 1 | AGO2 | 7mer-m8 | ucGCCCUGAAAC-UCCCGGUCAa | guCAGAGCUCAGCUGGGCCAGUc |
| NM_006306 | SMC1A | circRNA | chrX | 53432263 | 53432287 | - | 1 | 0 | AGO1-4 | 7mer-m8 | ucgCCCU-GAAAC--UCCCGGUCAa | ggaGUGAUCUCUGGUGGGGCCAGUg |
| NM_031407 | HUWE1 | circRNA | chrX | 53600726 | 53600747 | - | 10 | 0 | AGO1-4,AGO2 | 7mer-m8 | ucgcccUGAAACUCCCGGUCAa | acagcuACACUGUGGGCCAGUc |
| NM_015107 | PHF8 | circRNA | chrX | 54020101 | 54020121 | - | 1 | 0 | AGO2 | 7mer-m8 | ucGCCCUGAAACUCCCGGUCAa | cuUGGGGC-CUGCUGGCCAGUu |
| NM_001184819 | GNL3L | circRNA | chrX | 54570715 | 54570736 | + | 6 | 0 | AGO1,AGO1-4,AGO2 | 8mer | ucGCCCUGAAACUCCCGGUCAa | acCGUGGCUUUCAAGGCCAGUa |
| NM_001184819 | GNL3L | circRNA | chrX | 54589923 | 54589942 | + | 2 | 0 | AGO2 | 7mer-m8 | ucGCCCUGAAACUCCCGGUCAa | uuUGGGAC--UGCAGGCCAGUu |
| NM_001184819 | GNL3L | circRNA | chrX | 54592533 | 54592557 | + | 2 | 0 | AGO1-4,AGO2 | 8mer | ucGCCCUGAAACUC---CCGGUCAa | caUGAGCCAUUGUGCCUGGCCAGUa |
| NM_015185 | ARHGEF9 | circRNA | chrX | 62858020 | 62858041 | - | 2 | 0 | AGO1-4 | 8mer | ucgcccugaaacUCCCGGUCAa | acccguuaaaccACGGCCAGUa |
| NM_020730 | DLG3 | circRNA | chrX | 69674113 | 69674134 | + | 1 | 0 | AGO1-4 | 7mer-m8 | ucgcccugaaacucCCGGUCAa | cucugaaacgggccGGCCAGUc |
| TCONS_00016926 | TCONS_00016926 | circRNA | chrX | 73046328 | 73046351 | - | 7 | 0 | AGO2 | 7mer-m8 | ucGCCCUGAAAC--UCCCGGUCAa | gaCAGUAUUAUGCCUGGGCCAGUc |
| TCONS_l2_00030712 | TCONS_l2_00030712 | circRNA | chrX | 73219297 | 73219318 | + | 1 | 0 | AGO2 | 8mer | ucgcccuGAAACUCCCGGUCAa | uccucacCUCUGGAGGCCAGUa |
| NM_000390 | CHM | circRNA | chrX | 85236782 | 85236803 | - | 4 | 0 | AGO1,AGO1-4 | 7mer-m8 | ucgcccugaaacuCCCGGUCAa | uauggaggaaacuGGGCCAGUu |
| NM_080737 | SYTL4 | circRNA | chrX | 99933565 | 99933586 | - | 1 | 0 | AGO1-4 | 8mer | ucgcccugaaacUCCCGGUCAa | cccaugaggaacAAGGCCAGUa |
| NM_022977 | ACSL4 | circRNA | chrX | 108921515 | 108921535 | - | 3 | 0 | AGO1-4,AGO2 | 7mer-m8 | ucGCCCUGAAACUCCCGGUCAa | agCUGGAAUGACA-GGCCAGUg |
| NM_145305 | SLC25A43 | circRNA | chrX | 118551018 | 118551039 | + | 1 | 0 | AGO1-4 | 7mer-m8 | ucgcccuGAAACUCCCGGUCAa | uccucccCAUAGUGGGCCAGUc |
| NM_145305 | SLC25A43 | circRNA | chrX | 118555227 | 118555245 | + | 1 | 0 | AGO1-4 | 7mer-m8 | ucgCCCUGAAACUCCCGGUCAa | guaGAGGCUGU---GGCCAGUu |
| NM_013995 | LAMP2 | circRNA | chrX | 119570639 | 119570662 | - | 7 | 0 | AGO1,AGO1-4,AGO2 | 7mer-m8 | ucgcCCUGAAA-CU-CCCGGUCAa | uuuuGUAUUUUACAUGGGCCAGUu |
| NM_001081550 | THOC2 | circRNA | chrX | 122778507 | 122778527 | - | 8 | 0 | AGO1-4,AGO2 | 7mer-m8 | ucgccCUGAAACUCCCGGUCAa | auaucGUCUGU-AUGGCCAGUg |
| NM_001042749 | STAG2 | circRNA | chrX | 123190032 | 123190053 | + | 7 | 0 | AGO1-4,AGO2 | 8mer | ucgcccugaaacucCCGGUCAa | uuauucagcucaccGGCCAGUa |
| NM_000276 | OCRL | circRNA | chrX | 128694540 | 128694562 | + | 6 | 0 | AGO1-4 | 7mer-m8 | ucgcCCUGAAAC-UCCCGGUCAa | acuuGGAAUGUGAAUGGCCAGUc |
| NM_001170704 | MBNL3 | circRNA | chrX | 131507519 | 131507540 | - | 2 | 0 | AGO1-4,AGO2 | 7mer-m8 | ucgcccugaaACUCCCGGUCAa | guccaauaccUCAGGGCCAGUu |
| NM_133486 | MBNL3 | circRNA | chrX | 131507519 | 131507540 | - | 2 | 0 | AGO1-4,AGO2 | 7mer-m8 | ucgcccugaaACUCCCGGUCAa | guccaauaccUCAGGGCCAGUu |
| NM_032458 | PHF6 | circRNA | chrX | 133561548 | 133561569 | + | 7 | 0 | AGO1,AGO2 | 7mer-m8 | ucgCCCUGAAACUCCCGGUCAa | ccgGGGGUAAUCAUGGCCAGUc |
| NM_173694 | ATP11C | circRNA | chrX | 138809037 | 138809058 | - | 1 | 0 | AGO2 | 7mer-m8 | ucgcccugaaacucCCGGUCAa | aagagcuaaaaacuGGCCAGUg |
| NM_001242614 | CD99L2 | circRNA | chrX | 149963919 | 149963939 | - | 1 | 0 | AGO1-4 | 8mer | ucgccCUGAAACUCCCGGUCAa | accacGACCAAGA-GGCCAGUa |
| hsa_circ_0001948 | hsa_circ_001345 | circRNA | chrX | 149963919 | 149963939 | - | 1 | 0 | AGO1-4 | 8mer | ucgccCUGAAACUCCCGGUCAa | accacGACCAAGA-GGCCAGUa |
| NM_000425 | L1CAM | circRNA | chrX | 153138063 | 153138083 | - | 1 | 0 | AGO2 | 7mer-m8 | ucgcccUGAAACUCCCGGUCAa | gccucaAGUGUGA-GGCCAGUg |
| NM_005334 | HCFC1 | circRNA | chrX | 153222891 | 153222912 | - | 9 | 0 | AGO1-4,AGO2 | 7mer-m8 | ucgcccugaaacucCCGGUCAa | aucacuaccacgcaGGCCAGUg |
| NM_005334 | HCFC1 | circRNA | chrX | 153236721 | 153236742 | - | 4 | 0 | AGO1-4,AGO2 | 7mer-m8 | ucgcccugaaacucCCGGUCAa | ccgccccggaagccGGCCAGUg |
| NM_001569 | IRAK1 | circRNA | chrX | 153276428 | 153276449 | - | 6 | 0 | AGO1-4,AGO2 | 7mer-m8 | ucgcccugaaacuCCCGGUCAa | caccuccaaccucGGGCCAGUg |
| NM_001110556 | FLNA | circRNA | chrX | 153586668 | 153586689 | - | 13 | 1 | AGO1,AGO1-4,AGO2,AGO3 | 7mer-m8 | ucgcccugaaACUCCCGGUCAa | gccagcaaggUGAAGGCCAGUg |
| NM_000402 | G6PD | circRNA | chrX | 153761285 | 153761306 | - | 3 | 1 | AGO1-4,AGO2 | 8mer | ucgcccugaaacuCCCGGUCAa | acaaugugguccuGGGCCAGUa |
| NM_001042351 | G6PD | circRNA | chrX | 153761285 | 153761306 | - | 3 | 1 | AGO1-4,AGO2 | 8mer | ucgcccugaaacuCCCGGUCAa | acaaugugguccuGGGCCAGUa |
| NM_000402 | G6PD | circRNA | chrX | 153763533 | 153763554 | - | 6 | 0 | AGO1-4,AGO2 | 8mer | ucgcccUGAAACUCCCGGUCAa | acuccuAUGUGGCUGGCCAGUa |
| NM_001042351 | G6PD | circRNA | chrX | 153763533 | 153763554 | - | 6 | 0 | AGO1-4,AGO2 | 8mer | ucgcccUGAAACUCCCGGUCAa | acuccuAUGUGGCUGGCCAGUa |
| NM_018196 | TMLHE | circRNA | chrX | 154736600 | 154736623 | - | 1 | 2 | AGO2 | 7mer-m8 | ucgccCUGAAACU--CCCGGUCAa | cacauGAUUGGGAUUGGGCCAGUc |

Supplemental Table S3. Predicted long non-coding RNAs that target miR-193b-3p based on the starBase tool (<https://starbase.sysu.edu.cn/>).

| geneID | geneName | geneType | chromosome | start | end | strand | clipExpNum | degraExpNum | RBP | merClass | miRseq | targetSeq | pancancerNum |
| --- | --- | --- | --- | --- | --- | --- | --- | --- | --- | --- | --- | --- | --- |
| ENSG00000279443 | AL513497.1 | TEC | chr1 | 28872504 | 28872525 | + | 1 | 0 | AGO2 | 8mer | ucgcccugaaacucCCGGUCAa | gagccaccaugcccGGCCAGUa | 5 |
| ENSG00000259943 | AL050341.2 | antisense | chr1 | 40723265 | 40723286 | - | 1 | 0 | AGO1-4 | 8mer | ucgcCCUGAAACUCCCGGUCAa | aaauGGAGUCUUUUGGCCAGUa | 15 |
| ENSG00000234497 | ERICH3-AS1 | antisense | chr1 | 75090602 | 75090626 | + | 1 | 0 | AGO1-4 | 7mer-m8 | ucGCCCUGAA---ACUCCCGGUCAa | auUGGGUCUUGUAUGUUGGCCAGUc | 3 |
| ENSG00000226822 | AL390036.1 | lincRNA | chr1 | 1.09E+08 | 1.09E+08 | + | 1 | 0 | AGO1-4 | 7mer-m8 | ucgcccugaaacUCCCGGUCAa | aaguucaaucacAUGGCCAGUg | 3 |
| ENSG00000236943 | RP11-640M9.1 | antisense | chr1 | 1.45E+08 | 1.45E+08 | - | 1 | 0 | AGO1-4 | 7mer-m8 | ucGCCCUGAAACUCCCGGUCAa | gcUGAGUCCCUG-GGGCCAGUg | 0 |
| ENSG00000235919 | ASH1L-AS1 | antisense | chr1 | 1.56E+08 | 1.56E+08 | + | 1 | 0 | AGO1-4 | 8mer | ucgCCCUGAAACUCCCGGUCAa | guaGGGA--GUGA-GGCCAGUa | 8 |
| ENSG00000230630 | DNM3OS | antisense | chr1 | 1.72E+08 | 1.72E+08 | - | 1 | 0 | AGO2 | 8mer | ucgccCUGA--AACUCCCGGUCAa | acucuGACUACAUGUGGGCCAGUa | 9 |
| ENSG00000243155 | AL162431.2 | antisense | chr1 | 1.81E+08 | 1.81E+08 | - | 4 | 0 | AGO1-4 | 7mer-m8 | ucGCCCUGAAACUCCCGGUCAa | ccCAGCGCUGGAAGGGCCAGUg | 7 |
| ENSG00000232077 | LINC01031 | lincRNA | chr1 | 1.93E+08 | 1.93E+08 | + | 1 | 0 | AGO2 | 8mer | ucGCCCUGAAACU-C-CCGGUCAa | ccCAGGA-UUGGACGAGGCCAGUa | 5 |
| ENSG00000203709 | MIR29B2CHG | lincRNA | chr1 | 2.08E+08 | 2.08E+08 | - | 2 | 0 | AGO1-4 | 8mer | ucgcccUGAAACUCCCGGUCAa | acuacaGCAUUCUUGGCCAGUa | 13 |
| ENSG00000278908 | AC132154.1 | TEC | chr2 | 30900798 | 30900822 | + | 1 | 0 | AGO2 | 7mer-m8 | ucgcccugAAACU---CCCGGUCAa | uggcuucaUUUGGUUCUGGCCAGUg | 7 |
| ENSG00000279873 | LINC01126 | lincRNA | chr2 | 43455968 | 43455990 | + | 1 | 0 | AGO1-4 | 7mer-m8 | ucGCCCUGAAAC-UCCCGGUCAa | agCUGGGCUUUGUUGGGCCAGUu | 13 |
| ENSG00000212978 | AC016747.1 | processed_transcript | chr2 | 61370706 | 61370727 | - | 1 | 0 | AGO2 | 7mer-m8 | ucgcccuGAAACUCCCGGUCAa | gaaguuuCUUUCGGGGCCAGUg | 4 |
| ENSG00000281195 | AC007878.1 | lincRNA | chr2 | 71603093 | 71603117 | + | 1 | 0 | AGO2 | 7mer-m8 | ucGCCCUGA-AACUC--CCGGUCAa | uuUGGAAUUGUUGAGAAGGCCAGUu | 8 |
| ENSG00000222041 | CYTOR | lincRNA | chr2 | 87820960 | 87820982 | + | 5 | 0 | AGO1-4,AGO2 | 7mer-m8 | ucgcccugAAAC-UCCCGGUCAa | guuucccaUUUGUCUGGCCAGUc | 1 |
| ENSG00000172965 | MIR4435-2HG | lincRNA | chr2 | 1.12E+08 | 1.12E+08 | - | 8 | 0 | AGO1-4,AGO2 | 7mer-m8 | ucgcccugAAAC-UCCCGGUCAa | guuucccaUUUGUCUGGCCAGUc | 3 |
| ENSG00000285016 | AC017002.6 | processed_transcript | chr2 | 1.12E+08 | 1.12E+08 | - | 8 | 0 | AGO1-4,AGO2 | 7mer-m8 | ucgcccugAAAC-UCCCGGUCAa | guuucccaUUUGUCUGGCCAGUc | 0 |
| ENSG00000236859 | NIFK-AS1 | antisense | chr2 | 1.22E+08 | 1.22E+08 | + | 2 | 0 | AGO1-4,AGO2 | 7mer-m8 | ucgcccUGAAACUC-CCGGUCAa | auauuuACCUCGUGCGGCCAGUc | 14 |
| ENSG00000279598 | AC009948.5 | TEC | chr2 | 1.79E+08 | 1.79E+08 | + | 1 | 0 | AGO2 | 7mer-m8 | ucgcccugaaacUCCCGGUCAa | acuaauaacaacAGGGCCAGUu | 2 |
| ENSG00000272644 | AC097468.3 | lincRNA | chr2 | 2.2E+08 | 2.2E+08 | - | 1 | 0 | AGO1-4 | 7mer-m8 | ucgcccugaaacucCCGGUCAa | acccaaaggagccaGGCCAGUc | 4 |
| ENSG00000227308 | AC009502.1 | lincRNA | chr2 | 2.21E+08 | 2.21E+08 | + | 1 | 0 | AGO1-4 | 7mer-m8 | ucgccCUGAAACUC--CCGGUCAa | gccaaGGUUUCCAGGAGGCCAGUc | 3 |
| ENSG00000230530 | LIMD1-AS1 | bidirectional_promoter_lncRNA | chr3 | 45730555 | 45730576 | - | 24 | 0 | AGO1,AGO1-4,AGO2 | 7mer-m8 | ucgcccugaaACUCCCGGUCAa | cuuuagcaagUCGGGGCCAGUg | 15 |
| ENSG00000213600 | U73169.1 | sense_intronic | chr3 | 50277357 | 50277378 | + | 1 | 0 | AGO1-4 | 7mer-m8 | ucgcccugaaacucCCGGUCAa | agaguccugguacuGGCCAGUg | 4 |
| ENSG00000249592 | AC139887.2 | antisense | chr4 | 756330 | 756351 | - | 2 | 0 | AGO1-4 | 7mer-m8 | ucgcccugaaACUCCCGGUCAa | ugagaacacaUGCUGGCCAGUc | 8 |
| ENSG00000226950 | DANCR | processed_transcript | chr4 | 53579169 | 53579190 | + | 1 | 0 | AGO1-4 | 7mer-m8 | ucgcccugaaacucCCGGUCAa | agccuggagcacguGGCCAGUg | 12 |
| ENSG00000188242 | AC010442.1 | 3prime_overlapping_ncRNA | chr5 | 472538 | 472559 | - | 2 | 0 | AGO1-4 | 7mer-m8 | ucgcccugaaACUCCCGGUCAa | uugccaagaaUCACGGCCAGUc | 9 |
| ENSG00000245937 | LINC01184 | lincRNA | chr5 | 1.27E+08 | 1.27E+08 | - | 12 | 0 | AGO1,AGO1-4,AGO2 | 8mer | ucgcccuGAAACUCCCGGUCAa | cugucuuCUUU-AUGGCCAGUa | 11 |
| ENSG00000231074 | HCG18 | antisense | chr6 | 30294247 | 30294268 | - | 3 | 0 | AGO1,AGO1-4 | 7mer-m8 | ucgcccuGAAACUCCCGGUCAa | uuuuccuCUCUUCGGGCCAGUc | 12 |
| ENSG00000228506 | AL513550.1 | antisense | chr6 | 99873600 | 99873621 | + | 1 | 0 | AGO2 | 7mer-m8 | ucgcccugaaacucCCGGUCAa | accaaaccaauucuGGCCAGUu | 15 |
| ENSG00000243004 | AC005062.1 | sense_overlapping | chr7 | 20180067 | 20180076 | - | 2 | 0 | AGO2 | 7mer-m8 | ucgcccugaaacucCCGGUCAa | #NAME? | 14 |
| ENSG00000272905 | AC018648.1 | lincRNA | chr7 | 32885521 | 32885542 | + | 3 | 0 | AGO1-4 | 7mer-m8 | ucgcccugaaacucCCGGUCAa | auaguguuaaaauaGGCCAGUg | 7 |
| ENSG00000232956 | SNHG15 | lincRNA | chr7 | 45024819 | 45024840 | - | 1 | 0 | AGO1-4 | 7mer-m8 | ucgcccugaaaCUCCCGGUCAa | caguagccacaGGUGGCCAGUg | 5 |
| ENSG00000274272 | AC069281.2 | processed_transcript | chr7 | 1E+08 | 1E+08 | - | 1 | 0 | AGO2 | 7mer-m8 | ucGCCCUGAAACUCCCGGUCAa | gcCGAGGCUCU---GGCCAGUg | 10 |
| ENSG00000272219 | AC005072.1 | sense_intronic | chr7 | 1.02E+08 | 1.02E+08 | + | 1 | 0 | AGO1-4 | 7mer-m8 | ucgcccugaaACUCCCGGUCAa | aaauucucaaUGAGGGCCAGUu | 4 |
| ENSG00000273329 | AC078846.1 | lincRNA | chr7 | 1.29E+08 | 1.29E+08 | - | 3 | 0 | AGO1-4 | 8mer | ucGCCCUGAAACUCCCGGUCAa | auCAGGG-UCUCA-GGCCAGUa | 8 |
| ENSG00000260231 | JHDM1D-AS1 | bidirectional_promoter_lncRNA | chr7 | 1.4E+08 | 1.4E+08 | + | 1 | 0 | AGO1-4 | 7mer-m8 | ucGCCCUGAAACUCCCGGUCAa | ucUGUGACCU--CGGGCCAGUc | 8 |
| ENSG00000279608 | AL353795.3 | TEC | chr9 | 35042728 | 35042749 | - | 2 | 0 | AGO1-4 | 7mer-m8 | ucgcccUGAAACUCCCGGUCAa | aaucccACGUGUGUGGCCAGUc | 7 |
| ENSG00000281649 | EBLN3P | lincRNA | chr9 | 37087247 | 37087269 | + | 2 | 0 | AGO1-4 | 7mer-m8 | ucgcccugaaAC-UCCCGGUCAa | ucaguuauaaUGCAGGGCCAGUg | 12 |
| ENSG00000259953 | AL138756.1 | sense_overlapping | chr9 | 1.15E+08 | 1.15E+08 | - | 1 | 0 | AGO1-4 | 7mer-m8 | ucgcccugaAACU-CCCGGUCAa | uuuugcuugUUGAUUGGCCAGUu | 8 |
| ENSG00000224842 | AL161908.1 | antisense | chr9 | 1.29E+08 | 1.29E+08 | - | 1 | 0 | AGO1-4 | 7mer-m8 | ucGCCCUGAAACUCCCGGUCAa | gaUGUGAGGCUG-GGGCCAGUg | 7 |
| ENSG00000225361 | PPP1R26-AS1 | antisense | chr9 | 1.38E+08 | 1.38E+08 | - | 1 | 0 | AGO1-4 | 8mer | ucgcCCUGAAACUCCCGGUCAa | aaaaGGAGGUU---GGCCAGUa | 9 |
| ENSG00000233016 | SNHG7 | antisense | chr9 | 1.4E+08 | 1.4E+08 | - | 4 | 0 | AGO2 | 7mer-m8 | ucgcccugaaacucCCGGUCAa | ugacuucuccucccGGCCAGUu | 9 |
| ENSG00000226688 | ENTPD1-AS1 | antisense | chr10 | 97779019 | 97779040 | - | 1 | 0 | AGO1-4 | 7mer-m8 | ucgcccugaaacucCCGGUCAa | cugcuuauaacauuGGCCAGUu | 15 |
| ENSG00000130600 | H19 | processed_transcript | chr11 | 2018870 | 2018889 | - | 4 | 0 | AGO1-4 | 7mer-m8 | ucGCCCUGAAACUCCCGGUCAa | ggUGGGGC-CUGA-GGCCAGUg | 4 |
| ENSG00000269821 | KCNQ1OT1 | antisense | chr11 | 2682968 | 2682989 | - | 1 | 0 | AGO1-4 | 7mer-m8 | ucgcCCUGAAACUCCCGGUCAa | cugaGGAGAAUAAAGGCCAGUg | 7 |
| ENSG00000269821 | KCNQ1OT1 | antisense | chr11 | 2687217 | 2687237 | - | 1 | 0 | AGO1-4 | 7mer-m8 | ucGCCCUGAAACUCCCGGUCAa | cgUGUGAAUUCCA-GGCCAGUc | 7 |
| ENSG00000269821 | KCNQ1OT1 | antisense | chr11 | 2691977 | 2691998 | - | 1 | 0 | AGO1-4 | 7mer-m8 | ucgcccugaaacucCCGGUCAa | ccacaagagccacuGGCCAGUg | 7 |
| ENSG00000269821 | KCNQ1OT1 | antisense | chr11 | 2692085 | 2692106 | - | 4 | 0 | AGO1-4,AGO2 | 7mer-m8 | ucgcccugaaacucCCGGUCAa | cccaguggcccacuGGCCAGUg | 7 |
| ENSG00000246273 | SBF2-AS1 | antisense | chr11 | 9829962 | 9829983 | + | 1 | 0 | AGO1-4 | 7mer-m8 | ucgcccuGAAACUCCCGGUCAa | uuuaaucCUUAACUGGCCAGUu | 7 |
| ENSG00000255448 | AC055860.1 | processed_transcript | chr11 | 18026037 | 18026055 | - | 3 | 0 | AGO2 | 7mer-m8 | ucgcccuGAAACUCCCGGUCAa | ccaacucCUUU---GGCCAGUu | 2 |
| ENSG00000256341 | AP006333.1 | antisense | chr11 | 63886207 | 63886229 | - | 1 | 0 | AGO1-4 | 7mer-m8 | ucgcccugaaaCUC-CCGGUCAa | gggucgccagcGAGUGGCCAGUg | 8 |
| ENSG00000229719 | MIR194-2HG | lincRNA | chr11 | 64658825 | 64658843 | - | 13 | 0 | AGO1,AGO1-4,AGO2,AGO3 | 8mer | ucGCCCUGAAACUCCCGGUCAa | ucUGGGGC---GAGGGCCAGUa | 7 |
| ENSG00000245532 | NEAT1 | lincRNA | chr11 | 65191460 | 65191486 | + | 22 | 1 | AGO1-4,AGO2 | 7mer-m8 | ucgCCCUGAAACUC-----CCGGUCAa | cggGAGACAUGGAGUCCCUGGCCAGUg | 4 |
| ENSG00000245532 | NEAT1 | lincRNA | chr11 | 65204008 | 65204029 | + | 9 | 0 | AGO1-4 | 7mer-m8 | ucgcccUGAAACUCCCGGUCAa | gaggccACCGUCAUGGCCAGUu | 4 |
| ENSG00000245532 | NEAT1 | lincRNA | chr11 | 65211182 | 65211203 | + | 6 | 0 | AGO1-4,AGO2 | 7mer-m8 | ucgcccugaaacUCCCGGUCAa | uggccagagggaAGGGCCAGUc | 4 |
| ENSG00000255517 | AP002748.3 | antisense | chr11 | 66241972 | 66241994 | - | 2 | 0 | AGO1-4 | 7mer-m8 | ucgcCCUGAAAC-UCCCGGUCAa | agaaGAGCAGUGCAUGGCCAGUu | 6 |
| ENSG00000247137 | AP000873.2 | processed_transcript | chr11 | 82902275 | 82902296 | - | 1 | 0 | AGO1-4 | 7mer-m8 | ucgcccugaaACUCCCGGUCAa | gaguuauucaUGUGGGCCAGUu | 6 |
| ENSG00000279696 | AP001273.1 | TEC | chr11 | 93461855 | 93461876 | - | 2 | 0 | AGO2 | 7mer-m8 | ucgcccugaaacUCCCGGUCAa | uuccauuauaccAUGGCCAGUu | 12 |
| ENSG00000280237 | MIR4697HG | TEC | chr11 | 1.34E+08 | 1.34E+08 | - | 4 | 0 | AGO1-4,AGO2 | 7mer-m8 | ucGCCCUGAAACUC-CCGGUCAa | ugCUGUUCUCGAAGAGGCCAGUu | 18 |
| ENSG00000282977 | PCBP2-OT1 | non_coding | chr12 | 53858695 | 53858716 | + | 2 | 0 | AGO1-4,AGO2 | 7mer-m8 | ucgcccUGAAACUCCCGGUCAa | gccaacACACGGGGGGCCAGUg | 0 |
| ENSG00000257379 | AC023509.1 | processed_transcript | chr12 | 53861111 | 53861133 | + | 3 | 0 | AGO1-4 | 7mer-m8 | ucgcccugaaaCUC-CCGGUCAa | guagugcaagaGAGAGGCCAGUc | 12 |
| ENSG00000255737 | AGAP2-AS1 | antisense | chr12 | 58121671 | 58121695 | + | 1 | 0 | AGO1-4 | 7mer-m8 | ucgCCC--UGAAACUC-CCGGUCAa | ccaGGGUCAGUUCCUGCGGCCAGUc | 6 |
| ENSG00000257242 | LINC01619 | processed_transcript | chr12 | 92387219 | 92387240 | - | 2 | 0 | AGO2 | 8mer | ucgcccugaaacucCCGGUCAa | cgaggaaccaaauuGGCCAGUa | 13 |
| ENSG00000245017 | LINC02453 | lincRNA | chr12 | 98880363 | 98880389 | - | 1 | 0 | AGO1-4 | 7mer-m8 | ucGCCCUGAA-----ACUCCCGGUCAa | caCGGGGUUUCGCCAUGUUGGCCAGUc | 14 |
| ENSG00000281344 | HELLPAR | macro_lncRNA | chr12 | 1.03E+08 | 1.03E+08 | + | 1 | 0 | AGO2 | 7mer-m8 | ucgcccugaaacUCCCGGUCAa | uugauccccaaaAGGGCCAGUu | 6 |
| ENSG00000257279 | AC127164.1 | antisense | chr12 | 1.17E+08 | 1.17E+08 | - | 1 | 0 | AGO2 | 7mer-m8 | ucgcccugaaaCUCCCGGUCAa | acaugaguaacGAUGGCCAGUc | 5 |
| ENSG00000280287 | AC131212.3 | TEC | chr12 | 1.33E+08 | 1.33E+08 | + | 2 | 0 | AGO1,AGO2 | 7mer-m8 | ucgcccugaaacuCCCGGUCAa | aaaaugugcaauuGGGCCAGUu | 3 |
| ENSG00000257285 | AL132780.1 | antisense | chr14 | 23398897 | 23398918 | + | 22 | 0 | AGO1,AGO1-4,AGO2 | 7mer-m8 | ucgcccugAAACUCCCGGUCAa | cagugccaUCUCAGGGCCAGUg | 8 |
| ENSG00000257636 | G2E3-AS1 | antisense | chr14 | 30919664 | 30919685 | - | 1 | 0 | AGO1-4 | 8mer | ucgcccugaaacucCCGGUCAa | aaggcaacaagccaGGCCAGUa | 1 |
| ENSG00000258940 | AL132639.2 | antisense | chr14 | 39735674 | 39735695 | - | 1 | 0 | AGO1-4 | 7mer-m8 | ucgcccuGAAACUCCCGGUCAa | ucaguucCAUUAGCGGCCAGUu | 6 |
| ENSG00000259065 | AC005520.2 | antisense | chr14 | 74269659 | 74269680 | + | 1 | 0 | AGO1-4 | 8mer | ucgcccugaaacucCCGGUCAa | cagcagcagccacaGGCCAGUa | 8 |
| ENSG00000260711 | AL121839.2 | sense_intronic | chr14 | 92223860 | 92223880 | - | 1 | 0 | AGO2 | 7mer-m8 | ucgcccUGAAACUCCCGGUCAa | ugagcuAUUGUUA-GGCCAGUu | 11 |
| ENSG00000224078 | SNHG14 | processed_transcript | chr15 | 25344389 | 25344410 | + | 1 | 0 | AGO2 | 7mer-m8 | ucgcccuGAAACUCCCGGUCAa | cucccauCCUCAAGGGCCAGUg | 14 |
| ENSG00000259642 | ST20-AS1 | antisense | chr15 | 80217741 | 80217762 | + | 1 | 0 | AGO1-4 | 7mer-m8 | ucgcccugaaacucCCGGUCAa | gagccaccgcgccuGGCCAGUg | 9 |
| ENSG00000279162 | AC141586.5 | TEC | chr16 | 2644131 | 2644152 | + | 1 | 0 | AGO1-4 | 7mer-m8 | ucgcccugaaacucCCGGUCAa | ugugcccccaacucGGCCAGUc | 7 |
| ENSG00000261067 | AC109460.3 | processed_transcript | chr16 | 28986516 | 28986535 | + | 1 | 0 | AGO1-4 | 7mer-m8 | ucGCCCUGAAACU-CCCGGUCAa | acCCGGAC---GACGGGCCAGUg | 7 |
| ENSG00000261067 | AC109460.3 | processed_transcript | chr16 | 28995588 | 28995609 | + | 1 | 0 | AGO2 | 7mer-m8 | ucgcccugaaacucCCGGUCAa | acccgcgugcccguGGCCAGUg | 7 |
| ENSG00000278133 | AC135050.6 | sense_intronic | chr16 | 31135252 | 31135274 | + | 10 | 0 | AGO1,AGO1-4,AGO2,AGO3 | 7mer-m8 | ucgCCCUGAAACU-CCCGGUCAa | gcaGGGUGGAUAACGGGCCAGUg | 11 |
| ENSG00000245694 | CRNDE | lincRNA | chr16 | 54962948 | 54962972 | - | 1 | 0 | AGO1-4 | 7mer-m8 | ucgCCCU-GAAACUC--CCGGUCAa | uggGGGAGCGCGCAGCCGGCCAGUg | 7 |
| ENSG00000266962 | AC067852.2 | antisense | chr17 | 40706139 | 40706158 | - | 3 | 0 | AGO1-4,AGO2 | 7mer-m8 | ucGCCCUGAAACUCCCGGUCAa | ugUGUGACCUU--GGGCCAGUc | 13 |
| ENSG00000267121 | AC008105.3 | antisense | chr17 | 43298578 | 43298602 | - | 1 | 0 | AGO2 | 7mer-m8 | ucgCCCUGAAACU-C--CCGGUCAa | uggGGGGCGCGGACGCAGGCCAGUg | 6 |
| ENSG00000279281 | AC015883.1 | TEC | chr17 | 55526430 | 55526451 | + | 1 | 0 | AGO1-4 | 7mer-m8 | ucgcccugaaacUCCCGGUCAa | auaucaaacuccAAGGCCAGUg | 8 |
| ENSG00000267416 | AC025048.4 | lincRNA | chr17 | 58165888 | 58165909 | + | 1 | 0 | AGO1-4 | 7mer-m8 | ucgcccugaaacucCCGGUCAa | agacuccuacaccaGGCCAGUu | 3 |
| ENSG00000264548 | AC132872.2 | antisense | chr17 | 80174788 | 80174811 | + | 1 | 0 | AGO2 | 7mer-m8 | ucgccCUGAAACU--CCCGGUCAa | gcuccGAUCUGGAUCUGGCCAGUg | 6 |
| ENSG00000279744 | AC132938.5 | TEC | chr17 | 80421415 | 80421437 | + | 1 | 0 | AGO2 | 7mer-m8 | ucGCCCU-GAAACUCCCGGUCAa | ggCGAGAGCAGCAAGGGCCAGUg | 12 |
| ENSG00000275162 | AC005391.1 | sense_intronic | chr19 | 956624 | 956647 | + | 1 | 0 | AGO2 | 7mer-m8 | ucGCCCUGAAACUC--CCGGUCAa | cgCAGGAAUGCAGGCUGGCCAGUc | 4 |
| ENSG00000275234 | AC010503.4 | antisense | chr19 | 6469577 | 6469598 | + | 1 | 0 | AGO2 | 7mer-m8 | ucgcccugaaacucCCGGUCAa | gagccacugcacccGGCCAGUu | 14 |
| ENSG00000274425 | AC114271.1 | antisense | chr19 | 10444649 | 10444669 | + | 3 | 0 | AGO1-4 | 8mer | ucgcCCUGAAACUCCCGGUCAa | agacGUACAUU-AAGGCCAGUa | 5 |
| ENSG00000236144 | TMEM147-AS1 | antisense | chr19 | 36032438 | 36032457 | - | 2 | 0 | AGO1-4,AGO2 | 7mer-m8 | ucGCCCUGAAACUCCCGGUCAa | ucUGUGAUUCU--UGGCCAGUg | 5 |
| ENSG00000267309 | AC092295.2 | antisense | chr19 | 36981274 | 36981295 | + | 12 | 0 | AGO1,AGO1-4,AGO2 | 8mer | ucgCCCUGAAACUCCCGGUCAa | uaaGUGUAUCUCAUGGCCAGUa | 11 |
| ENSG00000276570 | AC010327.5 | processed_transcript | chr19 | 55741332 | 55741356 | - | 4 | 0 | AGO1-4,AGO2 | 7mer-m8 | ucGCCCUGAAACU---CCCGGUCAa | ccCUGCACUUUCAACCAGGCCAGUc | 5 |
| ENSG00000270001 | AL121894.2 | lincRNA | chr20 | 23612661 | 23612681 | - | 2 | 0 | AGO1-4,AGO2 | 7mer-m8 | ucgcccugaaACUCCCGGUCAa | ugggcccccaUGA-GGCCAGUg | 7 |
| ENSG00000177410 | ZFAS1 | antisense | chr20 | 47897273 | 47897292 | + | 23 | 0 | AGO1-4,AGO2 | 7mer-m8 | ucgcccUGAAACUCCCGGUCAa | cucuacACUAU--UGGCCAGUu | 9 |
| ENSG00000272675 | AC004019.18 | lincRNA | chr22 | 18037606 | 18037626 | + | 8 | 0 | AGO1,AGO1-4,AGO2 | 8mer | ucgcCCUGAAACUCCCGGUCAa | auguGAACCUU-CUGGCCAGUa | 0 |
| ENSG00000185065 | AC000068.1 | antisense | chr22 | 19435551 | 19435569 | + | 1 | 0 | AGO2 | 7mer-m8 | ucgccCUGAAACUCCCGGUCAa | cacacGGCUCU---GGCCAGUu | 6 |
| ENSG00000285314 | AC002470.2 | processed_transcript | chr22 | 21342359 | 21342383 | + | 1 | 0 | AGO2 | 7mer-m8 | ucgcccUGAAAC--U-CCCGGUCAa | aauacaAGUUUGCAACUGGCCAGUg | 0 |
| ENSG00000285314 | AC002470.2 | processed_transcript | chr22 | 21344812 | 21344833 | + | 1 | 0 | AGO1-4 | 7mer-m8 | ucGCCCUGAAACUCCCGGUCAa | gaCGUGAGUACUCUGGCCAGUg | 0 |
| ENSG00000278948 | AL031587.5 | TEC | chr22 | 38339747 | 38339768 | + | 3 | 0 | AGO1-4,AGO2 | 7mer-m8 | ucgcccUGAAACUCCCGGUCAa | aaauauGUUUUAUUGGCCAGUu | 11 |
| ENSG00000261251 | Z97055.2 | antisense | chr22 | 44209400 | 44209424 | + | 1 | 0 | AGO1-4 | 7mer-m8 | ucgCCCUGA-AA-CUC-CCGGUCAa | agaGGGUCUCUUACAGAGGCCAGUc | 9 |
| ENSG00000234869 | AL021392.1 | antisense | chr22 | 46937587 | 46937608 | + | 1 | 0 | AGO1-4 | 7mer-m8 | ucgcccUGAAACUCCCGGUCAa | acaguaAAUUCCUGGGCCAGUu | 5 |
| ENSG00000229807 | XIST | lincRNA | chrX | 73046328 | 73046351 | - | 7 | 0 | AGO2 | 7mer-m8 | ucGCCCUGAAAC--UCCCGGUCAa | gaCAGUAUUAUGCCUGGGCCAGUc | 3 |
| ENSG00000225470 | JPX | lincRNA | chrX | 73219297 | 73219318 | + | 1 | 0 | AGO2 | 8mer | ucgcccuGAAACUCCCGGUCAa | uccucacCUCUGGAGGCCAGUa | 5 |
| ENSG00000271533 | Z83843.1 | sense_intronic | chrX | 73432467 | 73432488 | - | 2 | 0 | AGO1-4 | 7mer-m8 | ucgccCUGAAACUCCCGGUCAa | ccuauGGCUUACUAGGCCAGUu | 5 |
| ENSG00000235703 | LINC00894 | antisense | chrX | 1.49E+08 | 1.49E+08 | + | 1 | 0 | AGO2 | 8mer | ucgcccUGAAACUCCCGGUCAa | ugauucACUU--GUGGCCAGUa | 6 |
